# Supplementary material for: Characterization of Bulk Phosphatidylcholine Compositions in Human Plasma Using Side-Chain Resolving Lipidomics
Source: Metabolites. 2019 Jun 8;9(6):109. doi: 10.3390/metabo9060109 (PMC6631474; doi:10.3390/metabo9060109)
Supplement: Supplementary file 1 [file metabolites-09-00109-s001.zip › Supplementary_Figure_S1.pdf]

PC aa C28:1 = R

No independent Variable measured.

Qualitative composition

PC aa C28:1 consists of:
PC 10:0_18:1, PC 12:0_16:1, PC 13:0_15:1, PC 14:0_14:1,
[13C1]SM 32:1
and further compounds

PC aa C30:0 = PC 16:0_14:0 + PC 18:0_12:0 + R

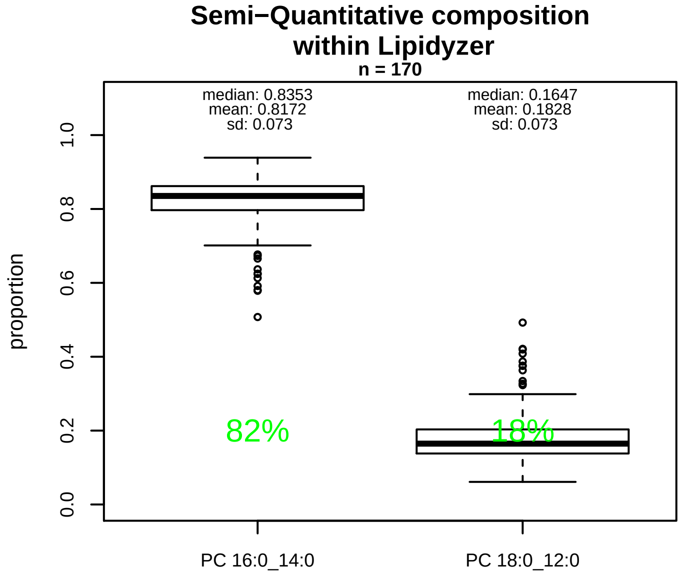

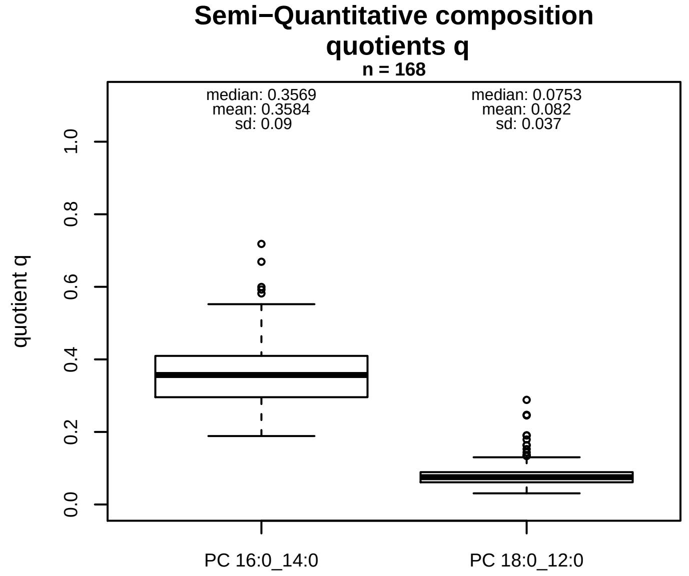

Semi−Quantitative composition: Lipidyzer

PC aa C30:0 consists of:
PC 16:0_14:0 81.7%
PC 18:0_12:0 18.3%
and of (not quantified):
PC 10:0_20:0, PC 11:0_19:0, PC 13:0_17:0, PC 15:0_15:0,
PC 9:0_21:0, PC O−16:0_15:0, PC O−18:0_13:0, [13C1]SM 3
and further compounds

Linear model

```
PC aa C30:0 ~ b * ( PC 16:0_14:0
   + PC 18:0_12:0 )
```

b = 1.30545

R^2 = 0.83347

Ranges

Measure	AbsoluteIDQ	sum(Lipidyzer)	delta
Min	2.17	0.6	1.57
Max	7.97	4.8	3.17
Mean	3.77	1.85	1.91
Median	3.42	1.65	1.77
SD	1.25	0.89	0.36

Composition: mean of proportions q

conc(PC aa C30:0) * 0.3584 = conc(PC 16:0_14:0) [var(q)=0.3584]
Percentiles: 5%−>0.2248, 25%−>0.2958, 75%−>0.4096, 95%−>0.5098
conc(PC aa C30:0) * 0.082 = conc(PC 18:0_12:0) [var(q)=0.082]
Percentiles: 5%−>0.0462, 25%−>0.0612, 75%−>0.089, 95%−>0.1495

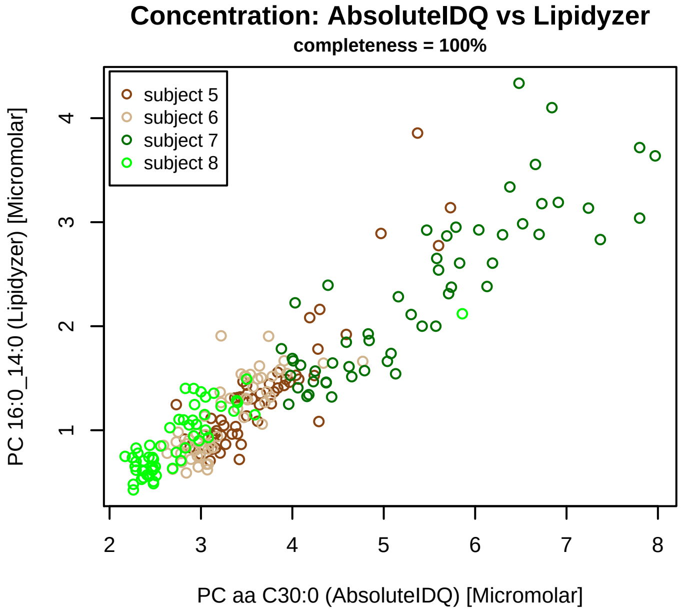

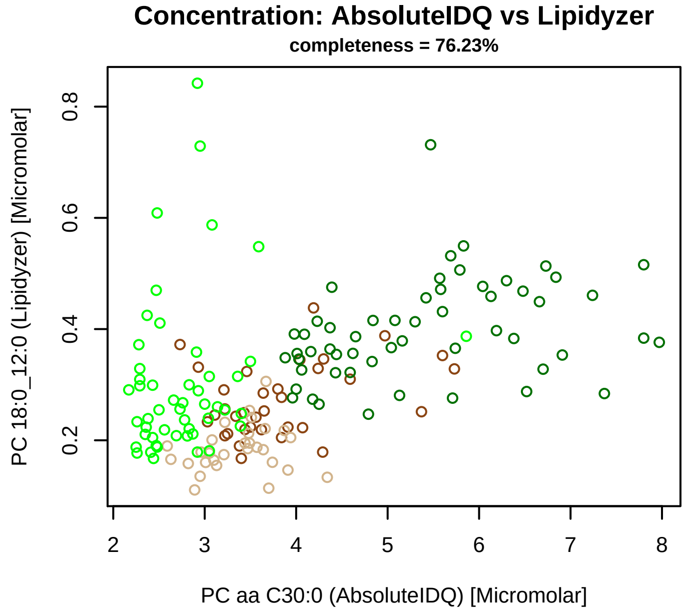

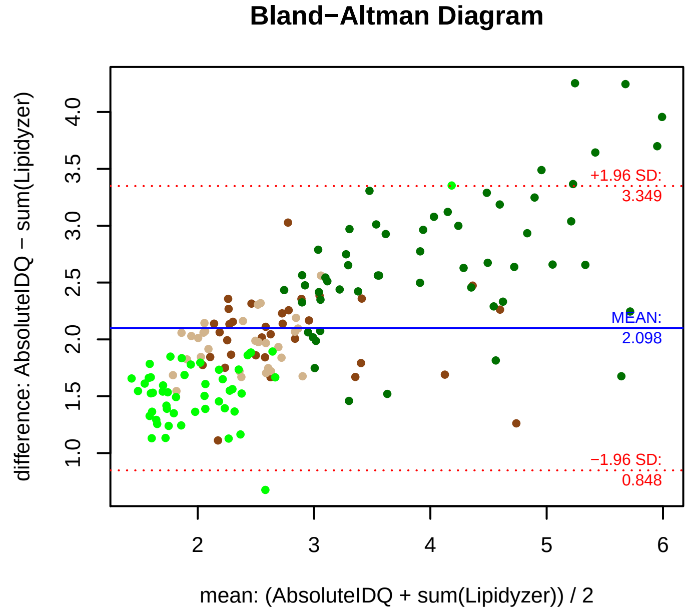

Stability of composition

(PC 16:0_14:0 + PC 18:0_12:0) / PC aa C30:0
Shapiro−Wilk Test of log quotients, pv: 0.42442; OK
SW: ok; ANOVA: Comp. ~ Subject_ID −> pv = 0.70763; EQUAL
Wilcoxon: Challenge; fasting: 0.5; sport: 0.125; OLTT: 0.5

PC 16:0_14:0 / PC aa C30:0
Shapiro−Wilk Test of log quotients, pv: 0.69141; OK
SW: ok; ANOVA: Comp. ~ Subject_ID −> pv = 0.47349; EQUAL
Wilcoxon: Challenge; fasting: 0.125; sport: 0.125; OLTT: 0.625

PC 18:0_12:0 / PC aa C30:0
Shapiro−Wilk Test of log quotients, pv: 3e−05; NO
SW: no; Kruskal: Comp. ~ Subject_ID −> pv = 0; DIFFERENCE
Wilcoxon: Challenge; fasting: 0.5; sport: 0.125; OLTT: 0.5

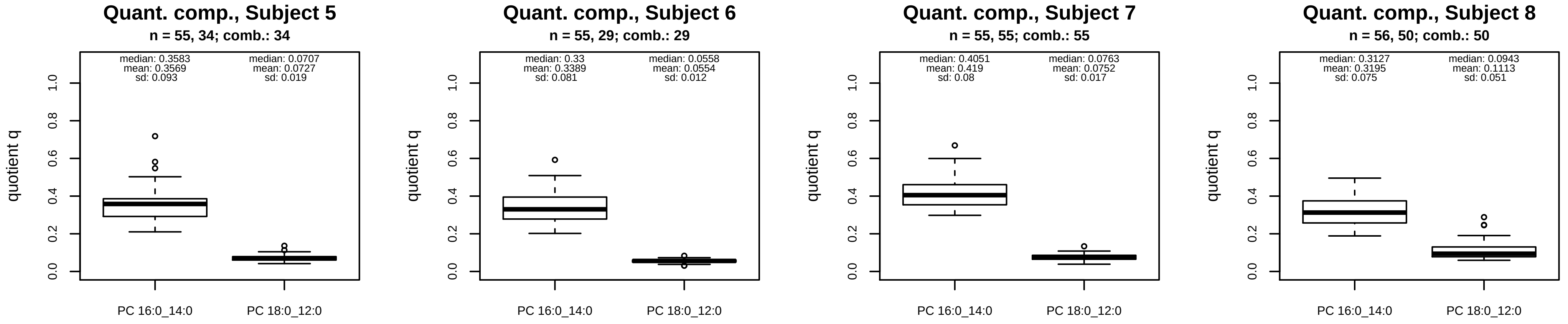

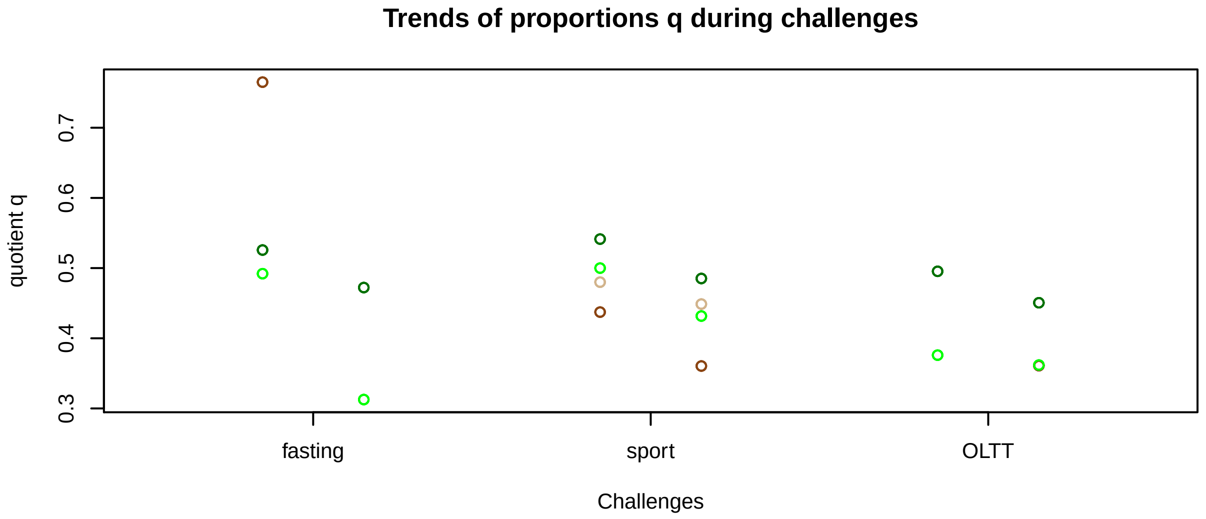

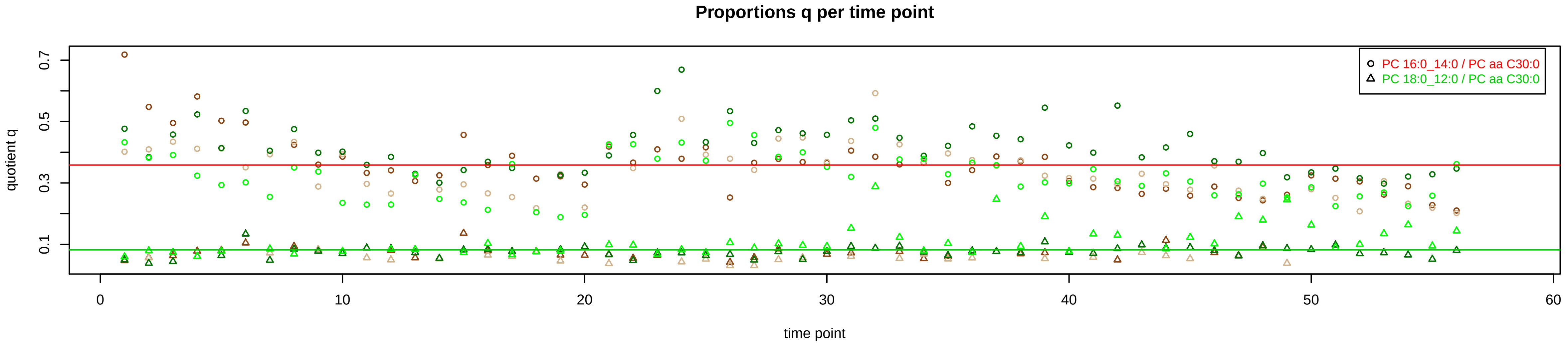

PC aa C32:0 = PC 16:0_16:0 + PC 18:0_14:0 + R

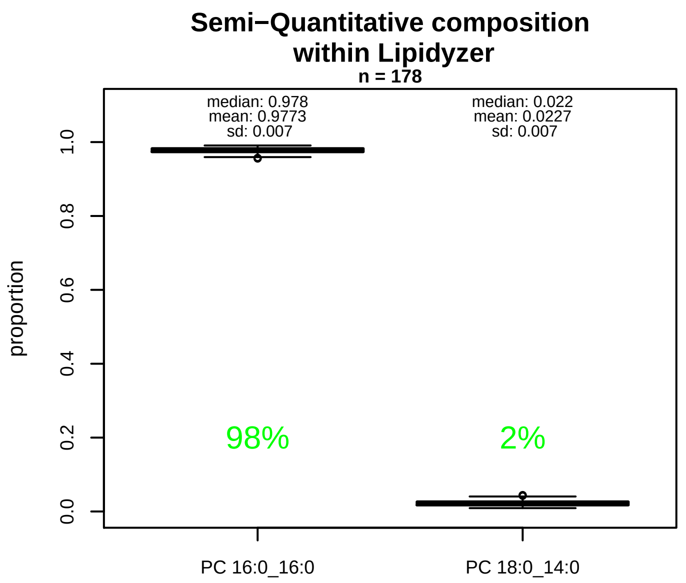

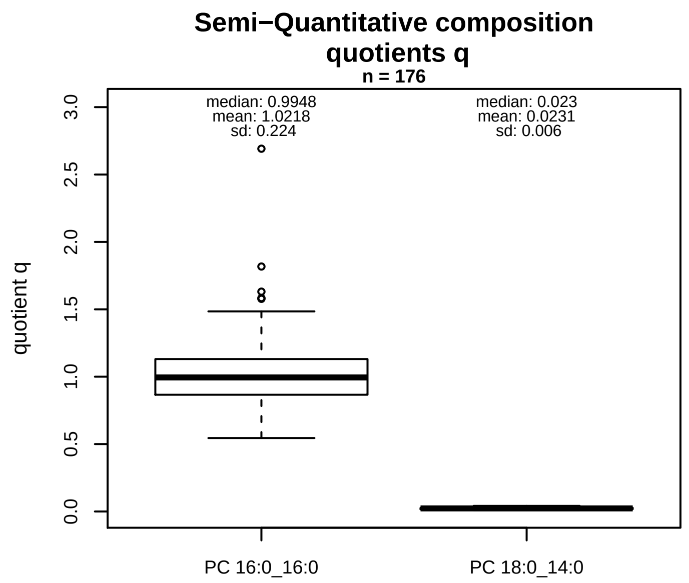

Semi−Quantitative composition: Lipidyzer

PC aa C32:0 consists of:
PC 16:0_16:0 97.7%
PC 18:0_14:0 2.3%
and of (not quantified):
PC 10:0_22:0, PC 11:0_21:0, PC 12:0_20:0, PC 13:0_19:0,
PC 15:0_17:0, PC 9:0_23:0, PC O−16:0_17:0, PC O−18:0_1[illegible]
[13C1]SM 36:0
and further compounds

Composition: mean of proportions q

conc(PC aa C32:0) * 1.0218 = conc(PC 16:0_16:0) [var(q)=1.0218]
Percentiles: 5%−>0.7561, 25%−>0.8661, 75%−>1.1308, 95%−>1.3897
conc(PC aa C32:0) * 0.0231 = conc(PC 18:0_14:0) [var(q)=0.0231]
Percentiles: 5%−>0.0138, 25%−>0.0173, 75%−>0.0274, 95%−>0.0347

Linear model

```
PC aa C32:0 ~ b * ( PC 16:0_16:0
   + PC 18:0_14:0 )
```

b = 0.49186

R^2 = 0.35292

Ranges

Measure	AbsoluteIDQ	sum(Lipidyzer)	delta
Min	8.95	7.19	1.76
Max	22.05	41.83	19.78
Mean	13.18	14	0.83
Median	12.22	13.74	1.52
SD	3.03	3.74	0.71

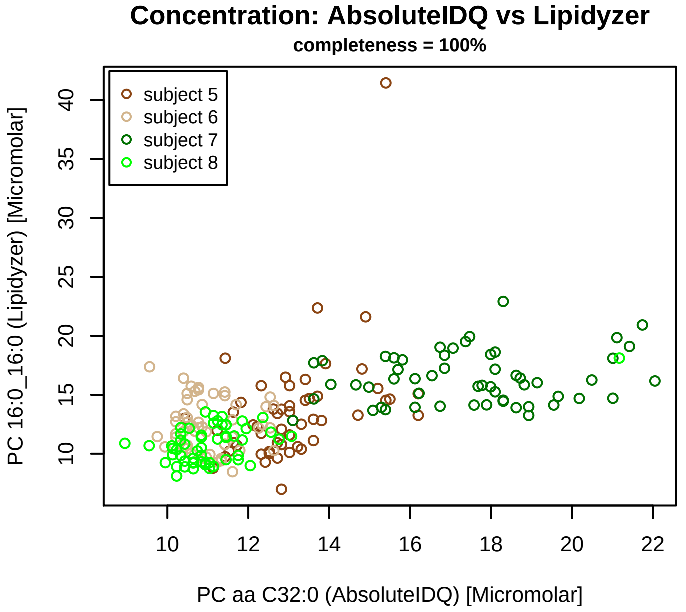

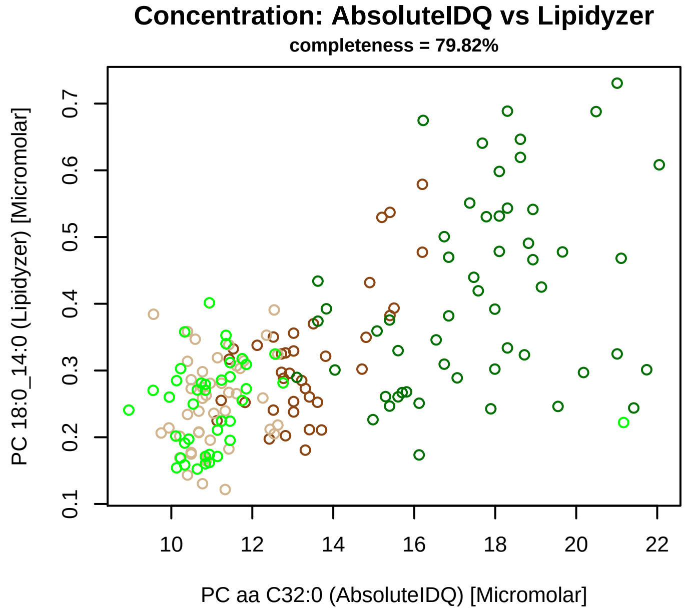

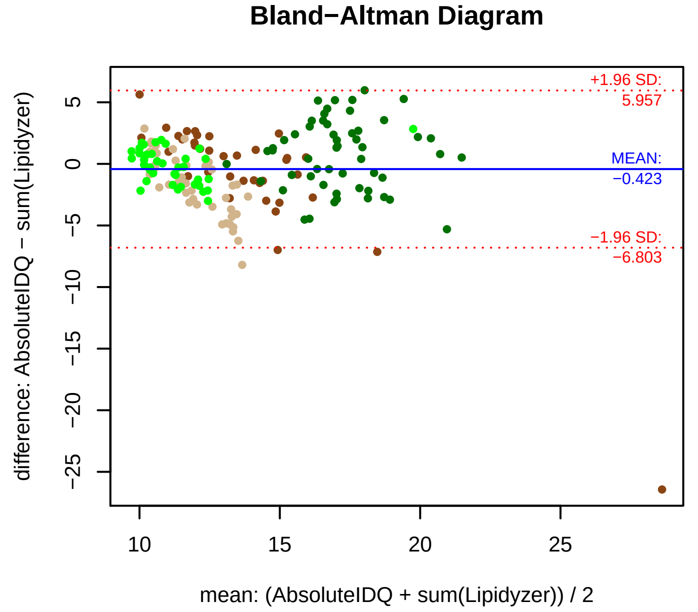

Stability of composition

(PC 16:0_16:0 + PC 18:0_14:0) / PC aa C32:0
Shapiro−Wilk Test of log quotients, pv: 0.00013; NO
SW: no; Kruskal: Comp. ~ Subject_ID −> pv = 0.00011; DIFFERENCE
Wilcoxon: Challenge; fasting: 0.75; sport: 0.125; OLTT: 1

PC 16:0_16:0 / PC aa C32:0
Shapiro−Wilk Test of log quotients, pv: 3e−05; NO
SW: no; Kruskal: Comp. ~ Subject_ID −> pv = 2e−05; DIFFERENCE
Wilcoxon: Challenge; fasting: 0.875; sport: 0.125; OLTT: 0.875

PC 18:0_14:0 / PC aa C32:0
Shapiro−Wilk Test of log quotients, pv: 0.02181; OK
SW: ok; ANOVA: Comp. ~ Subject_ID −> pv = 0.53573; EQUAL
Wilcoxon: Challenge; fasting: 0.25; sport: 0.625; OLTT: 0.5

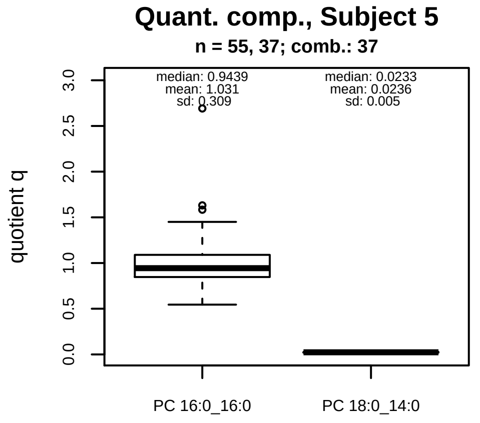

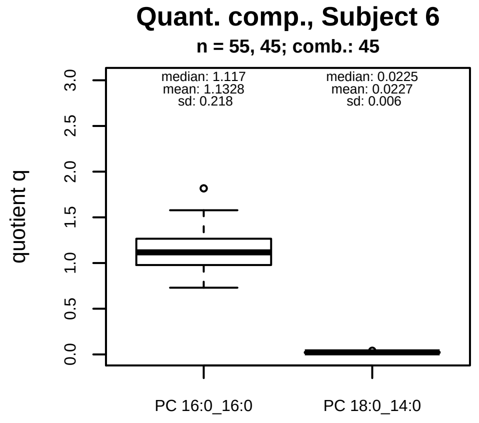

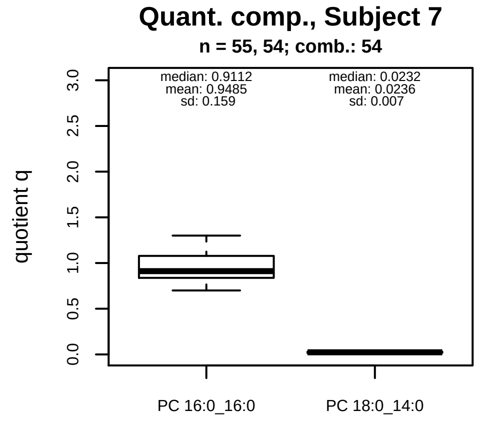

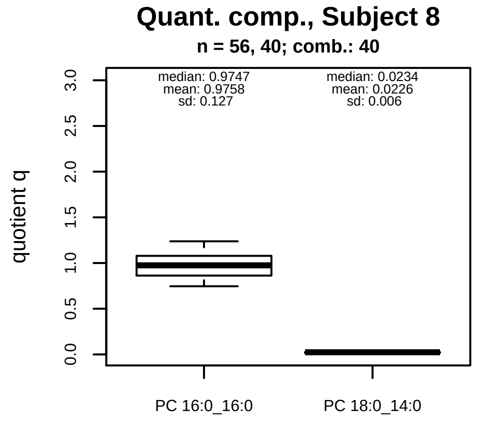

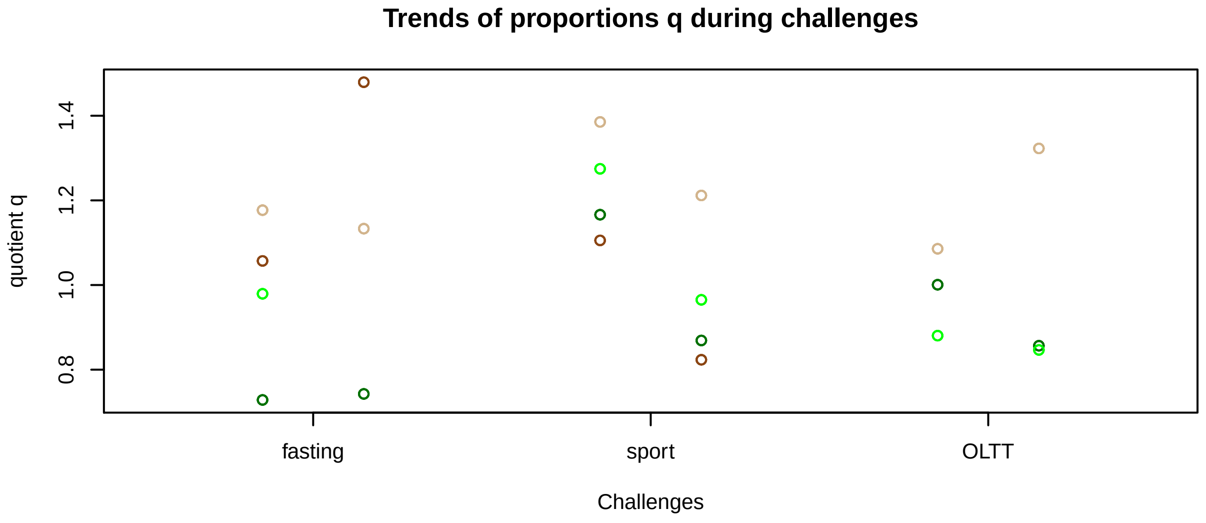

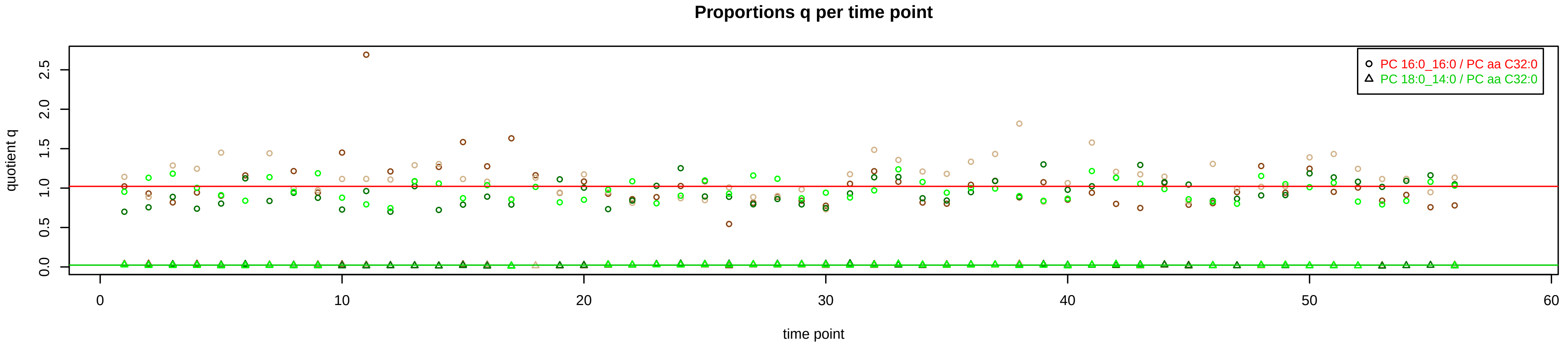

PC aa C32:1 = PC 14:0_18:1 + PC 16:0_16:1 + R

Semi−Quantitative composition within Lipidyzer

n = 222

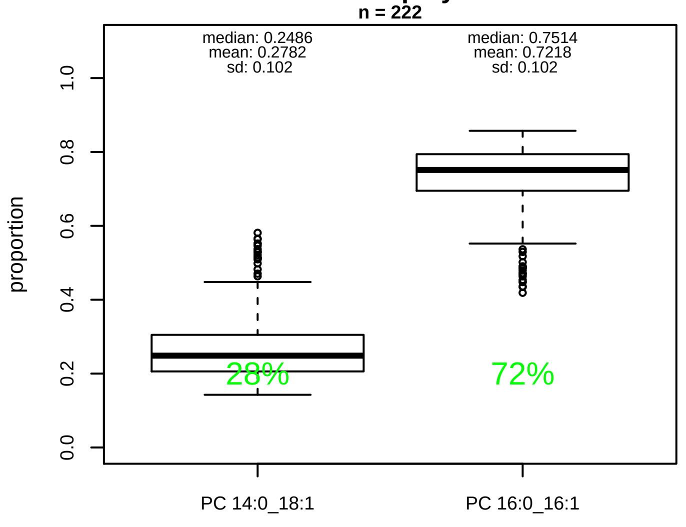

Semi−Quantitative composition quotients q

n = 220

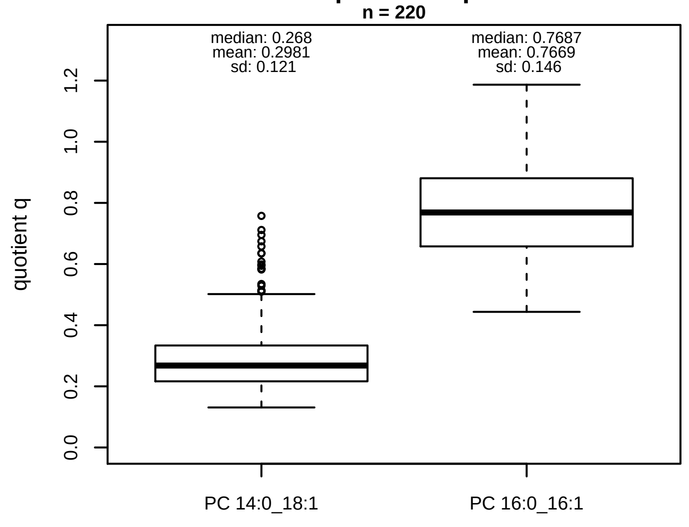

Semi−Quantitative composition: Lipidyzer

PC aa C32:1 consists of:
PC 14:0_18:1 27.8%
PC 16:0_16:1 72.2%
and of (not quantified):
PC 12:0_20:1, PC 13:0_19:1, PC 14:1_18:0, PC 15:0_17:1,
PC 15:1_17:0, PC O−16:0_17:1, PC O−18:0_15:1, PC O−16:
PC O−20:1_13:0
and further compounds

Composition: mean of proportions q

conc(PC aa C32:1) * 0.2981 = conc(PC 14:0_18:1) [var(q)=0.2981]
Percentiles: 5%−>0.1773, 25%−>0.2167, 75%−>0.3336, 95%−>0.5843
conc(PC aa C32:1) * 0.7669 = conc(PC 16:0_16:1) [var(q)=0.7669]
Percentiles: 5%−>0.5433, 25%−>0.6577, 75%−>0.8806, 95%−>0.9891

Linear model

```
PC aa C32:1 ~ b * ( PC 14:0_18:1
   + PC 16:0_16:1 )
```

b = 0.97254

R^2 = 0.95086

Ranges

Measure	AbsoluteIDQ	sum(Lipidyzer)	delta
Min	4.79	4.01	0.78
Max	38.24	41.4	3.16
Mean	13.42	14.16	0.74
Median	11.09	12.15	1.06
SD	8.15	8.14	0.01

Concentration: AbsoluteIDQ vs Lipidyzer

completeness = 99.55%

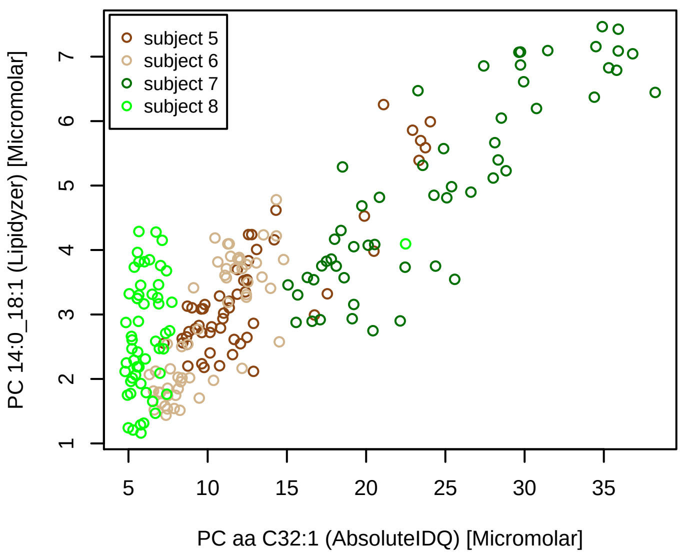

Concentration: AbsoluteIDQ vs Lipidyzer

completeness = 100%

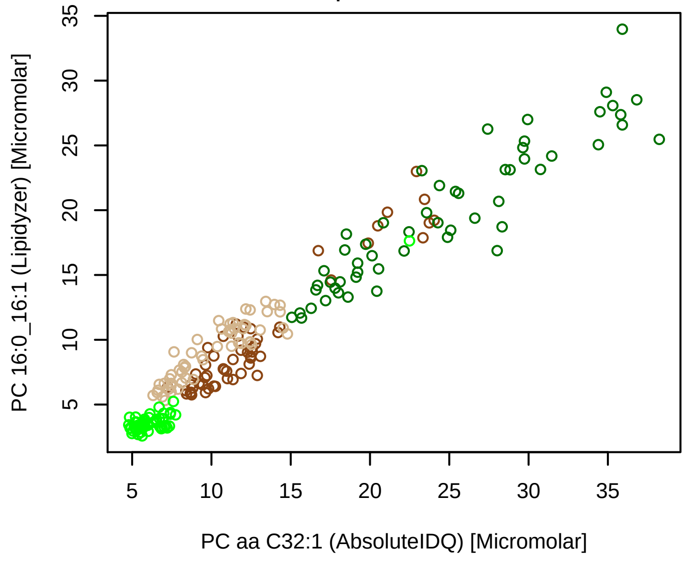

Bland−Altman Diagram

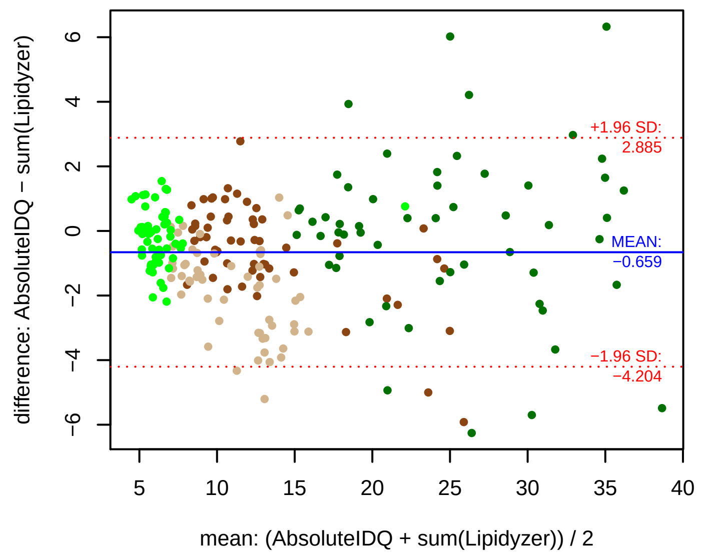

Stability of composition

(PC 14:0_18:1 + PC 16:0_16:1) / PC aa C32:1
Shapiro−Wilk Test of log quotients, pv: 0.23341; OK
SW: ok; ANOVA: Comp. ~ Subject_ID −> pv = 0.02667; EQUAL
Wilcoxon: Challenge; fasting: 0.25; sport: 0.625; OLTT: 0.875

PC 14:0_18:1 / PC aa C32:1
Shapiro−Wilk Test of log quotients, pv: 1e−05; NO
SW: ok; ANOVA: Comp. ~ Subject_ID −> pv = 0; DIFFERENCE
Wilcoxon: Challenge; fasting: 0.125; sport: 0.625; OLTT: 0.25

PC 16:0_16:1 / PC aa C32:1
Shapiro−Wilk Test of log quotients, pv: 0.00051; NO
SW: ok; ANOVA: Comp. ~ Subject_ID −> pv = 0; DIFFERENCE
Wilcoxon: Challenge; fasting: 1; sport: 0.375; OLTT: 1

Quant. comp., Subject 5

n = 55, 55; comb.: 55

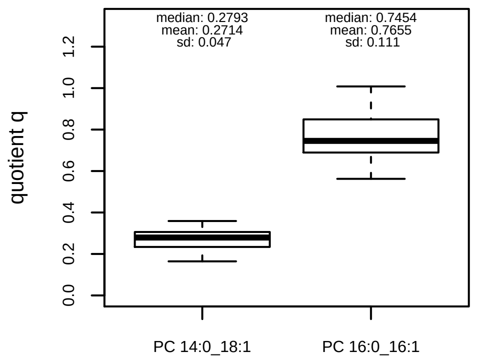

Quant. comp., Subject 6

n = 55, 55; comb.: 55

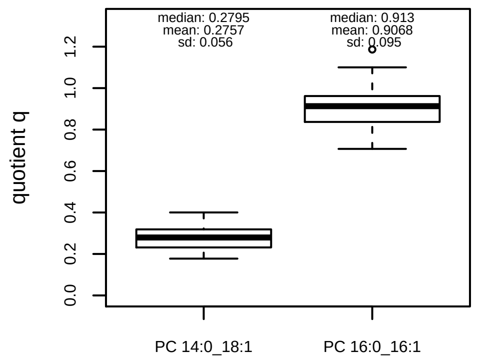

Quant. comp., Subject 7

n = 55, 55; comb.: 55

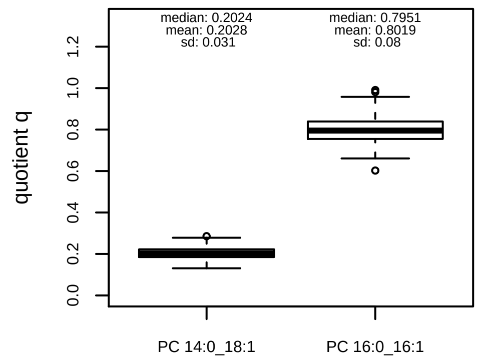

Quant. comp., Subject 8

n = 55, 56; comb.: 55

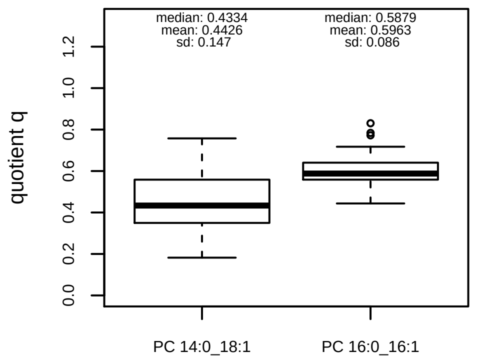

Trends of proportions q during challenges

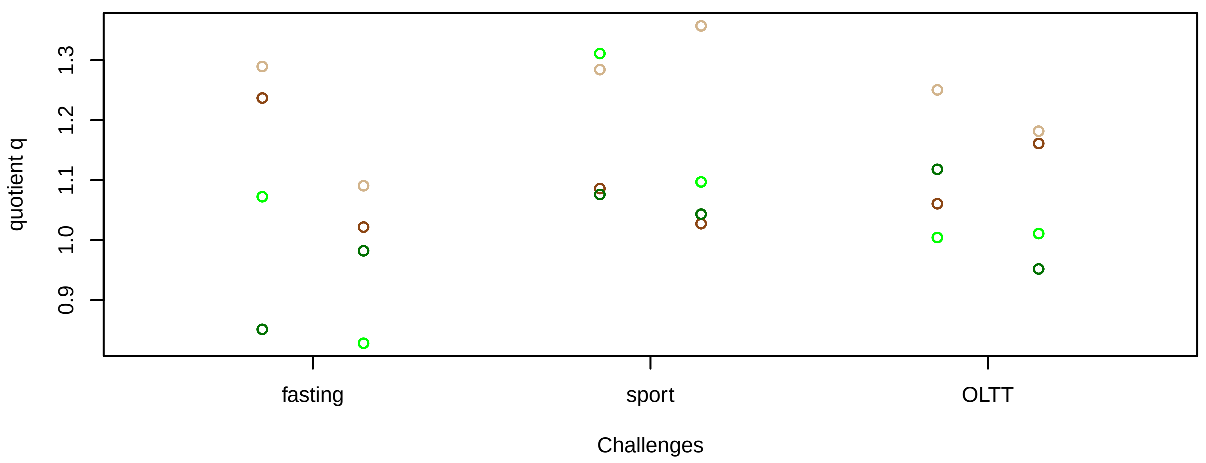

Proportions q per time point

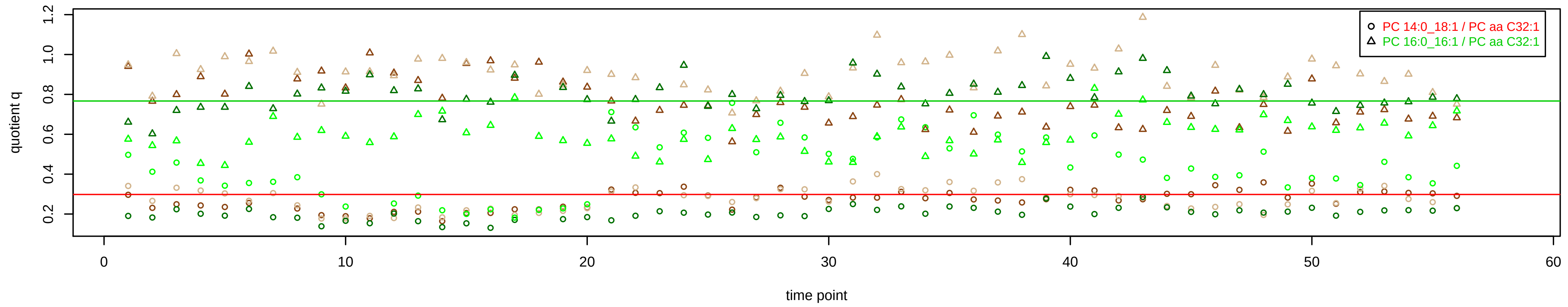

PC aa C32:2 = PC 14:0_18:2 + R

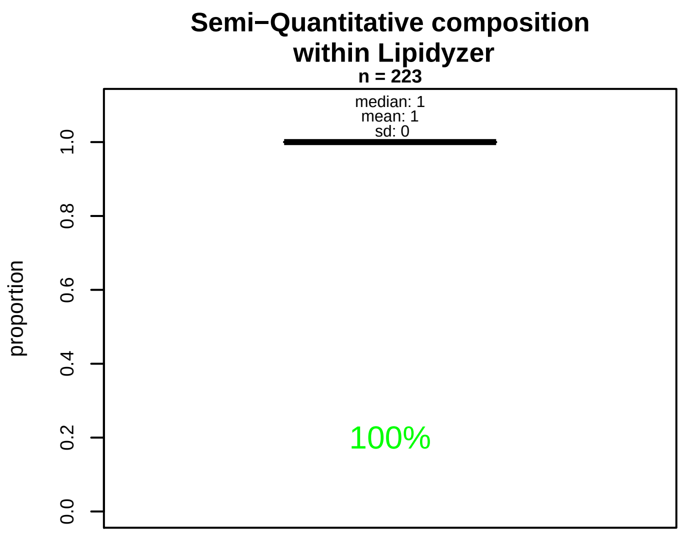

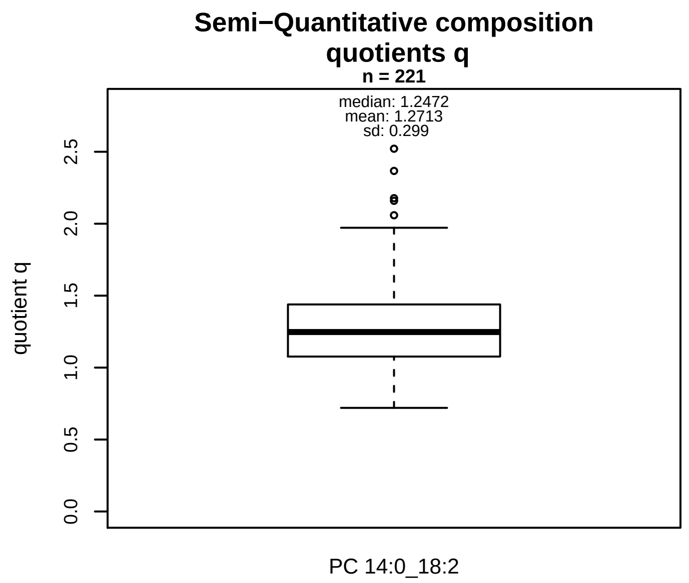

Semi−Quantitative composition: Lipidyzer

PC aa C32:2 consists of:
PC 14:0_18:2 100%
and of (not quantified):
PC 12:0_20:2, PC 14:1_18:1, PC 15:0_17:2, PC 16:1_16:1,
PC 15:1_17:1, PC O−16:0_17:2, PC O−16:1_17:1, PC O−18:
and further compounds

Composition: mean of proportions q

conc(PC aa C32:2) * 1.2713 = conc(PC 14:0_18:2) [var(q)=1.2713]
Percentiles: 5%−>0.8229, 25%−>1.0768, 75%−>1.439, 95%−>1.7371

Linear model

```
PC aa C32:2 ~ b * ( PC 14:0_18:2 )
```

b = 1.03115

R^2 = 0.78893

Ranges

Measure	AbsoluteIDQ	sum(Lipidyzer)	delta
Min	0.66	1.23	0.56
Max	5.99	6.04	0.05
Mean	2.55	2.99	0.43
Median	2.32	2.77	0.45
SD	1.27	1.09	0.18

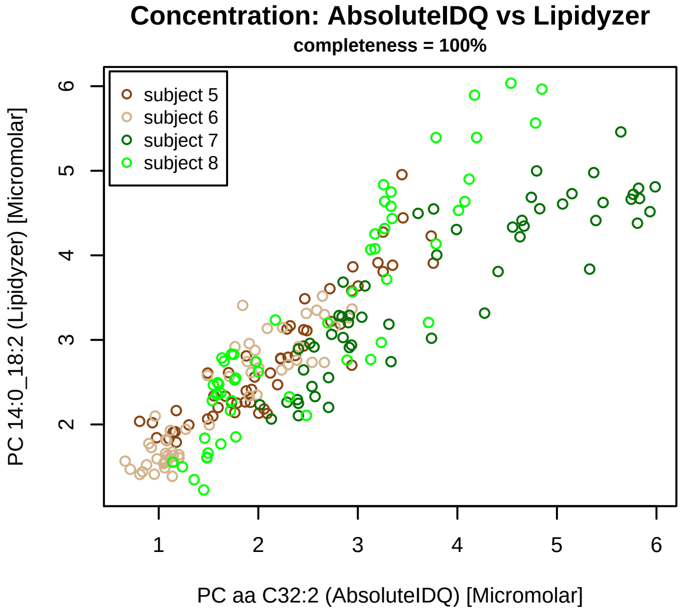

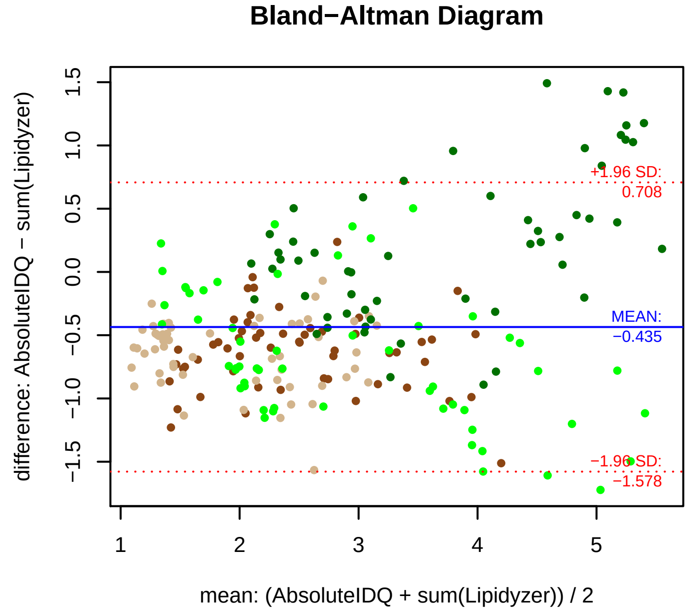

Stability of composition

PC 14:0_18:2 / PC aa C32:2
Shapiro−Wilk Test of log quotients, pv: 0.28199; OK
SW: no; Kruskal: Comp. ~ Subject_ID −> pv = 0; DIFFERENCE
Wilcoxon: Challenge; fasting: 0.875; sport: 0.875; OLTT: 0.25

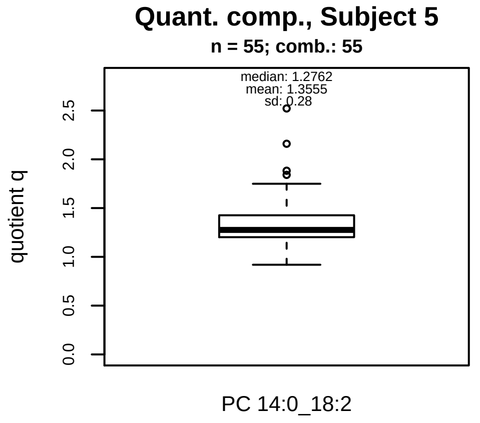

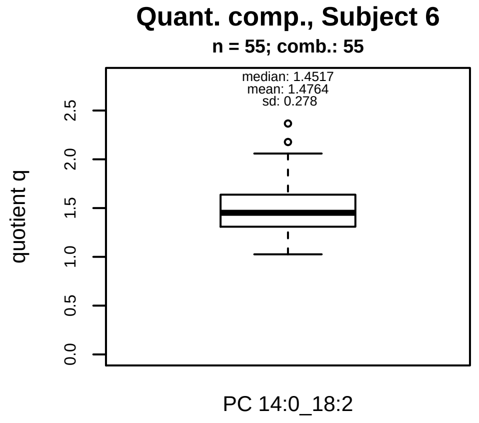

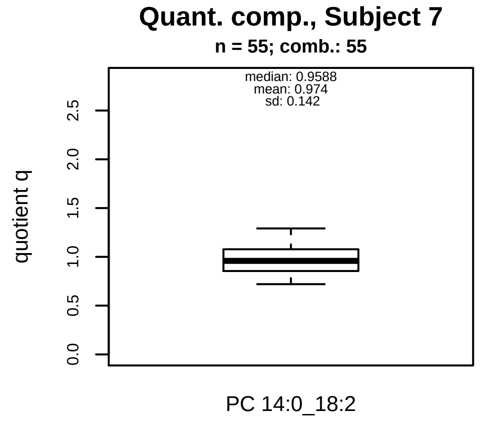

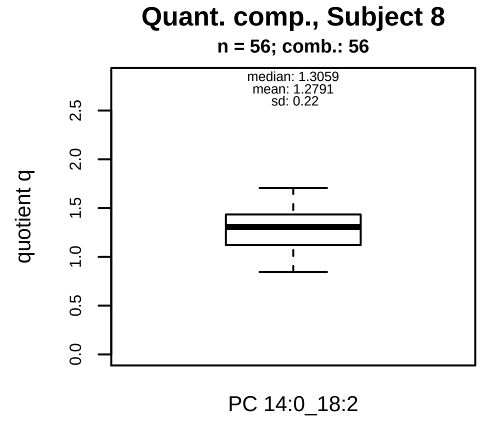

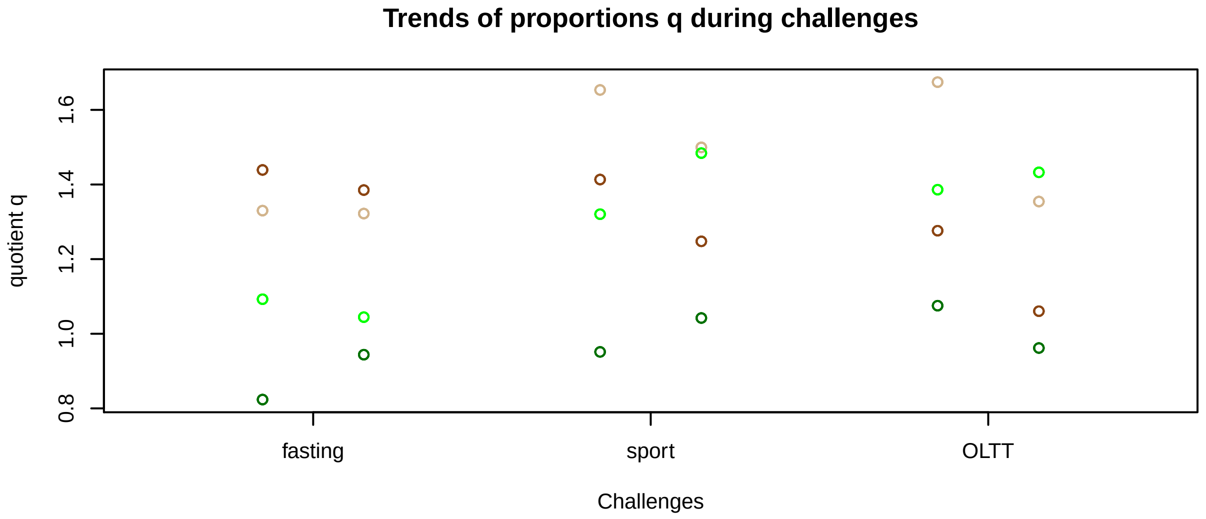

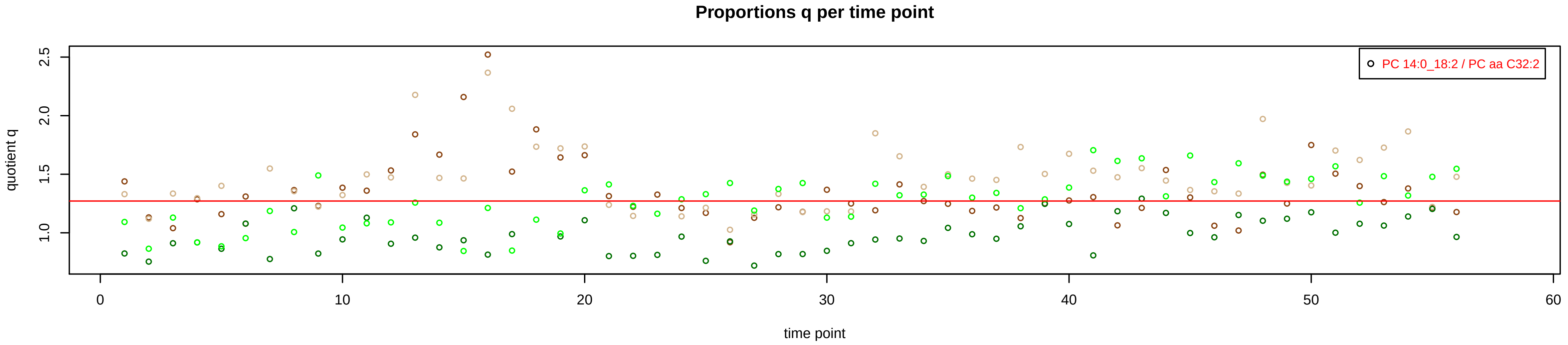

PC aa C32:3 = PC 14:0_18:3 + R

PC 14:0_18:3 excluded because of missingness > 75%

No independent Variable with coverage >0.25 out of PC 14:0_18:3

Qualitative composition

PC aa C32:3 consists of:
PC 14:0_18:3, PC 12:0_20:3, PC 14:1_18:2, PC 15:1_17:2, [13C1]SM 36:3
and further compounds

PC aa C34:1 = PC 14:0_20:1 + PC 16:0_18:1 + PC 18:0_16:1 + PC 20:0_14:1 + R

PC 14:0_20:1 excluded because of missingness > 75%

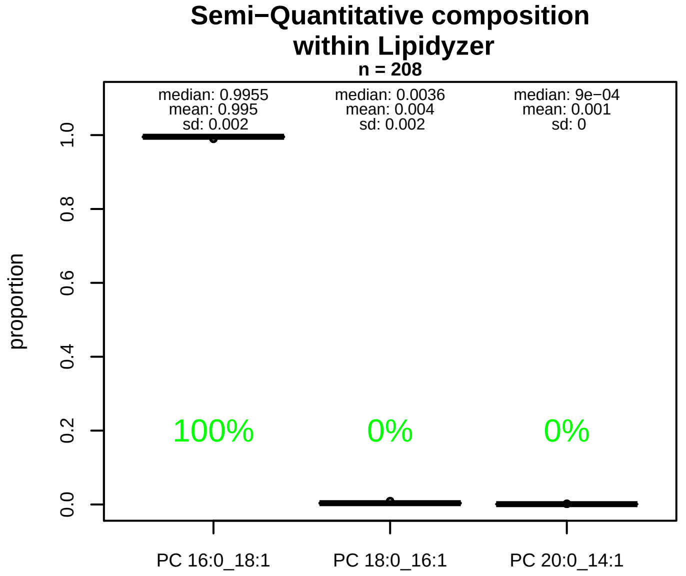

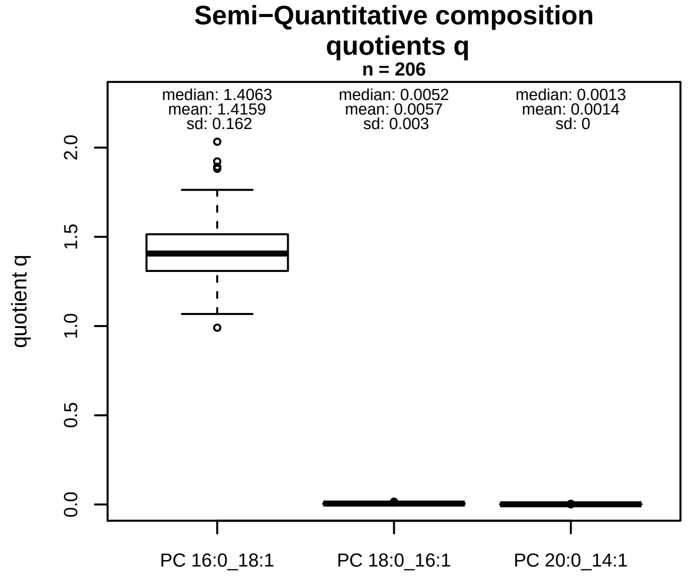

Semi−Quantitative composition: Lipidyzer

PC aa C34:1 consists of:
PC 16:0_18:1 99.5%
PC 18:0_16:1 0.4%
PC 20:0_14:1 0.1%
and of (not quantified):
PC 14:0_20:1, PC 12:0_22:1, PC 14:1_20:0, PC 15:0_19:1,
PC 15:1_19:0, PC 17:0_17:1, PC O−17:0_18:1, PC O−16:0_
PC O−18:0_17:1, PC O−20:0_15:1, PC O−16:1_19:0, PC O−
[13C1]SM 38:1
and further compounds

Composition: mean of proportions q

conc(PC aa C34:1) * 1.4159 = conc(PC 16:0_18:1) [var(q)=1.4159]
Percentiles: 5%−>1.1793, 25%−>1.3089, 75%−>1.5146, 95%−>1.6821
conc(PC aa C34:1) * 0.0057 = conc(PC 18:0_16:1) [var(q)=0.0057]
Percentiles: 5%−>0.0028, 25%−>0.0036, 75%−>0.0067, 95%−>0.012
conc(PC aa C34:1) * 0.0014 = conc(PC 20:0_14:1) [var(q)=0.0014]
Percentiles: 5%−>9e−04, 25%−>0.0011, 75%−>0.0016, 95%−>0.0022

Linear model

```
PC aa C34:1 ~ b * ( PC 16:0_18:1
   + PC 18:0_16:1
   + PC 20:0_14:1 )
```

b = 0.55441

R^2 = 0.86597

Ranges

Measure	AbsoluteIDQ	sum(Lipidyzer)	delta
Min	89.8	127.65	37.85
Max	325.5	471.26	145.76
Mean	187.14	268.99	81.85
Median	183.6	261.66	78.06
SD	49.04	84.03	35

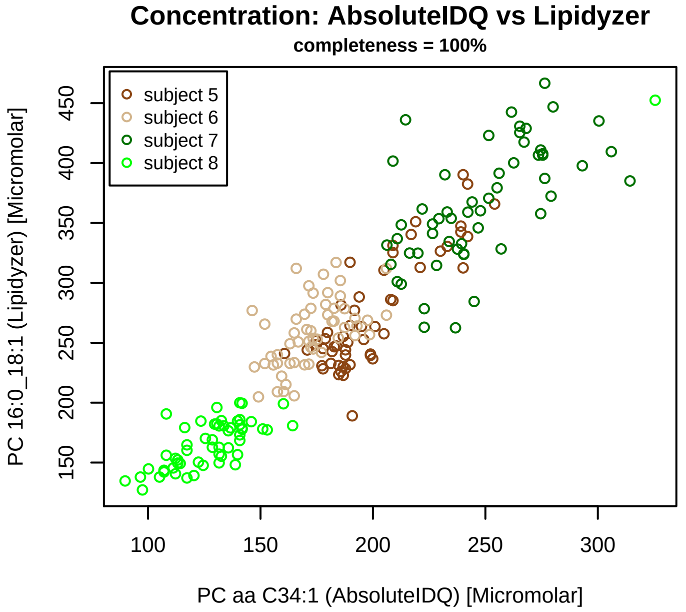

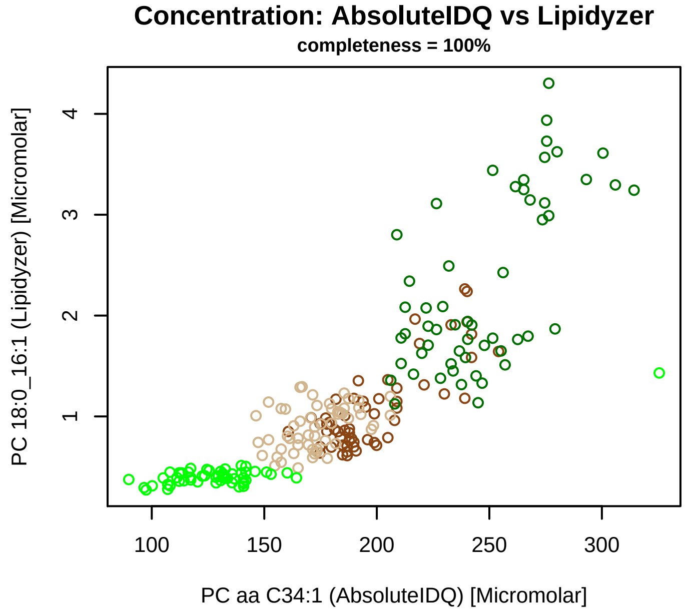

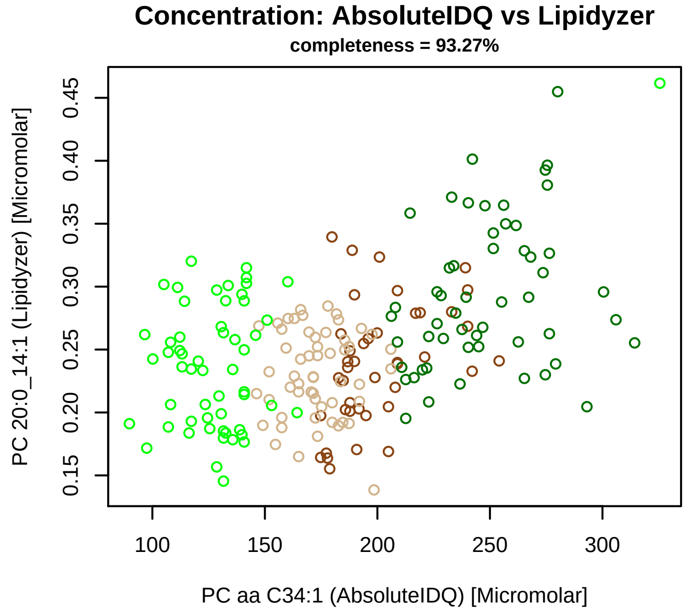

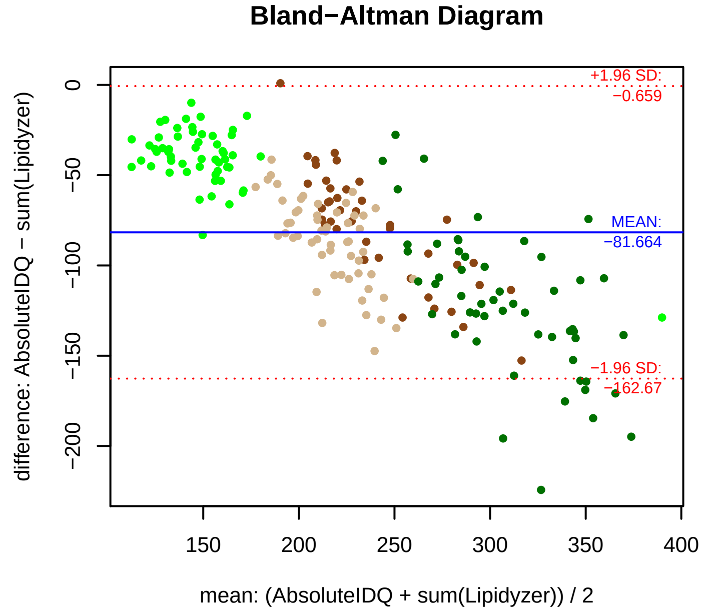

Stability of composition

(PC 16:0_18:1 + PC 18:0_16:1 + PC 20:0_14:1) / PC aa C34:1
Shapiro−Wilk Test of log quotients, pv: 0.82819; OK
SW: ok; ANOVA: Comp. ~ Subject_ID −> pv = 0.00213; DIFFERENCE
Wilcoxon: Challenge; fasting: 0.75; sport: 0.125; OLTT: 1

PC 16:0_18:1 / PC aa C34:1
Shapiro−Wilk Test of log quotients, pv: 0.85563; OK
SW: ok; ANOVA: Comp. ~ Subject_ID −> pv = 0.02562; EQUAL
Wilcoxon: Challenge; fasting: 0.625; sport: 0.125; OLTT: 1

PC 18:0_16:1 / PC aa C34:1
Shapiro−Wilk Test of log quotients, pv: 0.00069; NO
SW: ok; ANOVA: Comp. ~ Subject_ID −> pv = 0.00029; DIFFERENCE
Wilcoxon: Challenge; fasting: 0.125; sport: 0.25; OLTT: 0.125

PC 20:0_14:1 / PC aa C34:1
Shapiro−Wilk Test of log quotients, pv: 0.01446; OK
SW: ok; ANOVA: Comp. ~ Subject_ID −> pv = 0; DIFFERENCE
Wilcoxon: Challenge; fasting: 0.75; sport: 0.625; OLTT: 1

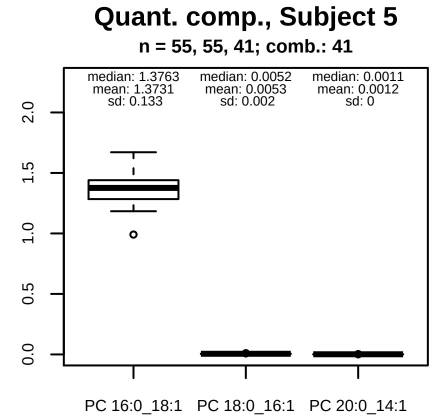

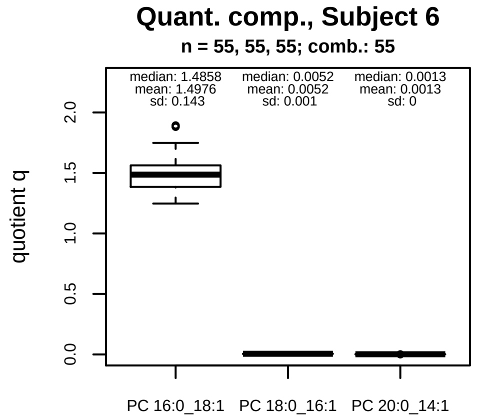

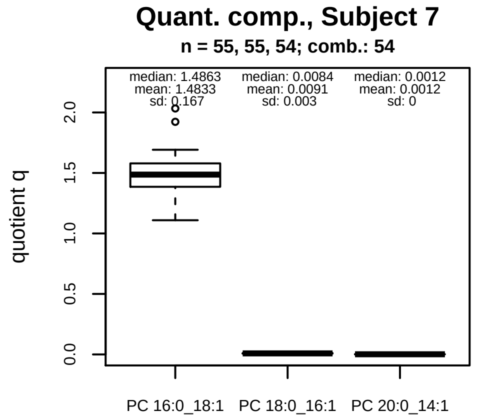

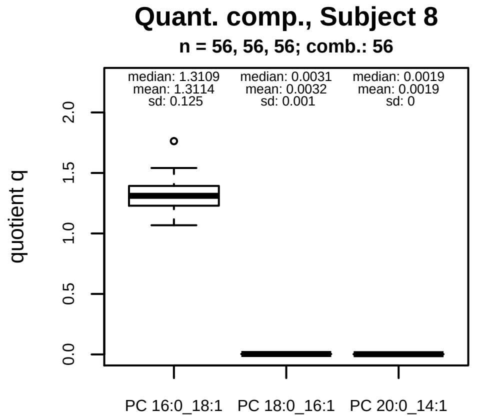

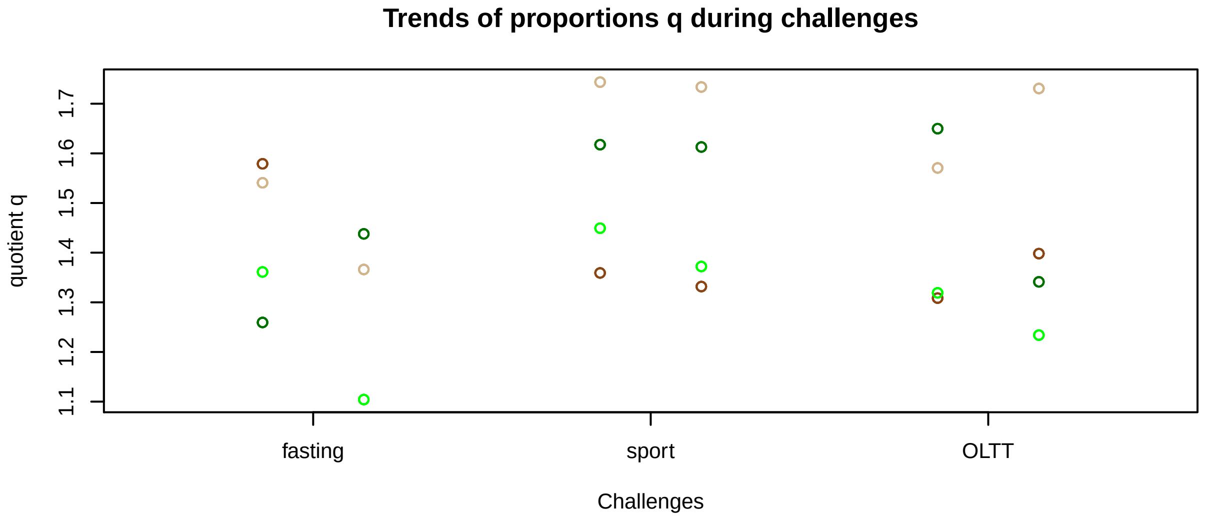

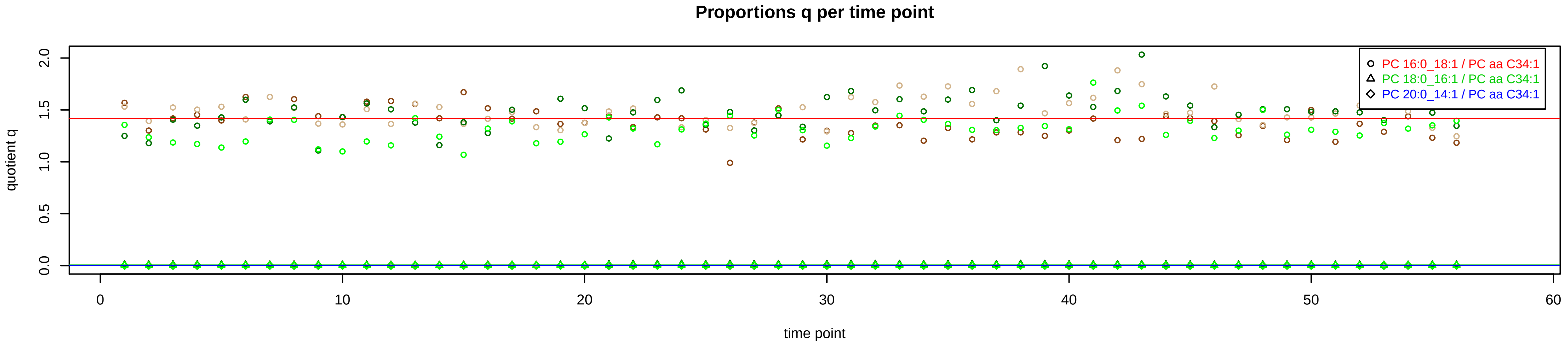

PC aa C34:2 = PC 14:0_20:2 + PC 16:0_18:2 + PC 18:1_16:1 + R

Semi−Quantitative composition within Lipidyzer
n = 70

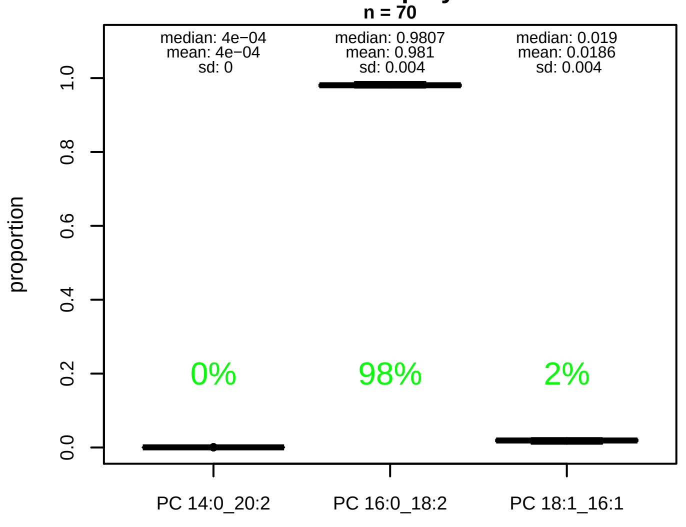

Semi−Quantitative composition quotients q
n = 70

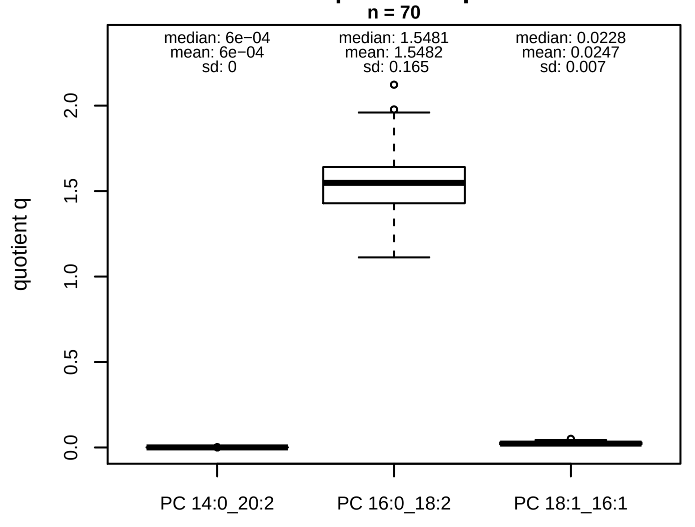

Semi−Quantitative composition: Lipidyzer

PC aa C34:2 consists of:
PC 14:0_20:2 <0.05%
PC 16:0_18:2 98.1%
PC 18:1_16:1 1.9%
and of (not quantified):
PC 12:0_22:2, PC 14:1_20:1, PC 15:1_19:1, PC 17:0_17:2,
PC 17:1_17:1, PC 18:0_16:2, PC O−18:0_17:2, PC O−16:1_[illegible]
[13C1]SM 38:2
and further compounds

Linear model

```
PC aa C34:2 ~ b * ( PC 14:0_20:2
  + PC 16:0_18:2
  + PC 18:1_16:1 )
```

b = 0.39293

R^2 = 0.49433

Ranges

Measure	AbsoluteIDQ	sum(Lipidyzer)	delta
Min	233.6	349.63	116.03
Max	465	708.04	243.04
Mean	320.57	543.8	223.23
Median	315.6	551.65	236.05
SD	40.33	79.51	39.18

Composition: mean of proportions q

conc(PC aa C34:2) * 6e−04 = conc(PC 14:0_20:2) [var(q)=6e−04]
Percentiles: 5%−>4e−04, 25%−>5e−04, 75%−>7e−04, 95%−>0.001
conc(PC aa C34:2) * 1.5482 = conc(PC 16:0_18:2) [var(q)=1.5482]
Percentiles: 5%−>1.2968, 25%−>1.4291, 75%−>1.6414, 95%−>1.8214
conc(PC aa C34:2) * 0.0247 = conc(PC 18:1_16:1) [var(q)=0.0247]
Percentiles: 5%−>0.0155, 25%−>0.0188, 75%−>0.0299, 95%−>0.0371

Concentration: AbsoluteIDQ vs Lipidyzer
completeness = 31.39%

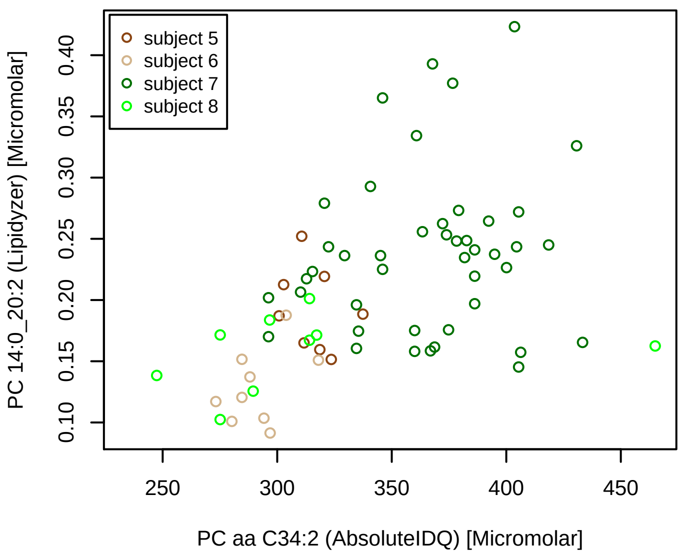

Concentration: AbsoluteIDQ vs Lipidyzer
completeness = 100%

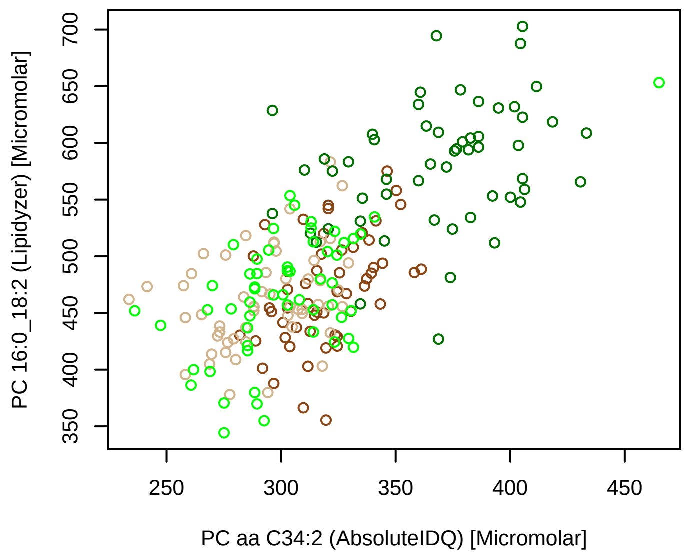

Concentration: AbsoluteIDQ vs Lipidyzer
completeness = 100%

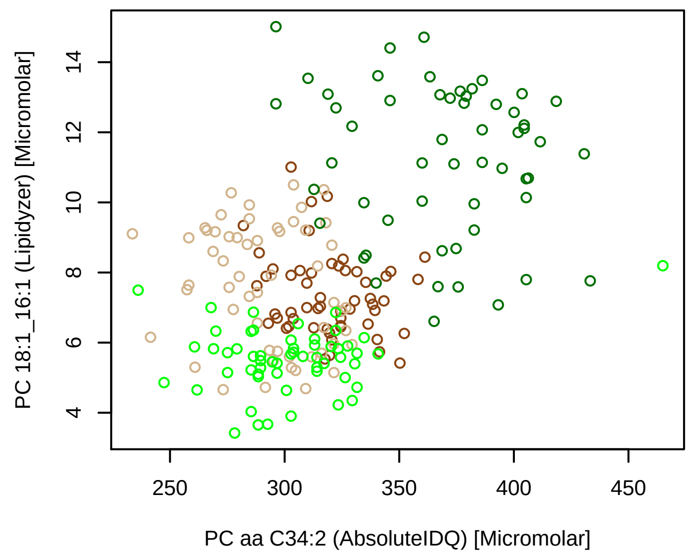

Bland−Altman Diagram

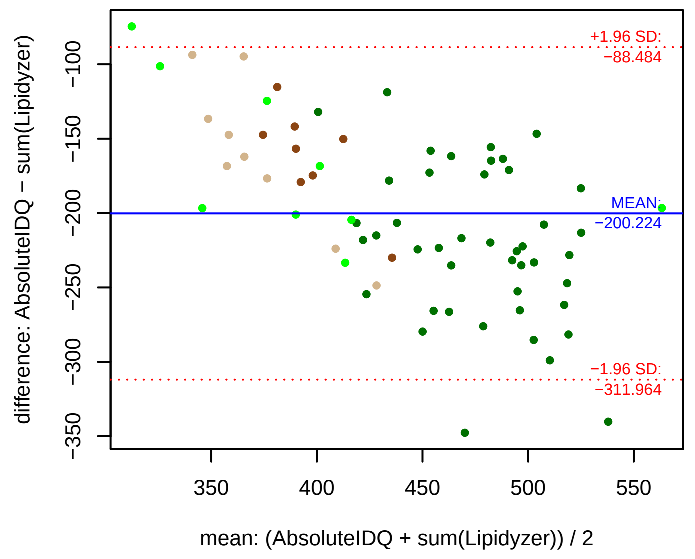

Stability of composition

(PC 14:0_20:2 + PC 16:0_18:2 + PC 18:1_16:1) / PC aa C34:2
Shapiro−Wilk Test of log quotients, pv: 0.55528; OK
SW: ok; ANOVA: Comp. ~ Subject_ID −> pv = 0.38377; EQUAL
Wilcoxon: Challenge; fasting: 1; sport: 1; OLTT: 1

PC 14:0_20:2 / PC aa C34:2
Shapiro−Wilk Test of log quotients, pv: 0.38507; OK
SW: ok; ANOVA: Comp. ~ Subject_ID −> pv = 0.61799; EQUAL
Wilcoxon: Challenge; fasting: 1; sport: 1; OLTT: 1

PC 16:0_18:2 / PC aa C34:2
Shapiro−Wilk Test of log quotients, pv: 0.95815; OK
SW: ok; ANOVA: Comp. ~ Subject_ID −> pv = 0.021; EQUAL
Wilcoxon: Challenge; fasting: 0.375; sport: 0.875; OLTT: 0.625

PC 18:1_16:1 / PC aa C34:2
Shapiro−Wilk Test of log quotients, pv: 0.00691; NO
SW: no; Kruskal: Comp. ~ Subject_ID −> pv = 0; DIFFERENCE
Wilcoxon: Challenge; fasting: 0.25; sport: 0.375; OLTT: 0.125

Quant. comp., Subject 5
n = 8, 55, 55; comb.: 8

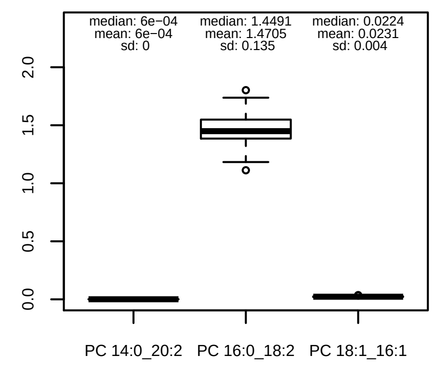

Quant. comp., Subject 6
n = 9, 55, 55; comb.: 9

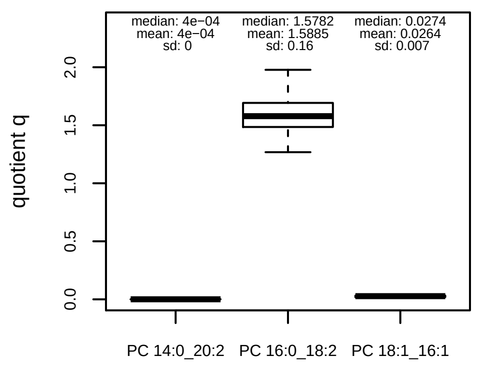

Quant. comp., Subject 7
n = 44, 55, 55; comb.: 44

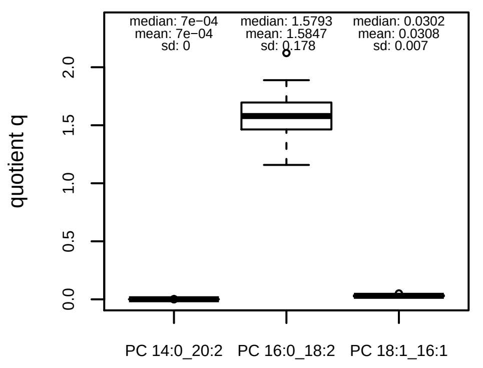

Quant. comp., Subject 8
n = 9, 56, 56; comb.: 9

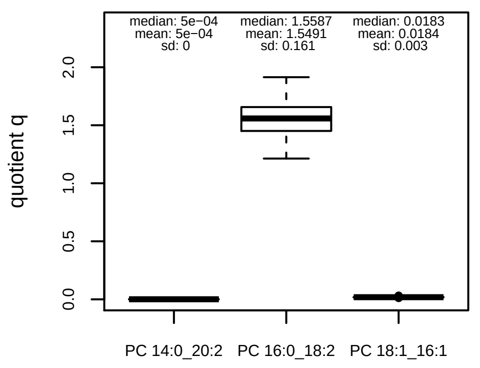

Trends of proportions q during challenges

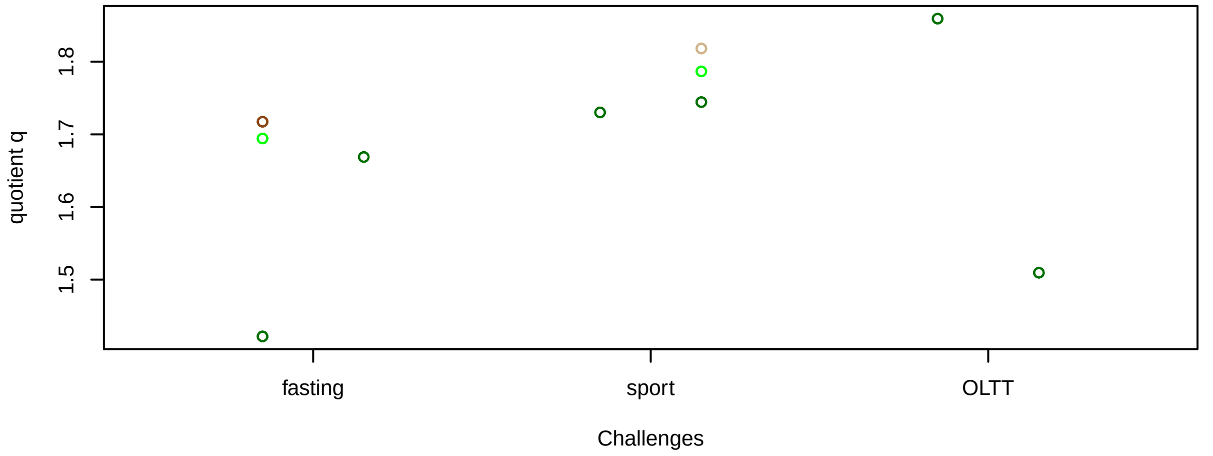

Proportions q per time point

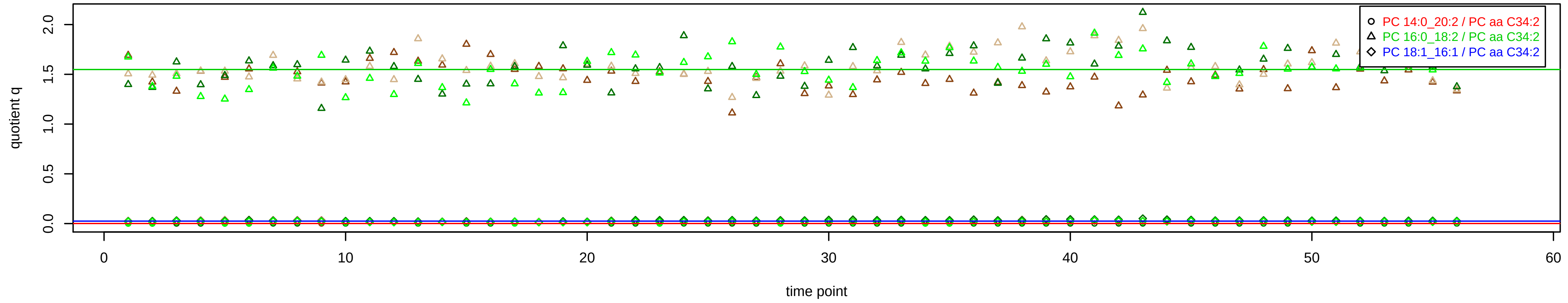

PC aa C34:3 = PC 14:0_20:3 + PC 16:0_18:3 + PC 18:2_16:1 + R

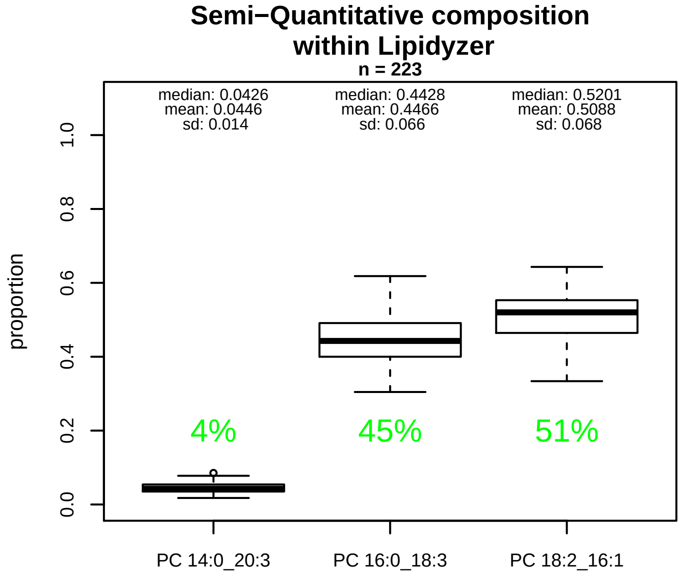

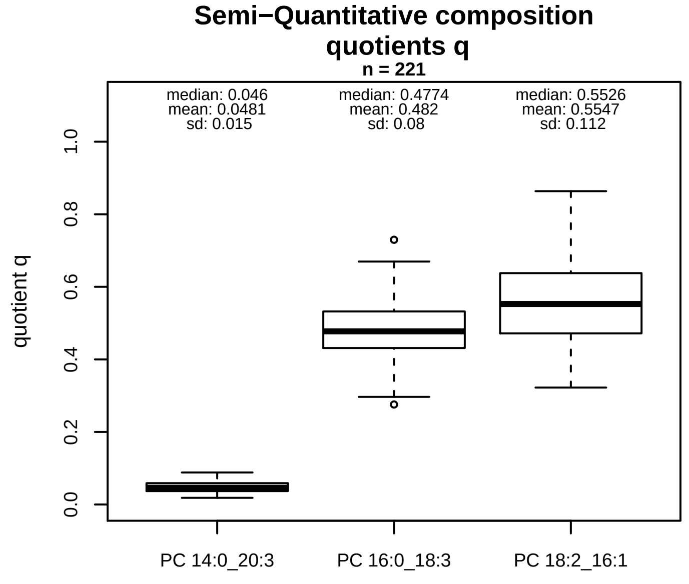

Semi−Quantitative composition: Lipidyzer

PC aa C34:3 consists of:
PC 14:0_20:3 4.5%
PC 16:0_18:3 44.7%
PC 18:2_16:1 50.9%
and of (not quantified):
PC 14:1_20:2, PC 16:1_18:2, PC 17:1_17:2, PC O−18:1_17:2
and further compounds

Linear model

```
PC aa C34:3 ~ b * ( PC 14:0_20:3
  + PC 16:0_18:3
  + PC 18:2_16:1 )
```

b = 1.0004

R^2 = 0.85431

Ranges

Measure	AbsoluteIDQ	sum(Lipidyzer)	delta
Min	8.23	8.34	0.11
Max	29	29.94	0.94
Mean	15.67	16.72	1.05
Median	13.98	15.66	1.68
SD	4.82	4.43	0.39

Composition: mean of proportions q

conc(PC aa C34:3) * 0.0481 = conc(PC 14:0_20:3) [var(q)=0.0481]
Percentiles: 5%−>0.0252, 25%−>0.0369, 75%−>0.0588, 95%−>0.0729
conc(PC aa C34:3) * 0.482 = conc(PC 16:0_18:3) [var(q)=0.482]
Percentiles: 5%−>0.3539, 25%−>0.4312, 75%−>0.5322, 95%−>0.6165
conc(PC aa C34:3) * 0.5547 = conc(PC 18:2_16:1) [var(q)=0.5547]
Percentiles: 5%−>0.3811, 25%−>0.4718, 75%−>0.6377, 95%−>0.7406

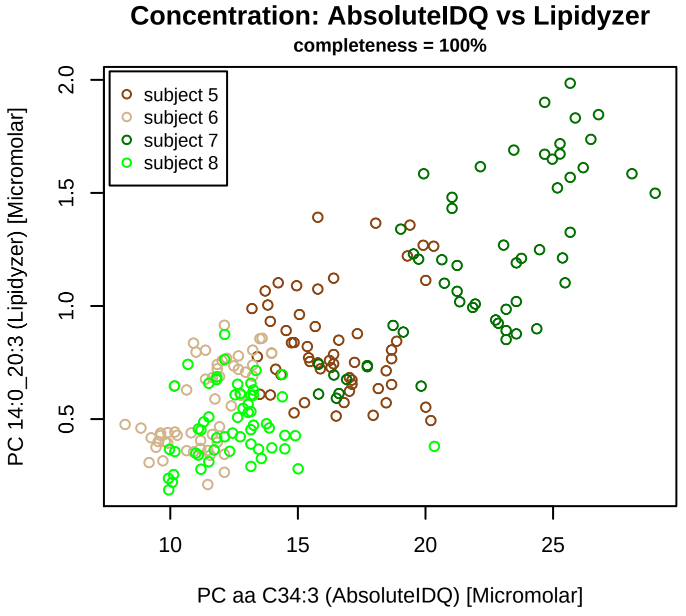

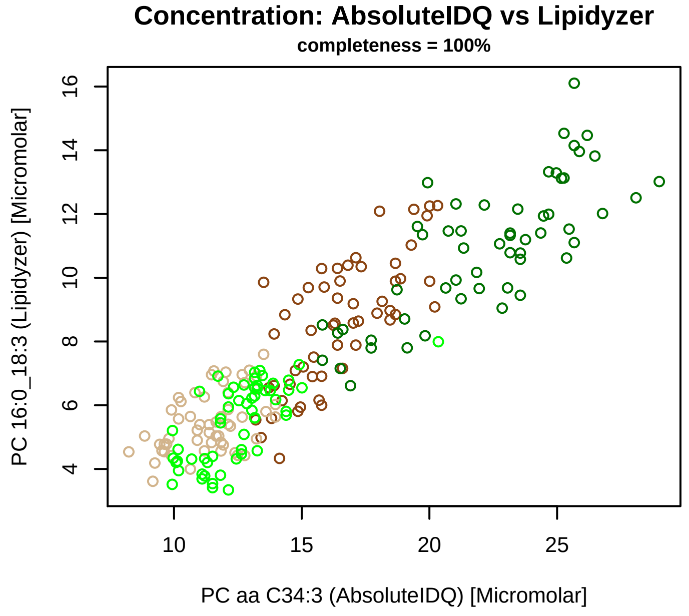

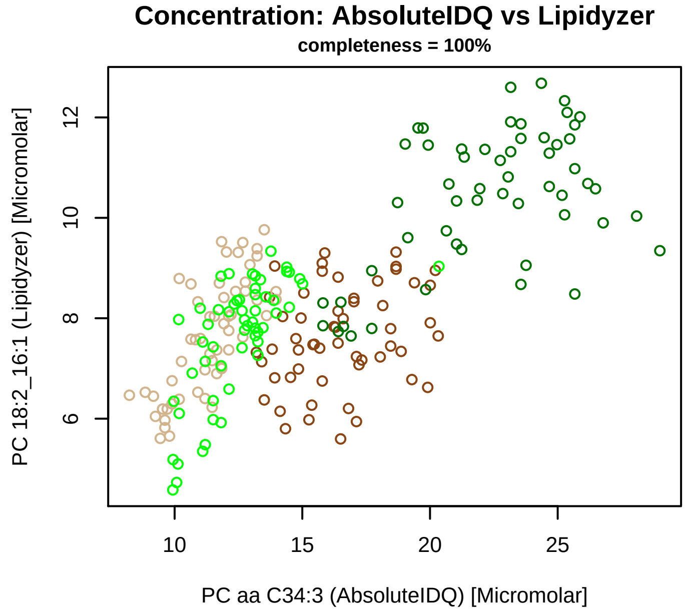

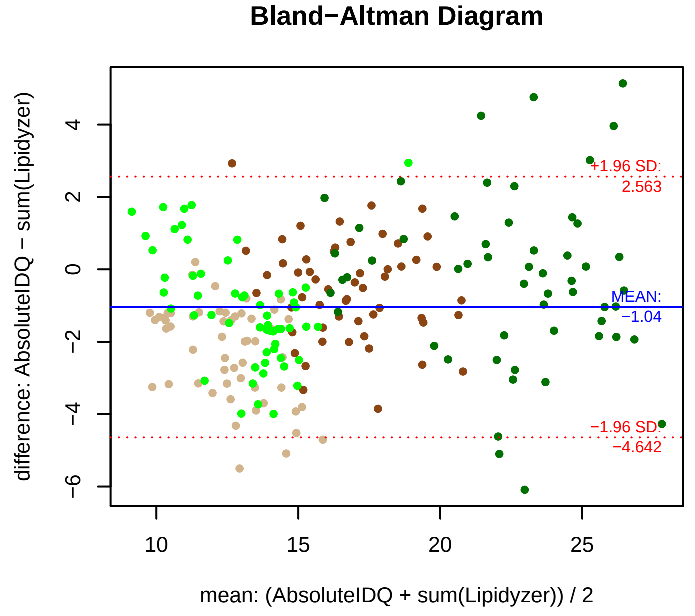

Stability of composition

(PC 14:0_20:3 + PC 16:0_18:3 + PC 18:2_16:1) / PC aa C34:3
Shapiro−Wilk Test of log quotients, pv: 0.6127; OK
SW: ok; ANOVA: Comp. ~ Subject_ID −> pv = 0.52902; EQUAL
Wilcoxon: Challenge; fasting: 0.375; sport: 0.25; OLTT: 0.375

PC 14:0_20:3 / PC aa C34:3
Shapiro−Wilk Test of log quotients, pv: 0.00108; NO
SW: ok; ANOVA: Comp. ~ Subject_ID −> pv = 4e−05; DIFFERENCE
Wilcoxon: Challenge; fasting: 0.125; sport: 0.25; OLTT: 0.25

PC 16:0_18:3 / PC aa C34:3
Shapiro−Wilk Test of log quotients, pv: 0.13301; OK
SW: ok; ANOVA: Comp. ~ Subject_ID −> pv = 0; DIFFERENCE
Wilcoxon: Challenge; fasting: 0.125; sport: 1; OLTT: 0.375

PC 18:2_16:1 / PC aa C34:3
Shapiro−Wilk Test of log quotients, pv: 0.0115; OK
SW: ok; ANOVA: Comp. ~ Subject_ID −> pv = 2e−04; DIFFERENCE
Wilcoxon: Challenge; fasting: 0.625; sport: 0.25; OLTT: 0.625

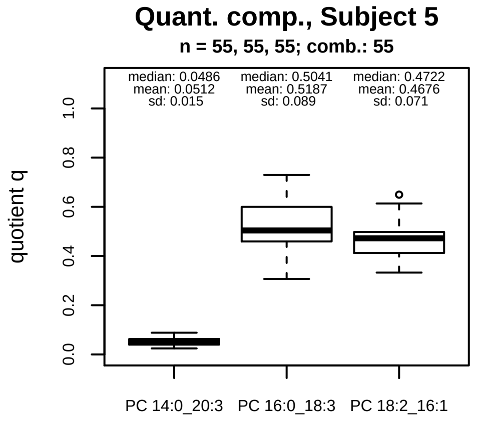

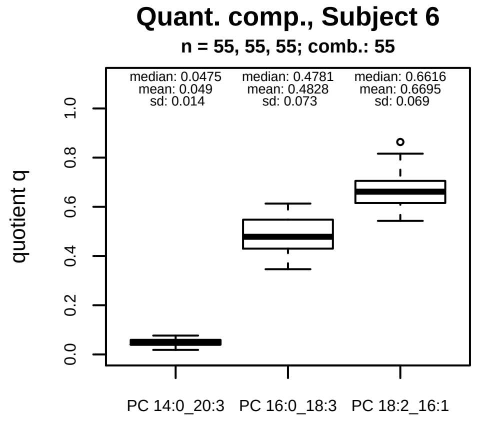

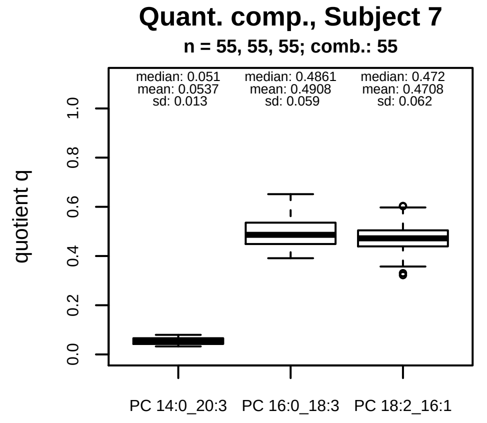

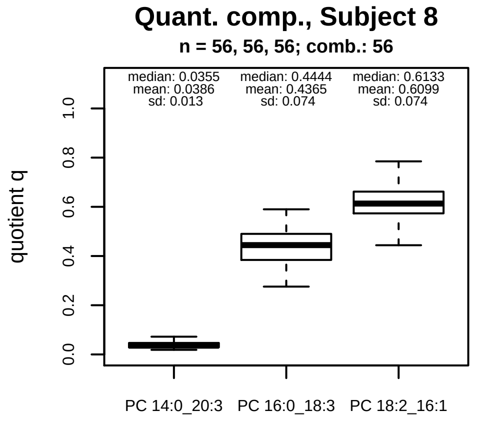

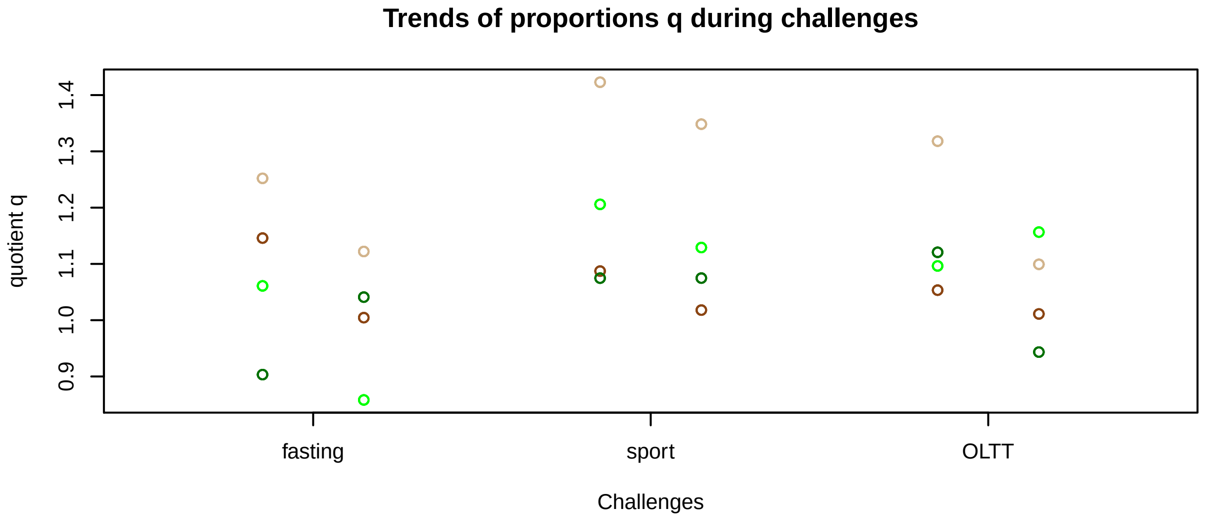

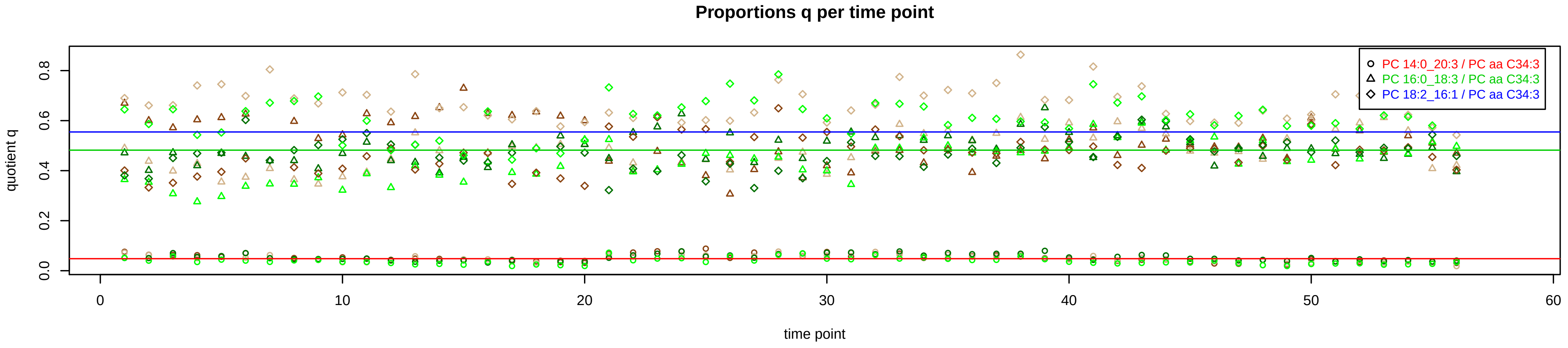

PC aa C34:4 = PC 14:0_20:4 + PC 16:0_18:4 + R

PC 16:0_18:4 excluded because of missingness > 75%

Semi−Quantitative composition within Lipidyzer

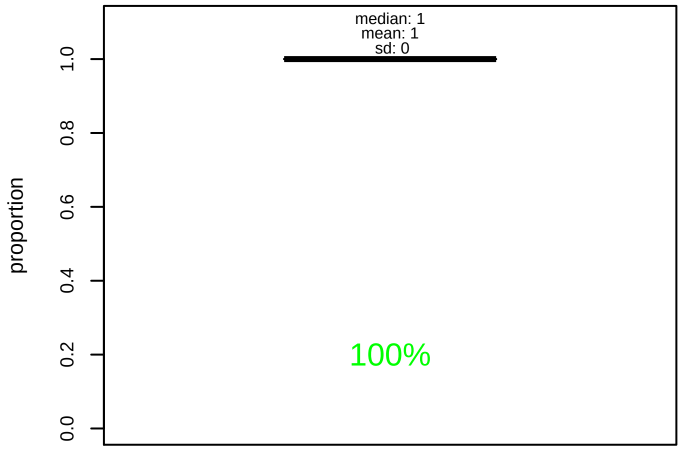

Semi−Quantitative composition quotients q

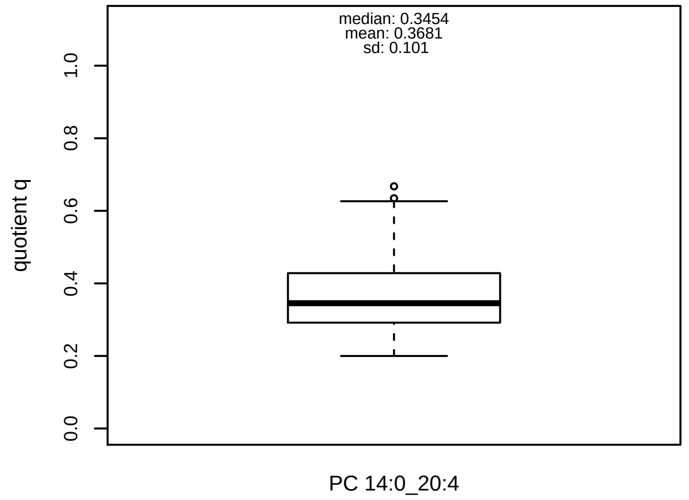

Semi−Quantitative composition: Lipidyzer

PC aa C34:4 consists of:
PC 14:0_20:4 100%
and of (not quantified):
PC 16:0_18:4, PC 12:0_22:4, PC 14:1_20:3, PC 16:1_18:3,
PC O−15:0_20:4
and further compounds

Composition: mean of proportions q

conc(PC aa C34:4) * 0.3681 = conc(PC 14:0_20:4) [var(q)=0.3681]
Percentiles: 5%−>0.2438, 25%−>0.2917, 75%−>0.4282, 95%−>0.565

Linear model

```
PC aa C34:4 ~ b * ( PC 14:0_20:4 )
```

b = 2.47409

R^2 = 0.53623

Ranges

Measure	AbsoluteIDQ	sum(Lipidyzer)	delta
Min	0.65	0.18	0.47
Max	2.72	0.96	1.77
Mean	1.41	0.49	0.92
Median	1.34	0.49	0.84
SD	0.52	0.16	0.37

Concentration: AbsoluteIDQ vs Lipidyzer

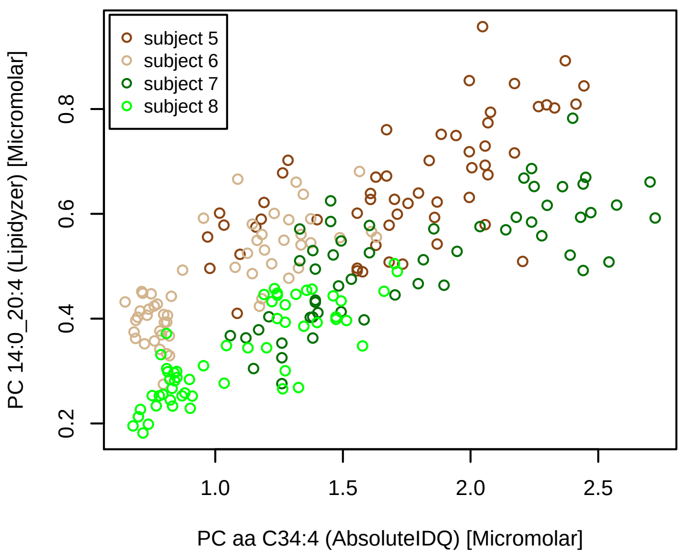

Bland−Altman Diagram

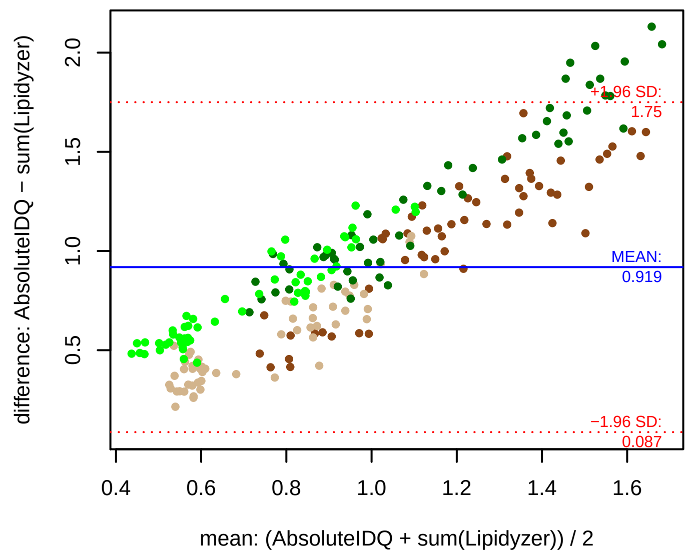

Stability of composition

PC 14:0_20:4 / PC aa C34:4
Shapiro−Wilk Test of log quotients, pv: 0.01137; OK
SW: ok; ANOVA: Comp. ~ Subject_ID −> pv = 0; DIFFERENCE
Wilcoxon: Challenge; fasting: 0.25; sport: 0.875; OLTT: 0.375

Quant. comp., Subject 5

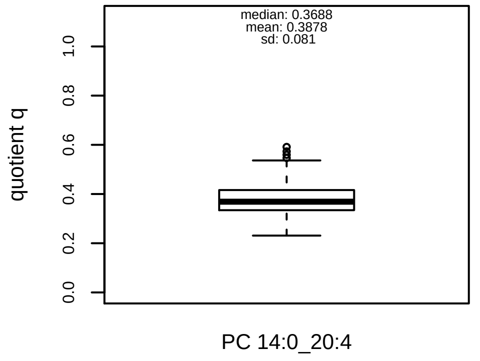

Quant. comp., Subject 6

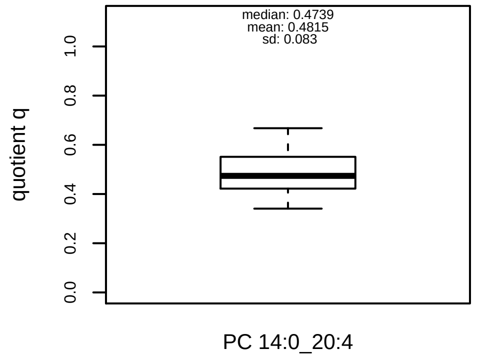

Quant. comp., Subject 7

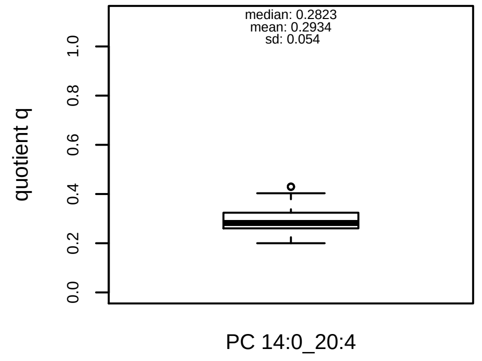

Quant. comp., Subject 8

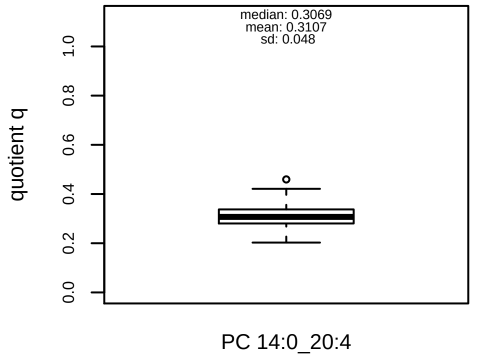

Trends of proportions q during challenges

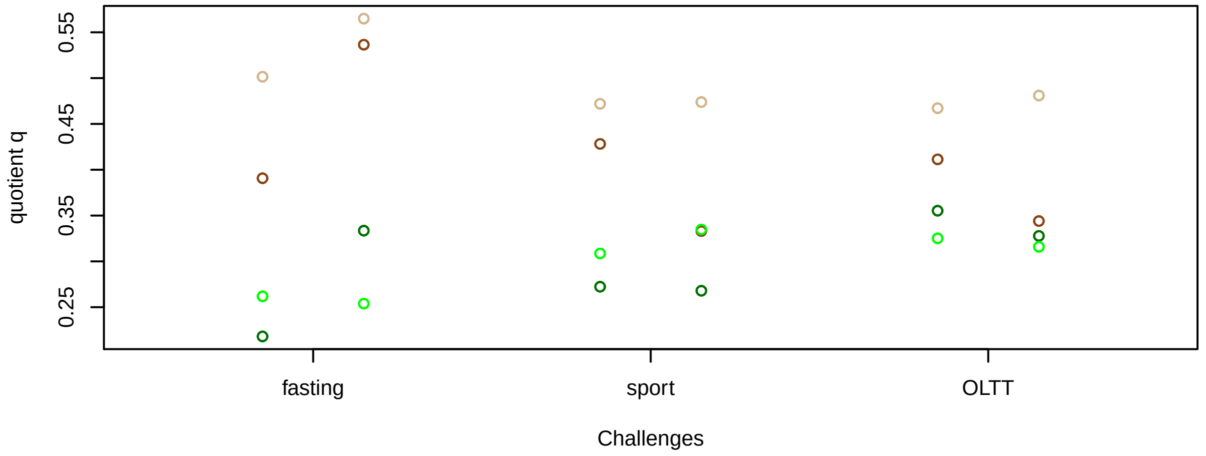

Proportions q per time point

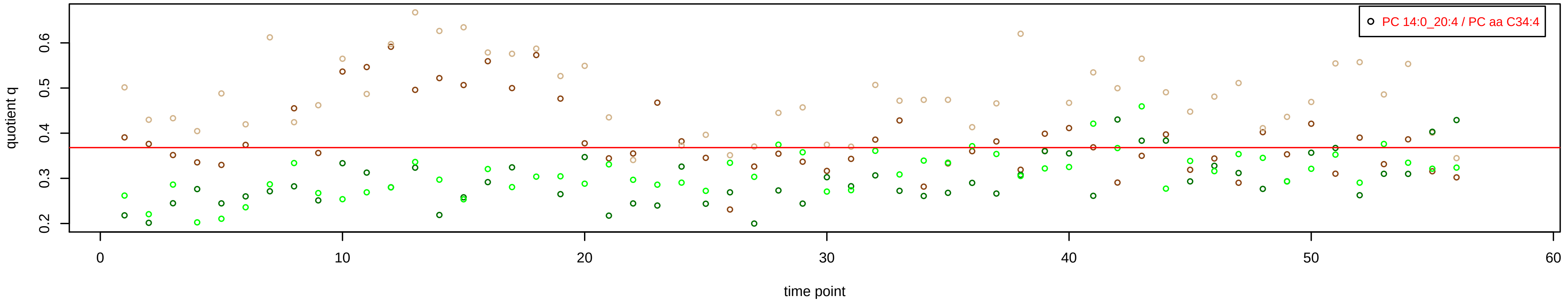

PC aa C36:0 = PC 18:0_18:0 + R

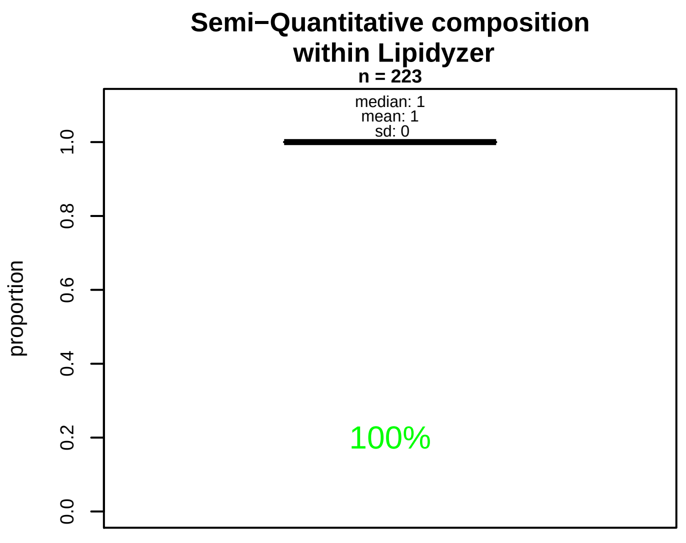

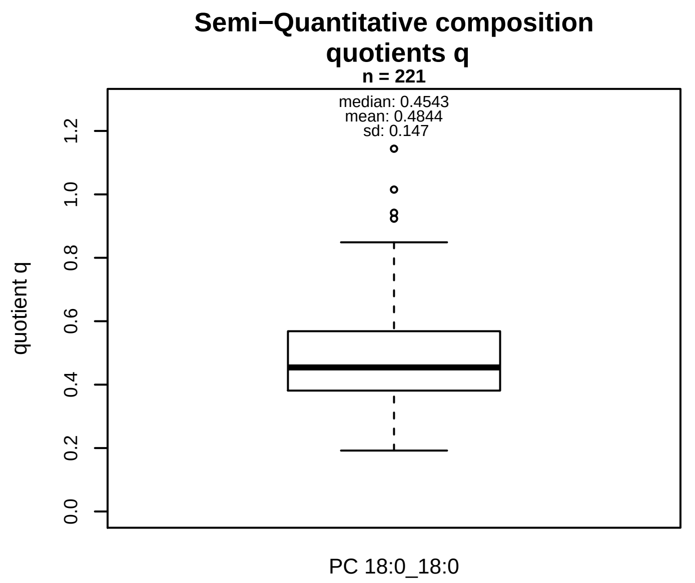

Semi−Quantitative composition: Lipidyzer

PC aa C36:0 consists of:
PC 18:0_18:0 100%
and of (not quantified):
PC 11:0_25:0, PC 12:0_24:0, PC 13:0_23:0, PC 14:0_22:0,
PC 15:0_21:0, PC 16:0_20:0, PC 17:0_19:0, PC 15:1_22:6,
PC 17:2_20:5, PC O−17:0_20:0, PC O−16:0_21:0, PC O−18:
[13C1]SM 40:0
and further compounds

Composition: mean of proportions q

conc(PC aa C36:0) * 0.4844 = conc(PC 18:0_18:0) [var(q)=0.4844]
Percentiles: 5%−>0.3066, 25%−>0.3812, 75%−>0.5685, 95%−>0.747

Linear model

```
PC aa C36:0 ~ b * ( PC 18:0_18:0 )
```

b = 0.75569

R^2 = 0.14288

Ranges

Measure	AbsoluteIDQ	sum(Lipidyzer)	delta
Min	1.69	0.56	1.12
Max	6.62	3.5	3.12
Mean	2.72	1.27	1.44
Median	2.48	1.24	1.24
SD	0.77	0.39	0.39

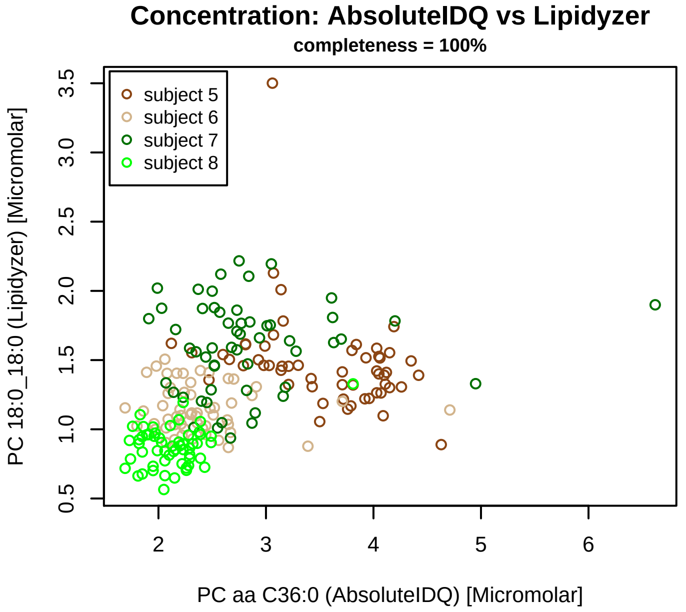

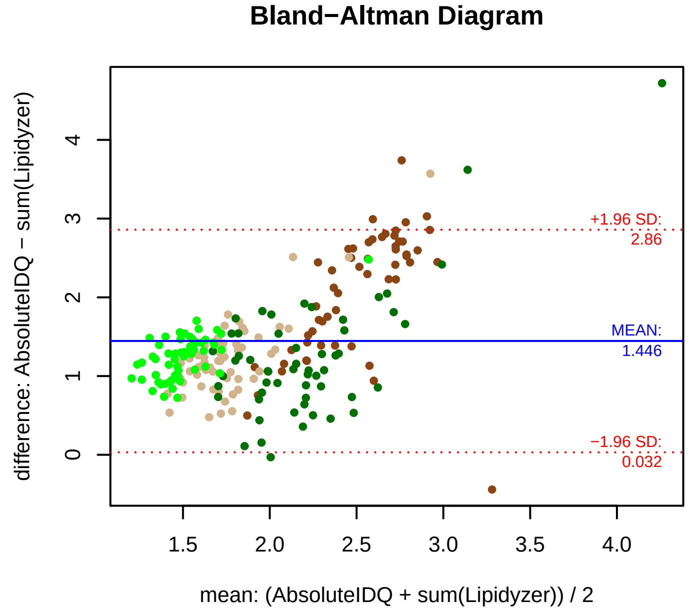

Stability of composition

PC 18:0_18:0 / PC aa C36:0
Shapiro−Wilk Test of log quotients, pv: 0.64583; OK
SW: ok; ANOVA: Comp. ~ Subject_ID −> pv = 0.477; EQUAL
Wilcoxon: Challenge; fasting: 0.625; sport: 0.25; OLTT: 0.625

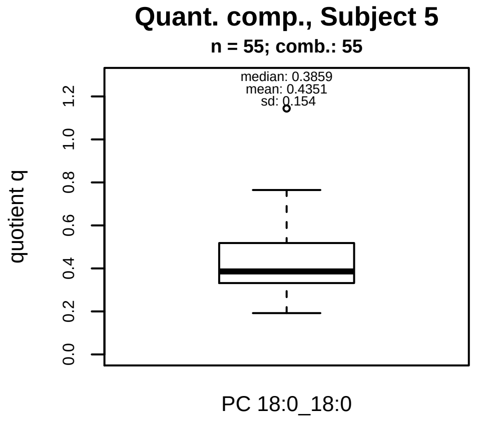

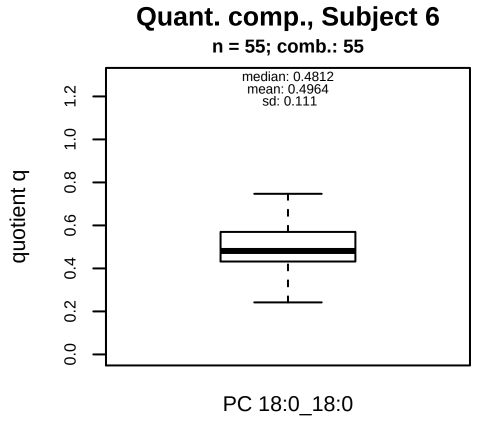

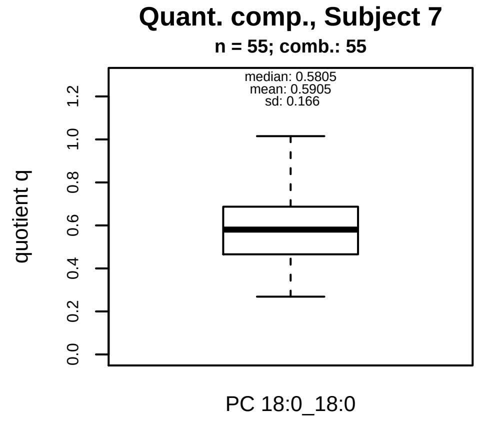

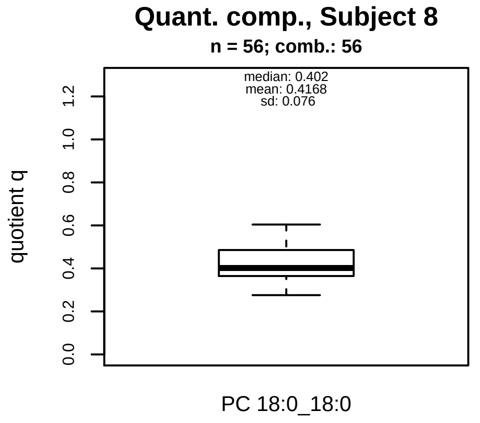

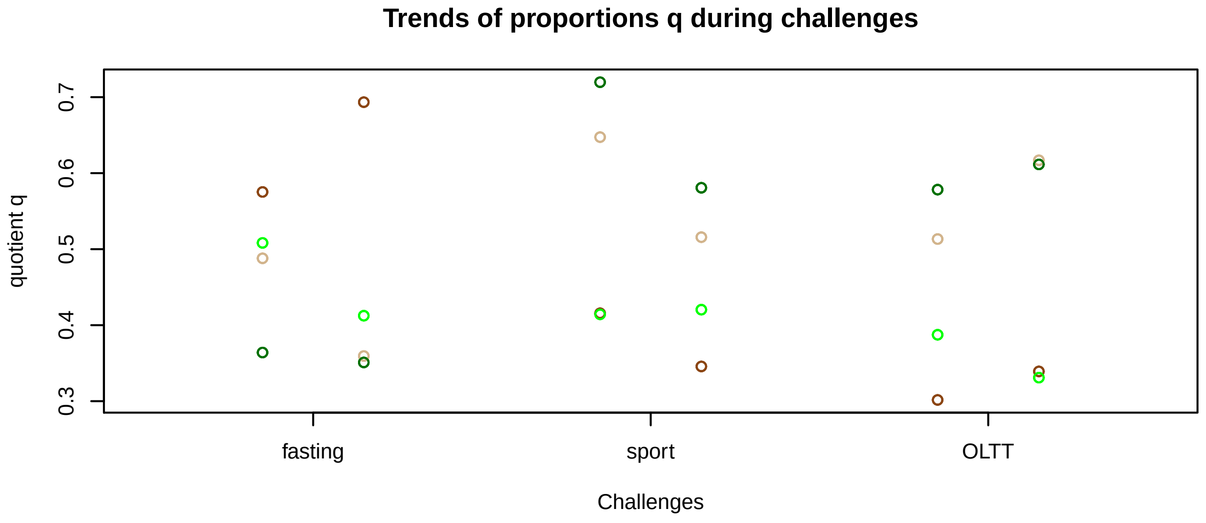

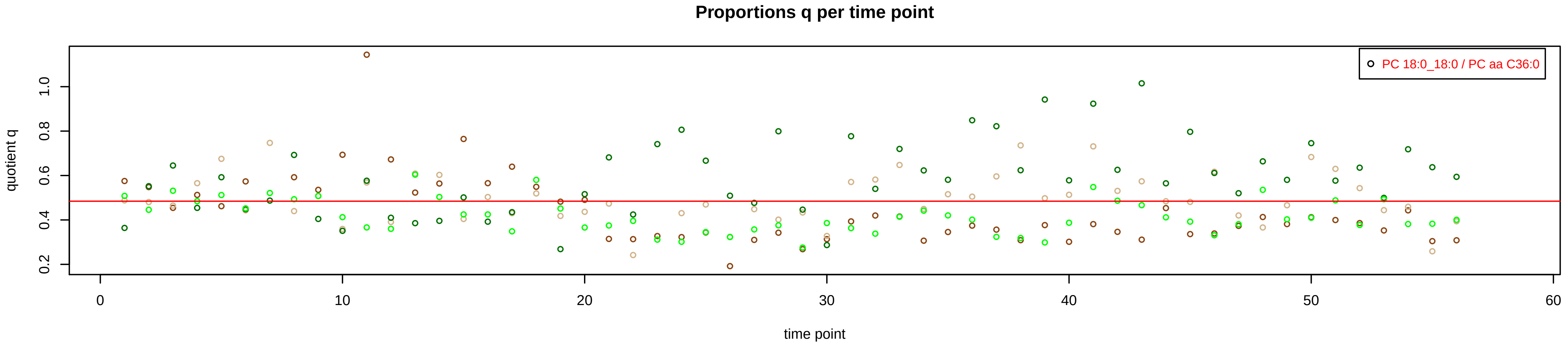

PC aa C36:1 = PC 16:0_20:1 + PC 18:0_18:1 + PC 20:0_16:1 + R

PC 20:0_16:1 excluded because of missingness > 75%

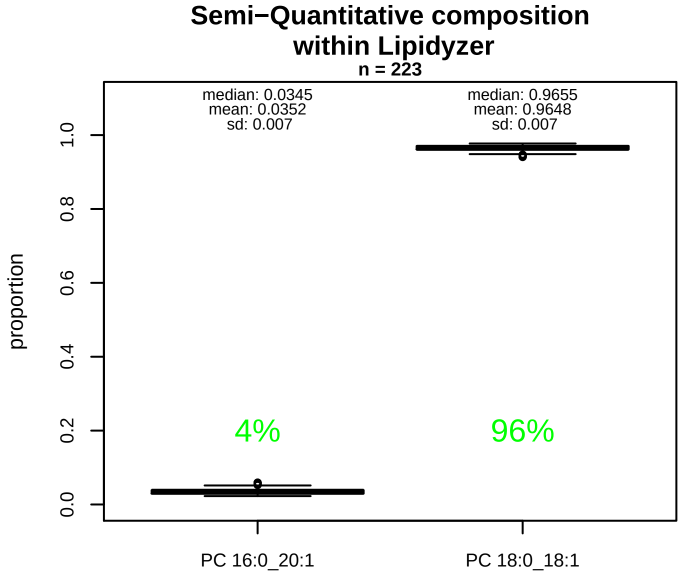

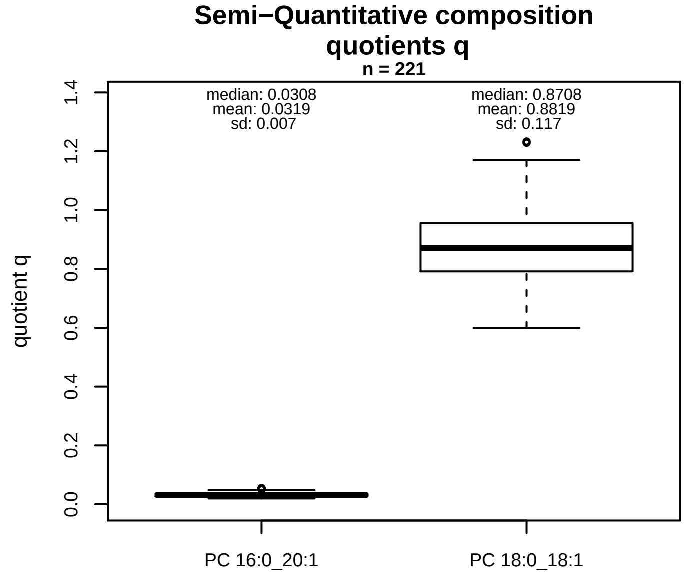

Semi−Quantitative composition: Lipidyzer

PC aa C36:1 consists of:
PC 16:0_20:1 3.5%
PC 18:0_18:1 96.5%
and of (not quantified):
PC 20:0_16:1, PC 14:0_22:1, PC 14:1_22:0, PC 15:1_21:0,
PC 16:1_20:0, PC 17:0_19:1, PC 17:1_19:0, PC O−18:0_19:[illegible]
PC O−20:0_17:1, PC O−16:1_21:0, PC O−18:1_19:0, PC O−[illegible]
[13C1]SM 40:1
and further compounds

Composition: mean of proportions q

conc(PC aa C36:1) * 0.0319 = conc(PC 16:0_20:1) [var(q)=0.0319]
Percentiles: 5%−>0.0231, 25%−>0.027, 75%−>0.0359, 95%−>0.0447
conc(PC aa C36:1) * 0.8819 = conc(PC 18:0_18:1) [var(q)=0.8819]
Percentiles: 5%−>0.7171, 25%−>0.7916, 75%−>0.9562, 95%−>1.0794

Linear model

```
PC aa C36:1 ~ b * ( PC 16:0_20:1
   + PC 18:0_18:1 )
```

b = 0.89548

R^2 = 0.7898

Ranges

Measure	AbsoluteIDQ	sum(Lipidyzer)	delta
Min	20.7	19.85	0.85
Max	68.9	68.55	0.35
Mean	41.15	37.5	3.66
Median	40.6	34.64	5.96
SD	11.29	11.16	0.13

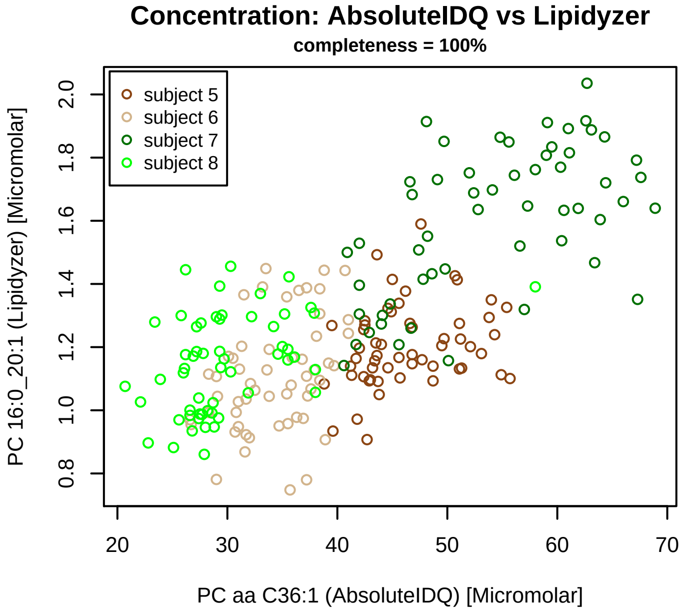

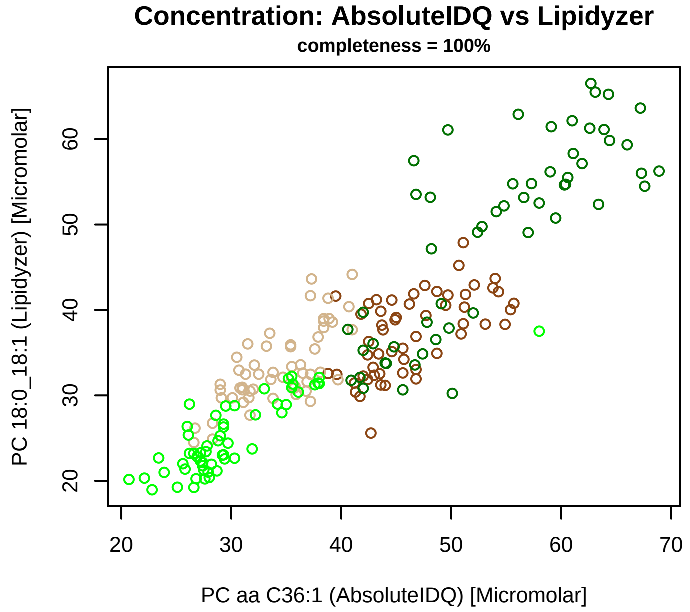

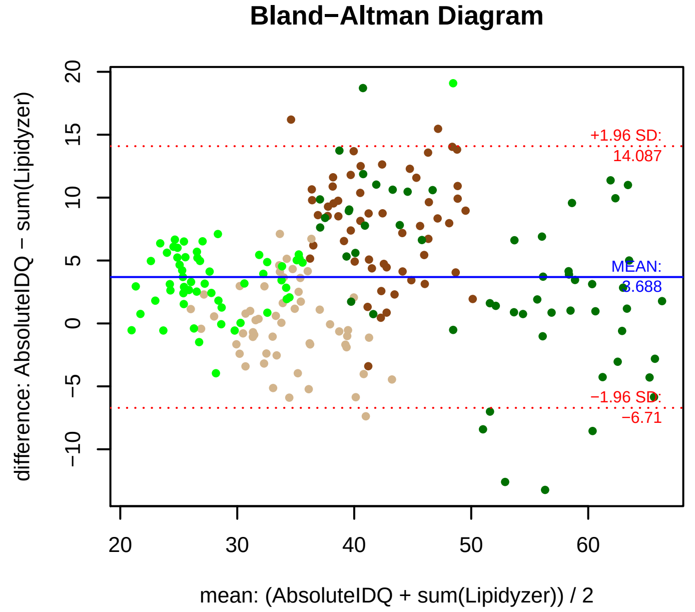

Stability of composition

(PC 16:0_20:1 + PC 18:0_18:1) / PC aa C36:1
Shapiro−Wilk Test of log quotients, pv: 0.7629; OK
SW: ok; ANOVA: Comp. ~ Subject_ID −> pv = 0.16842; EQUAL
Wilcoxon: Challenge; fasting: 0.25; sport: 0.125; OLTT: 0.875

PC 16:0_20:1 / PC aa C36:1
Shapiro−Wilk Test of log quotients, pv: 0.27012; OK
SW: ok; ANOVA: Comp. ~ Subject_ID −> pv = 0; DIFFERENCE
Wilcoxon: Challenge; fasting: 0.25; sport: 0.125; OLTT: 0.625

PC 18:0_18:1 / PC aa C36:1
Shapiro−Wilk Test of log quotients, pv: 0.66034; OK
SW: ok; ANOVA: Comp. ~ Subject_ID −> pv = 0.39144; EQUAL
Wilcoxon: Challenge; fasting: 0.25; sport: 0.125; OLTT: 0.875

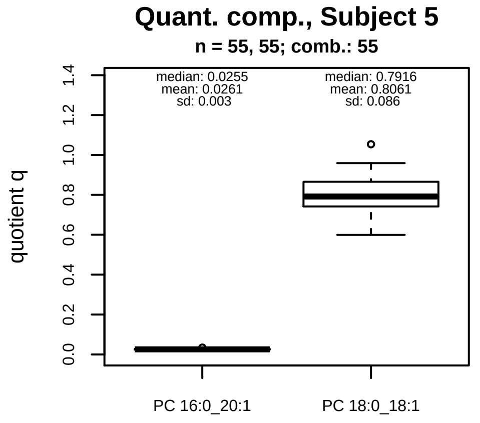

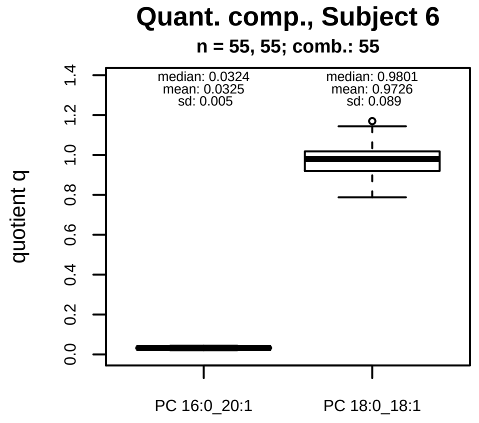

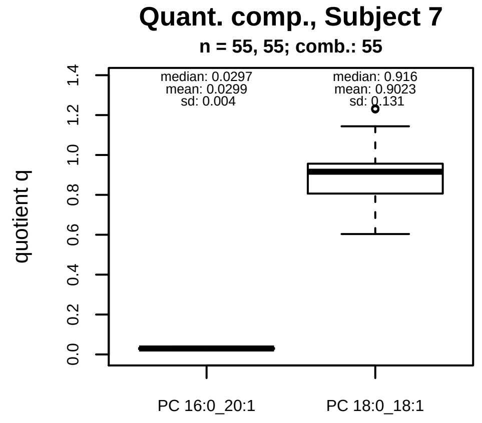

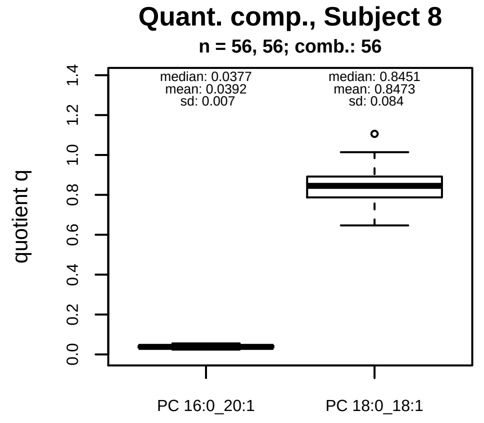

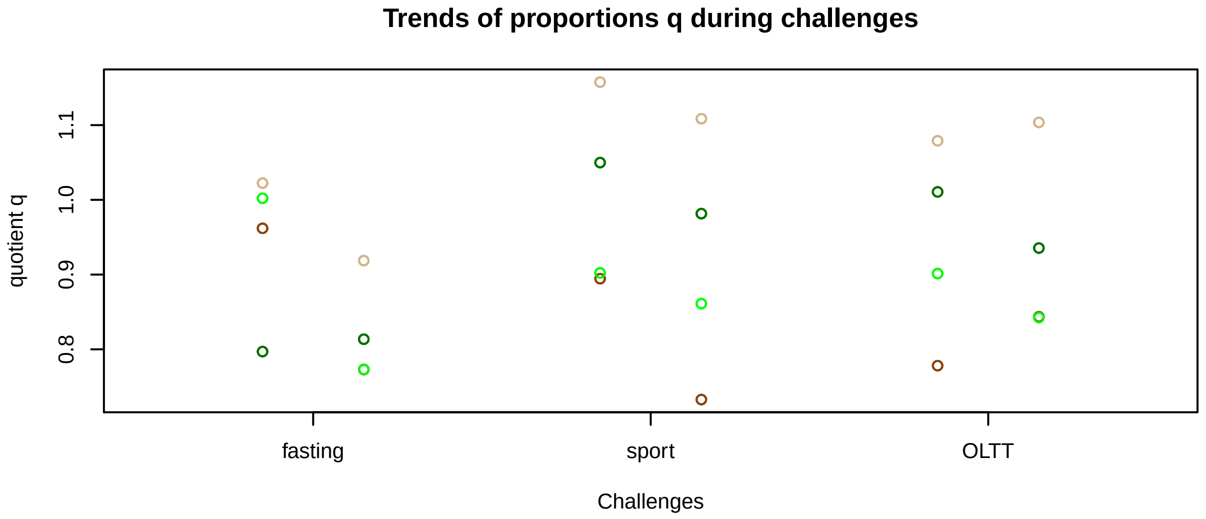

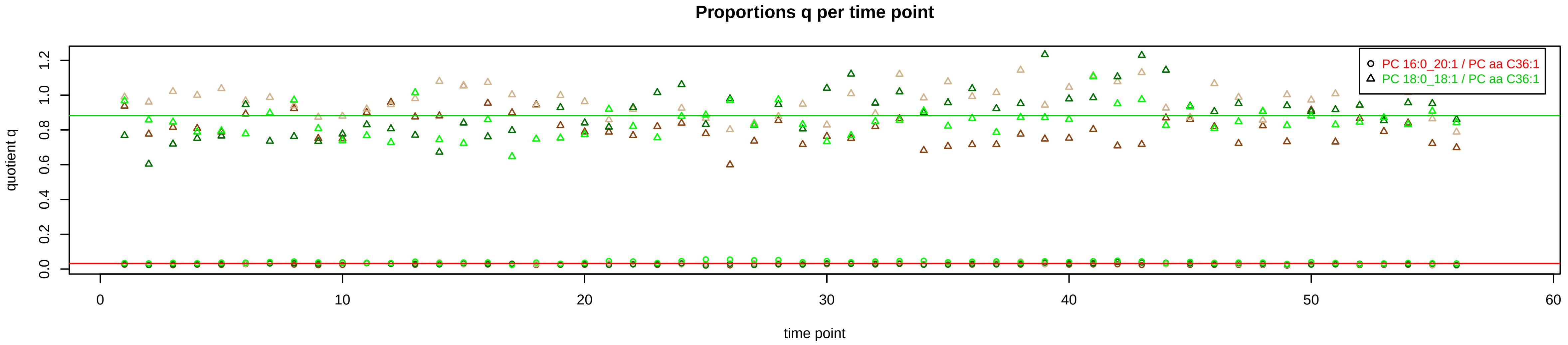

PC aa C36:2 = PC 16:0_20:2 + PC 18:0_18:2 + PC 18:1_18:1 + R

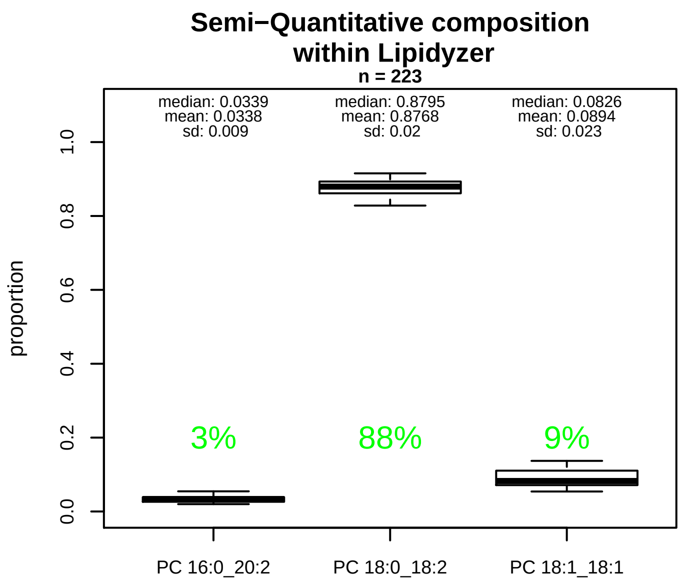

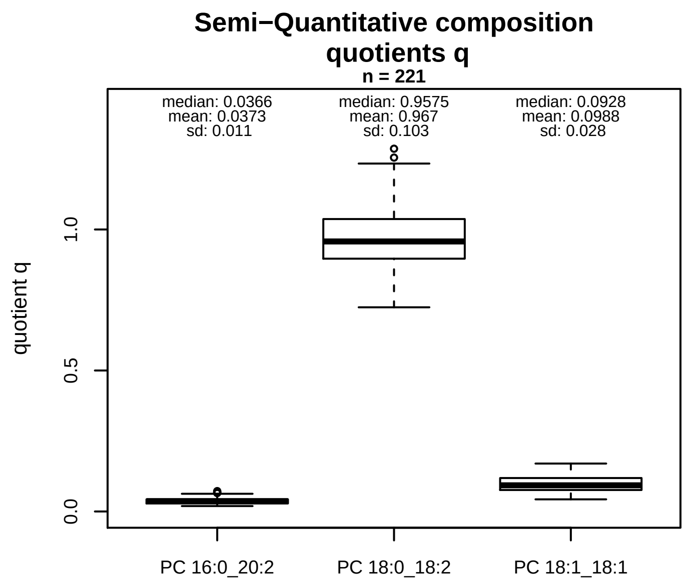

Semi−Quantitative composition: Lipidyzer

PC aa C36:2 consists of:
PC 16:0_20:2 3.4%
PC 18:0_18:2 87.7%
PC 18:1_18:1 8.9%
and of (not quantified):
PC14 0 22:2, PC14 1 22:1, PC16 1 20:1, PC17 1 19:1,
PC17 2 19:0, PCO−20 0 17:2, PCO−18 1 19:1, PCO−20 1 17
[13C1]SM 40:2
and further compounds

Composition: mean of proportions q

conc(PC aa C36:2) * 0.0373 = conc(PC 16:0_20:2) [var(q)=0.0373]
Percentiles: 5%−>0.0226, 25%−>0.0285, 75%−>0.0432, 95%−>0.0581
conc(PC aa C36:2) * 0.967 = conc(PC 18:0_18:2) [var(q)=0.967]
Percentiles: 5%−>0.8172, 25%−>0.8964, 75%−>1.037, 95%−>1.1362
conc(PC aa C36:2) * 0.0988 = conc(PC 18:1_18:1) [var(q)=0.0988]
Percentiles: 5%−>0.0601, 25%−>0.0762, 75%−>0.1186, 95%−>0.1467

Linear model

```
PC aa C36:2 ~ b * ( PC 16:0_20:2
  + PC 18:0_18:2
  + PC 18:1_18:1 )
```

b = 0.6305

R^2 = 0.57943

Ranges

Measure	AbsoluteIDQ	sum(Lipidyzer)	delta
Min	125.5	141.63	16.13
Max	256.9	302.37	45.47
Mean	190.68	209.51	18.83
Median	189.6	204.41	14.81
SD	27.84	33.51	5.67

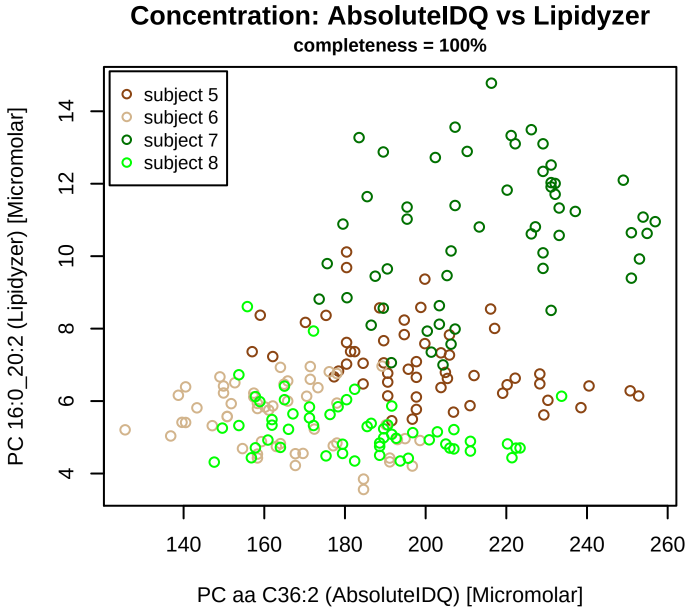

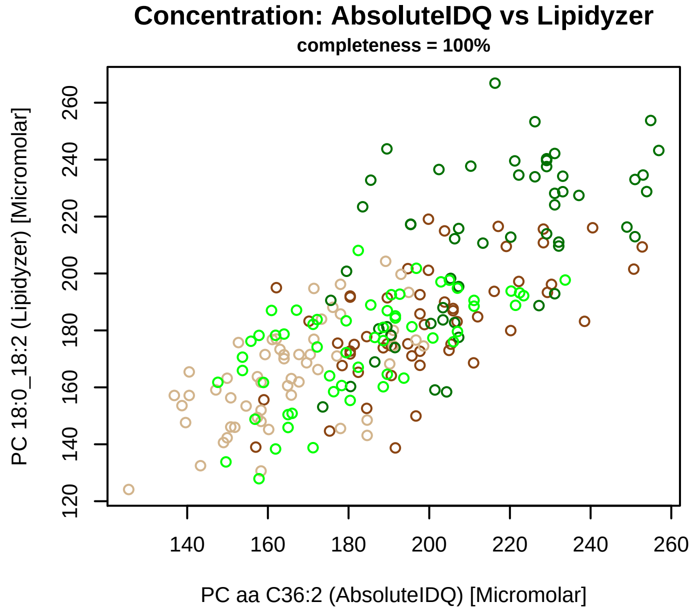

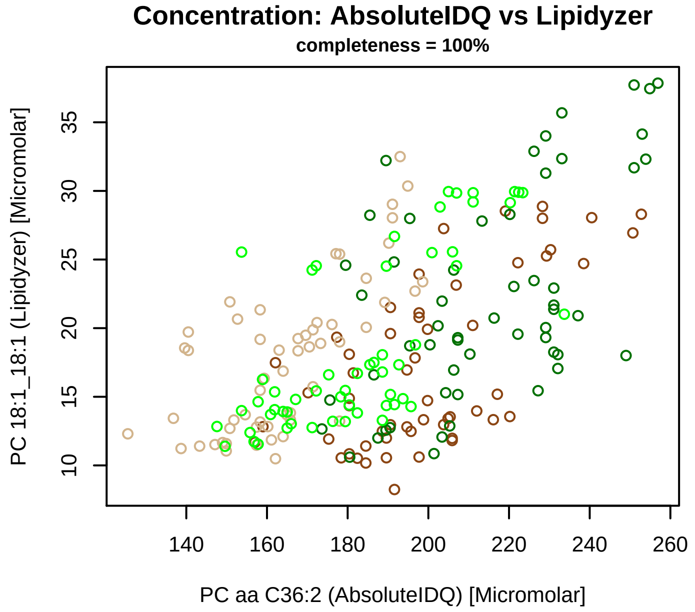

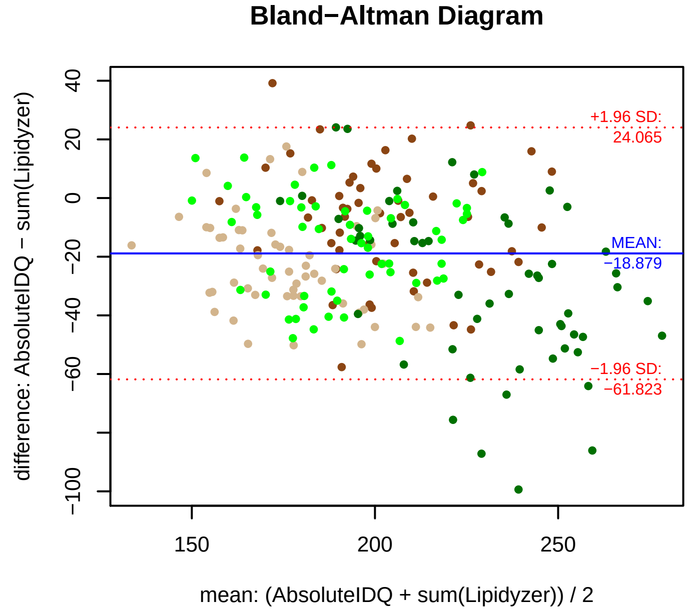

Stability of composition

(PC 16:0_20:2 + PC 18:0_18:2 + PC 18:1_18:1) / PC aa C36:2
Shapiro−Wilk Test of log quotients, pv: 0.68759; OK
SW: ok; ANOVA: Comp. ~ Subject_ID −> pv = 0.03886; EQUAL
Wilcoxon: Challenge; fasting: 0.25; sport: 0.125; OLTT: 0.375

PC 16:0_20:2 / PC aa C36:2
Shapiro−Wilk Test of log quotients, pv: 0.06915; OK
SW: no; Kruskal: Comp. ~ Subject_ID −> pv = 0; DIFFERENCE
Wilcoxon: Challenge; fasting: 0.375; sport: 0.125; OLTT: 0.125

PC 18:0_18:2 / PC aa C36:2
Shapiro−Wilk Test of log quotients, pv: 0.72516; OK
SW: ok; ANOVA: Comp. ~ Subject_ID −> pv = 0.08316; EQUAL
Wilcoxon: Challenge; fasting: 0.125; sport: 0.125; OLTT: 0.375

PC 18:1_18:1 / PC aa C36:2
Shapiro−Wilk Test of log quotients, pv: 0.00285; NO
SW: no; Kruskal: Comp. ~ Subject_ID −> pv = 0.00031; DIFFERENCE
Wilcoxon: Challenge; fasting: 0.875; sport: 0.125; OLTT: 0.375

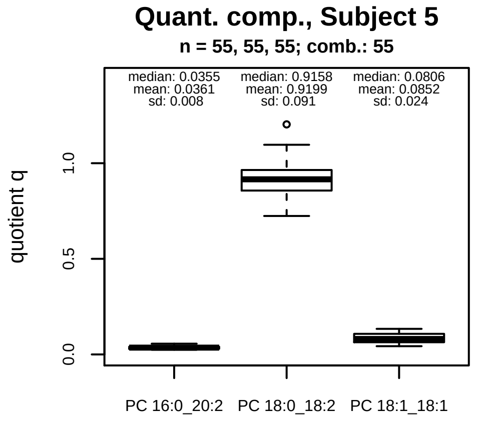

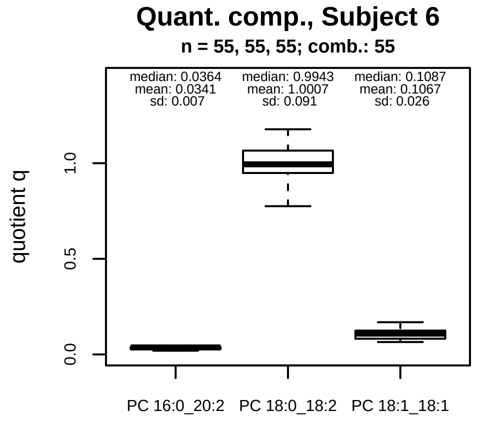

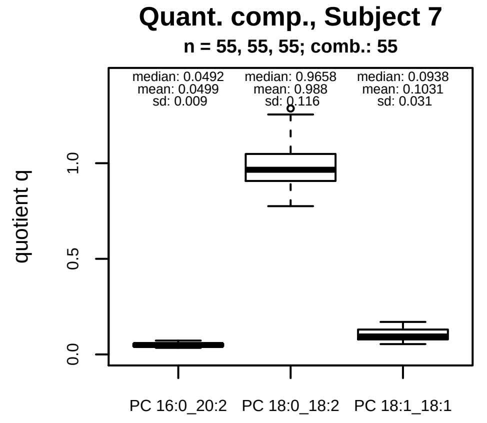

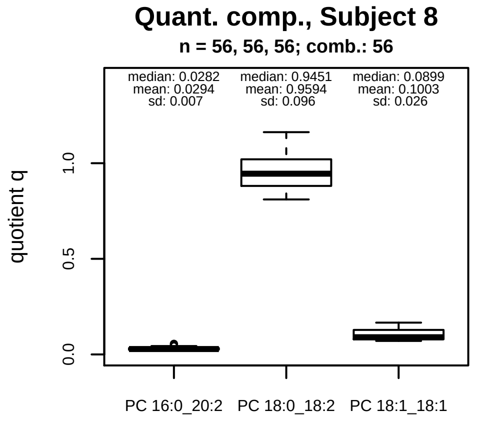

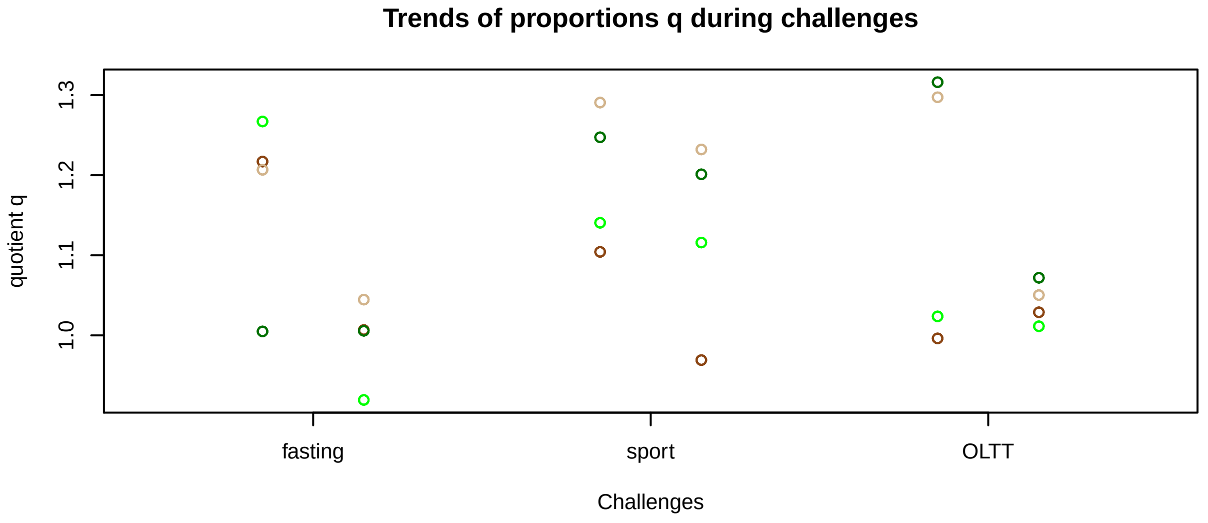

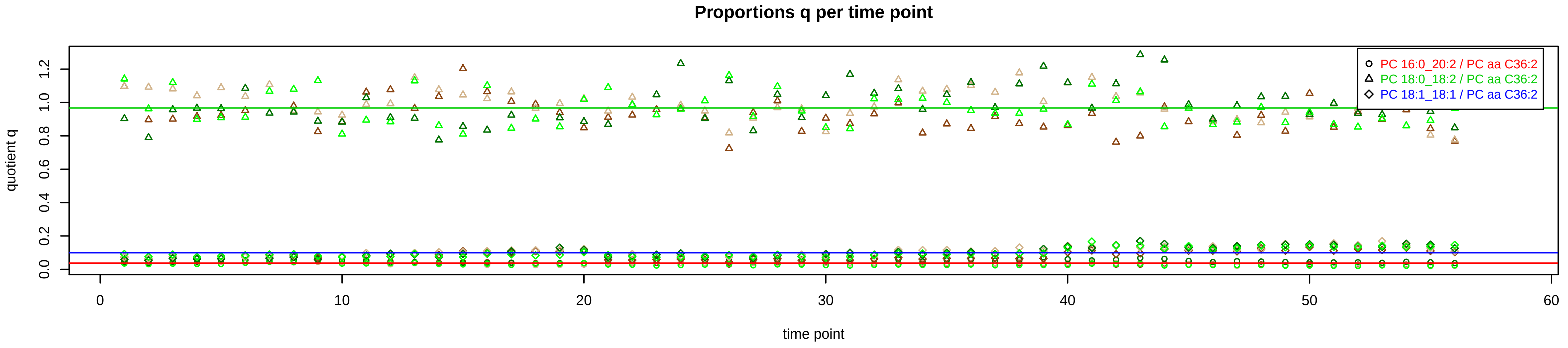

PC aa C36:3 = PC 16:0_20:3 + PC 18:0_18:3 + PC 18:1_18:2 + R

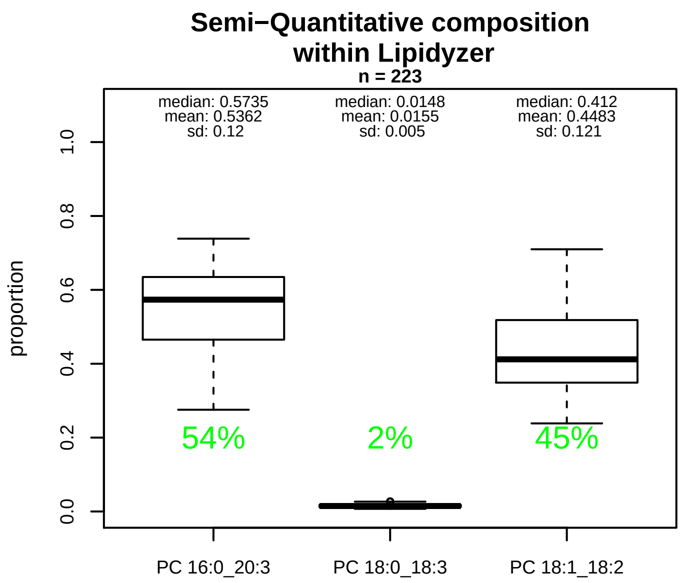

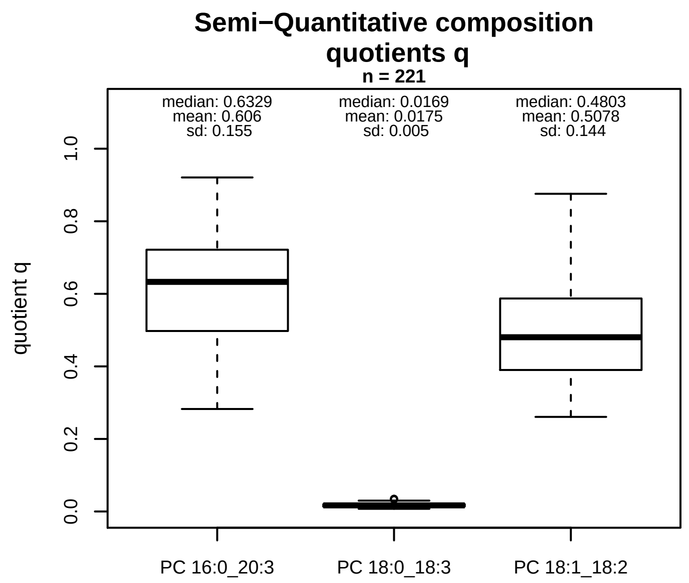

Semi−Quantitative composition: Lipidyzer

PC aa C36:3 consists of:
PC 16:0_20:3 53.6%
PC 18:0_18:3 1.5%
PC 18:1_18:2 44.8%
and of (not quantified):
PC14 1 22:2, PC16 1 20:2, PC17 2 19:1, PCO−20 1 17:2, SM40.3
and further compounds

Linear model

```
PC aa C36:3 ~ b * ( PC 16:0_20:3
   + PC 18:0_18:3
   + PC 18:1_18:2 )
```

b = 0.79714

R^2 = 0.80444

Ranges

Measure	AbsoluteIDQ	sum(Lipidyzer)	delta
Min	84.1	86.21	2.11
Max	222	250.69	28.69
Mean	133.31	150	16.69
Median	126	141.27	15.27
SD	31.67	35.54	3.87

Composition: mean of proportions q

conc(PC aa C36:3) * 0.606 = conc(PC 16:0_20:3) [var(q)=0.606]
Percentiles: 5%−>0.3434, 25%−>0.4975, 75%−>0.7216, 95%−>0.8409
conc(PC aa C36:3) * 0.0175 = conc(PC 18:0_18:3) [var(q)=0.0175]
Percentiles: 5%−>0.01, 25%−>0.0137, 75%−>0.0203, 95%−>0.0279
conc(PC aa C36:3) * 0.5078 = conc(PC 18:1_18:2) [var(q)=0.5078]
Percentiles: 5%−>0.3177, 25%−>0.3901, 75%−>0.5871, 95%−>0.7643

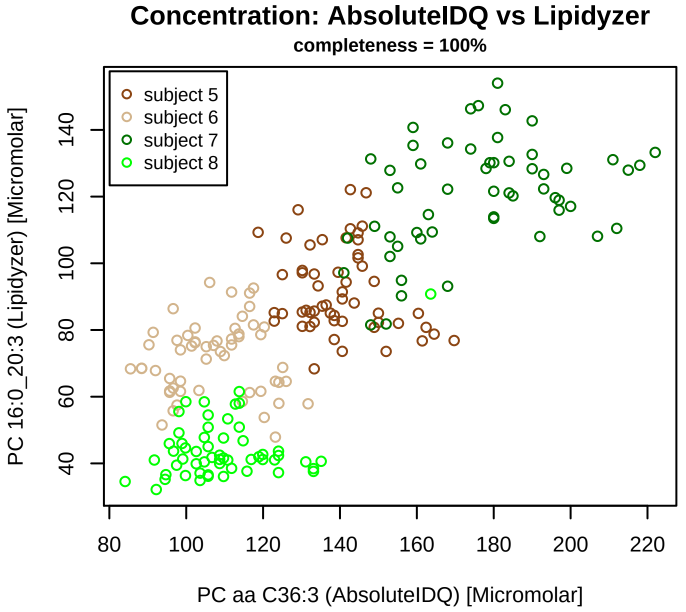

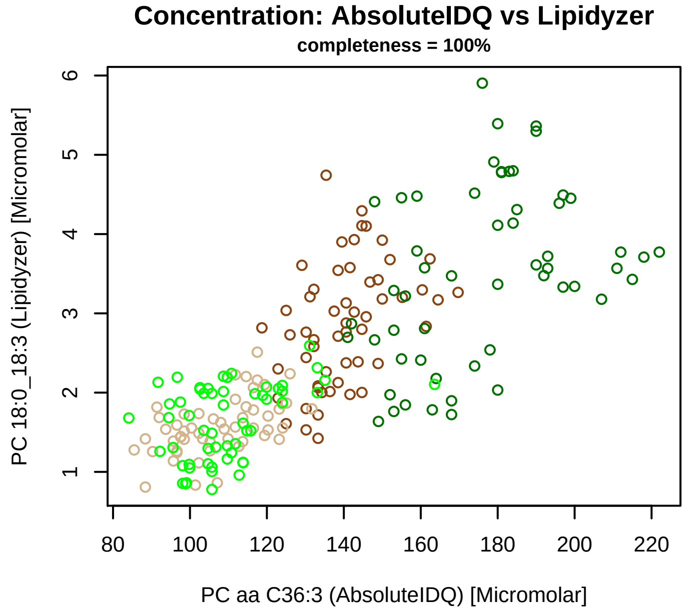

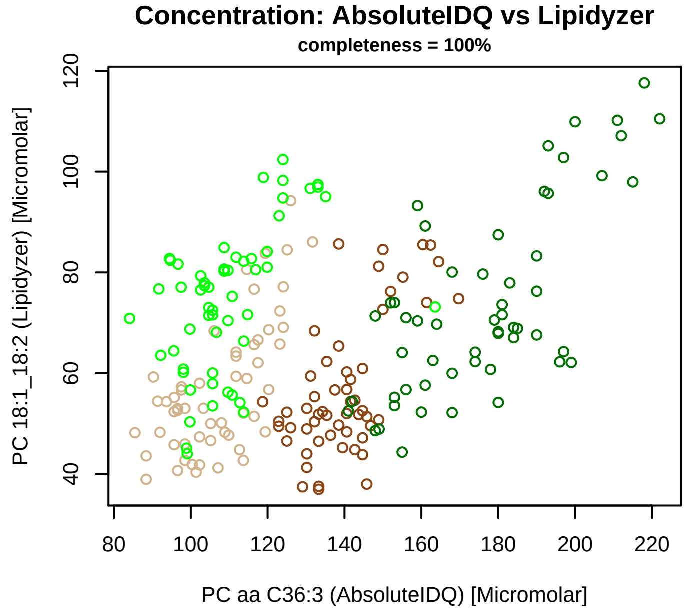

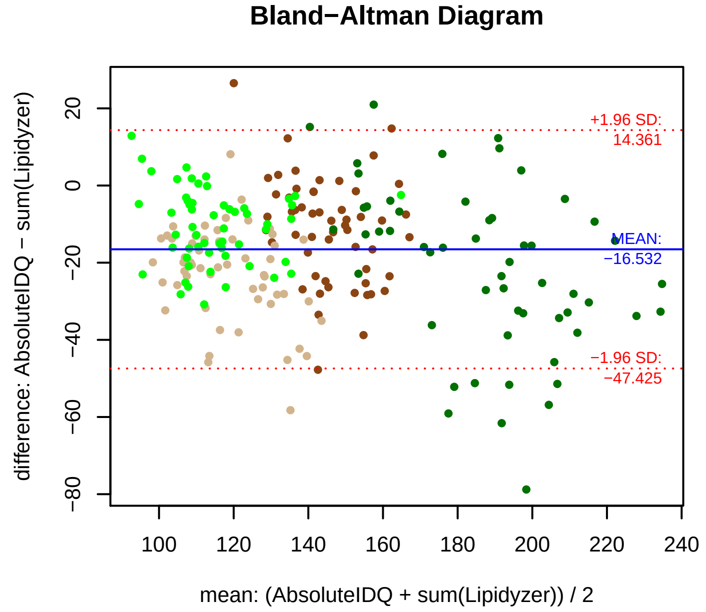

Stability of composition

(PC 16:0_20:3 + PC 18:0_18:3 + PC 18:1_18:2) / PC aa C36:3
Shapiro−Wilk Test of log quotients, pv: 0.43192; OK
SW: ok; ANOVA: Comp. ~ Subject_ID −> pv = 0.6275; EQUAL
Wilcoxon: Challenge; fasting: 0.25; sport: 0.125; OLTT: 0.25

PC 16:0_20:3 / PC aa C36:3
Shapiro−Wilk Test of log quotients, pv: 0; NO
SW: no; Kruskal: Comp. ~ Subject_ID −> pv = 0; DIFFERENCE
Wilcoxon: Challenge; fasting: 0.375; sport: 0.125; OLTT: 0.125

PC 18:0_18:3 / PC aa C36:3
Shapiro−Wilk Test of log quotients, pv: 0.6707; OK
SW: ok; ANOVA: Comp. ~ Subject_ID −> pv = 0.00016; DIFFERENCE
Wilcoxon: Challenge; fasting: 0.125; sport: 0.625; OLTT: 0.625

PC 18:1_18:2 / PC aa C36:3
Shapiro−Wilk Test of log quotients, pv: 0.00353; NO
SW: no; Kruskal: Comp. ~ Subject_ID −> pv = 0; DIFFERENCE
Wilcoxon: Challenge; fasting: 0.125; sport: 0.125; OLTT: 1

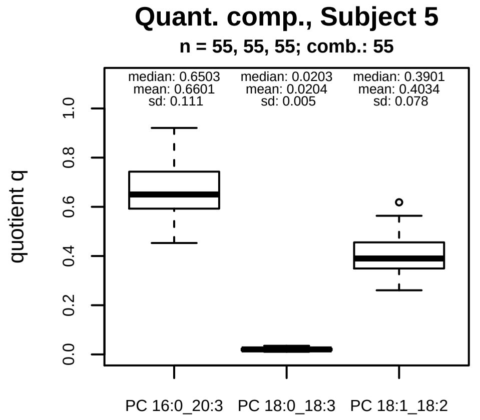

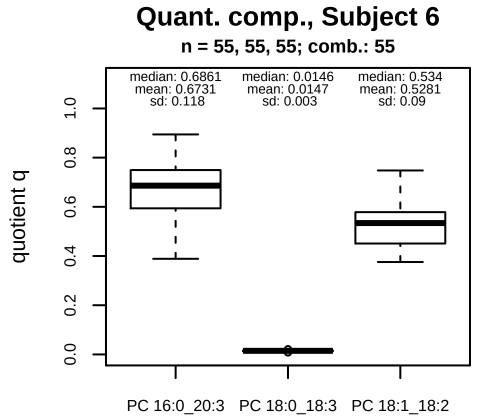

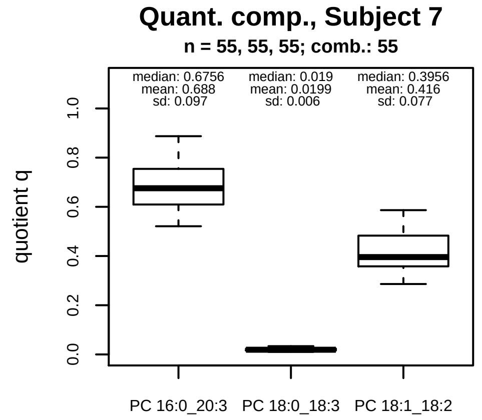

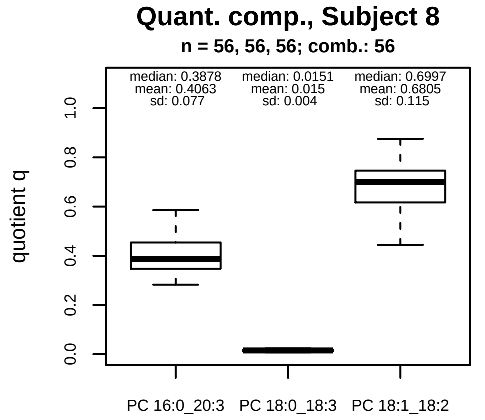

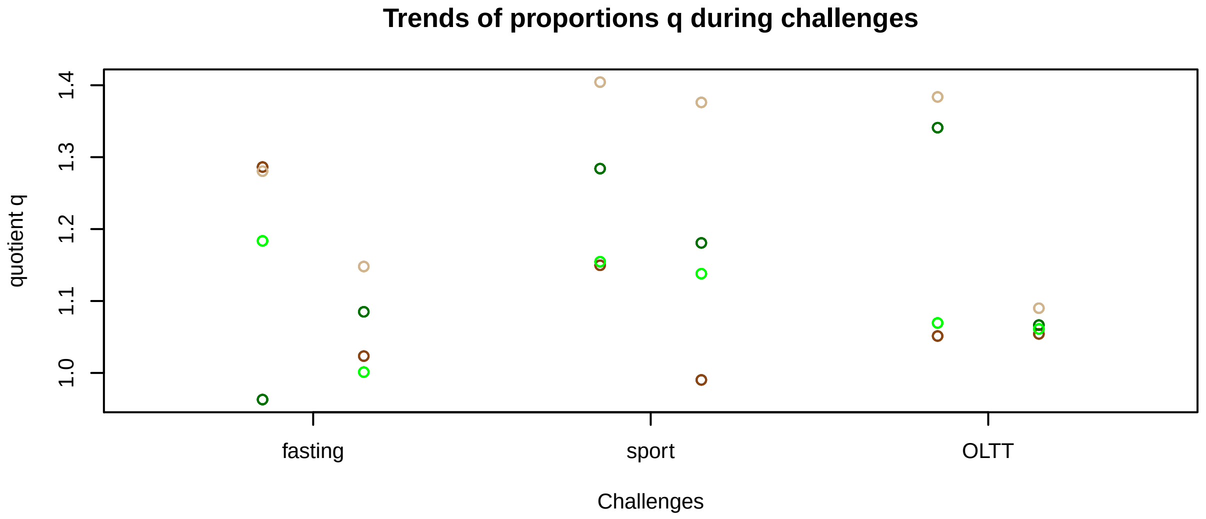

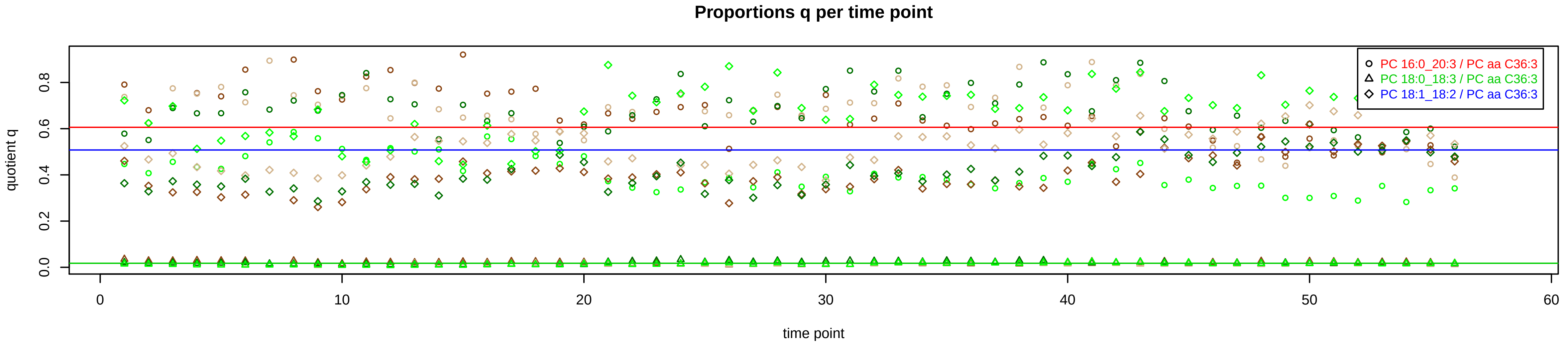

PC aa C36:4 = PC 14:0_22:4 + PC 16:0_20:4 + PC 18:0_18:4 + PC 18:1_18:3 + PC 18:2_18:2 + R

PC 18:0_18:4 excluded because of missingness > 75%

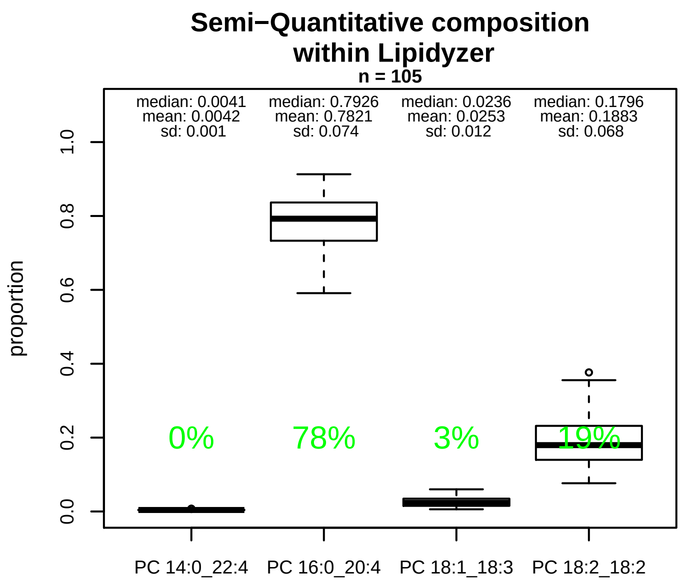

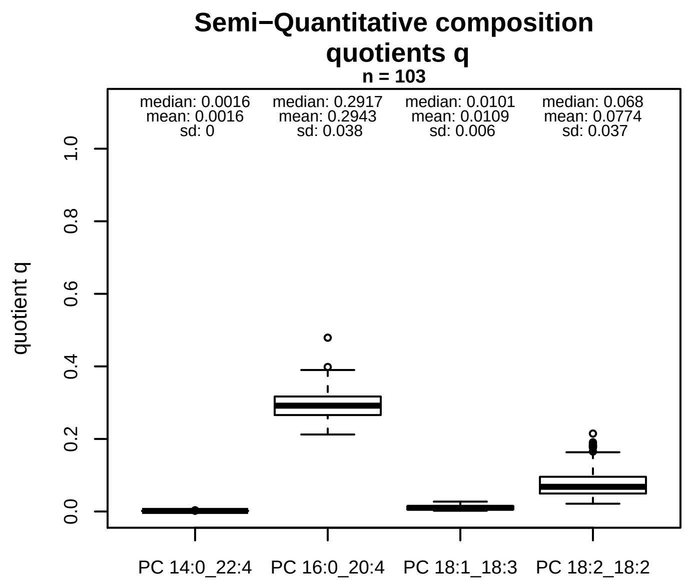

Semi−Quantitative composition: Lipidyzer

PC aa C36:4 consists of:
PC 14:0_22:4 0.4%
PC 16:0_20:4 78.2%
PC 18:1_18:3 2.5%
PC 18:2_18:2 18.8%
and of (not quantified):
PC O−17:0_20:4
and further compounds

Linear model

```
PC aa C36:4 ~ b * ( PC 14:0_22:4
  + PC 16:0_20:4
  + PC 18:1_18:3
  + PC 18:2_18:2 )
```

b = 2.55262

R^2 = 0.65155

Ranges

Measure	AbsoluteIDQ	sum(Lipidyzer)	delta
Min	101.4	47.02	54.38
Max	290.2	98.75	191.45
Mean	180.31	70.22	110.09
Median	179.9	69.92	109.98
SD	35.18	10.41	24.76

Composition: mean of proportions q

conc(PC aa C36:4) * 0.0016 = conc(PC 14:0_22:4) [var(q)=0.0016]
Percentiles: 5%−>0.0011, 25%−>0.0013, 75%−>0.0018, 95%−>0.0025
conc(PC aa C36:4) * 0.2943 = conc(PC 16:0_20:4) [var(q)=0.2943]
Percentiles: 5%−>0.2393, 25%−>0.2658, 75%−>0.317, 95%−>0.3555
conc(PC aa C36:4) * 0.0109 = conc(PC 18:1_18:3) [var(q)=0.0109]
Percentiles: 5%−>0.0033, 25%−>0.0056, 75%−>0.0147, 95%−>0.022
conc(PC aa C36:4) * 0.0774 = conc(PC 18:2_18:2) [var(q)=0.0774]
Percentiles: 5%−>0.0354, 25%−>0.0496, 75%−>0.0957, 95%−>0.1558

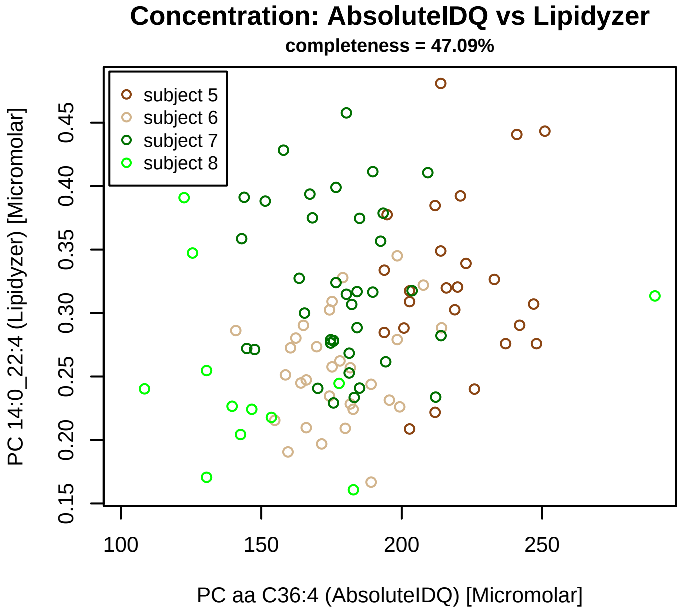

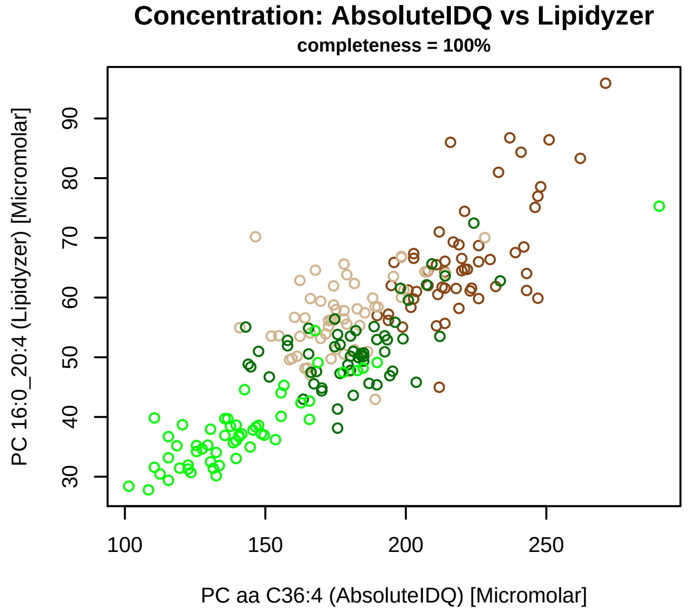

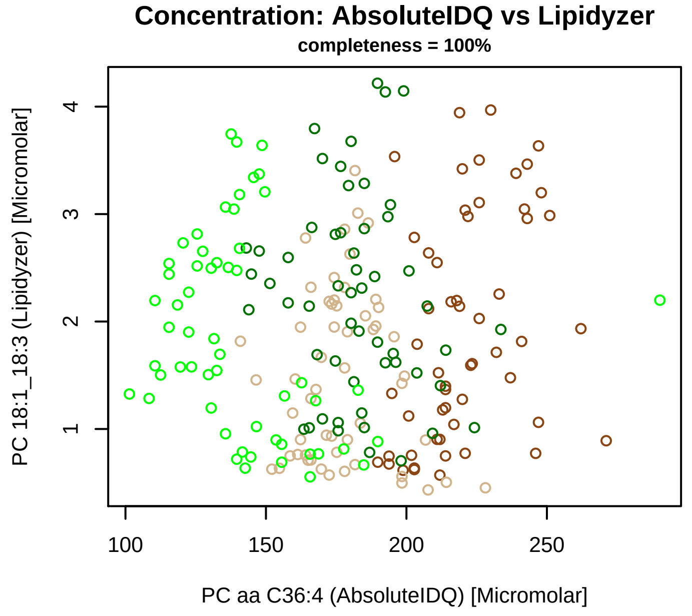

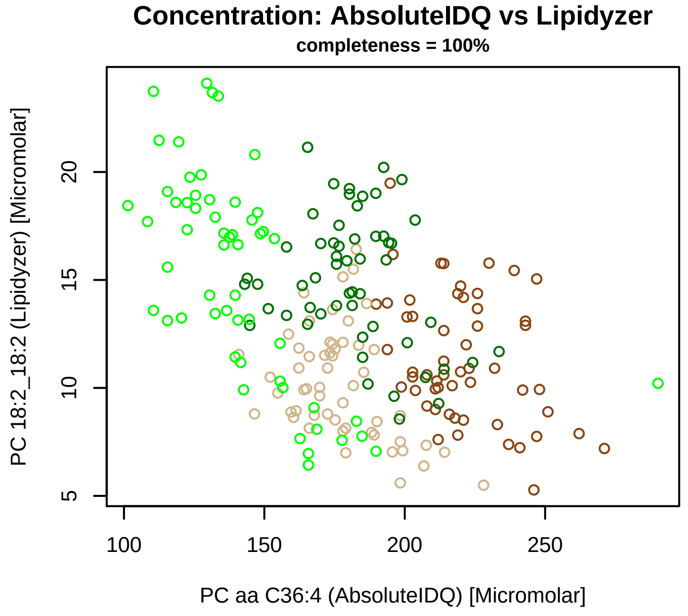

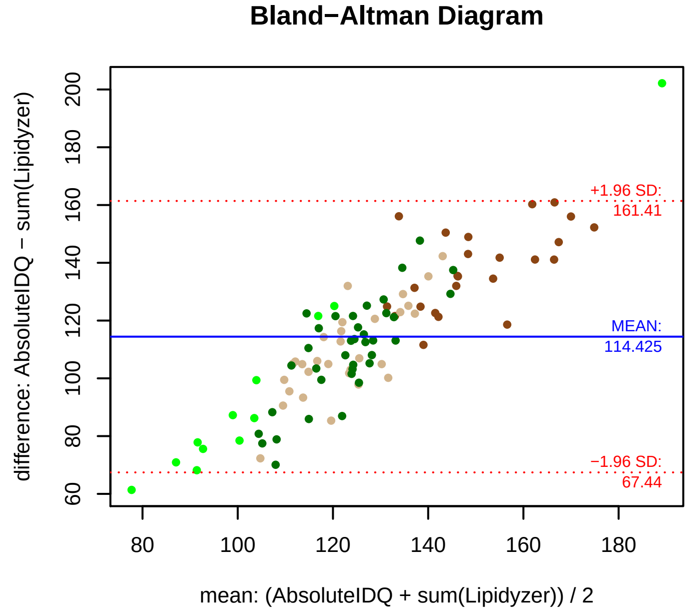

Stability of composition

(PC 14:0_22:4 + PC 16:0_20:4 + PC 18:1_18:3 + PC 18:2_18:2) / PC aa C36:4
Shapiro−Wilk Test of log quotients, pv: 0.91369; OK
SW: ok; ANOVA: Comp. ~ Subject_ID −> pv = 0.21225; EQUAL
Wilcoxon: Challenge; sport: 0.5; OLTT: 1

PC 14:0_22:4 / PC aa C36:4
Shapiro−Wilk Test of log quotients, pv: 0.48012; OK
SW: ok; ANOVA: Comp. ~ Subject_ID −> pv = 0.00455; DIFFERENCE
Wilcoxon: Challenge; sport: 0.5; OLTT: 1

PC 16:0_20:4 / PC aa C36:4
Shapiro−Wilk Test of log quotients, pv: 0.22093; OK
SW: no; Kruskal: Comp. ~ Subject_ID −> pv = 0; DIFFERENCE
Wilcoxon: Challenge; fasting: 0.875; sport: 0.25; OLTT: 0.25

PC 18:1_18:3 / PC aa C36:4
Shapiro−Wilk Test of log quotients, pv: 2e−05; NO
SW: no; Kruskal: Comp. ~ Subject_ID −> pv = 0; DIFFERENCE
Wilcoxon: Challenge; fasting: 0.125; sport: 1; OLTT: 0.625

PC 18:2_18:2 / PC aa C36:4
Shapiro−Wilk Test of log quotients, pv: 0.52175; OK
SW: no; Kruskal: Comp. ~ Subject_ID −> pv = 0; DIFFERENCE
Wilcoxon: Challenge; fasting: 0.125; sport: 0.125; OLTT: 0.625

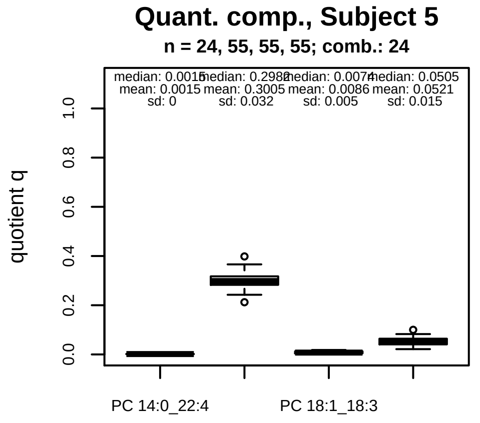

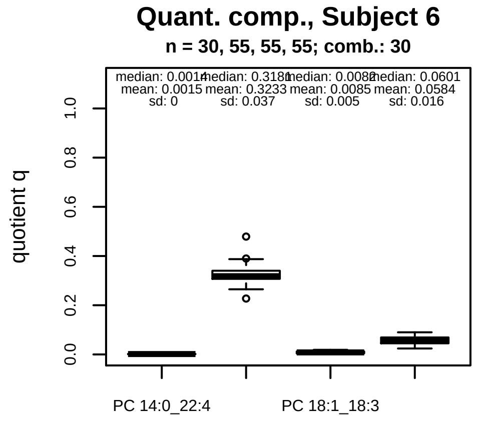

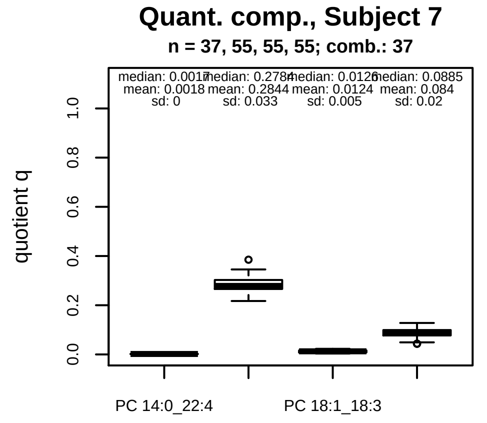

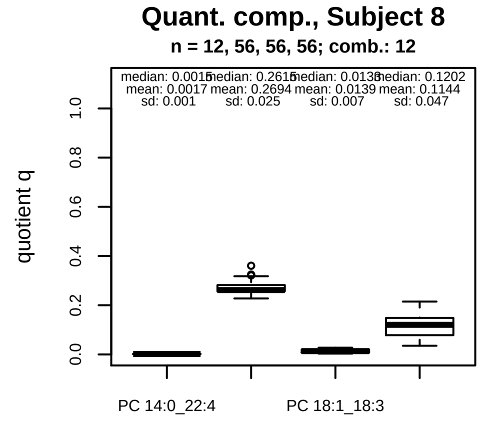

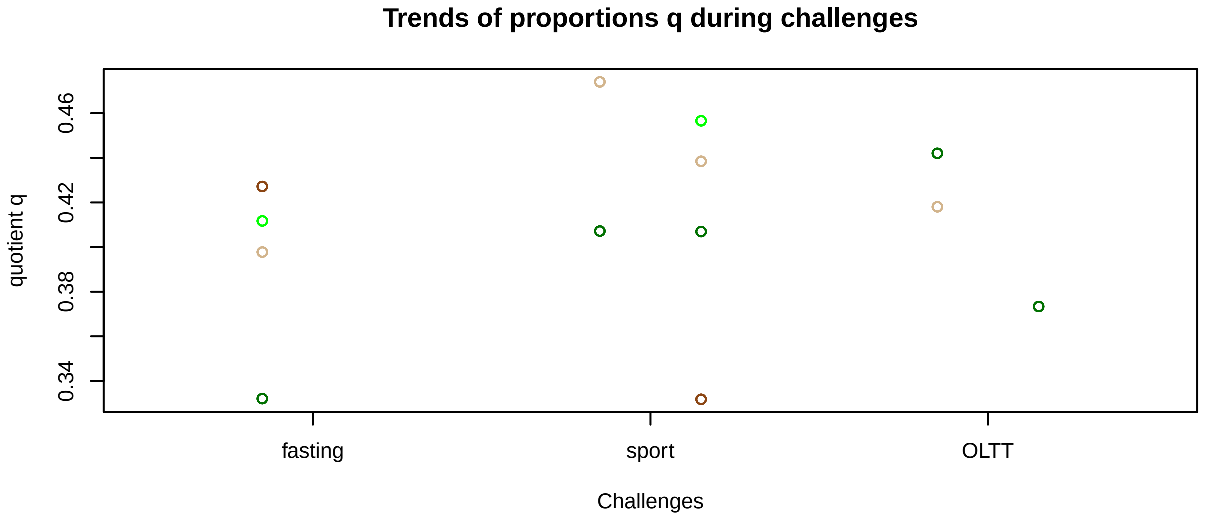

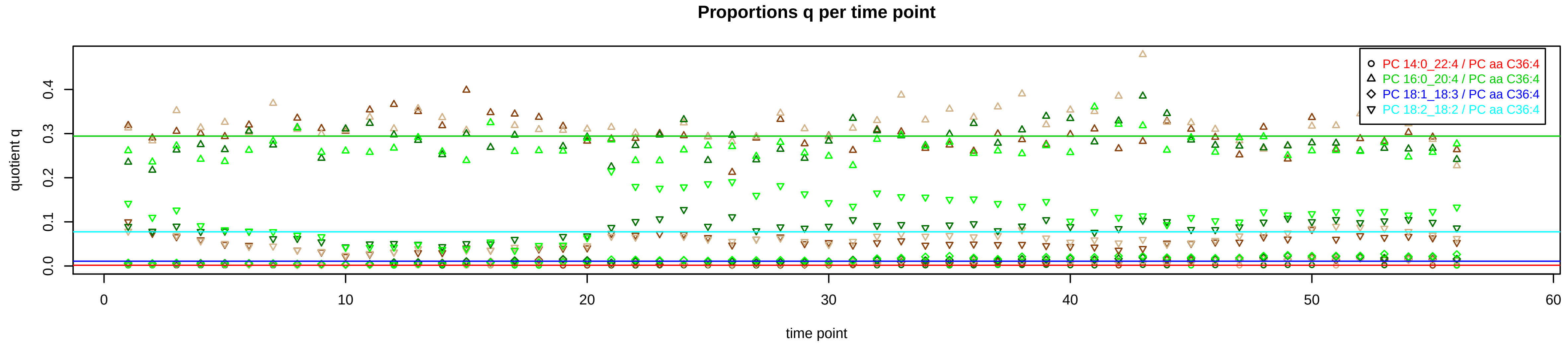

PC aa C36:5 = PC 14:0_22:5 + PC 16:0_20:5 + PC 18:2_18:3 + R

PC 16:0_20:5 excluded because of missingness > 75%

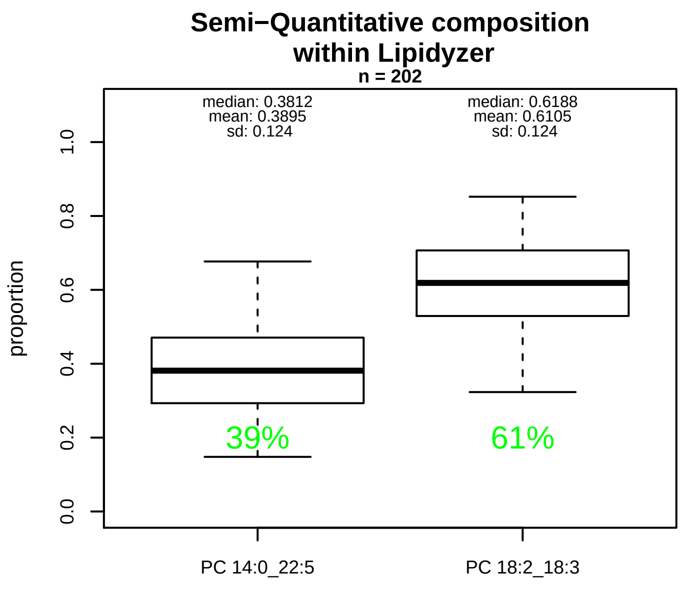

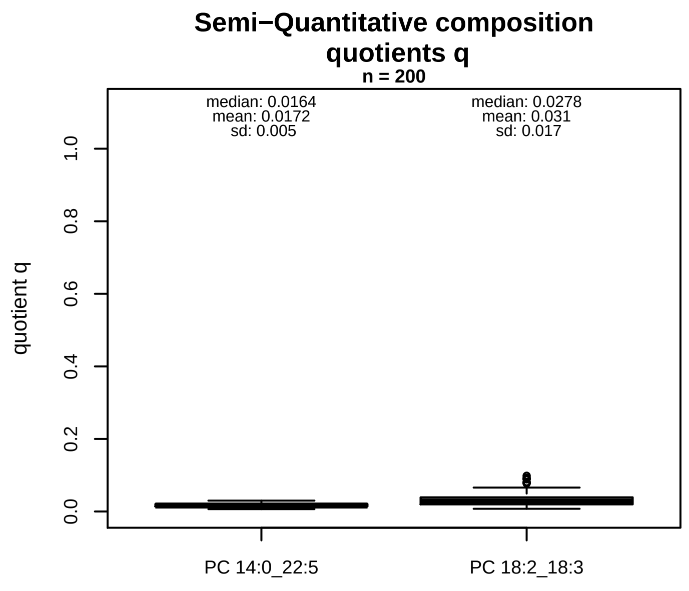

Semi−Quantitative composition: Lipidyzer

PC aa C36:5 consists of:
PC 14:0_22:5 39%
PC 18:2_18:3 61%
and of (not quantified):
PC 16:0_20:5, PC 14 1_22:4, PC 16 1_20:4, PC 18 1_18:4,
and further compounds

Composition: mean of proportions q

conc(PC aa C36:5) * 0.0172 = conc(PC 14:0_22:5) [var(q)=0.0172]
Percentiles: 5%−>0.0101, 25%−>0.0138, 75%−>0.0206, 95%−>0.0262
conc(PC aa C36:5) * 0.031 = conc(PC 18:2_18:3) [var(q)=0.031]
Percentiles: 5%−>0.0113, 25%−>0.0196, 75%−>0.0391, 95%−>0.0615

Linear model

```
PC aa C36:5 ~ b * ( PC 14:0_22:5
   + PC 18:2_18:3 )
```

b = 9.52537

R^2 = 0.17224

Ranges

Measure	AbsoluteIDQ	sum(Lipidyzer)	delta
Min	12.6	0.49	12.11
Max	50.5	2.12	48.38
Mean	27.44	1.24	26.2
Median	28.7	1.24	27.46
SD	9.37	0.38	8.99

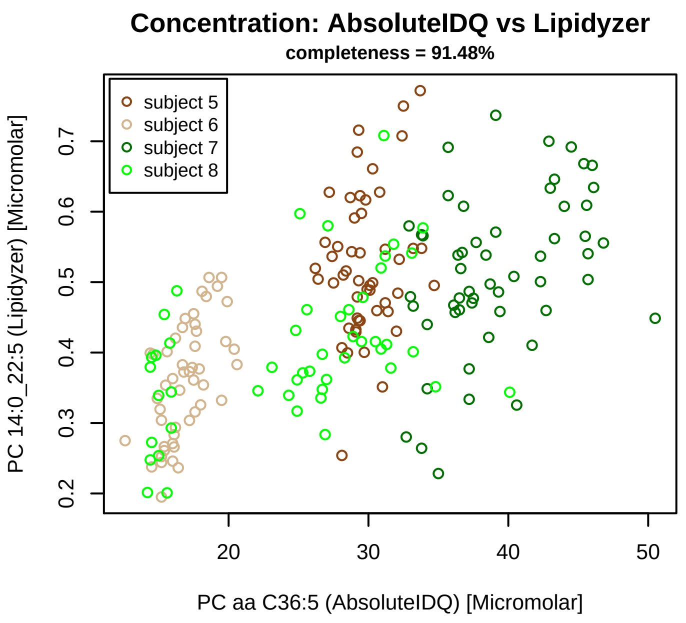

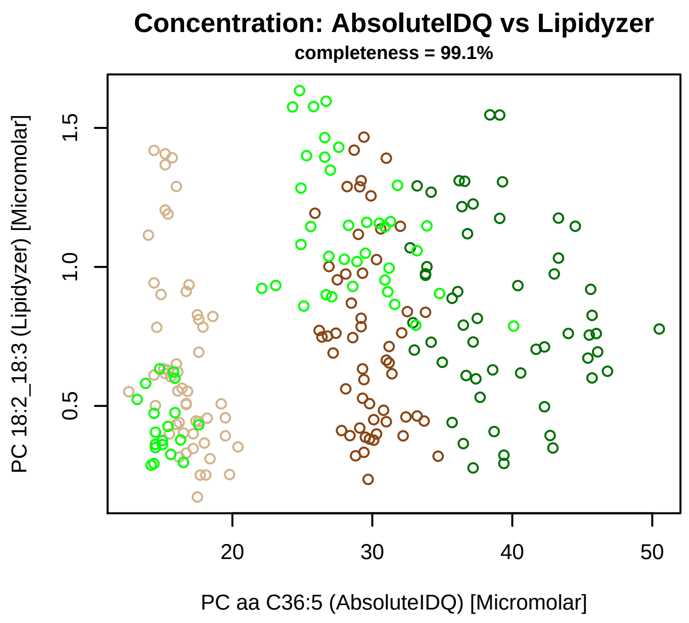

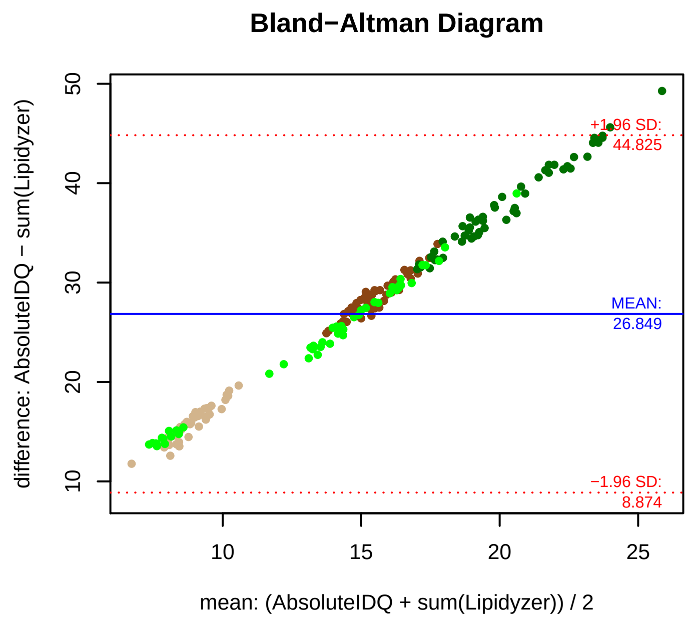

Stability of composition

(PC 14:0_22:5 + PC 18:2_18:3) / PC aa C36:5
Shapiro−Wilk Test of log quotients, pv: 0.49031; OK
SW: ok; ANOVA: Comp. ~ Subject_ID −> pv = 0.43298; EQUAL
Wilcoxon: Challenge; fasting: 1; sport: 0.125; OLTT: 0.5

PC 14:0_22:5 / PC aa C36:5
Shapiro−Wilk Test of log quotients, pv: 0.03493; OK
SW: ok; ANOVA: Comp. ~ Subject_ID −> pv = 2e−04; DIFFERENCE
Wilcoxon: Challenge; fasting: 1; sport: 0.625; OLTT: 1

PC 18:2_18:3 / PC aa C36:5
Shapiro−Wilk Test of log quotients, pv: 0.25945; OK
SW: ok; ANOVA: Comp. ~ Subject_ID −> pv = 0.0212; EQUAL
Wilcoxon: Challenge; fasting: 0.25; sport: 0.125; OLTT: 0.125

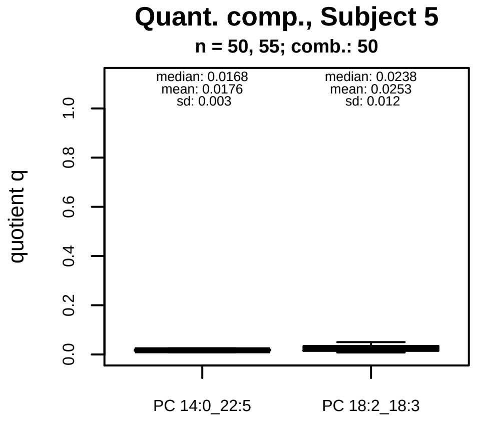

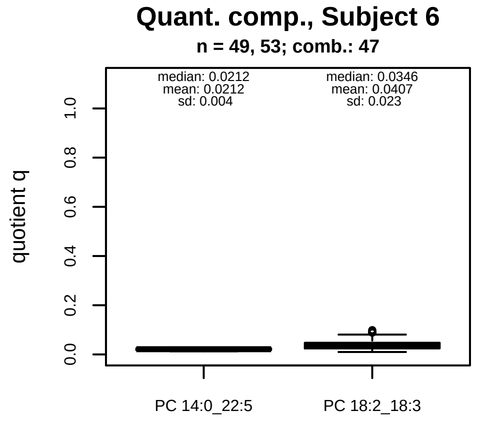

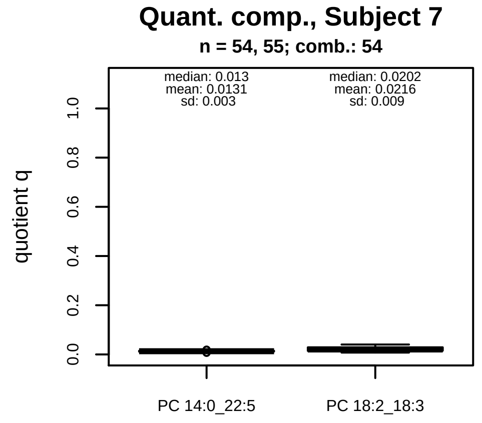

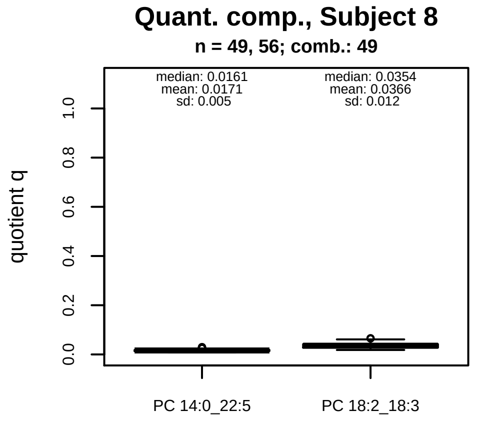

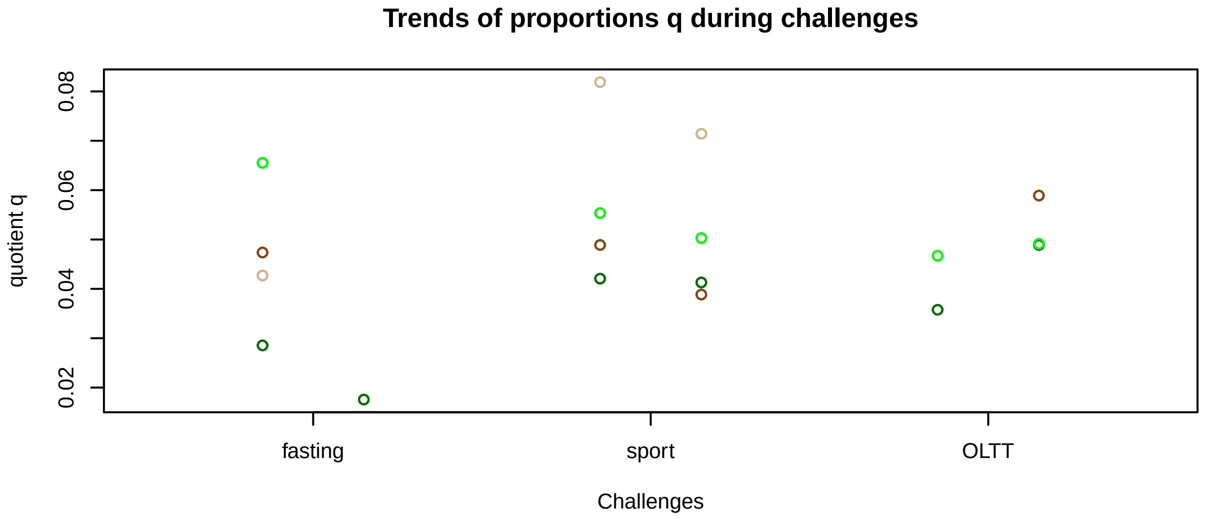

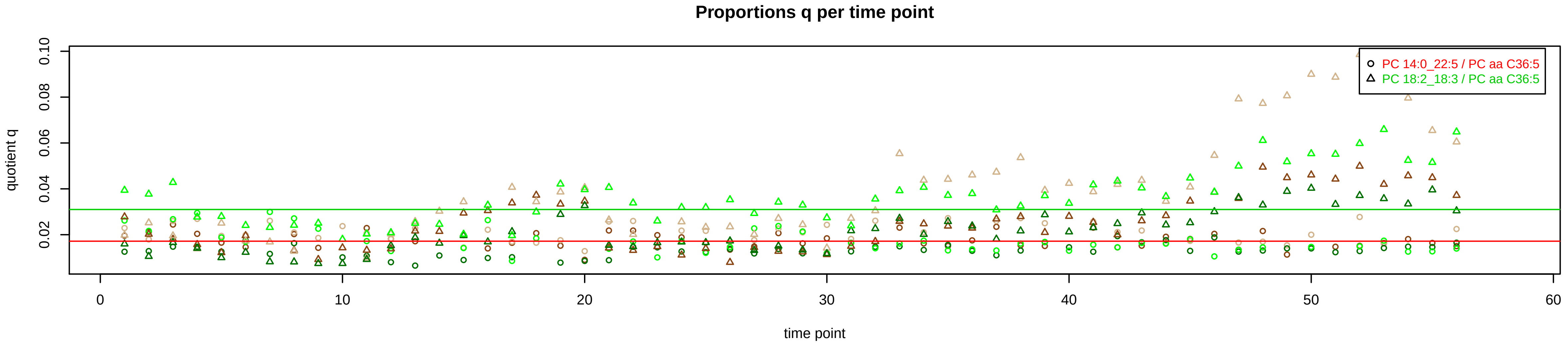

PC aa C36:6 = PC 14:0_22:6 + R

Semi−Quantitative composition within Lipidyzer

n = 199

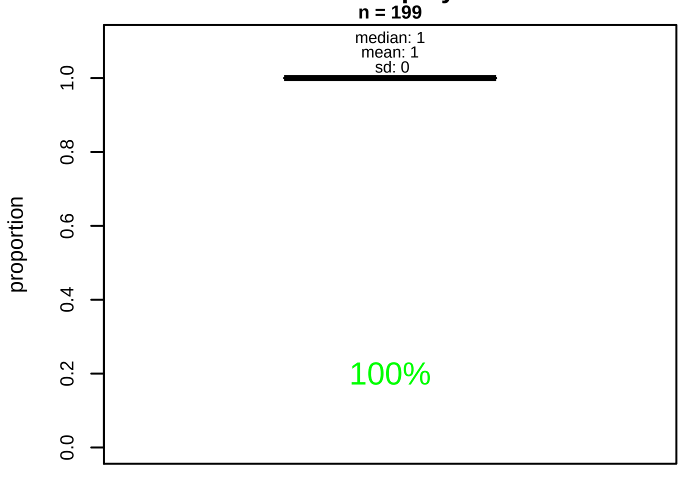

Semi−Quantitative composition quotients q

n = 197

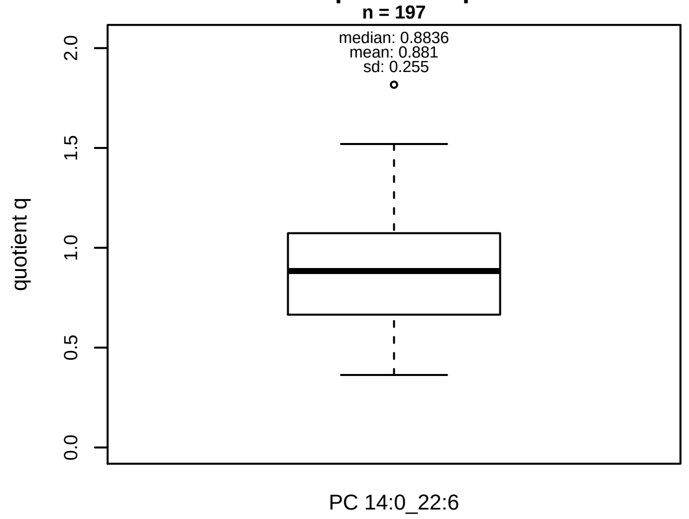

Semi−Quantitative composition: Lipidyzer

PC aa C36:6 consists of:
PC 14:0_22:6 100%
and of (not quantified):
PC 14:1_22:5, PC 16:1_20:5, PC 18:3_18:3, PC 18:4_18:2,
and further compounds

Composition: mean of proportions q

conc(PC aa C36:6) * 0.881 = conc(PC 14:0_22:6) [var(q)=0.881]
Percentiles: 5%->0.4947, 25%->0.6651, 75%->1.0732, 95%->1.2925

Linear model

```
PC aa C36:6 ~ b * ( PC 14:0_22:6 )
```

b = 0.74555

R^2 = 0.32274

Ranges

Measure	AbsoluteIDQ	sum(Lipidyzer)	delta
Min	0.39	0.27	0.12
Max	1.69	1.67	0.02
Mean	0.91	0.79	0.13
Median	0.9	0.76	0.14
SD	0.33	0.26	0.08

Concentration: AbsoluteIDQ vs Lipidyzer

completeness = 89.24%

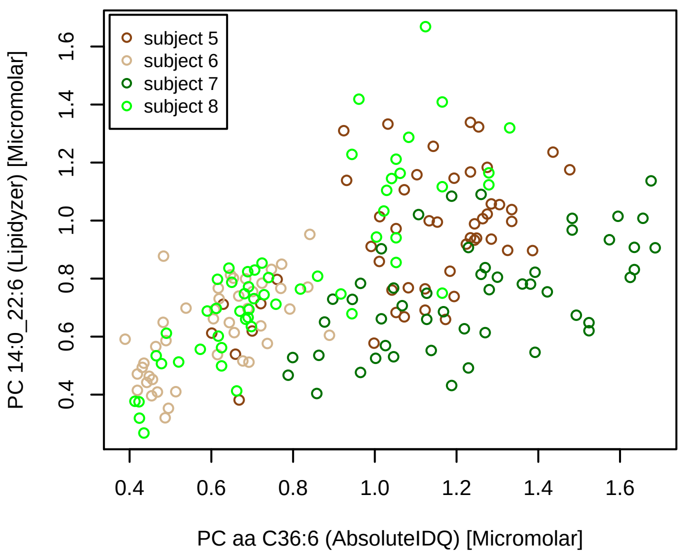

Bland−Altman Diagram

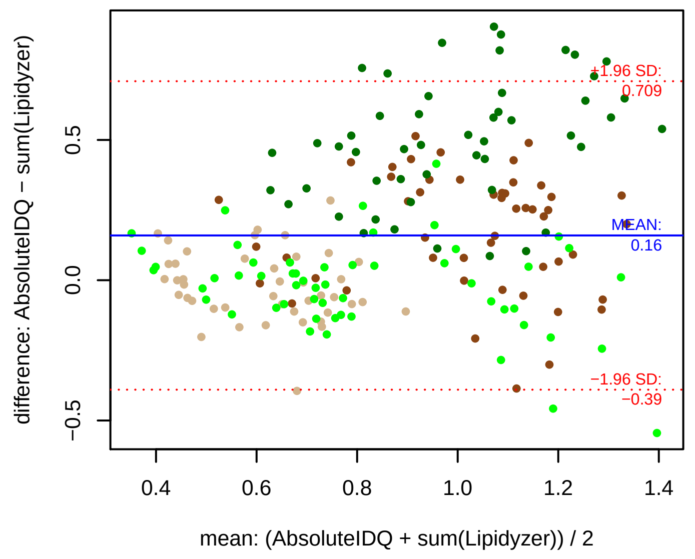

Stability of composition

PC 14:0_22:6 / PC aa C36:6
Shapiro−Wilk Test of log quotients, pv: 0.00996; NO
SW: ok; ANOVA: Comp. ~ Subject_ID −> pv = 0.68751; EQUAL
Wilcoxon: Challenge; fasting: 0.25; sport: 0.625; OLTT: 0.25

Quant. comp., Subject 5

n = 50; comb.: 50

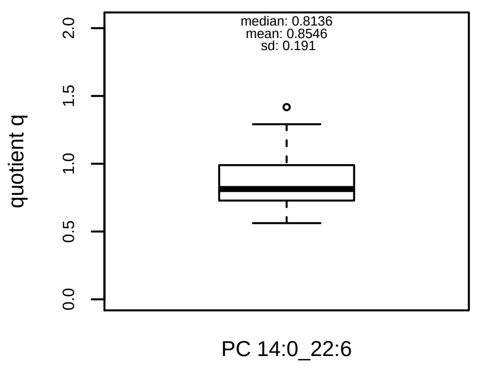

Quant. comp., Subject 6

n = 43; comb.: 43

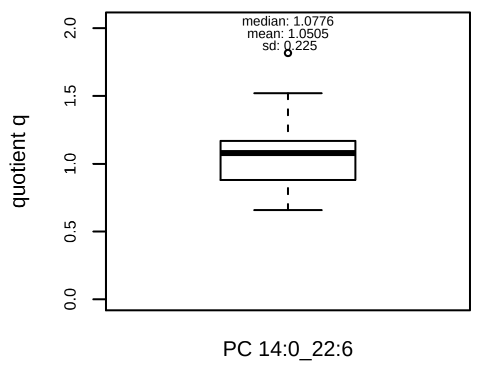

Quant. comp., Subject 7

n = 50; comb.: 50

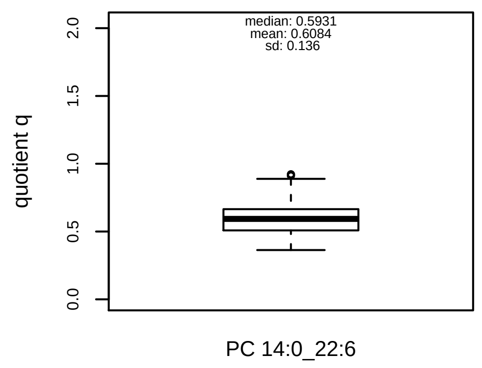

Quant. comp., Subject 8

n = 54; comb.: 54

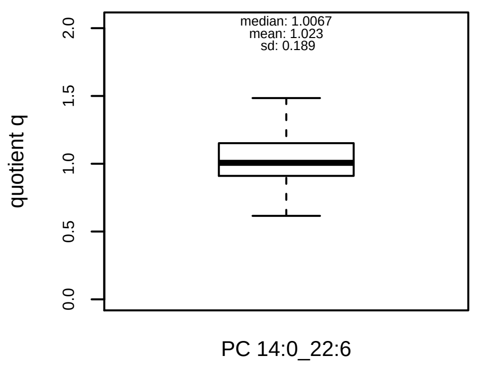

Trends of proportions q during challenges

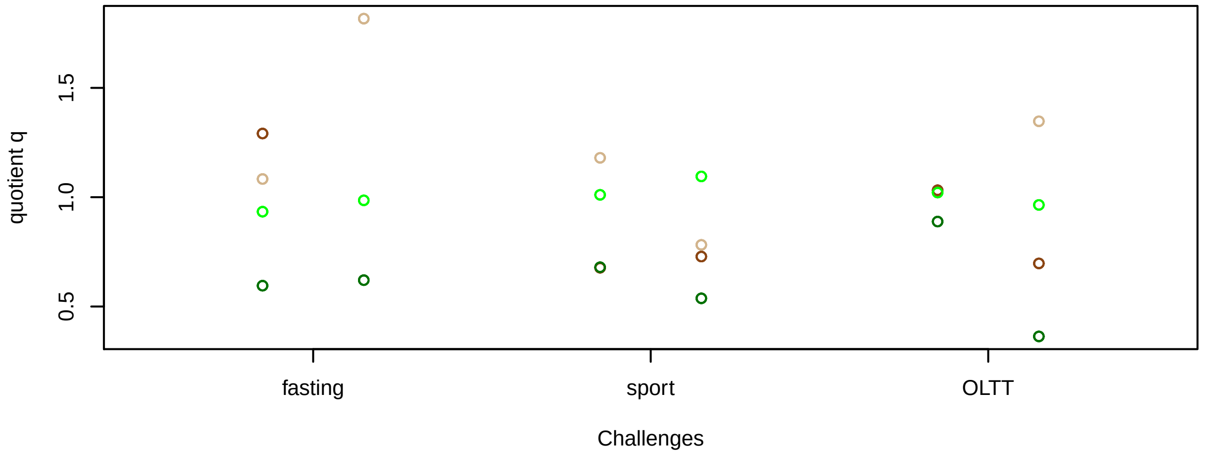

Proportions q per time point

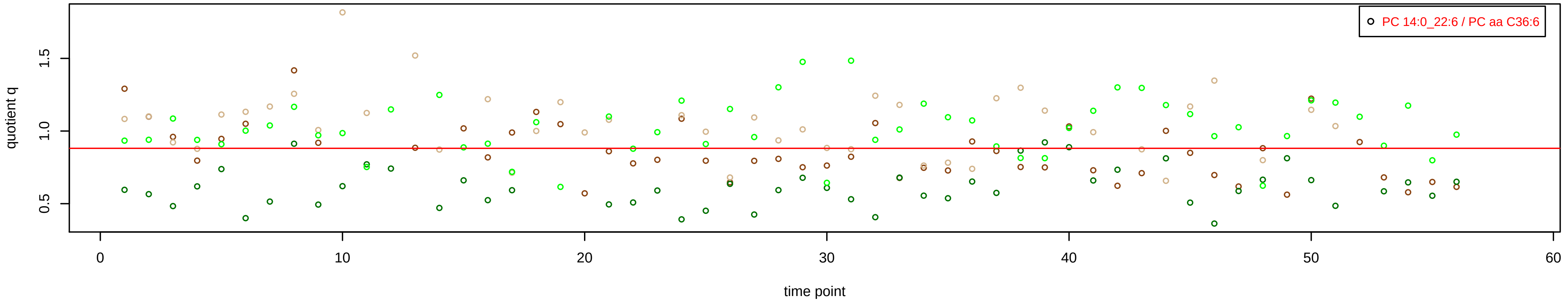

PC aa C38:0 = PC 18:0_20:0 + R

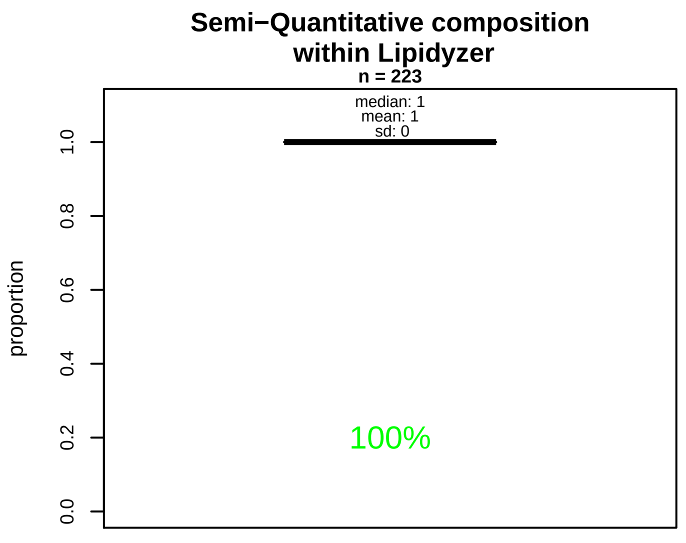

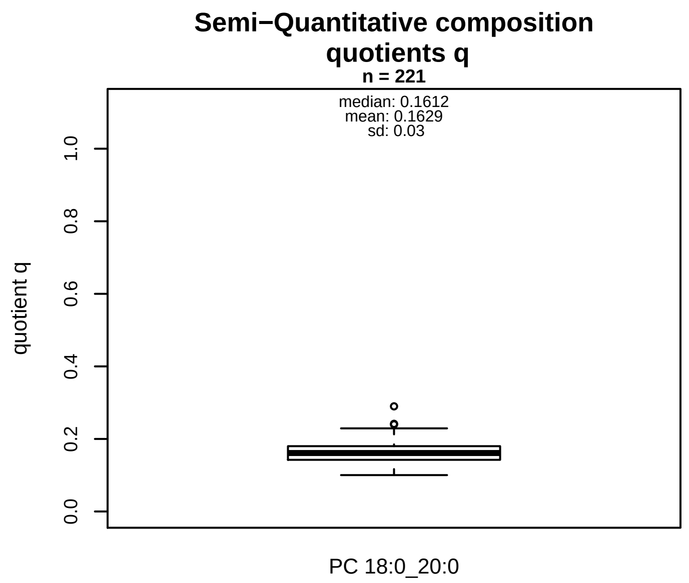

Semi−Quantitative composition: Lipidyzer

PC aa C38:0 consists of:
PC 18:0_20:0 100%
and of (not quantified):
PC 12:0_26:0, PC 13:0_25:0, PC 14:0_24:0, PC 16:0_22:0,
PC 17:0_21:0, PC 17:1_22:6, PC 19:0_19:0, PC O−17:0_22:(
PC O−18:0_21:0, PC O−20:0_19:0, PC O−18:1_22:6, SM42.(
and further compounds

Composition: mean of proportions q

conc(PC aa C38:0) * 0.1629 = conc(PC 18:0_20:0) [var(q)=0.1629]
Percentiles: 5%−>0.1193, 25%−>0.1424, 75%−>0.18, 95%−>0.2153

Linear model

```
PC aa C38:0 ~ b * ( PC 18:0_20:0 )
```

b = 3.58022

R^2 = 0.34478

Ranges

Measure	AbsoluteIDQ	sum(Lipidyzer)	delta
Min	2.33	0.35	1.98
Max	5.48	0.92	4.56
Mean	3.74	0.6	3.14
Median	3.66	0.59	3.07
SD	0.71	0.12	0.6

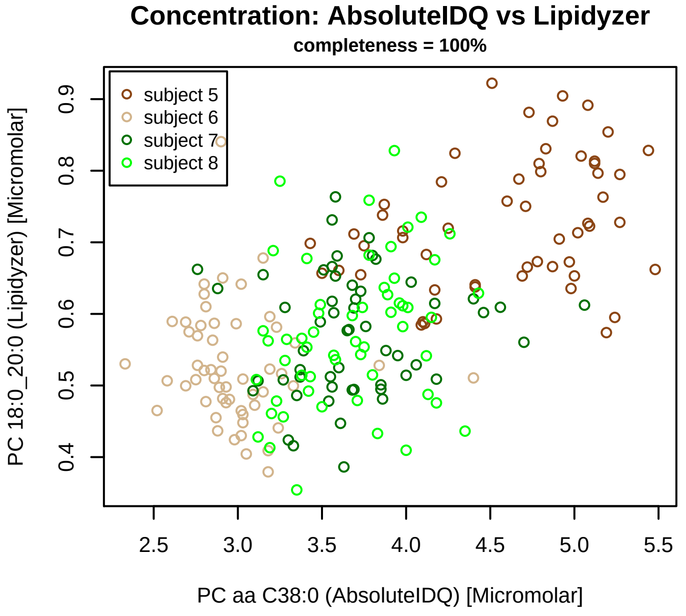

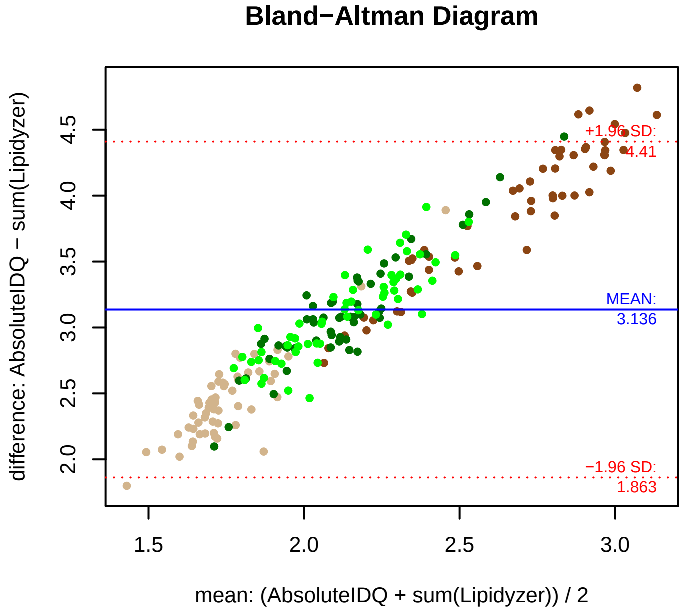

Stability of composition

PC 18:0_20:0 / PC aa C38:0
Shapiro−Wilk Test of log quotients, pv: 0.94774; OK
SW: ok; ANOVA: Comp. ~ Subject_ID −> pv = 0.05256; EQUAL
Wilcoxon: Challenge; fasting: 0.625; sport: 0.25; OLTT: 1

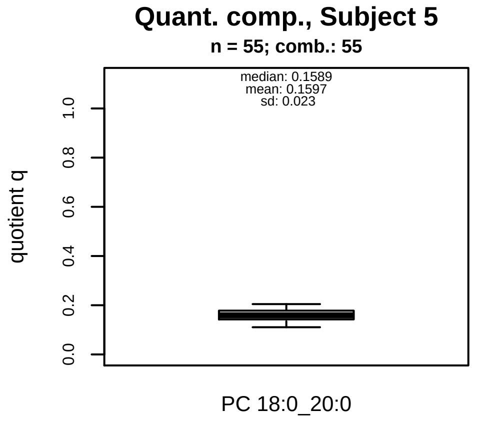

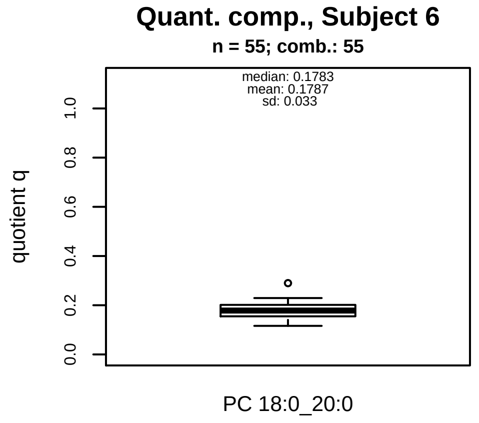

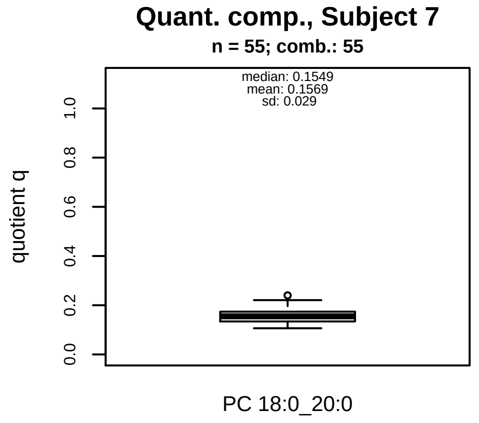

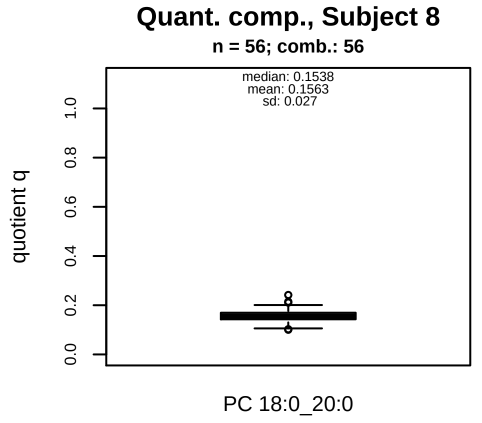

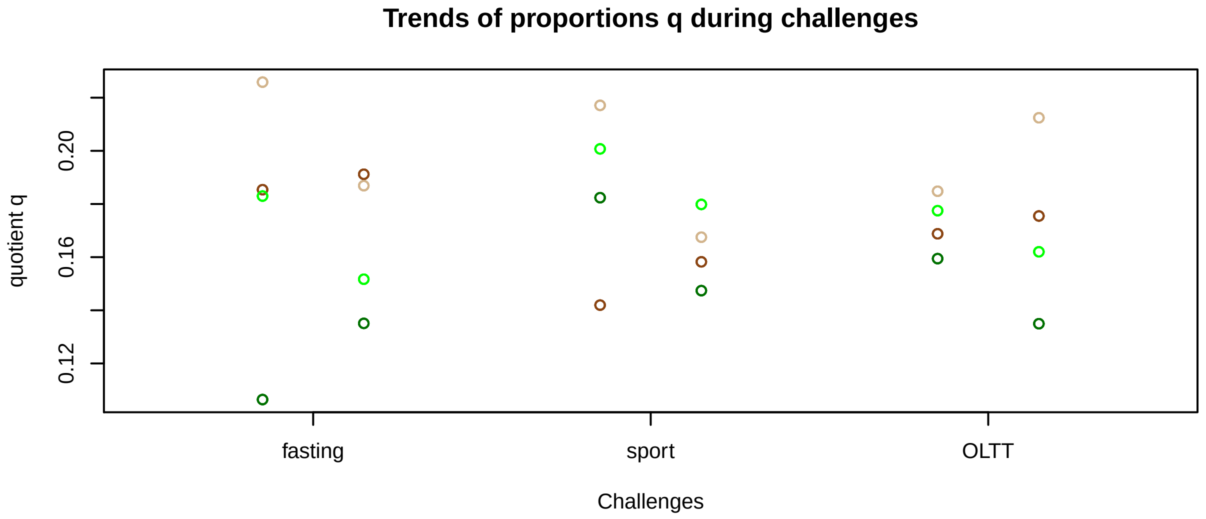

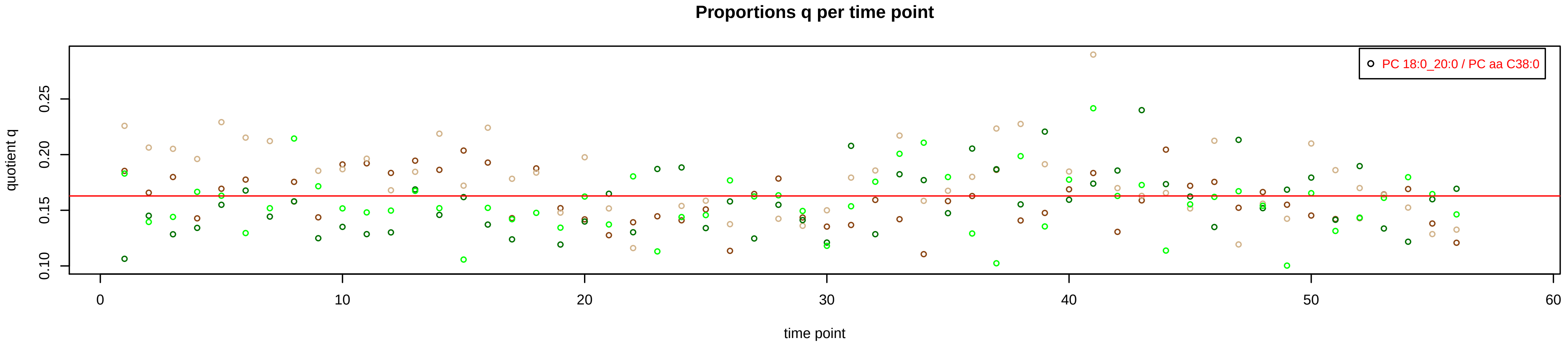

PC aa C38:3 = PC 18:0_20:3 + PC 18:1_20:2 + PC 18:2_20:1 + PC 20:0_18:3 + R

PC 20:0_18:3 excluded because of missingness > 75%

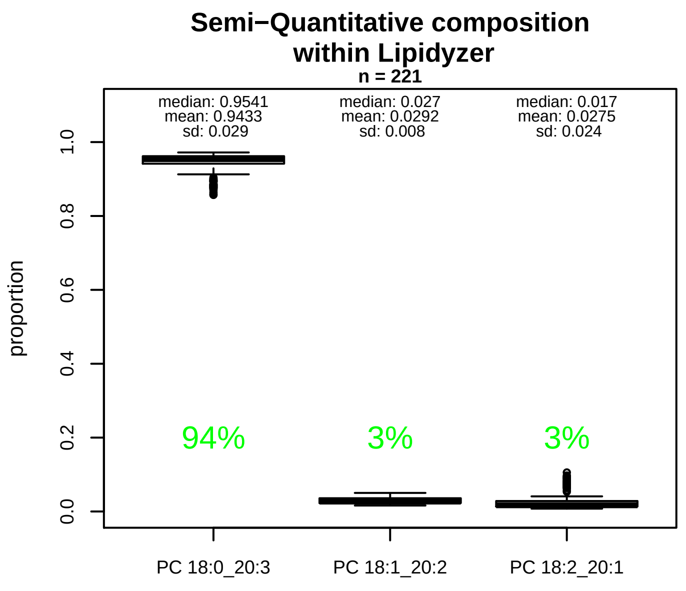

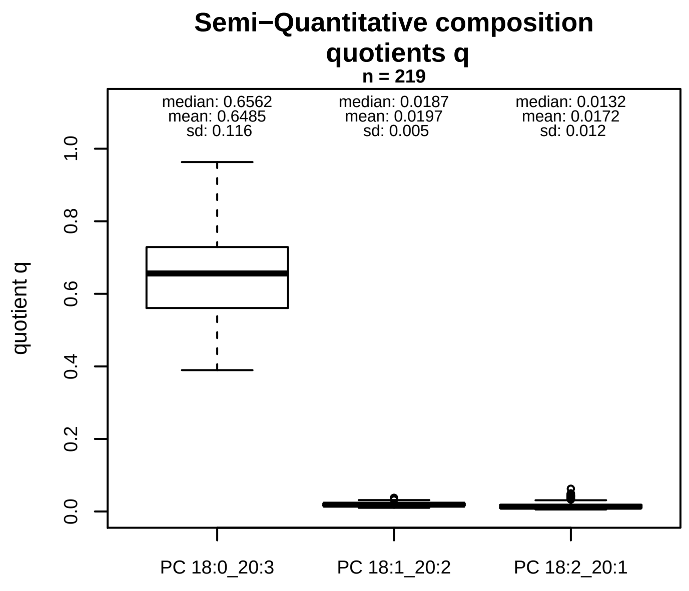

Semi−Quantitative composition: Lipidyzer

PC aa C38:3 consists of:
PC 18:0_20:3 94.3%
PC 18:1_20:2 2.9%
PC 18:2_20:1 2.7%
and of (not quantified):
PC 20:0_18:3, PC 16:1_22:2, PC 18:3_20:0, [13C1]SM 42:3, and further compounds

Linear model

```
PC aa C38:3 ~ b * ( PC 18:0_20:3
  + PC 18:1_20:2
  + PC 18:2_20:1 )
```

b = 0.98751

R^2 = 0.80663

Ranges

Measure	AbsoluteIDQ	sum(Lipidyzer)	delta
Min	25.7	13.72	11.98
Max	66.8	54.35	12.45
Mean	44.46	30.82	13.64
Median	42.4	30.43	11.97
SD	11.16	10.15	1.01

Composition: mean of proportions q

conc(PC aa C38:3) * 0.6485 = conc(PC 18:0_20:3) [var(q)=0.6485]
Percentiles: 5%−>0.4476, 25%−>0.5606, 75%−>0.7287, 95%−>0.8496
conc(PC aa C38:3) * 0.0197 = conc(PC 18:1_20:2) [var(q)=0.0197]
Percentiles: 5%−>0.0126, 25%−>0.0161, 75%−>0.0224, 95%−>0.0299
conc(PC aa C38:3) * 0.0172 = conc(PC 18:2_20:1) [var(q)=0.0172]
Percentiles: 5%−>0.007, 25%−>0.0096, 75%−>0.0184, 95%−>0.0436

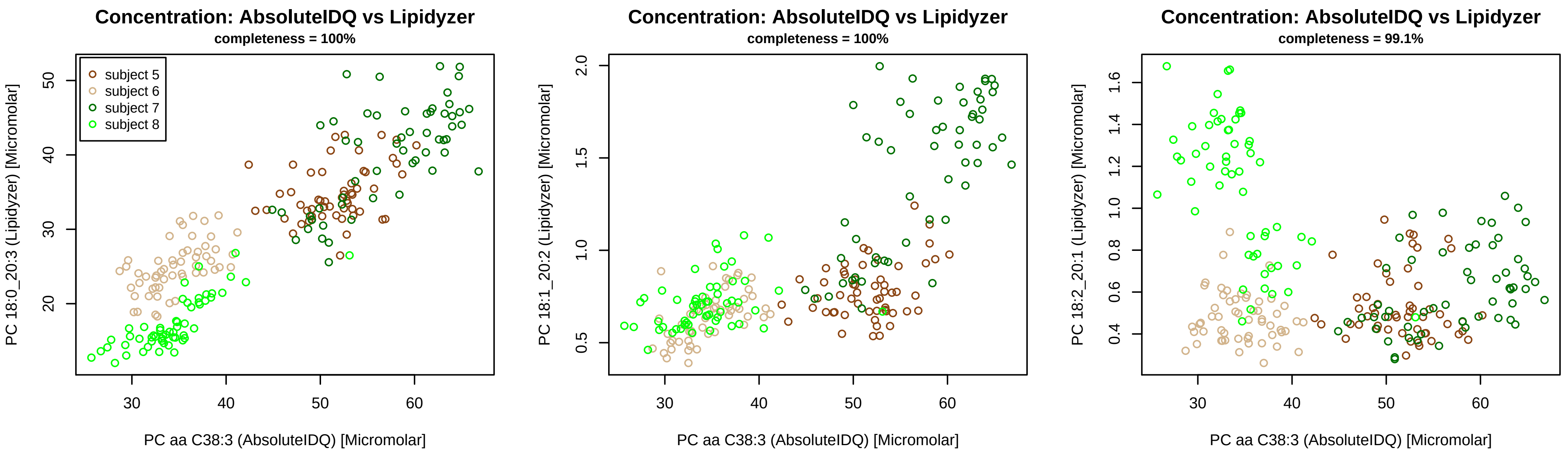

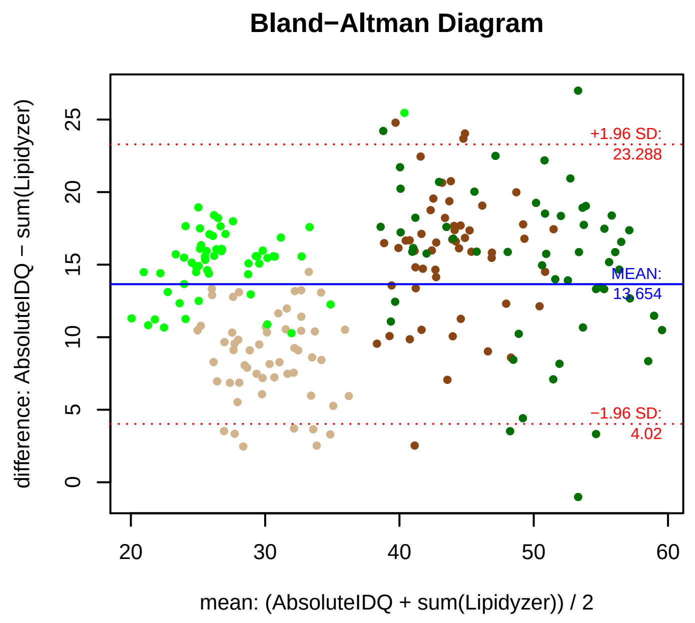

Stability of composition

(PC 18:0_20:3 + PC 18:1_20:2 + PC 18:2_20:1) / PC aa C38:3
Shapiro−Wilk Test of log quotients, pv: 0.06972; OK
SW: ok; ANOVA: Comp. ~ Subject_ID −> pv = 0; DIFFERENCE
Wilcoxon: Challenge; fasting: 0.5; sport: 0.125; OLTT: 0.25

PC 18:1_20:2 / PC aa C38:3
Shapiro−Wilk Test of log quotients, pv: 0.11436; OK
SW: ok; ANOVA: Comp. ~ Subject_ID −> pv = 0; DIFFERENCE
Wilcoxon: Challenge; fasting: 0.125; sport: 1; OLTT: 0.375

PC 18:0_20:3 / PC aa C38:3
Shapiro−Wilk Test of log quotients, pv: 2e−04; NO
SW: ok; ANOVA: Comp. ~ Subject_ID −> pv = 0; DIFFERENCE
Wilcoxon: Challenge; fasting: 0.25; sport: 0.125; OLTT: 0.25

PC 18:2_20:1 / PC aa C38:3
Shapiro−Wilk Test of log quotients, pv: 0; NO
SW: no; Kruskal: Comp. ~ Subject_ID −> pv = 0; DIFFERENCE
Wilcoxon: Challenge; fasting: 0.5; sport: 0.25; OLTT: 0.875

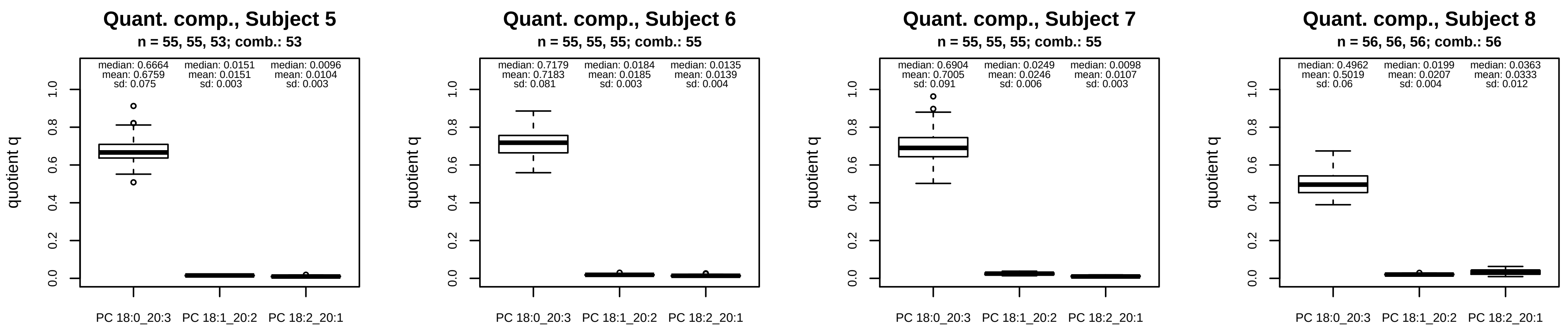

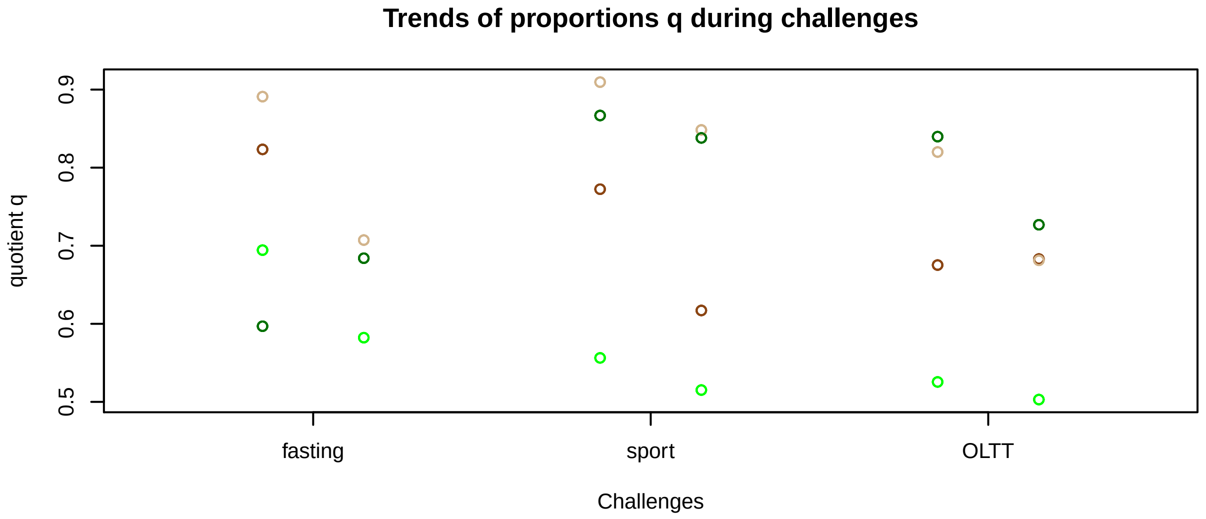

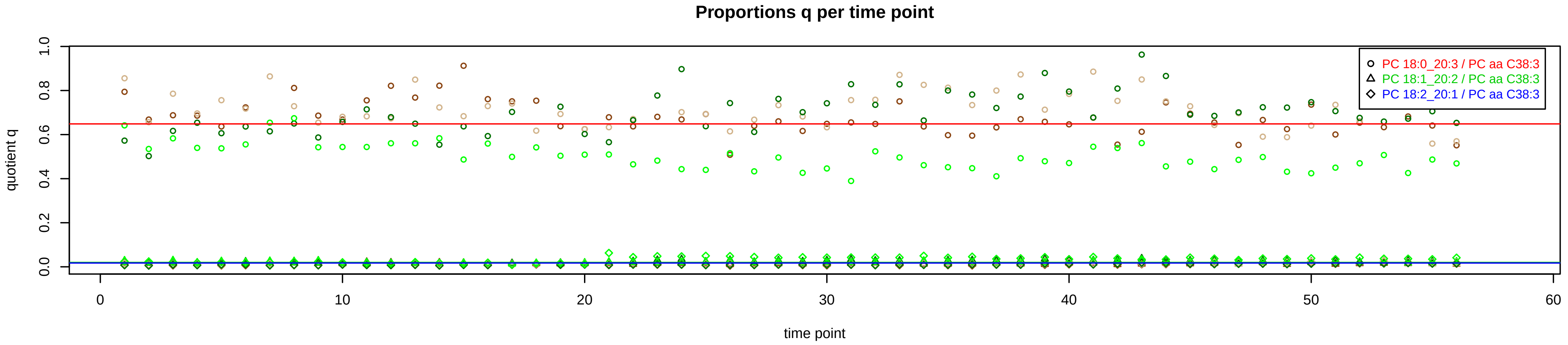

PC aa C38:4 = PC 16:0_22:4 + PC 18:0_20:4 + PC 18:1_20:3 + PC 18:2_20:2 + R

Semi−Quantitative composition within Lipidyzer

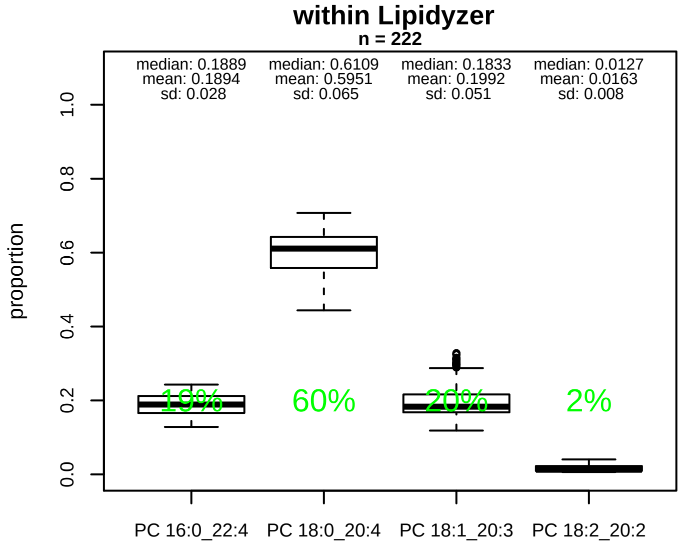

Semi−Quantitative composition quotients q

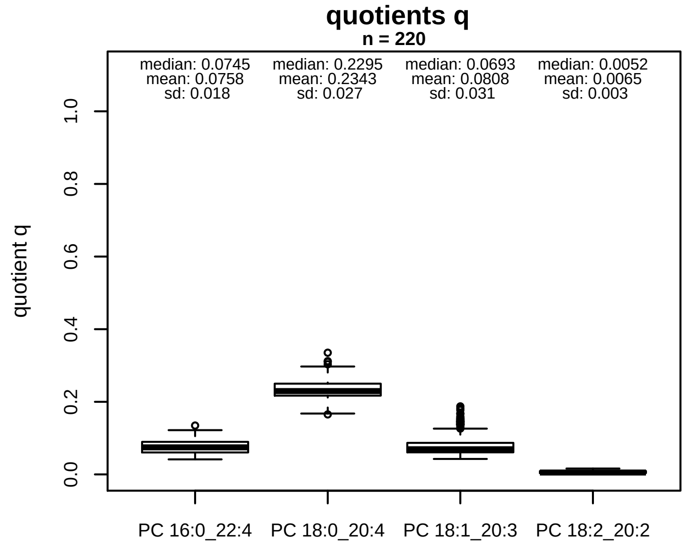

Semi−Quantitative composition: Lipidyzer

PC aa C38:4 consists of:
PC 16:0_22:4 18.9%
PC 18:0_20:4 59.5%
PC 18:1_20:3 19.9%
PC 18:2_20:2 1.6%
and of (not quantified):
PC 18:4_20:0
and further compounds

Linear model

```
PC aa C38:4 ~ b * ( PC 16:0_22:4
  + PC 18:0_20:4
  + PC 18:1_20:3
  + PC 18:2_20:2 )
```

b = 1.99845

R^2 = 0.58796

Ranges

Measure	AbsoluteIDQ	sum(Lipidyzer)	delta
Min	54	19.61	34.39
Max	153	61.31	91.69
Mean	99.43	39.14	60.29
Median	95.5	40.09	55.41
SD	21.26	8.1	13.16

Composition: mean of proportions q

conc(PC aa C38:4) * 0.0758 = conc(PC 16:0_22:4) [var(q)=0.0758]
Percentiles: 5%−>0.0504, 25%−>0.0605, 75%−>0.0897, 95%−>0.1059
conc(PC aa C38:4) * 0.2343 = conc(PC 18:0_20:4) [var(q)=0.2343]
Percentiles: 5%−>0.1971, 25%−>0.217, 75%−>0.2499, 95%−>0.2867
conc(PC aa C38:4) * 0.0808 = conc(PC 18:1_20:3) [var(q)=0.0808]
Percentiles: 5%−>0.049, 25%−>0.0608, 75%−>0.0869, 95%−>0.15
conc(PC aa C38:4) * 0.0065 = conc(PC 18:2_20:2) [var(q)=0.0065]
Percentiles: 5%−>0.0028, 25%−>0.0037, 75%−>0.0094, 95%−>0.0126

Concentration: AbsoluteIDQ vs Lipidyzer

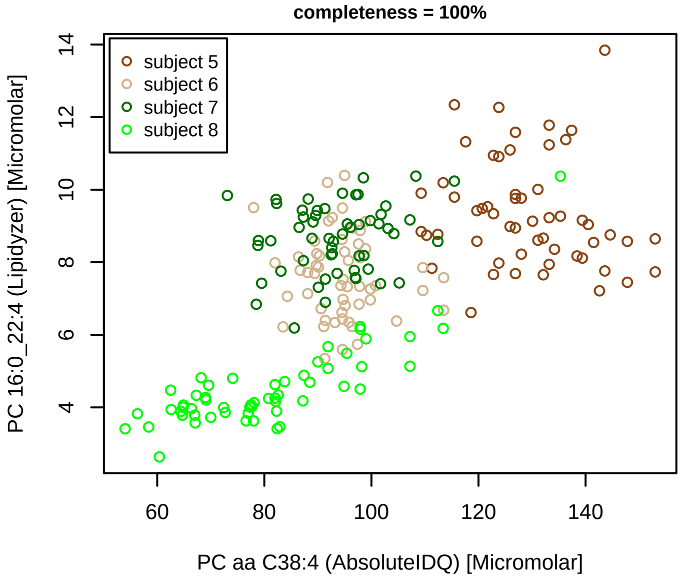

Concentration: AbsoluteIDQ vs Lipidyzer

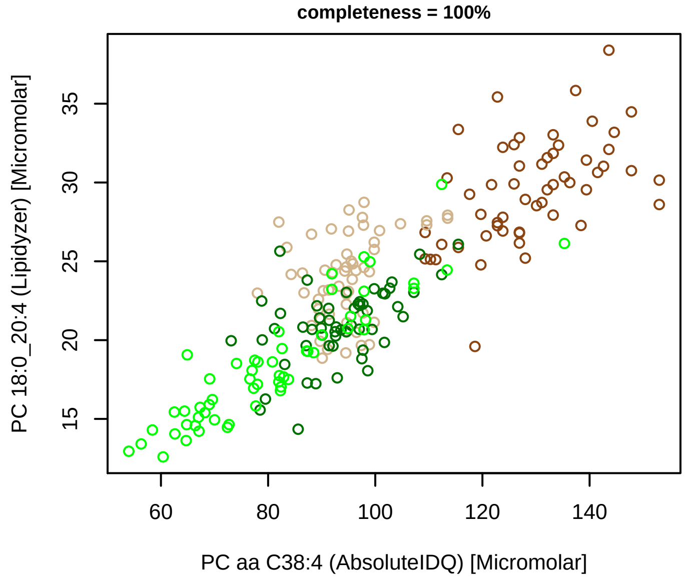

Concentration: AbsoluteIDQ vs Lipidyzer

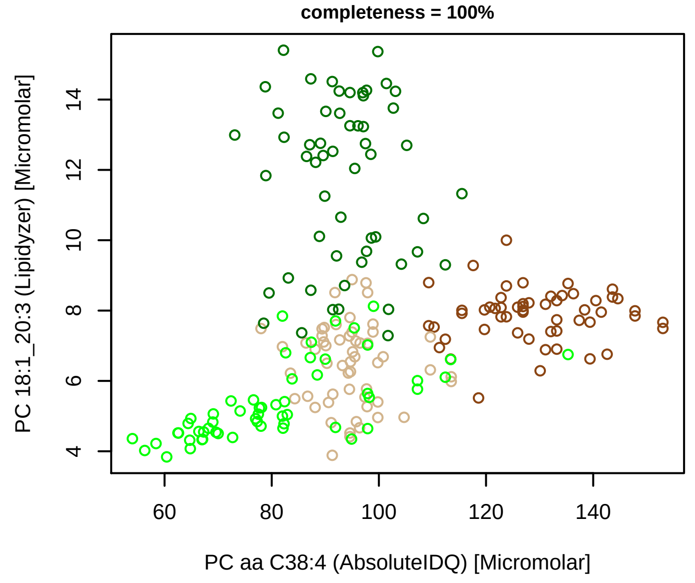

Concentration: AbsoluteIDQ vs Lipidyzer

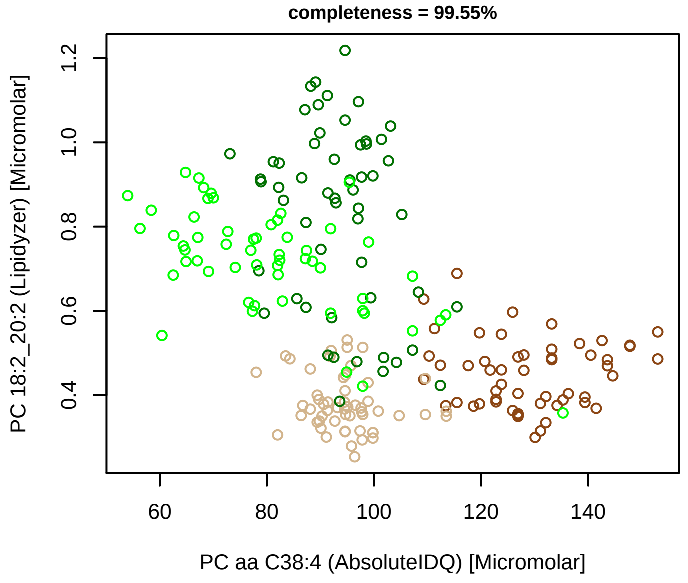

Bland−Altman Diagram

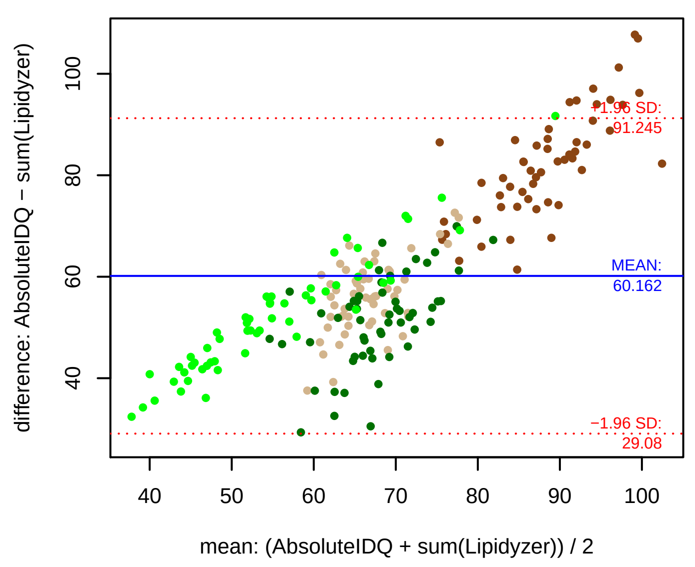

Stability of composition

(PC 16:0_22:4 + PC 18:0_20:4 + PC 18:1_20:3 + PC 18:2_20:2) / PC aa C38:4
Shapiro−Wilk Test of log quotients, pv: 0.00246; NO
SW: ok; ANOVA: Comp. ~ Subject_ID −> pv = 0.52376; EQUAL
Wilcoxon: Challenge; fasting: 0.25; sport: 0.125; OLTT: 0.625

PC 16:0_22:4 / PC aa C38:4
Shapiro−Wilk Test of log quotients, pv: 0.01057; OK
SW: ok; ANOVA: Comp. ~ Subject_ID −> pv = 2e−05; DIFFERENCE
Wilcoxon: Challenge; fasting: 0.625; sport: 0.875; OLTT: 0.375

PC 18:0_20:4 / PC aa C38:4
Shapiro−Wilk Test of log quotients, pv: 0.04492; OK
SW: ok; ANOVA: Comp. ~ Subject_ID −> pv = 0.05199; EQUAL
Wilcoxon: Challenge; fasting: 0.375; sport: 0.125; OLTT: 0.625

PC 18:1_20:3 / PC aa C38:4
Shapiro−Wilk Test of log quotients, pv: 0; NO
SW: ok; ANOVA: Comp. ~ Subject_ID −> pv = 2e−05; DIFFERENCE
Wilcoxon: Challenge; fasting: 0.125; sport: 0.25; OLTT: 0.125

PC 18:2_20:2 / PC aa C38:4
Shapiro−Wilk Test of log quotients, pv: 0; NO
SW: no; Kruskal: Comp. ~ Subject_ID −> pv = 0; DIFFERENCE
Wilcoxon: Challenge; fasting: 0.125; sport: 0.625; OLTT: 0.875

Quant. comp., Subject 5

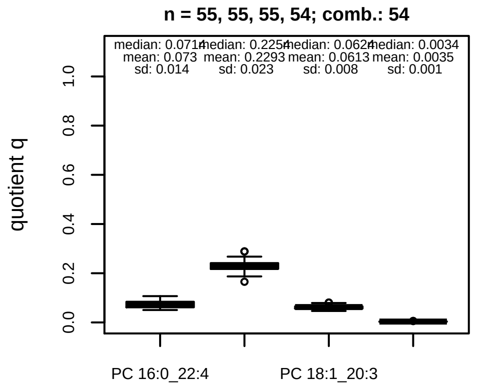

Quant. comp., Subject 6

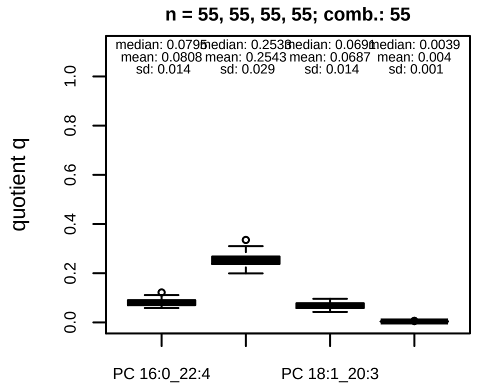

Quant. comp., Subject 7

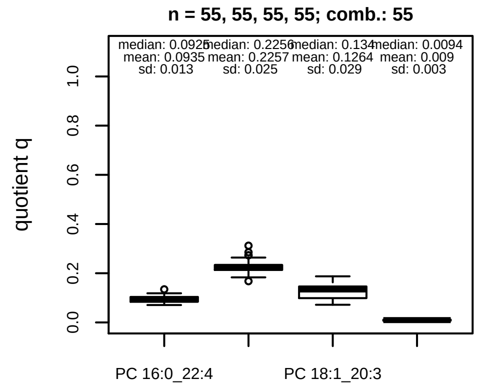

Quant. comp., Subject 8

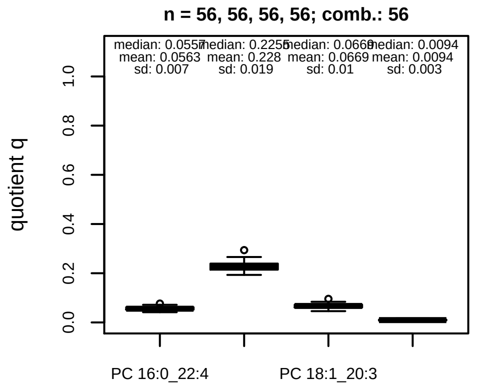

Trends of proportions q during challenges

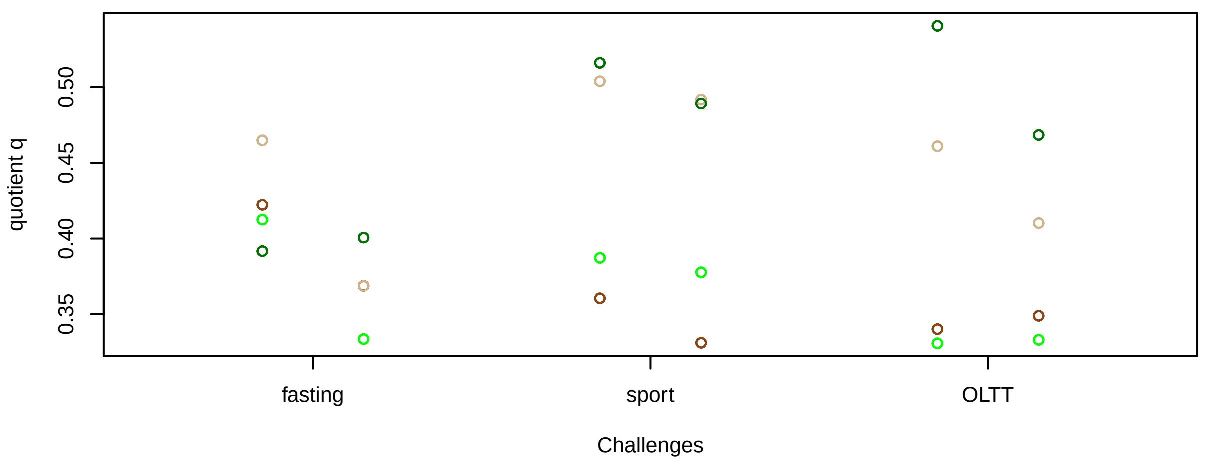

Proportions q per time point

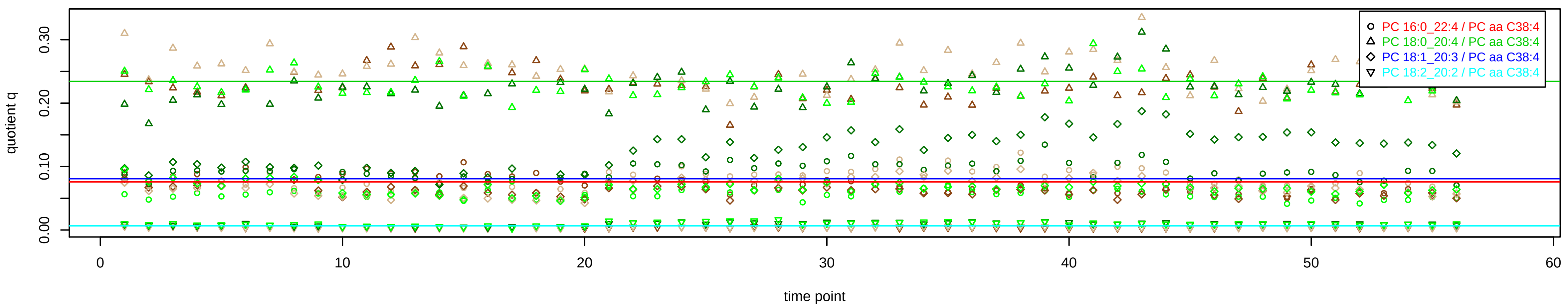

PC aa C38:5 = PC 16:0_22:5 + PC 18:0_20:5 + PC 18:1_20:4 + PC 18:2_20:3 + R

PC 18:0_20:5 excluded because of missingness > 75%

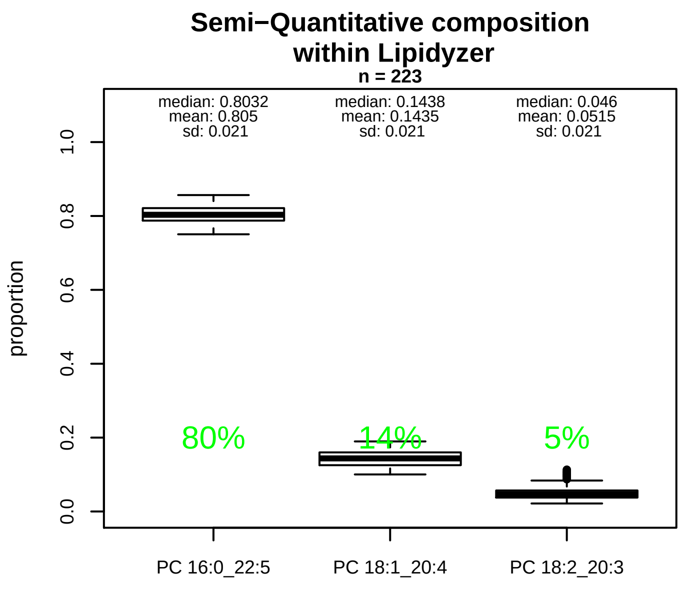

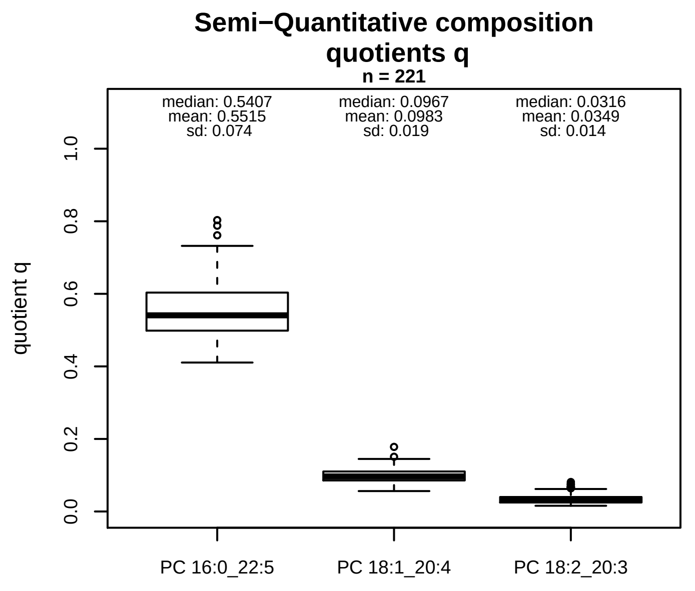

Semi−Quantitative composition: Lipidyzer

PC aa C38:5 consists of:
PC 16:0_22:5 80.5%
PC 18:1_20:4 14.4%
PC 18:2_20:3 5.1%
and of (not quantified):
PC 18:0_20:5, PC 16:1_22:4, PC 18:3_20:2, PC 18:4_20:1,
and further compounds

Linear model

```
PC aa C38:5 ~ b * ( PC 16:0_22:5
   + PC 18:1_20:4
   + PC 18:2_20:3 )
```

b = 1.21475

R^2 = 0.677

Ranges

Measure	AbsoluteIDQ	sum(Lipidyzer)	delta
Min	34.9	22.92	11.98
Max	84.4	63.92	20.48
Mean	57.86	39.37	18.49
Median	58	40.77	17.23
SD	11.88	8.02	3.85

Composition: mean of proportions q

conc(PC aa C38:5) * 0.5515 = conc(PC 16:0_22:5) [var(q)=0.5515]
Percentiles: 5%−>0.438, 25%−>0.4985, 75%−>0.6034, 95%−>0.6725
conc(PC aa C38:5) * 0.0983 = conc(PC 18:1_20:4) [var(q)=0.0983]
Percentiles: 5%−>0.0687, 25%−>0.0856, 75%−>0.1103, 95%−>0.1286
conc(PC aa C38:5) * 0.0349 = conc(PC 18:2_20:3) [var(q)=0.0349]
Percentiles: 5%−>0.0201, 25%−>0.0248, 75%−>0.0399, 95%−>0.0647

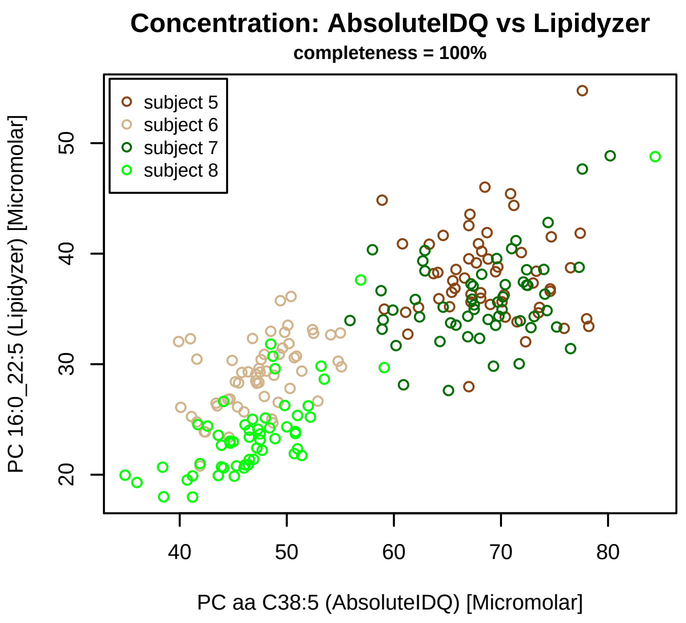

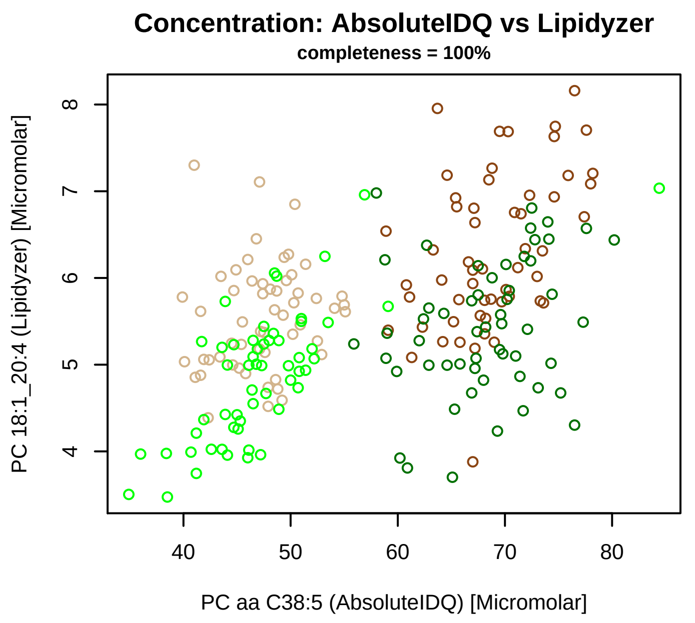

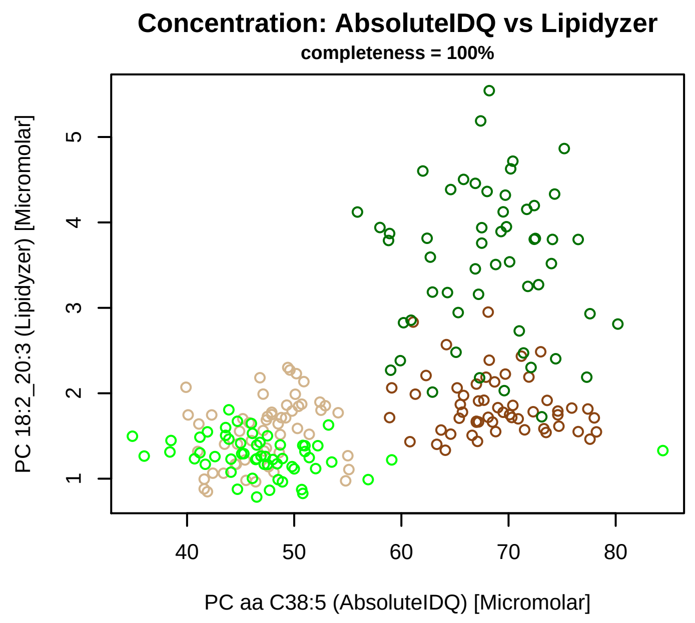

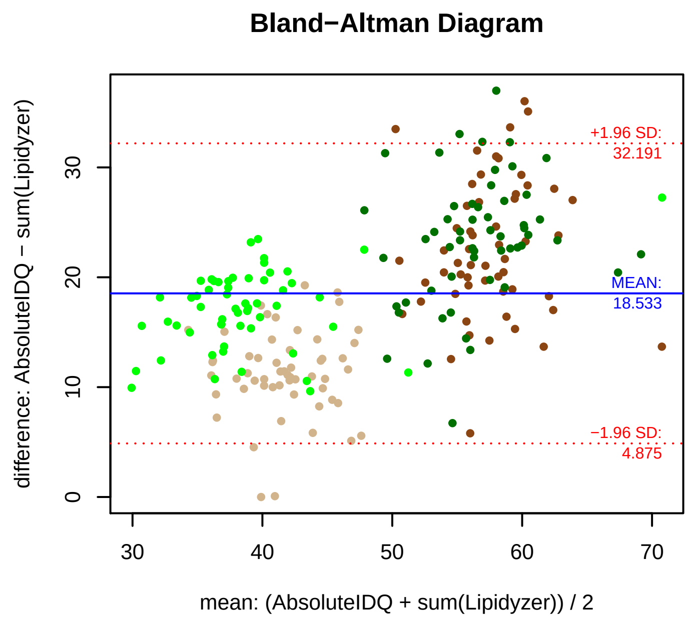

Stability of composition

(PC 16:0_22:5 + PC 18:1_20:4 + PC 18:2_20:3) / PC aa C38:5
Shapiro−Wilk Test of log quotients, pv: 0.37049; OK
SW: ok; ANOVA: Comp. ~ Subject_ID −> pv = 2e−05; DIFFERENCE
Wilcoxon: Challenge; fasting: 0.625; sport: 0.25; OLTT: 0.25

PC 16:0_22:5 / PC aa C38:5
Shapiro−Wilk Test of log quotients, pv: 0.26347; OK
SW: ok; ANOVA: Comp. ~ Subject_ID −> pv = 0; DIFFERENCE
Wilcoxon: Challenge; fasting: 0.875; sport: 0.25; OLTT: 0.25

PC 18:1_20:4 / PC aa C38:5
Shapiro−Wilk Test of log quotients, pv: 0.41624; OK
SW: ok; ANOVA: Comp. ~ Subject_ID −> pv = 0.82498; EQUAL
Wilcoxon: Challenge; fasting: 0.375; sport: 0.25; OLTT: 0.375

PC 18:2_20:3 / PC aa C38:5
Shapiro−Wilk Test of log quotients, pv: 0.00026; NO
SW: ok; ANOVA: Comp. ~ Subject_ID −> pv = 0.03257; EQUAL
Wilcoxon: Challenge; fasting: 0.125; sport: 0.125; OLTT: 0.125

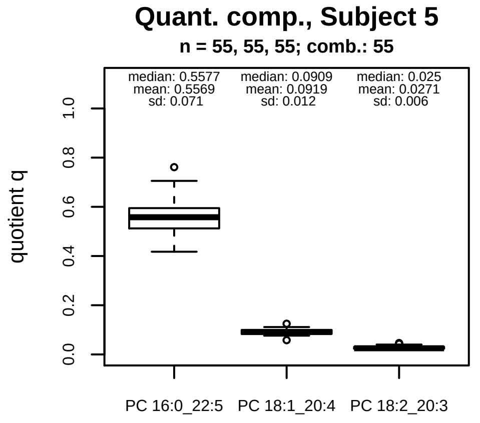

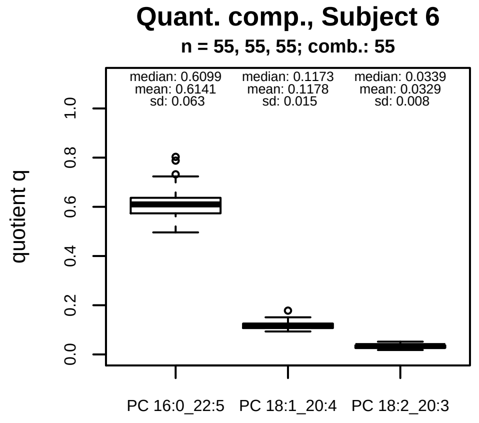

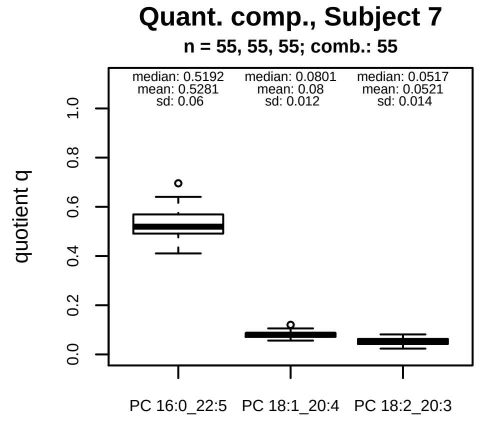

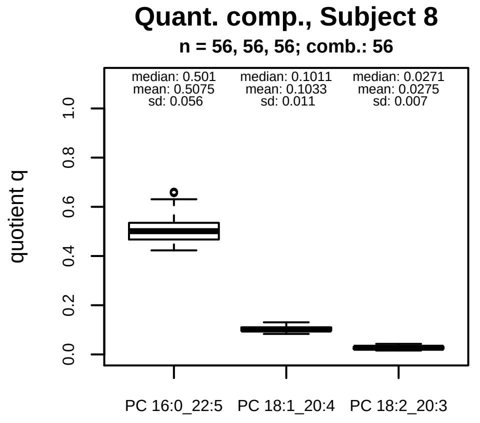

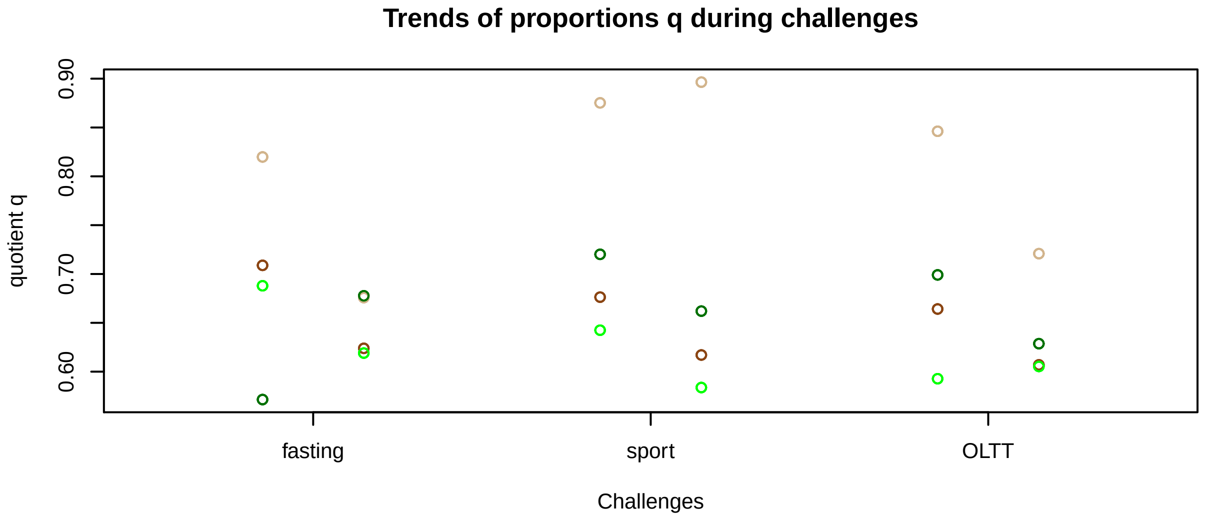

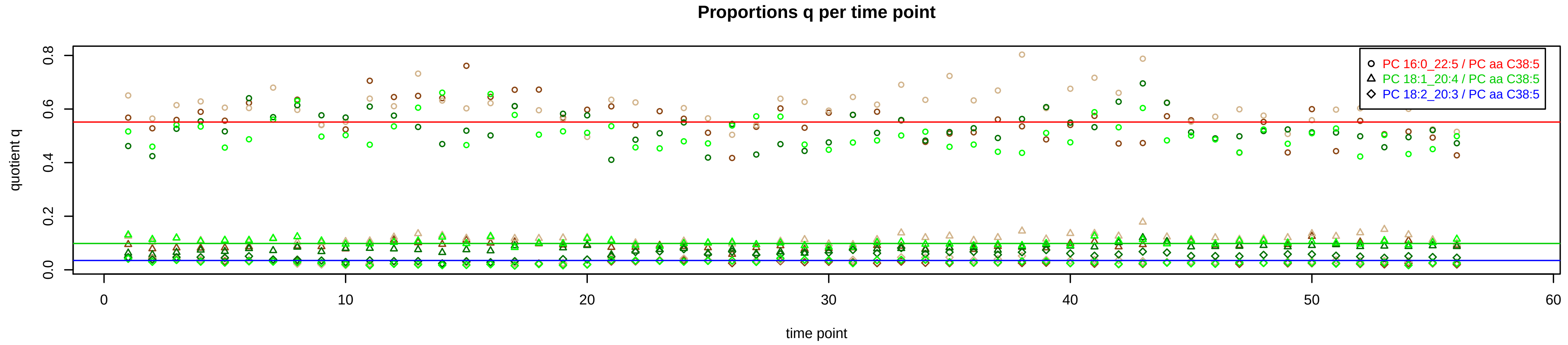

PC aa C38:6 = PC 16:0_22:6 + PC 18:1_20:5 + PC 18:2_20:4 + R

PC 18:1_20:5 excluded because of missingness > 75%

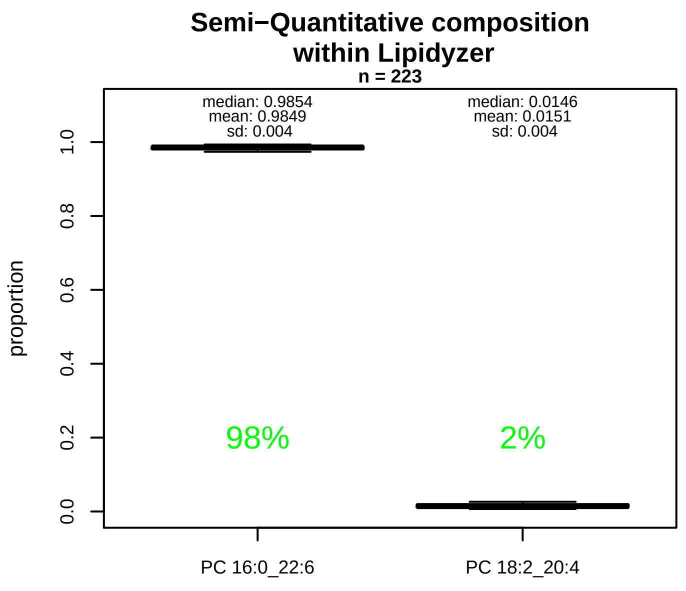

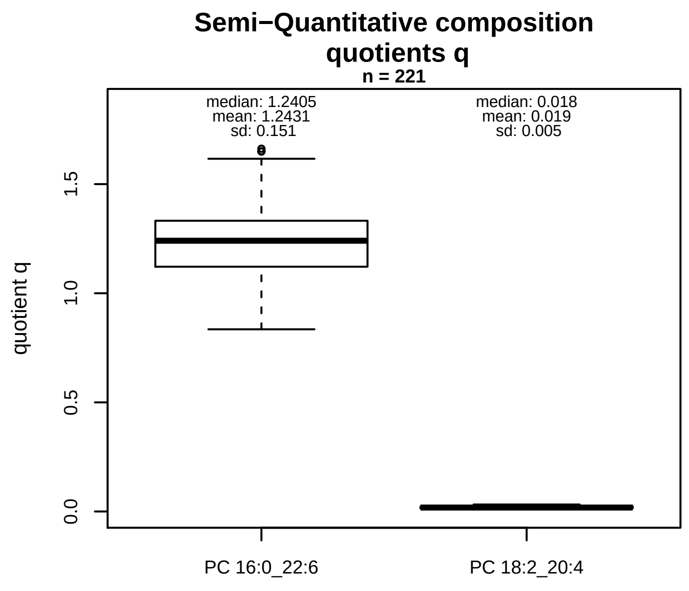

Semi−Quantitative composition: Lipidyzer

PC aa C38:6 consists of:
PC 16:0_22:6 98.5%
PC 18:2_20:4 1.5%
and of (not quantified):
PC 18:1_20:5, PC 16:1_22:5, PC 18:3_20:3, PC 18:4_20:2, and further compounds

Composition: mean of proportions q

conc(PC aa C38:6) * 1.2431 = conc(PC 16:0_22:6) [var(q)=1.2431]
Percentiles: 5%−>1.0168, 25%−>1.1214, 75%−>1.3321, 95%−>1.5009
conc(PC aa C38:6) * 0.019 = conc(PC 18:2_20:4) [var(q)=0.019]
Percentiles: 5%−>0.0118, 25%−>0.0148, 75%−>0.0234, 95%−>0.0279

Linear model

```
PC aa C38:6 ~ b * ( PC 16:0_22:6
   + PC 18:2_20:4 )
```

b = 0.71118

R^2 = 0.77057

Ranges

Measure	AbsoluteIDQ	sum(Lipidyzer)	delta
Min	50.4	67.49	17.09
Max	141.6	188.25	46.65
Mean	87.19	109.18	21.99
Median	82.9	100.57	17.67
SD	21.12	26.01	4.89

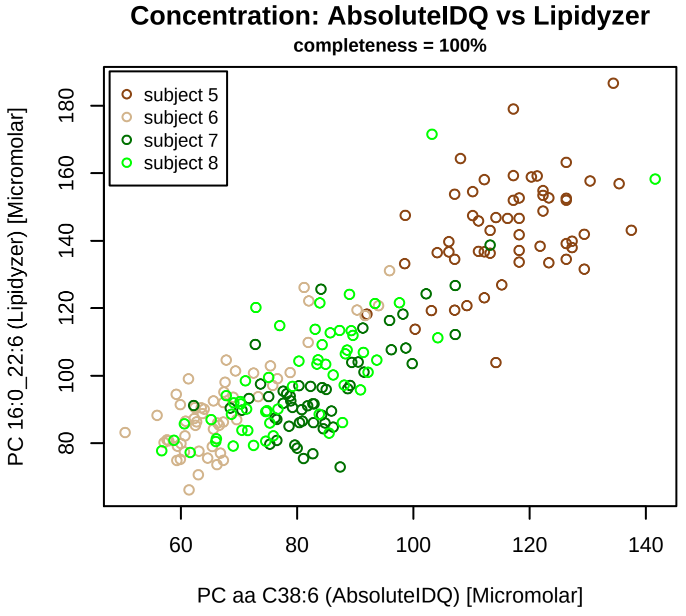

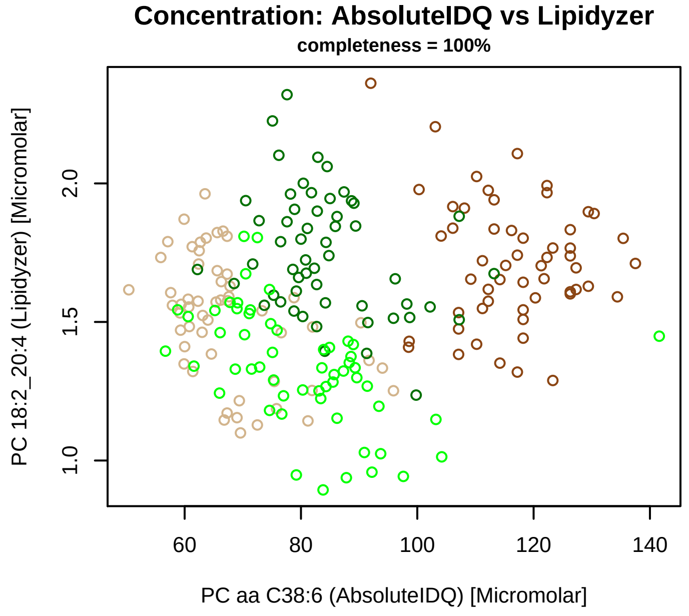

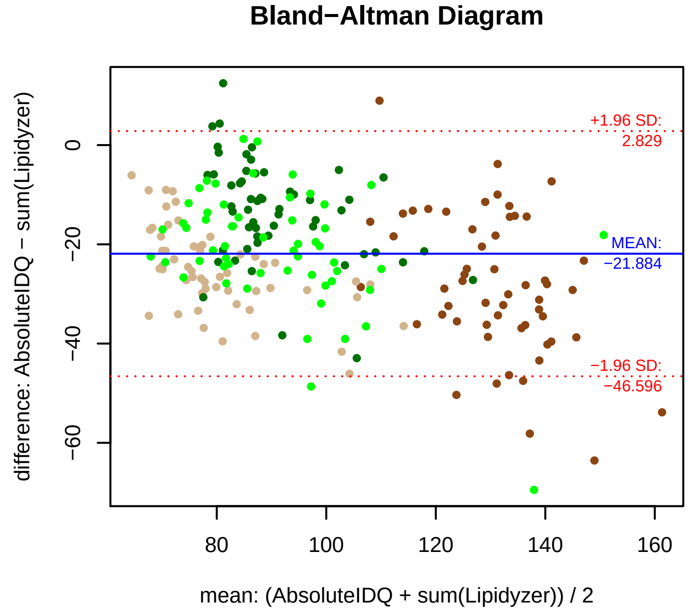

Stability of composition

(PC 16:0_22:6 + PC 18:2_20:4) / PC aa C38:6
Shapiro−Wilk Test of log quotients, pv: 0.86063; OK
SW: ok; ANOVA: Comp. ~ Subject_ID −> pv = 0.06938; EQUAL
Wilcoxon: Challenge; fasting: 0.625; sport: 0.625; OLTT: 0.25

PC 16:0_22:6 / PC aa C38:6
Shapiro−Wilk Test of log quotients, pv: 0.79949; OK
SW: ok; ANOVA: Comp. ~ Subject_ID −> pv = 0.05839; EQUAL
Wilcoxon: Challenge; fasting: 0.625; sport: 0.625; OLTT: 0.25

PC 18:2_20:4 / PC aa C38:6
Shapiro−Wilk Test of log quotients, pv: 0.00068; NO
SW: no; Kruskal: Comp. ~ Subject_ID −> pv = 0; DIFFERENCE
Wilcoxon: Challenge; fasting: 0.125; sport: 0.125; OLTT: 0.625

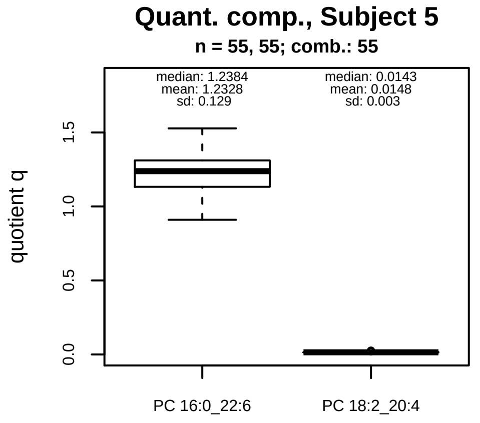

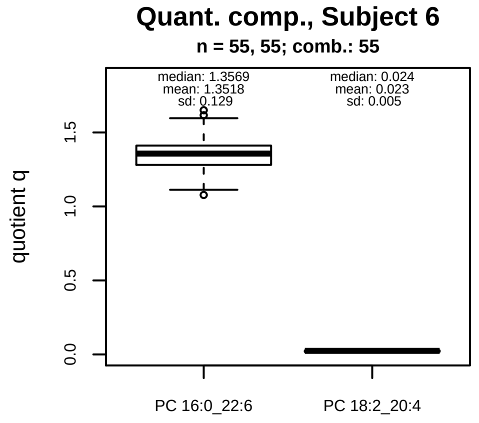

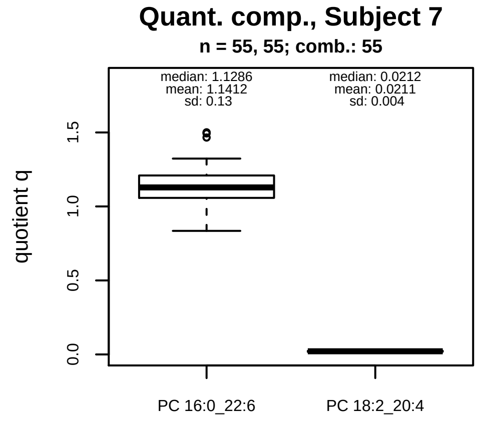

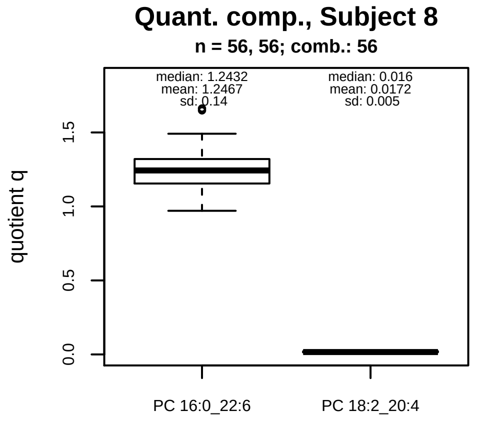

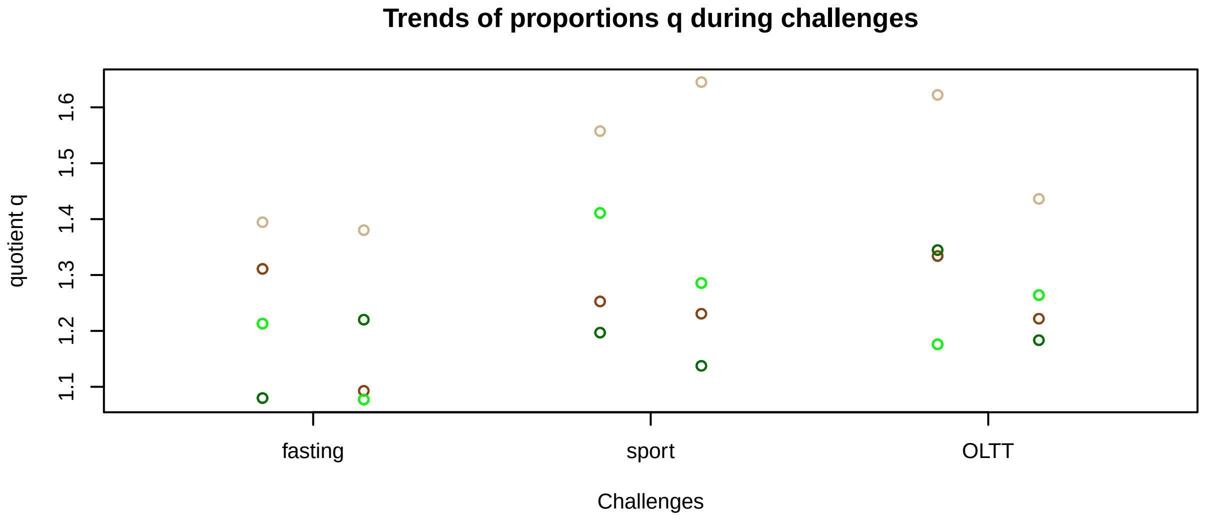

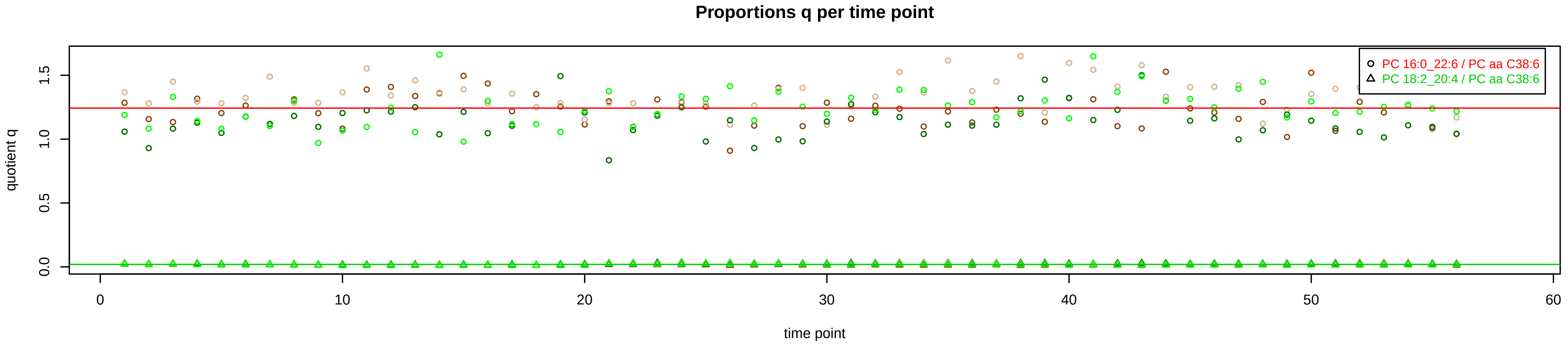

PC aa C40:1 = R

No independent Variable measured.

Qualitative composition

PC aa C40:1 consists of:
PC 16:0_24:1, PC 18:0_22:1, PC 18:1_22:0, PC 19:1_21:0,
PC O−20:1_21:0
and further compounds

PC aa C40:2 = PC 18:0_22:2 + PC 18:1_22:1 + R

PC 18:0_22:2, PC 18:1_22:1 excluded because of missingness > 75%

No independent Variable with coverage >0.25 out of PC 18:0_22:2, PC 18:1_22:1

Qualitative composition

PC aa C40:2 consists of:
PC 18:0_22:2, PC 18:1_22:1, PC 16:1_24:1, PC 18:2_22:0,
PC 20:1_20:1
and further compounds

PC aa C40:3 = PC 18:2_22:1 + PC 20:0_20:3 + R

PC 18:2_22:1 excluded because of missingness > 75%

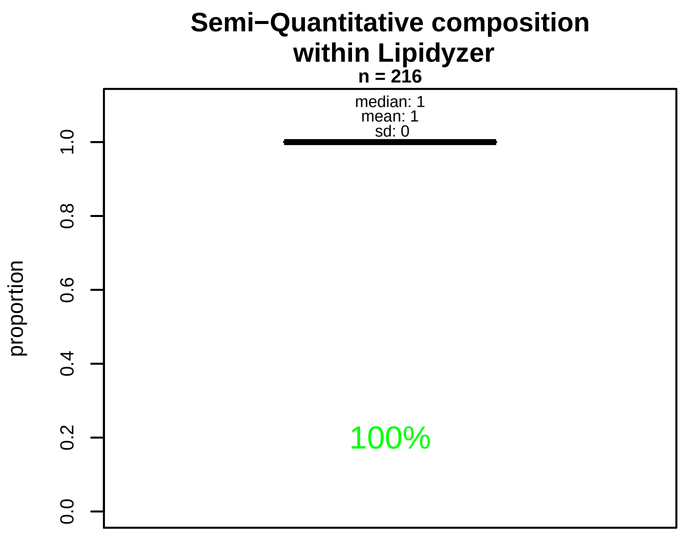

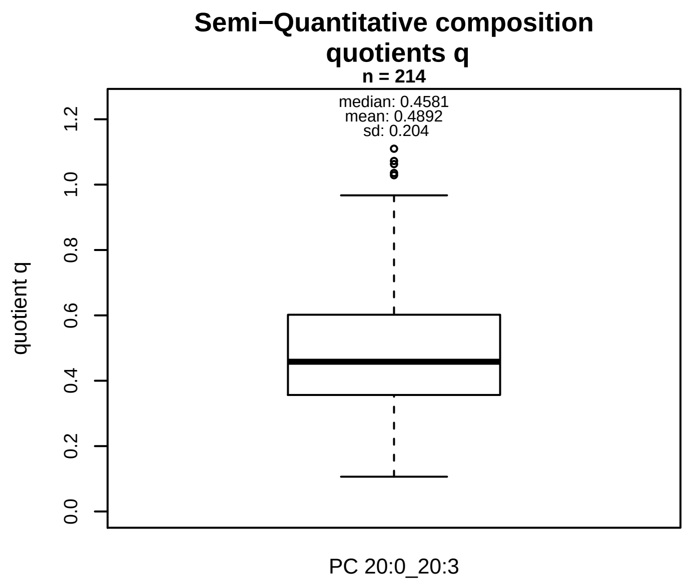

Semi−Quantitative composition: Lipidyzer

PC aa C40:3 consists of:
PC 20:0_20:3 100%
and of (not quantified):
PC 18:2_22:1, PC 18:0_22:3, PC 18:1_22:2, PC 18:3_22:0,
PC 20:1_20:2
and further compounds

Composition: mean of proportions q

conc(PC aa C40:3) * 0.4892 = conc(PC 20:0_20:3) [var(q)=0.4892]
Percentiles: 5%−>0.198, 25%−>0.3568, 75%−>0.5993, 95%−>0.8453

Linear model

```
PC aa C40:3 ~ b * ( PC 20:0_20:3 )
```

b = −0.06122

R^2 = 0.00209

Ranges

Measure	AbsoluteIDQ	sum(Lipidyzer)	delta
Min	0.42	0.11	0.31
Max	1.31	0.96	0.35
Mean	0.77	0.36	0.41
Median	0.73	0.32	0.41
SD	0.19	0.14	0.05

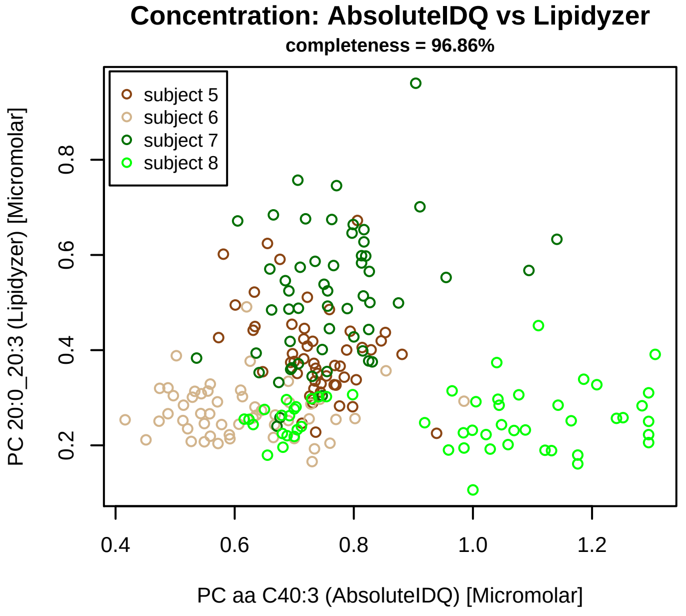

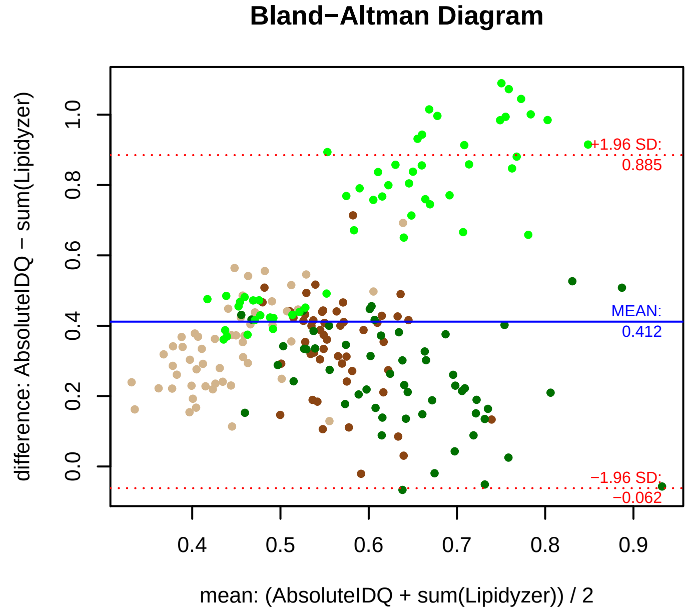

Stability of composition

PC 20:0_20:3 / PC aa C40:3
Shapiro−Wilk Test of log quotients, pv: 0.0089; NO
SW: ok; ANOVA: Comp. ~ Subject_ID −> pv = 0; DIFFERENCE
Wilcoxon: Challenge; fasting: 1; sport: 0.375; OLTT: 0.125

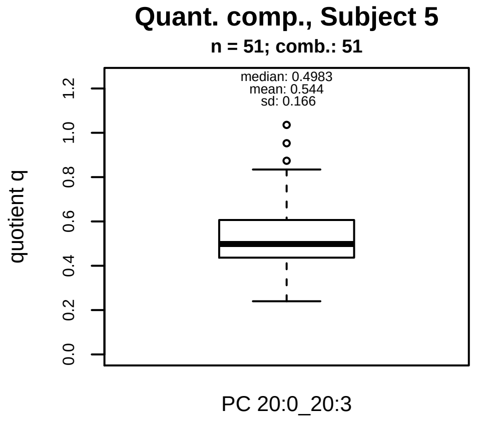

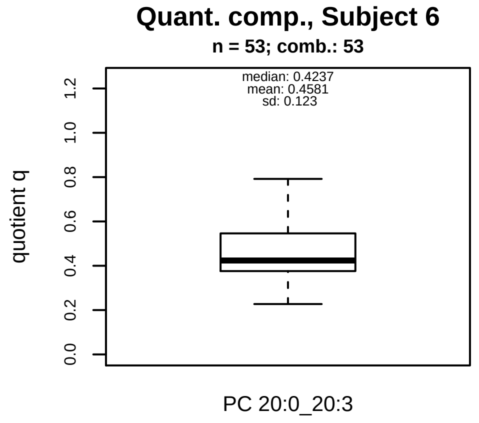

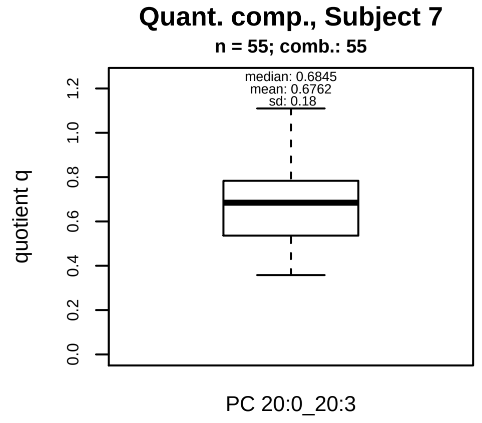

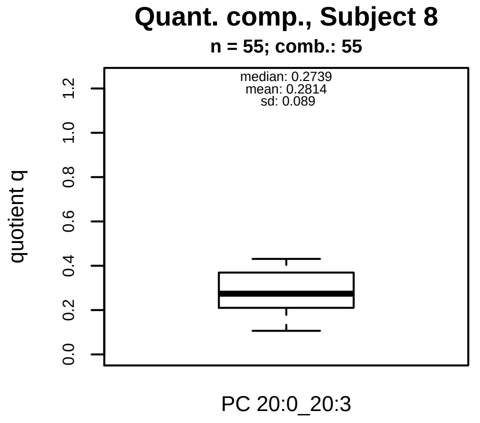

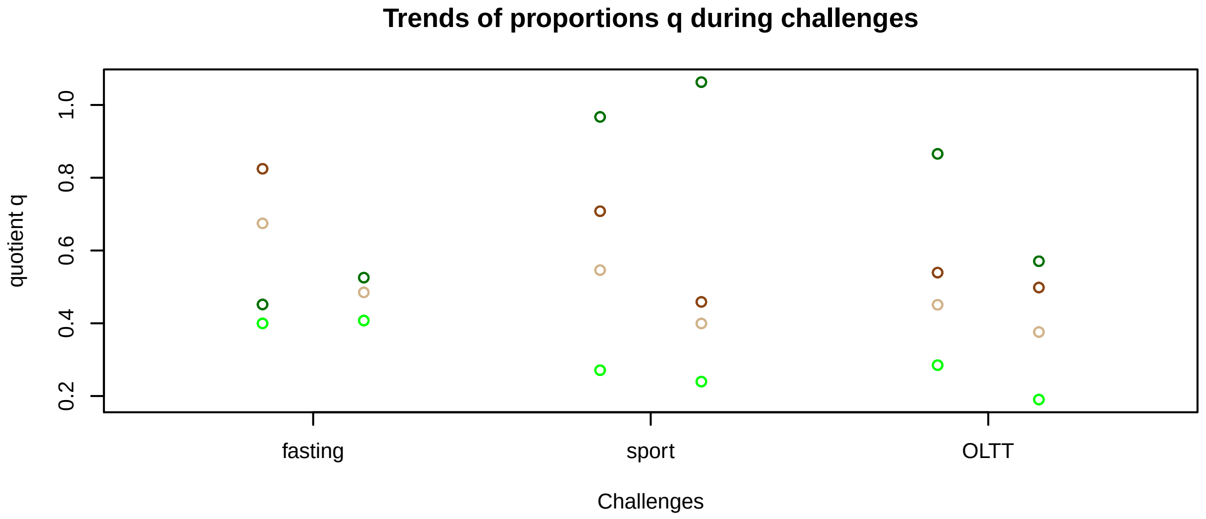

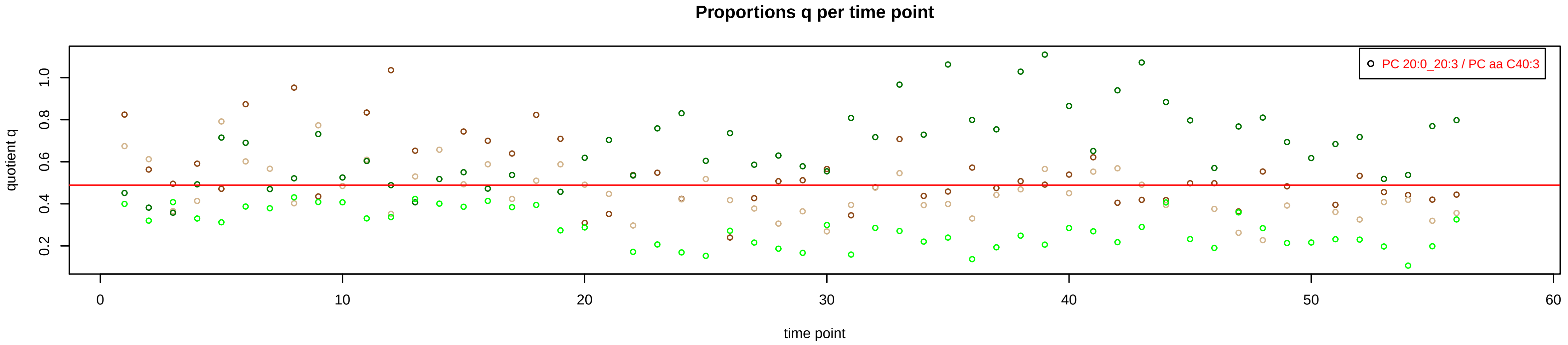

PC aa C40:4 = PC 18:0_22:4 + PC 20:0_20:4 + R

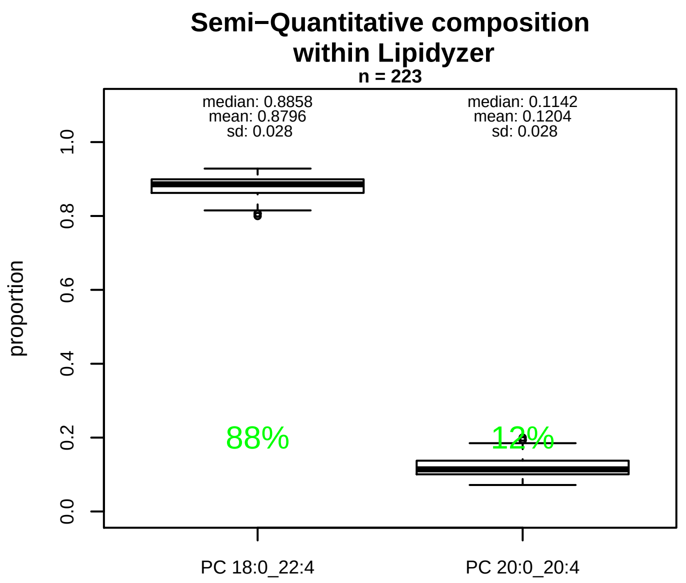

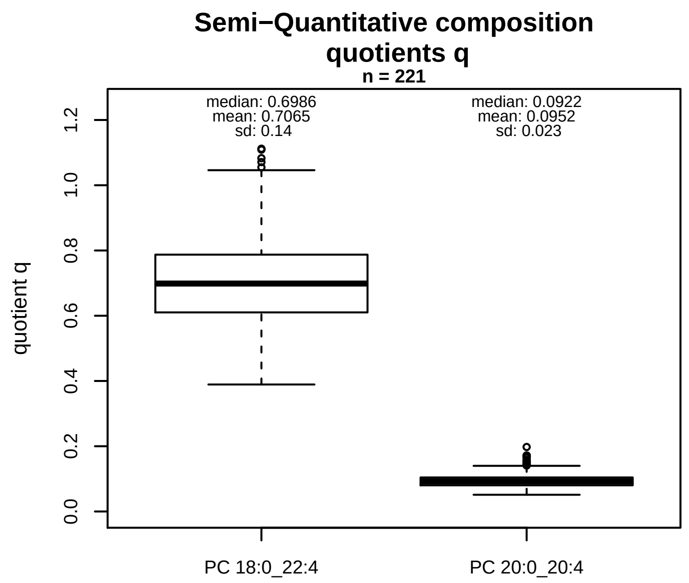

Semi−Quantitative composition: Lipidyzer

PC aa C40:4 consists of:
PC 18:0_22:4 88%
PC 20:0_20:4 12%
and of (not quantified):
PC 18:2_22:2, PC 18:3_22:1, PC 18:4_22:0, PC 20:1_20:3, PC 20:2_20:2
and further compounds

Linear model

```
PC aa C40:4 ~ b * ( PC 18:0_22:4
   + PC 20:0_20:4 )
```

b = 0.82589

R^2 = 0.67225

Ranges

Measure	AbsoluteIDQ	sum(Lipidyzer)	delta
Min	1.84	1.08	0.76
Max	5.56	6.11	0.55
Mean	3.46	2.79	0.67
Median	3.25	2.85	0.4
SD	0.89	0.88	0

Composition: mean of proportions q

conc(PC aa C40:4) * 0.7065 = conc(PC 18:0_22:4) [var(q)=0.7065]
Percentiles: 5%−>0.4936, 25%−>0.6103, 75%−>0.7872, 95%−>0.9503
conc(PC aa C40:4) * 0.0952 = conc(PC 20:0_20:4) [var(q)=0.0952]
Percentiles: 5%−>0.0644, 25%−>0.0803, 75%−>0.1044, 95%−>0.1461

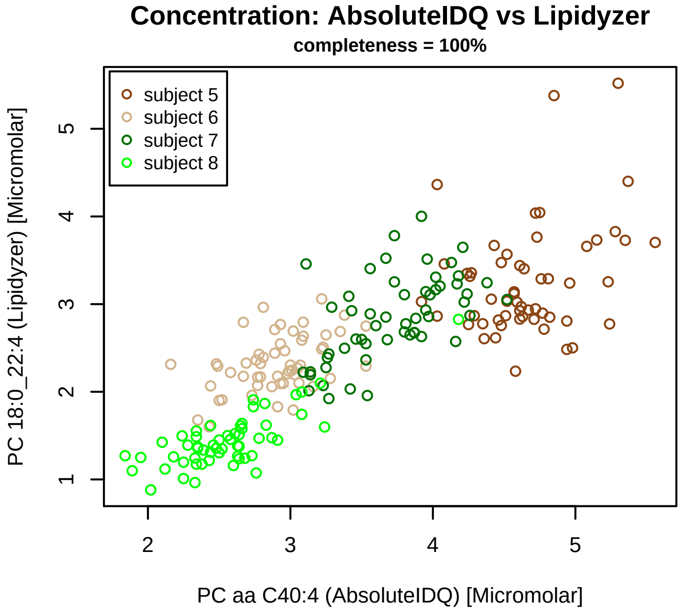

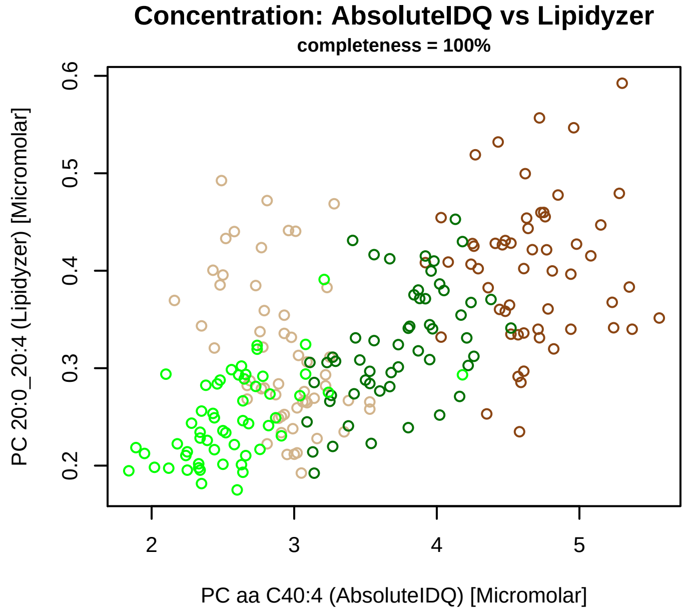

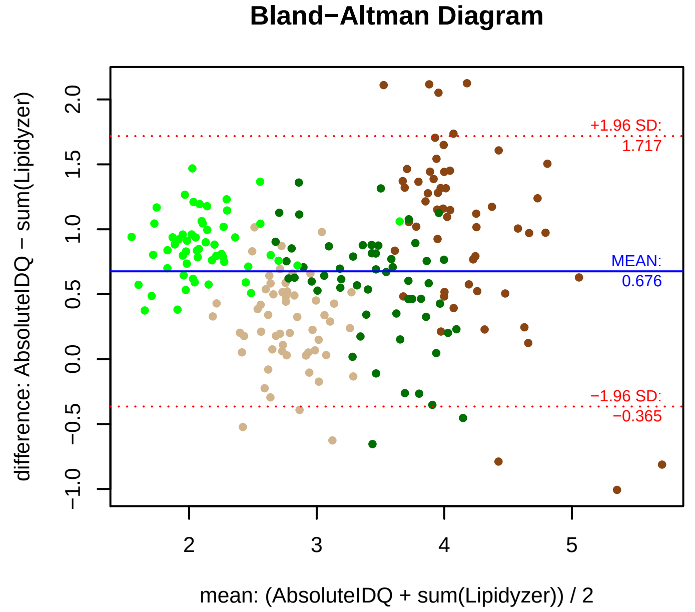

Stability of composition

(PC 18:0_22:4 + PC 20:0_20:4) / PC aa C40:4
Shapiro−Wilk Test of log quotients, pv: 0.92811; OK
SW: ok; ANOVA: Comp. ~ Subject_ID −> pv = 0; DIFFERENCE
Wilcoxon: Challenge; fasting: 0.25; sport: 0.875; OLTT: 0.125

PC 18:0_22:4 / PC aa C40:4
Shapiro−Wilk Test of log quotients, pv: 0.64098; OK
SW: ok; ANOVA: Comp. ~ Subject_ID −> pv = 0; DIFFERENCE
Wilcoxon: Challenge; fasting: 0.25; sport: 0.875; OLTT: 0.125

PC 20:0_20:4 / PC aa C40:4
Shapiro−Wilk Test of log quotients, pv: 0.00125; NO
SW: ok; ANOVA: Comp. ~ Subject_ID −> pv = 0.19594; EQUAL
Wilcoxon: Challenge; fasting: 0.375; sport: 1; OLTT: 0.25

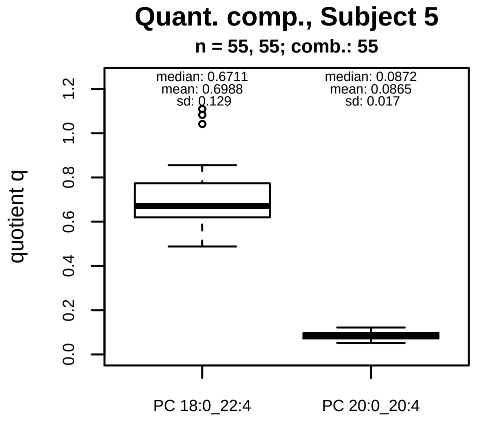

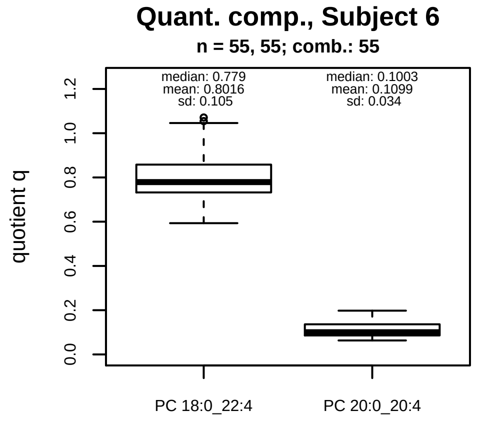

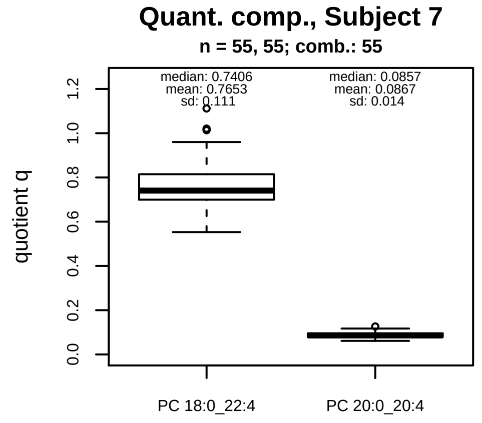

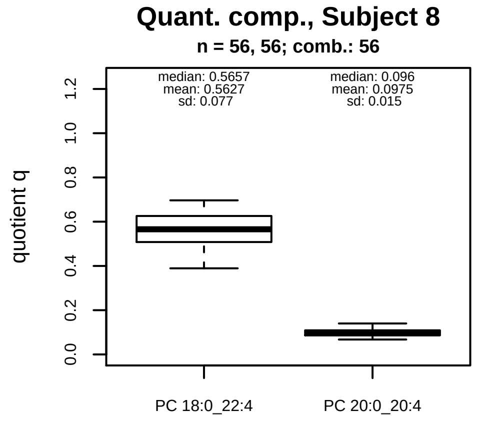

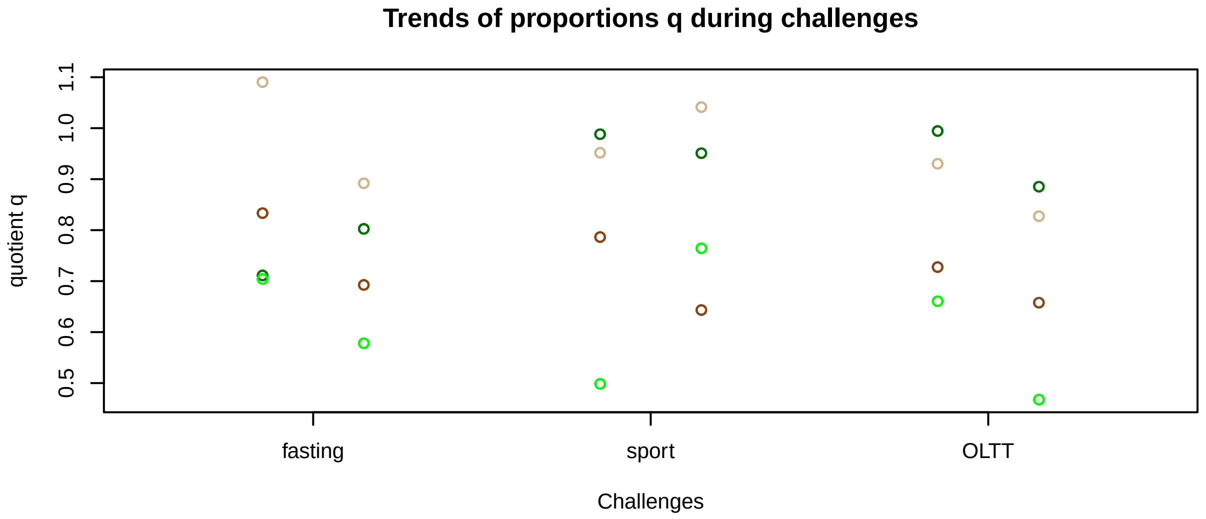

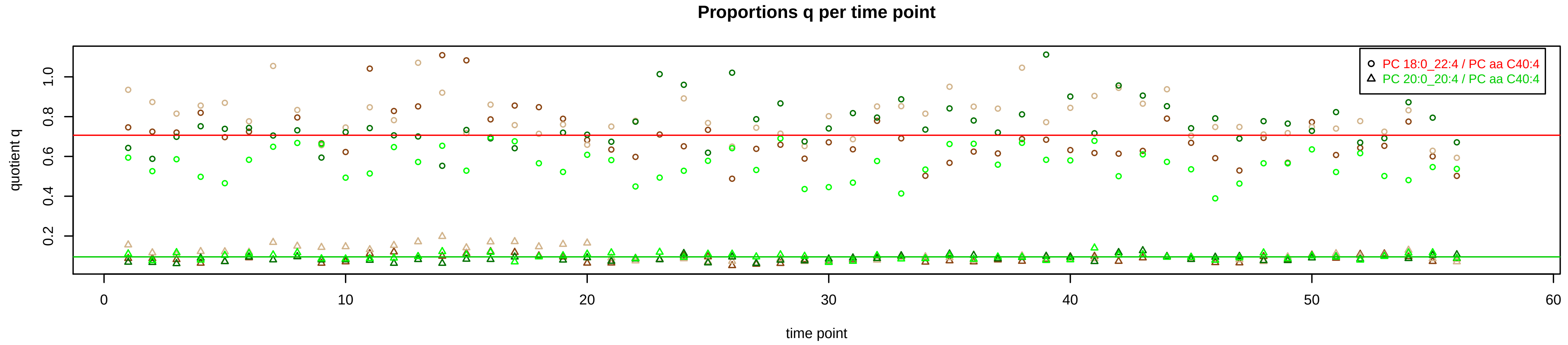

PC aa C40:5 = PC 18:0_22:5 + PC 18:1_22:4 + R

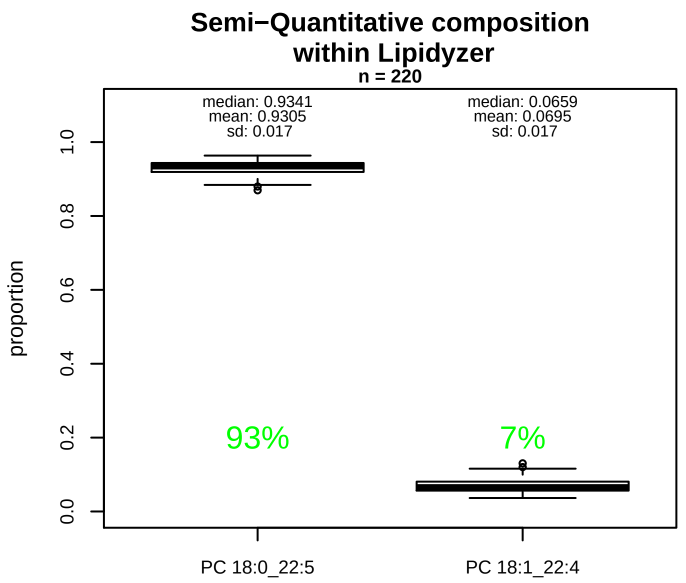

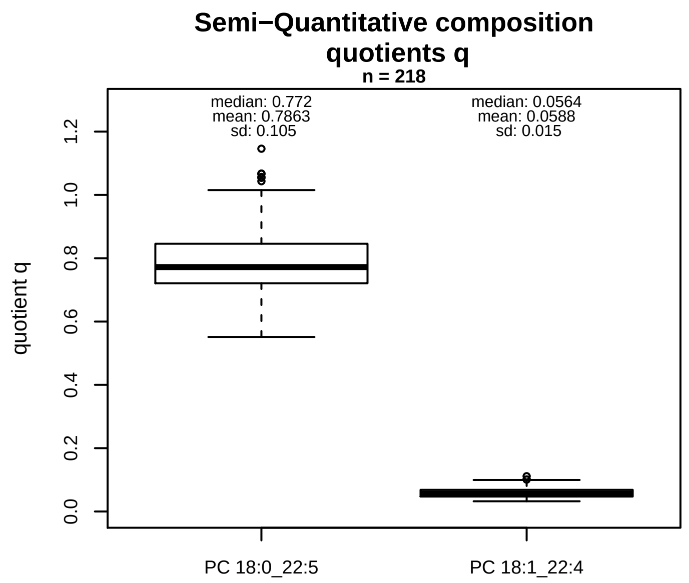

Semi−Quantitative composition: Lipidyzer

PC aa C40:5 consists of:
PC 18:0_22:5 93%
PC 18:1_22:4 7%
and of (not quantified):
PC 18:3_22:2, PC 18:4_22:1, PC 20:0_20:5, PC 20:1_20:4,
PC 20:2_20:3
and further compounds

Linear model

```
PC aa C40:5 ~ b * ( PC 18:0_22:5
   + PC 18:1_22:4 )
```

b = 1.00995

R^2 = 0.76522

Ranges

Measure	AbsoluteIDQ	sum(Lipidyzer)	delta
Min	5.45	4.55	0.9
Max	16.1	16.02	0.08
Mean	10.39	8.74	1.65
Median	9.76	8.55	1.21
SD	2.72	2.34	0.37

Composition: mean of proportions q

conc(PC aa C40:5) * 0.7863 = conc(PC 18:0_22:5) [var(q)=0.7863]
Percentiles: 5%−>0.6343, 25%−>0.721, 75%−>0.8457, 95%−>0.9819
conc(PC aa C40:5) * 0.0588 = conc(PC 18:1_22:4) [var(q)=0.0588]
Percentiles: 5%−>0.0373, 25%−>0.0473, 75%−>0.0684, 95%−>0.084

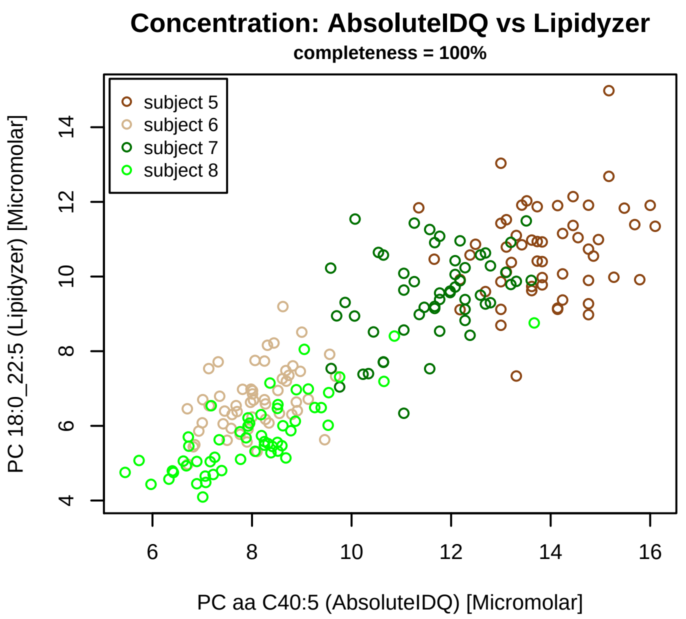

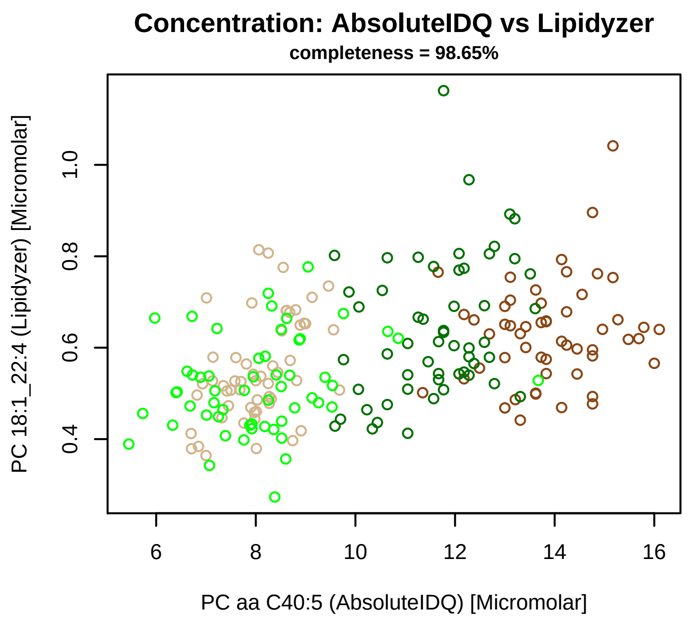

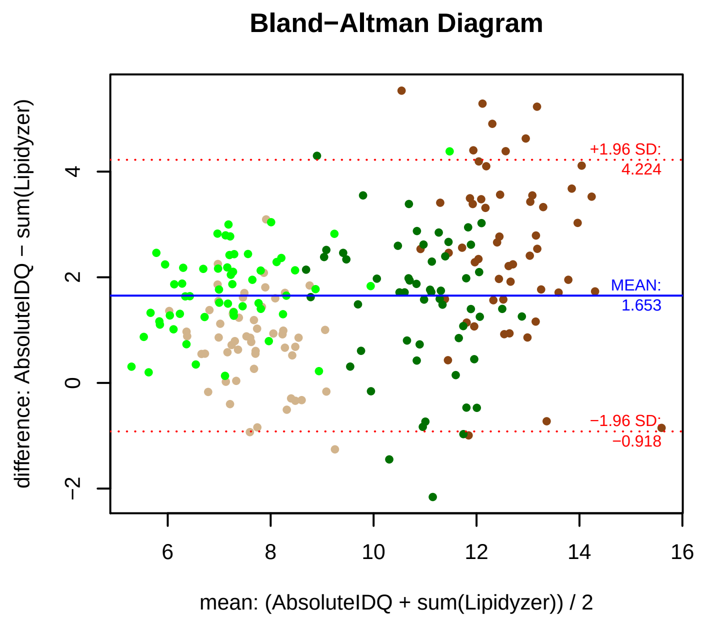

Stability of composition

(PC 18:0_22:5 + PC 18:1_22:4) / PC aa C40:5
Shapiro−Wilk Test of log quotients, pv: 0.55079; OK
SW: ok; ANOVA: Comp. ~ Subject_ID −> pv = 0.06767; EQUAL
Wilcoxon: Challenge; fasting: 0.25; sport: 0.125; OLTT: 0.25

PC 18:0_22:5 / PC aa C40:5
Shapiro−Wilk Test of log quotients, pv: 0.63789; OK
SW: ok; ANOVA: Comp. ~ Subject_ID −> pv = 0.00534; DIFFERENCE
Wilcoxon: Challenge; fasting: 0.25; sport: 0.125; OLTT: 0.25

PC 18:1_22:4 / PC aa C40:5
Shapiro−Wilk Test of log quotients, pv: 0.30263; OK
SW: ok; ANOVA: Comp. ~ Subject_ID −> pv = 0; DIFFERENCE
Wilcoxon: Challenge; fasting: 0.125; sport: 0.625; OLTT: 1

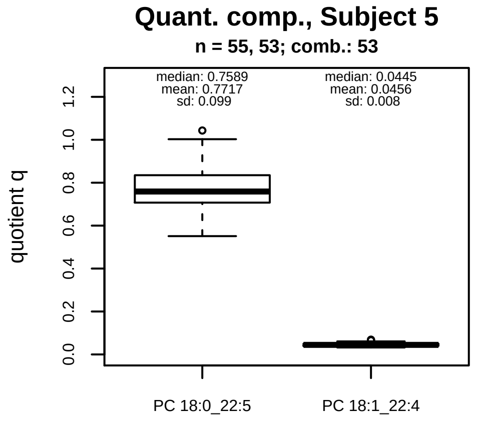

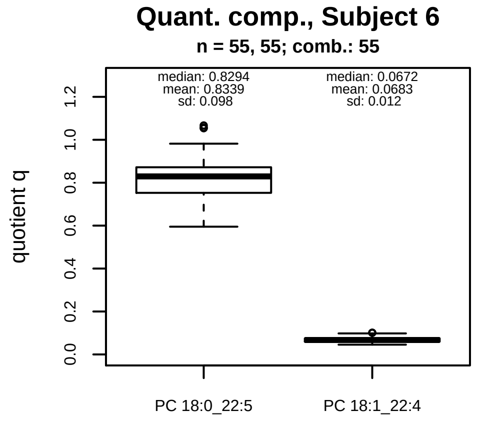

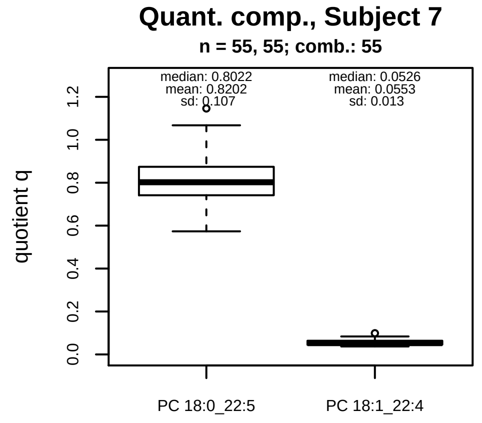

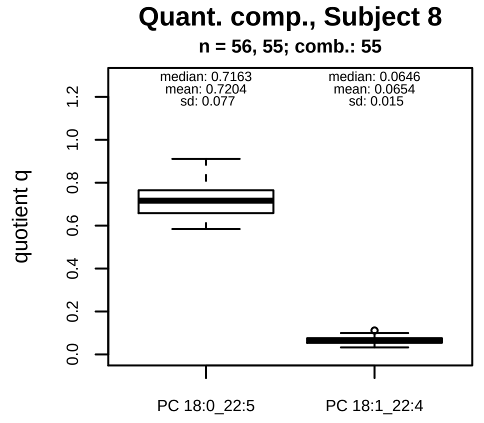

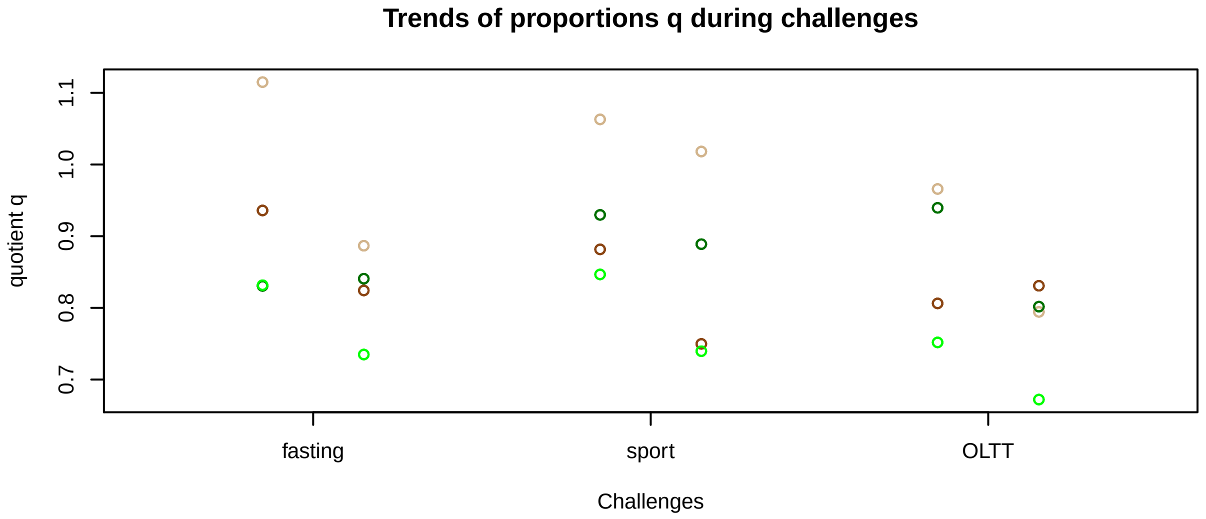

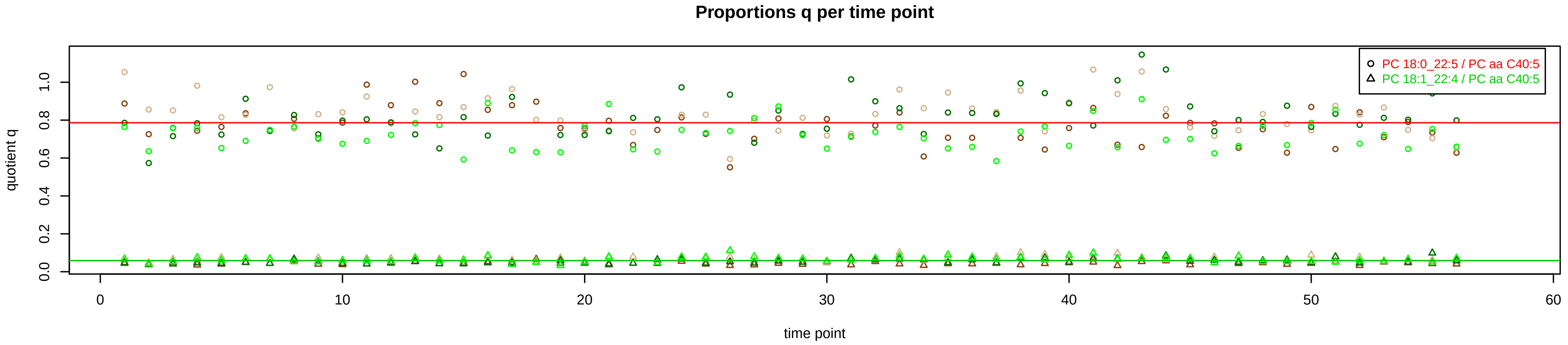

PC aa C40:6 = PC 18:0_22:6 + PC 18:1_22:5 + PC 18:2_22:4 + R

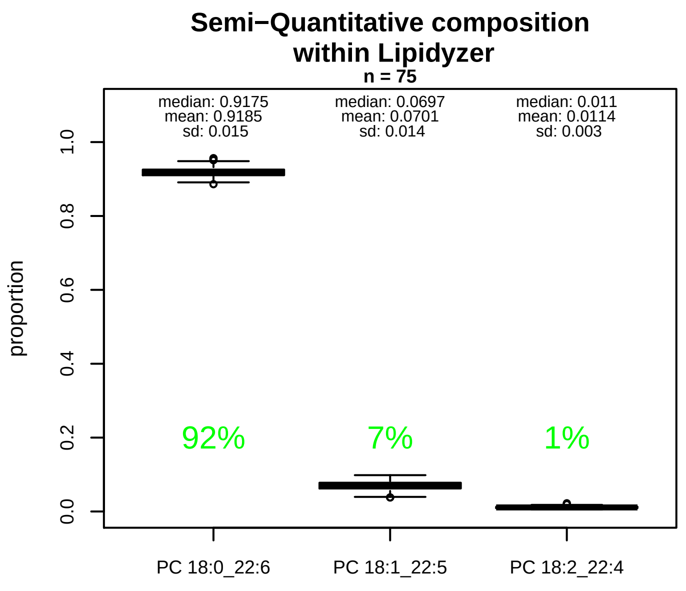

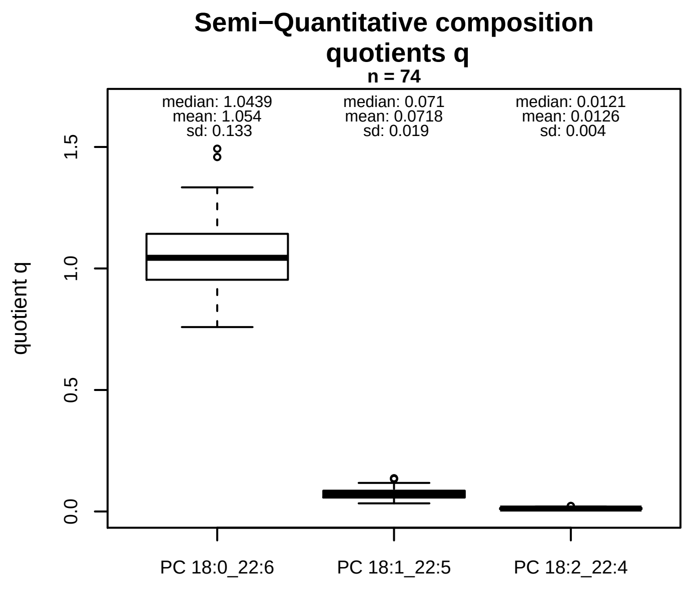

Semi−Quantitative composition: Lipidyzer

PC aa C40:6 consists of:
- PC 18:0_22:6 91.8%
- PC 18:1_22:5 7%
- PC 18:2_22:4 1.1%

and of (not quantified):
PC 18:4_22:2, PC 20:1_20:5, PC 20:2_20:4, PC 20:3_20:3, and further compounds

Linear model

```
PC aa C40:6 ~ b * ( PC 18:0_22:6
   + PC 18:1_22:5
   + PC 18:2_22:4 )
```

b = 0.91019

R^2 = 0.82665

Ranges

Measure	AbsoluteIDQ	sum(Lipidyzer)	delta
Min	14.3	16.78	2.48
Max	43.5	43.62	0.12
Mean	25.21	24.91	0.3
Median	23.5	24.17	0.67
SD	7.46	5.35	2.11

Composition: mean of proportions q

conc(PC aa C40:6) * 1.054 = conc(PC 18:0_22:6) [var(q)=1.054]
Percentiles: 5%−>0.8588, 25%−>0.9535, 75%−>1.1426, 95%−>1.2734
conc(PC aa C40:6) * 0.0718 = conc(PC 18:1_22:5) [var(q)=0.0718]
Percentiles: 5%−>0.0429, 25%−>0.0573, 75%−>0.0856, 95%−>0.1039
conc(PC aa C40:6) * 0.0126 = conc(PC 18:2_22:4) [var(q)=0.0126]
Percentiles: 5%−>0.0076, 25%−>0.0099, 75%−>0.0147, 95%−>0.0186

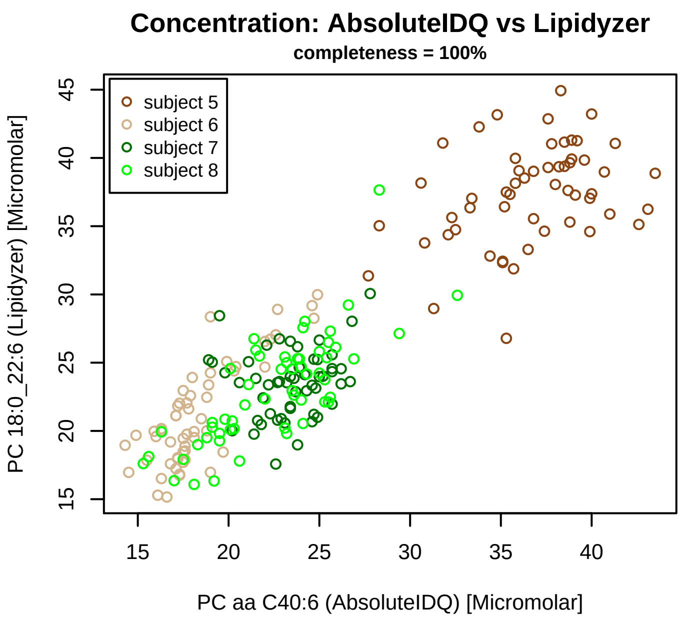

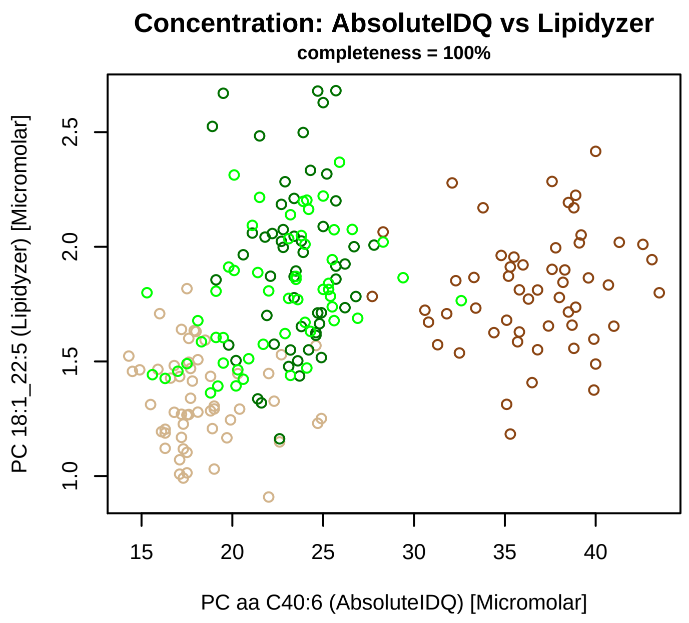

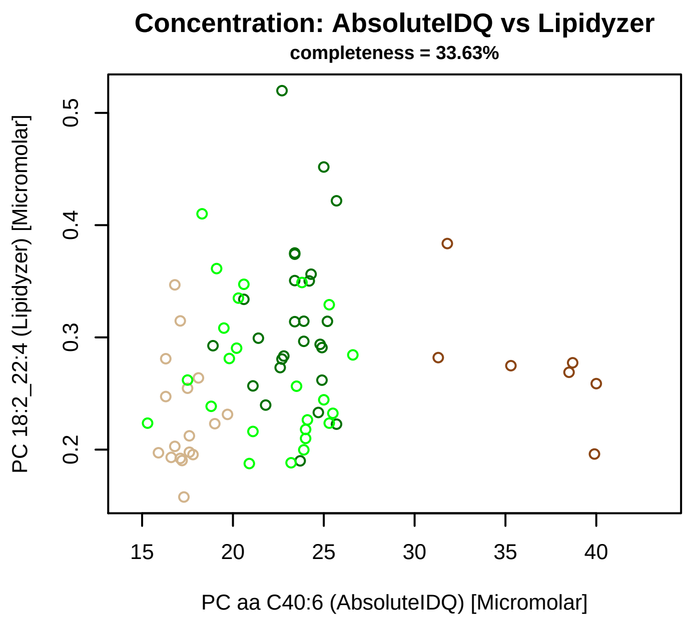

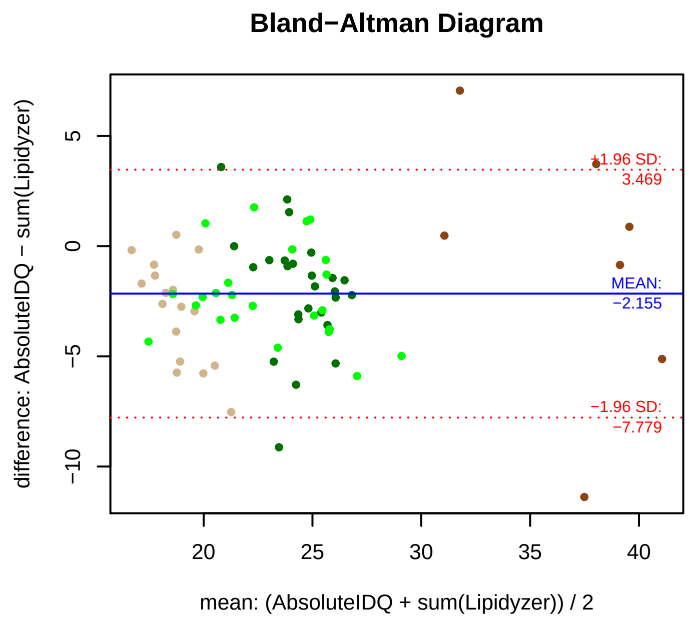

Stability of composition

(PC 18:0_22:6 + PC 18:1_22:5 + PC 18:2_22:4) / PC aa C40:6
Shapiro−Wilk Test of log quotients, pv: 0.76064; OK
SW: ok; ANOVA: Comp. ~ Subject_ID −> pv = 0.82272; EQUAL
Wilcoxon: Challenge; sport: 1

PC 18:0_22:6 / PC aa C40:6
Shapiro−Wilk Test of log quotients, pv: 0.25525; OK
SW: ok; ANOVA: Comp. ~ Subject_ID −> pv = 0.08; EQUAL
Wilcoxon: Challenge; fasting: 0.125; sport: 0.875; OLTT: 0.375

PC 18:1_22:5 / PC aa C40:6
Shapiro−Wilk Test of log quotients, pv: 0.04469; OK
SW: ok; ANOVA: Comp. ~ Subject_ID −> pv = 0; DIFFERENCE
Wilcoxon: Challenge; fasting: 0.625; sport: 0.875; OLTT: 0.875

PC 18:2_22:4 / PC aa C40:6
Shapiro−Wilk Test of log quotients, pv: 0.65312; OK
SW: ok; ANOVA: Comp. ~ Subject_ID −> pv = 0.02697; EQUAL
Wilcoxon: Challenge; sport: 1

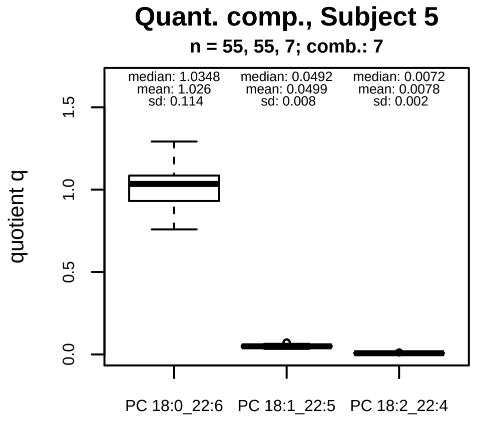

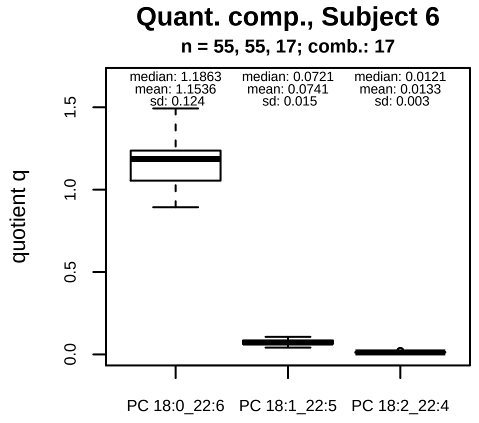

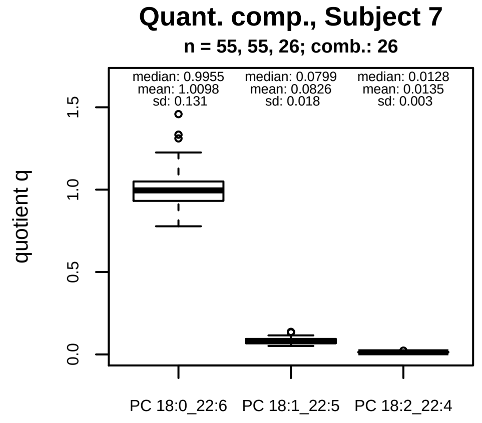

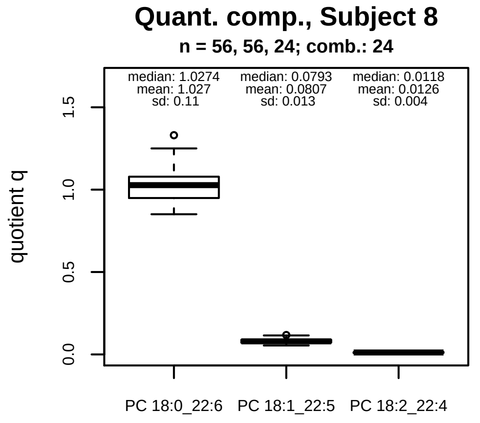

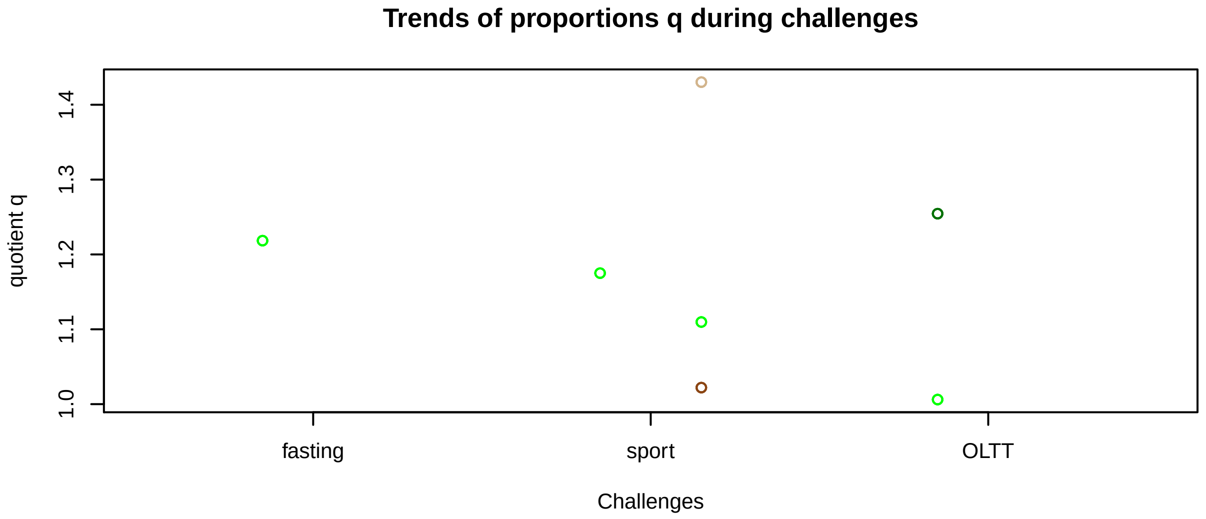

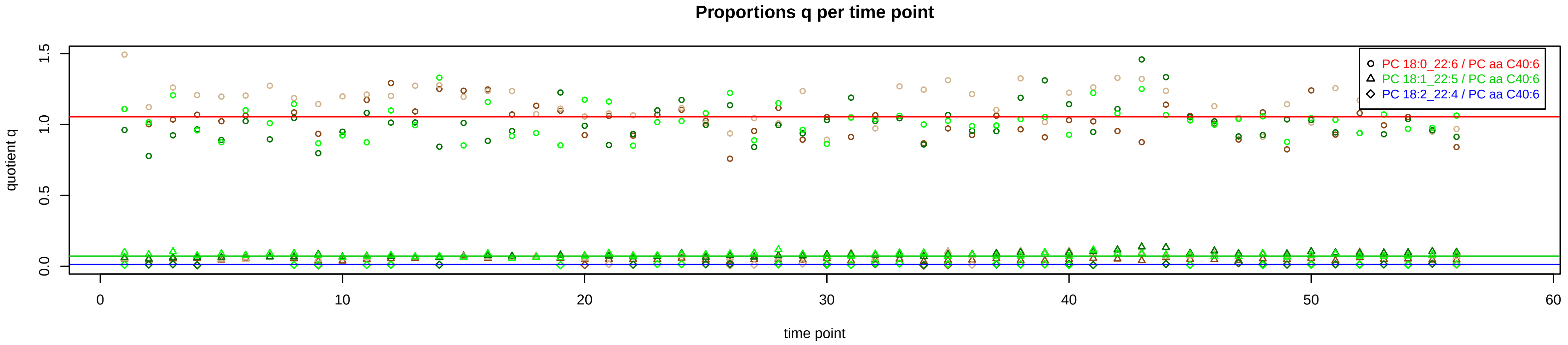

PC aa C42:0 = R

No independent Variable measured.

Qualitative composition

PC aa C42:0 consists of:
PC 16:0_26:0, PC 18:0_24:0, PC 20:0_22:0, PC 21:0_21:0,
and further compounds

PC aa C42:1 = R

No independent Variable measured.

Qualitative composition

PC aa C42:1 consists of:
PC 18:0_24:1, PC 18:1_24:0, PC 20:0_22:1, PC 20:1_22:0,
and further compounds

PC aa C42:2 = R

No independent Variable measured.

Qualitative composition

PC aa C42:2 consists of:
PC 16:0_26:2, PC 18:1_24:1, PC 18:2_24:0, PC 20:0_22:2,
PC 20:2_22:0
and further compounds

PC aa C42:4 = R

No independent Variable measured.

Qualitative composition

PC aa C42:4 consists of:
PC 18:3_24:1, PC 18:4_24:0, PC 20:0_22:4, PC 20:2_22:2,
PC 20:4_22:0
and further compounds

PC aa C42:5 = PC 20:0_22:5 + R

PC 20:0_22:5 excluded because of missingness > 75%

No independent Variable with coverage >0.25 out of PC 20:0_22:5

Qualitative composition

PC aa C42:5 consists of:
PC 20:0_22:5, PC 20:1_22:4, PC 20:3_22:2, PC 20:4_22:1,
PC 20:5_22:0
and further compounds

PC aa C42:6 = PC 20:0_22:6 + R

Semi−Quantitative composition within Lipidyzer

n = 145

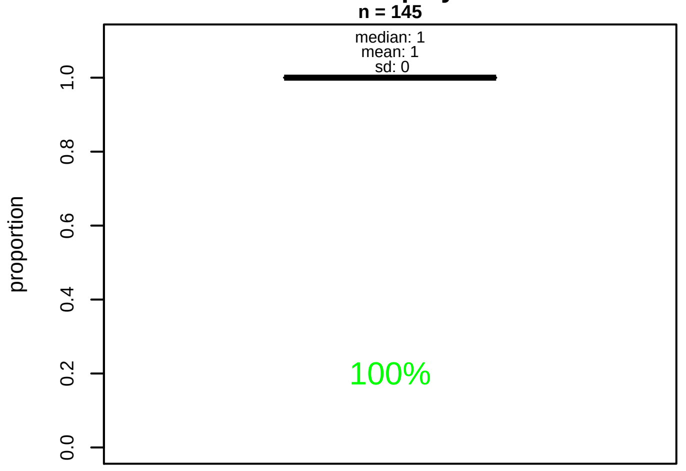

Semi−Quantitative composition quotients q

n = 144

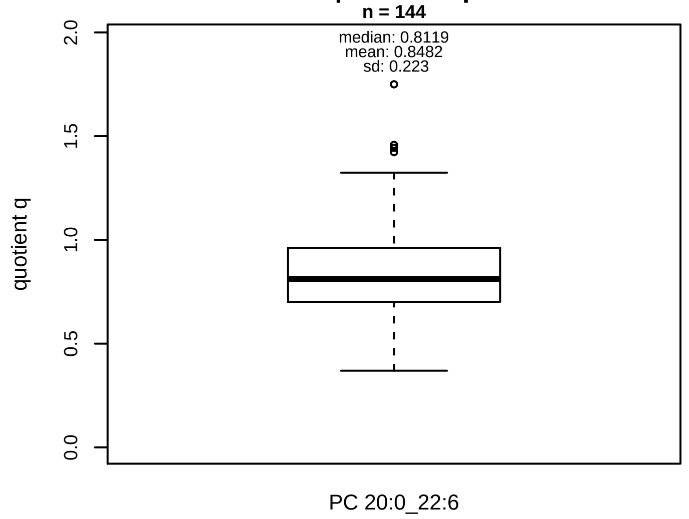

Semi−Quantitative composition: Lipidyzer

PC aa C42:6 consists of:
PC 20:0_22:6 100%
and of (not quantified):
PC 20:5_22:1
and further compounds

Composition: mean of proportions q

conc(PC aa C42:6) * 0.8482 = conc(PC 20:0_22:6) [var(q)=0.8482]
Percentiles: 5%−>0.5587, 25%−>0.703, 75%−>0.9614, 95%−>1.2468

Linear model

```
PC aa C42:6 ~ b * ( PC 20:0_22:6 )
```

b = 0.21904

R^2 = 0.05859

Ranges

Measure	AbsoluteIDQ	sum(Lipidyzer)	delta
Min	0.41	0.27	0.14
Max	0.97	0.86	0.11
Mean	0.69	0.58	0.11
Median	0.69	0.57	0.13
SD	0.13	0.13	0

Concentration: AbsoluteIDQ vs Lipidyzer

completeness = 65.02%

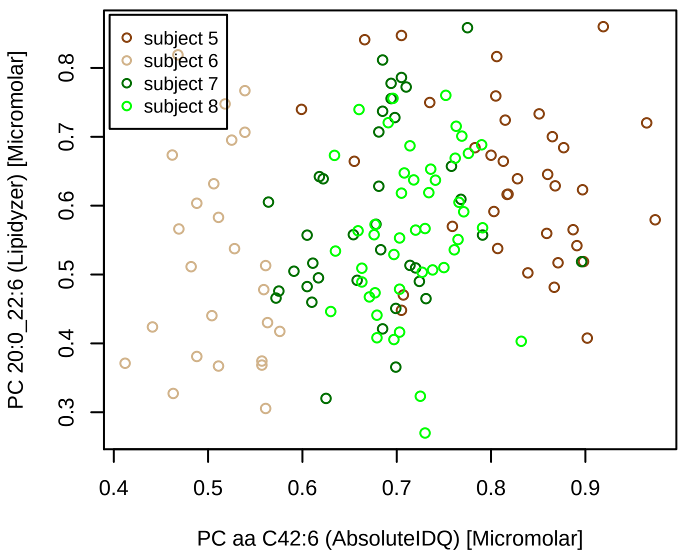

Bland−Altman Diagram

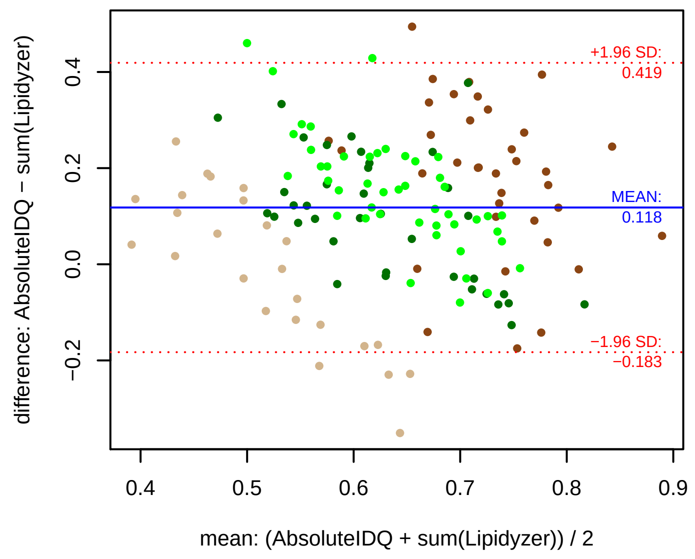

Stability of composition

PC 20:0_22:6 / PC aa C42:6
Shapiro−Wilk Test of log quotients, pv: 0.79973; OK
SW: no; Kruskal: Comp. ~ Subject_ID −> pv = 0.00367; DIFFERENCE
Wilcoxon: Challenge; fasting: 0.5; sport: 1; OLTT: 1

Quant. comp., Subject 5

n = 36; comb.: 36

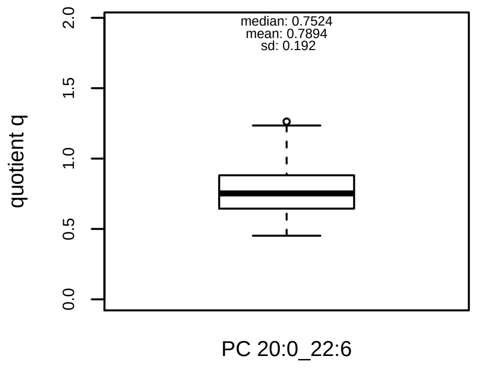

Quant. comp., Subject 6

n = 25; comb.: 25

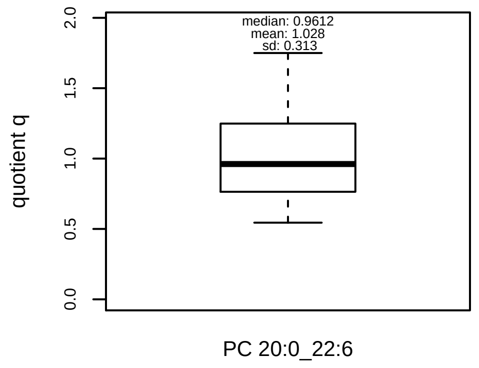

Quant. comp., Subject 7

n = 37; comb.: 37

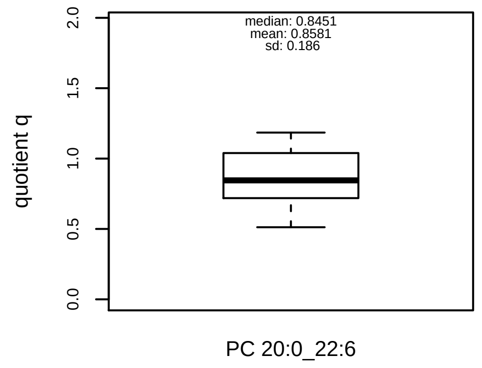

Quant. comp., Subject 8

n = 46; comb.: 46

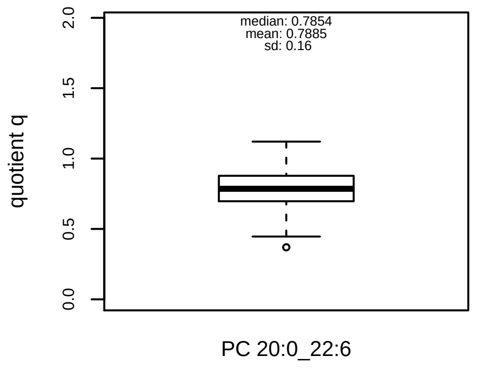

Trends of proportions q during challenges

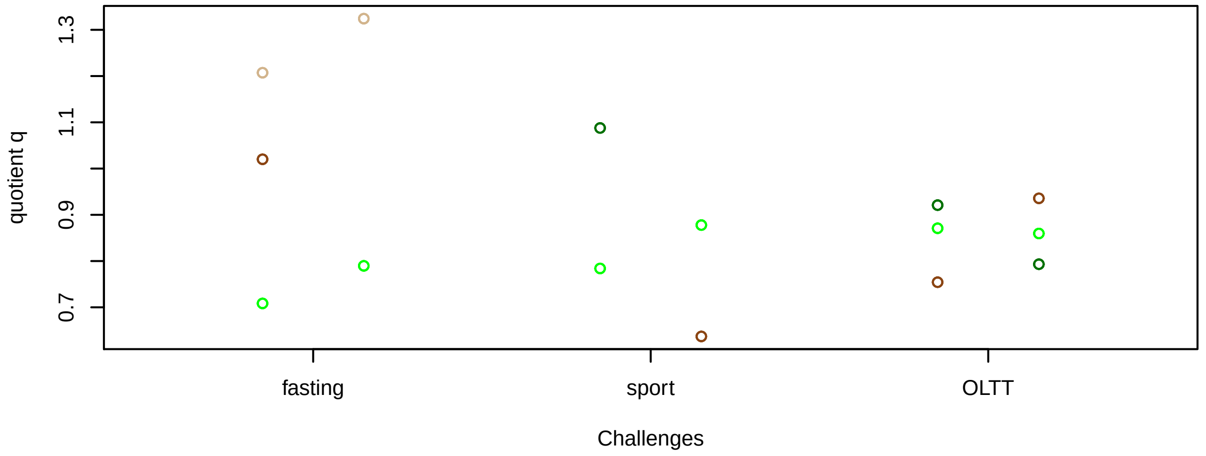

Proportions q per time point

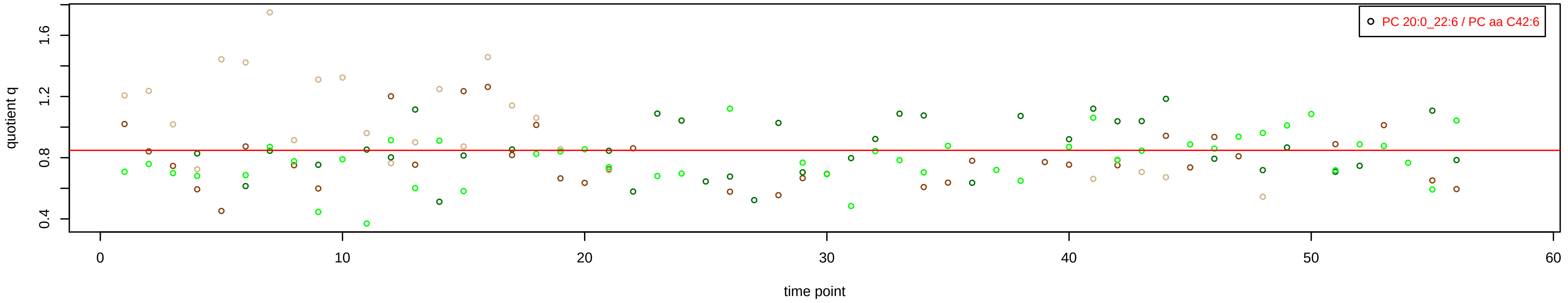

PC ae C30:0 = R

No independent Variable measured.

Qualitative composition

PC ae C30:0 consists of:
PC O−14:0_16:0, PC O−18:0_12:0, PC 10:0_19:0, PC 12:0_1
PC 13:0_16:0, PC 14:0_15:0, PC 20:0_9:0, PC 8:0_21:0,
[13C1]SM 33:0
and further compounds

PC ae C32:1 = PC 15:0_16:1 + PC 17:0_14:1 + R

PC 15:0_16:1, PC 17:0_14:1 excluded because of missingness > 75%

No independent Variable with coverage >0.25 out of PC 15:0_16:1, PC 17:0_14:1

Qualitative composition

PC ae C32:1 consists of:
PC 15:0_16:1, PC 17:0_14:1, PC O−14:0_18:1, PC O−16:0_[illegible]
PC O−18:0_14:1, PC O−20:1_12:0, PC 12:0_19:1, PC 13:0_[illegible]
PC 16:0_15:1
and further compounds

PC ae C32:2 = R

No independent Variable measured.

Qualitative composition

PC ae C32:2 consists of:
PC O−14:0_18:2, PC O−14:1_18:1, PC O−16:1_16:1, PC 13:
PC 15:1_16:1
and further compounds

PC ae C34:0 = R

No independent Variable measured.

Qualitative composition

PC ae C34:0 consists of:
PC O−16:0_18:0, PC O−17:0_17:0, PC O−20:0_14:0, PC 10:
PC 11:0_22:0, PC 13:0_20:0, PC 15:0_18:0, PC 16:0_17:0,
PC 21:0_12:0
and further compounds

PC ae C34:1 = PC 15:0_18:1 + PC 17:0_16:1 + R

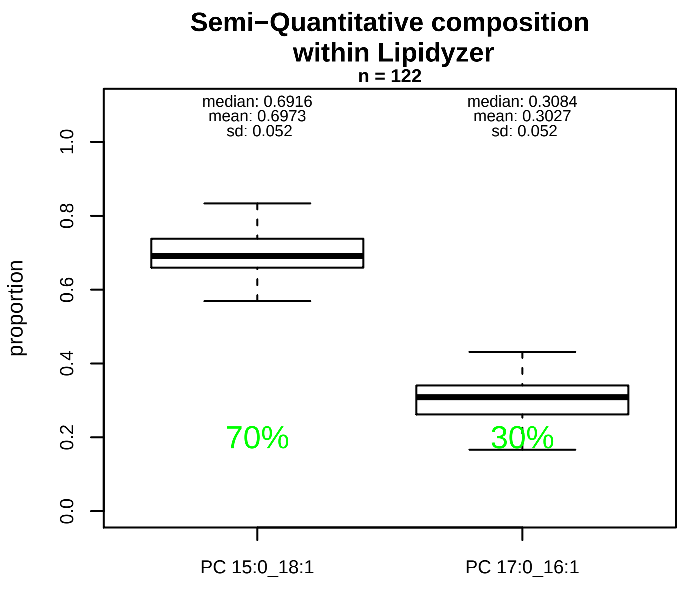

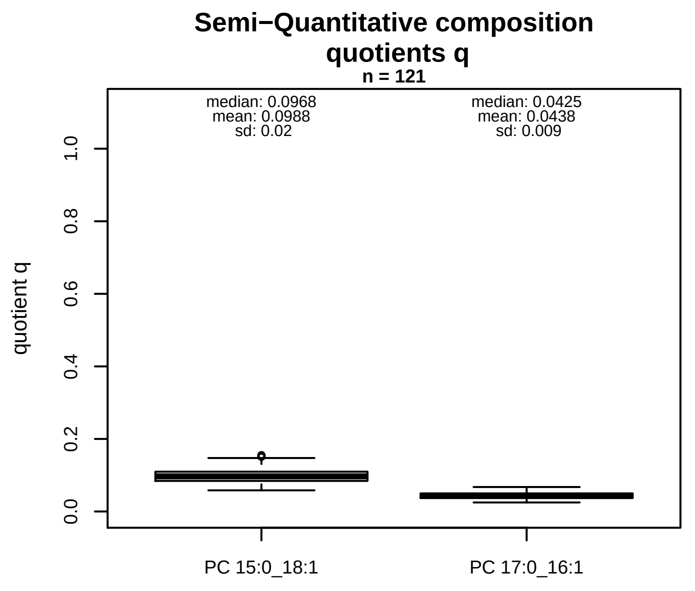

Semi−Quantitative composition: Lipidyzer

PC ae C34:1 consists of:
PC 15:0_18:1 69.7%
PC 17:0_16:1 30.3%
and of (not quantified):
PC O−16:0_18:1, PC O−18:0_16:1, PC O−20:0_14:1, PC O−PC 13:0_20:1, PC 14:0_19:1, PC 14:1_19:0, PC 15:1_18:0, [13C1]SM 37:1
and further compounds

Linear model

```
PC ae C34:1 ~ b * ( PC 15:0_18:1
   + PC 17:0_16:1 )
```

b = 4.14514

R^2 = 0.57752

Ranges

Measure	AbsoluteIDQ	sum(Lipidyzer)	delta
Min	5.38	0.78	4.6
Max	15.34	2.26	13.08
Mean	9.27	1.45	7.82
Median	8.81	1.51	7.3
SD	2.43	0.35	2.08

Composition: mean of proportions q

conc(PC ae C34:1) * 0.0988 = conc(PC 15:0_18:1) [var(q)=0.0988]
Percentiles: 5%−>0.0705, 25%−>0.0843, 75%−>0.1097, 95%−>0.1373
conc(PC ae C34:1) * 0.0438 = conc(PC 17:0_16:1) [var(q)=0.0438]
Percentiles: 5%−>0.0307, 25%−>0.0371, 75%−>0.0499, 95%−>0.0601

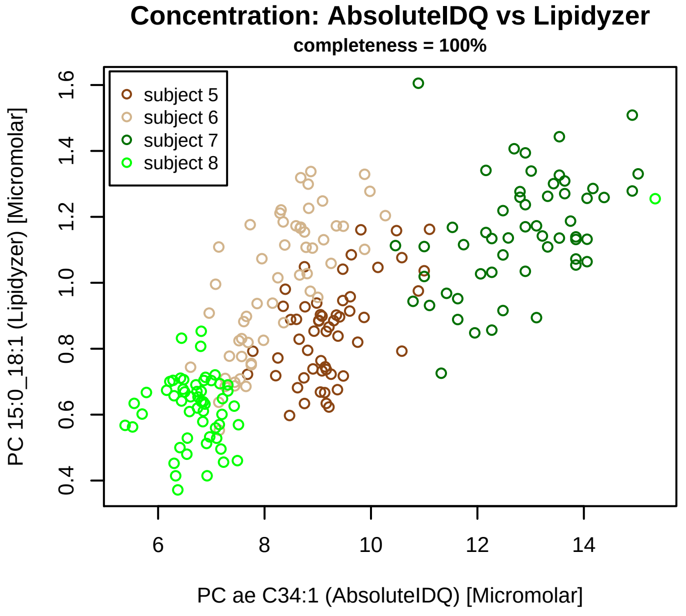

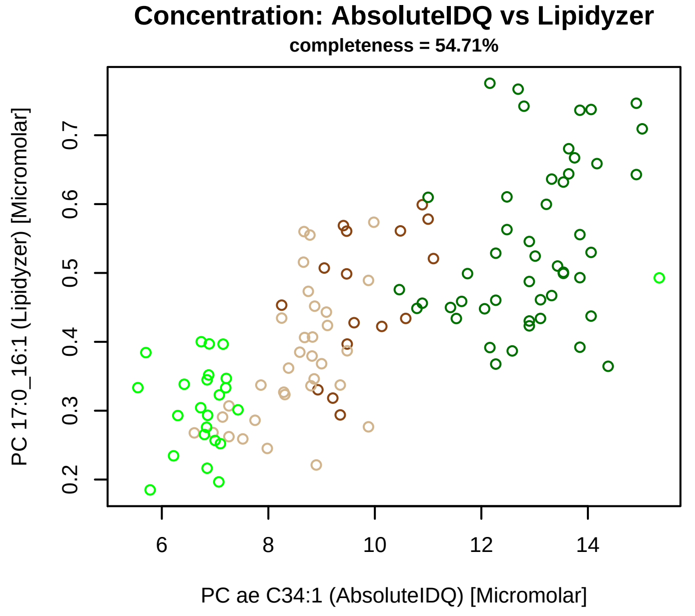

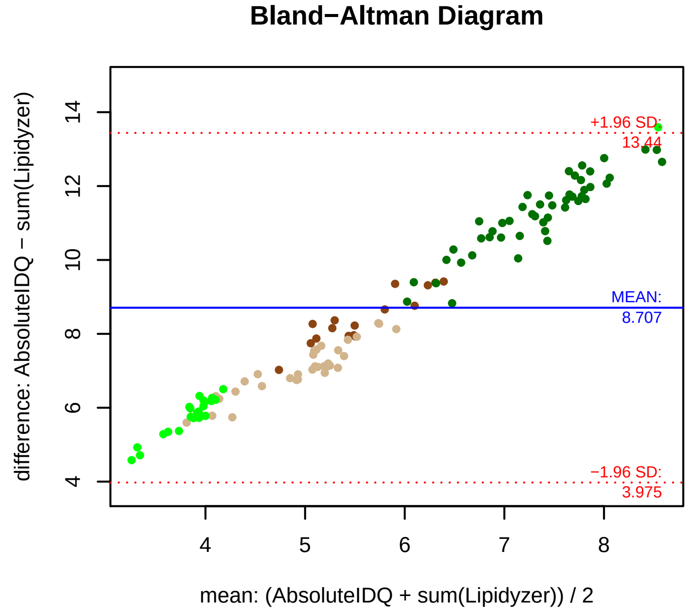

Stability of composition

(PC 15:0_18:1 + PC 17:0_16:1) / PC ae C34:1
Shapiro−Wilk Test of log quotients, pv: 0.31322; OK
SW: ok; ANOVA: Comp. ~ Subject_ID −> pv = 0.0045; DIFFERENCE
Wilcoxon: Challenge; sport: 0.5; OLTT: 1

PC 17:0_16:1 / PC ae C34:1
Shapiro−Wilk Test of log quotients, pv: 0.65757; OK
SW: ok; ANOVA: Comp. ~ Subject_ID −> pv = 0.322; EQUAL
Wilcoxon: Challenge; sport: 0.75; OLTT: 1

PC 15:0_18:1 / PC ae C34:1
Shapiro−Wilk Test of log quotients, pv: 0.37336; OK
SW: ok; ANOVA: Comp. ~ Subject_ID −> pv = 0.01068; EQUAL
Wilcoxon: Challenge; fasting: 0.25; sport: 0.625; OLTT: 0.875

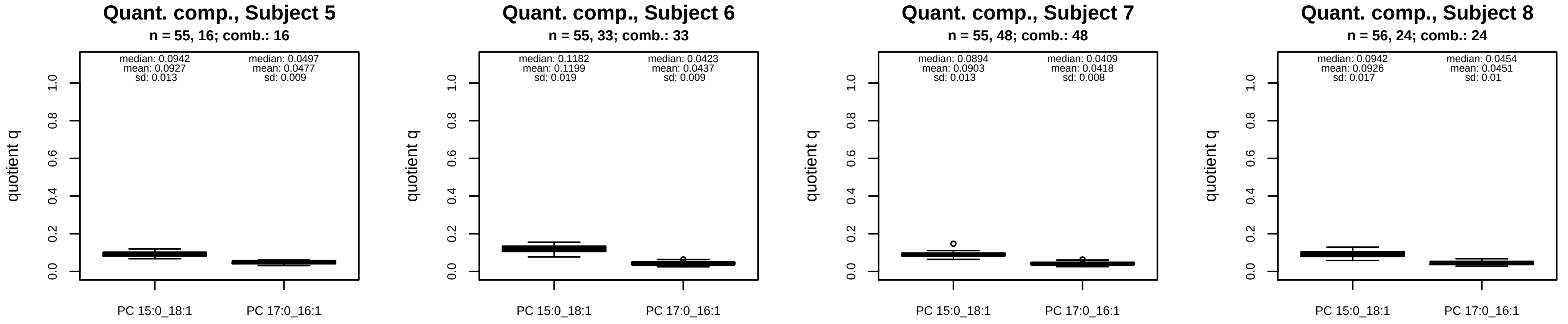

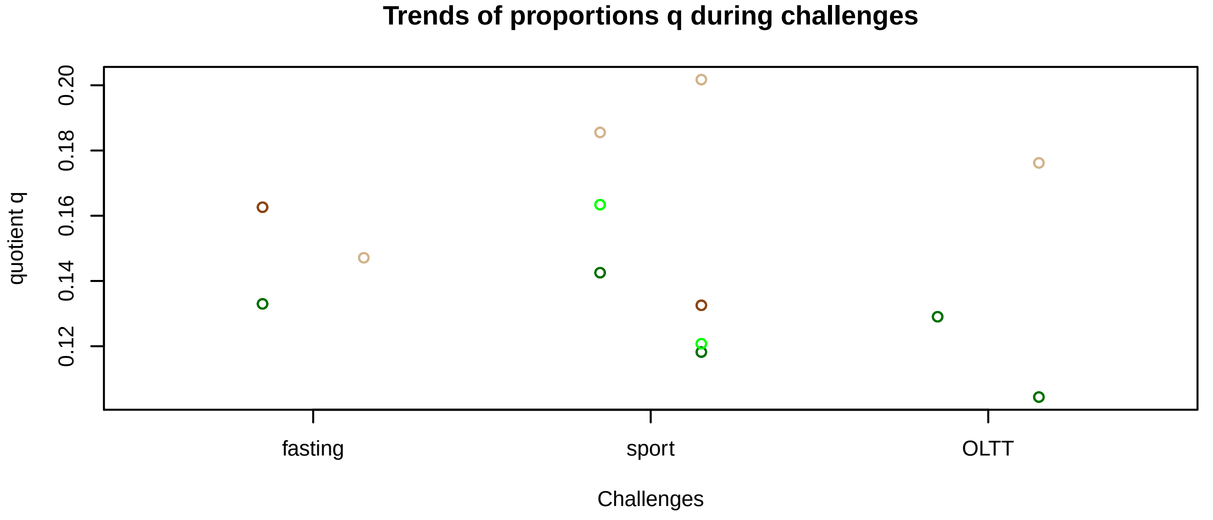

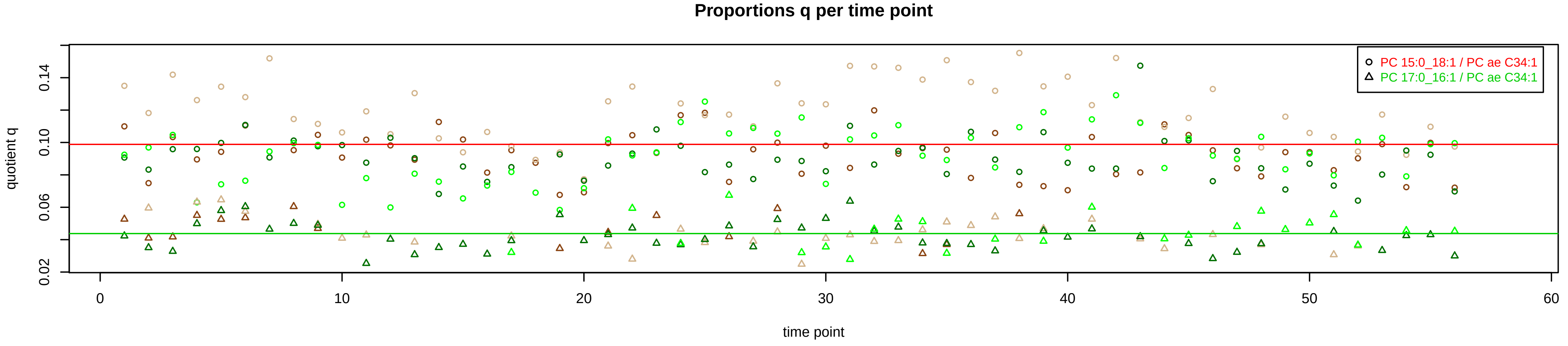

PC ae C34:2 = PC 15:0_18:2 + R

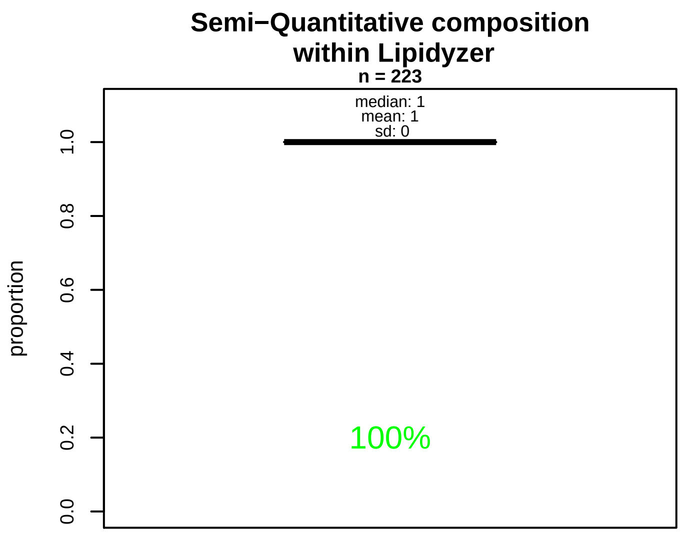

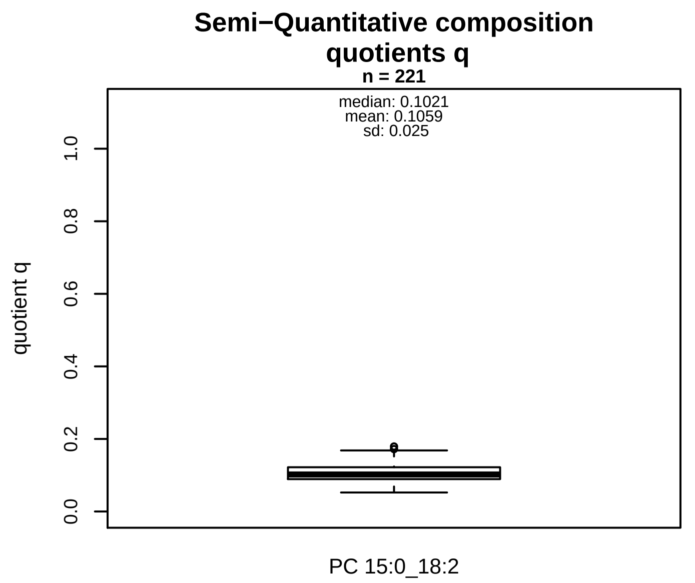

Semi−Quantitative composition: Lipidyzer

PC ae C34:2 consists of:
PC 15:0_18:2 100%
and of (not quantified):
PC O−16:0_18:2, PC O−16:1_18:1, PC O−20:1_14:1, PC 13:
PC 14:1_19:1, PC 15:1_18:1, PC 16:0_17:2, PC 16:1_17:1,
and further compounds

Composition: mean of proportions q

conc(PC ae C34:2) * 0.1059 = conc(PC 15:0_18:2) [var(q)=0.1059]
Percentiles: 5%−>0.0698, 25%−>0.0892, 75%−>0.1219, 95%−>0.1541

Linear model

```
PC ae C34:2 ~ b * ( PC 15:0_18:2 )
```

b = 3.24787

R^2 = 0.07136

Ranges

Measure	AbsoluteIDQ	sum(Lipidyzer)	delta
Min	6.73	0.63	6.1
Max	16.96	1.65	15.31
Mean	10.48	1.07	9.41
Median	10.19	1.07	9.12
SD	2.28	0.19	2.1

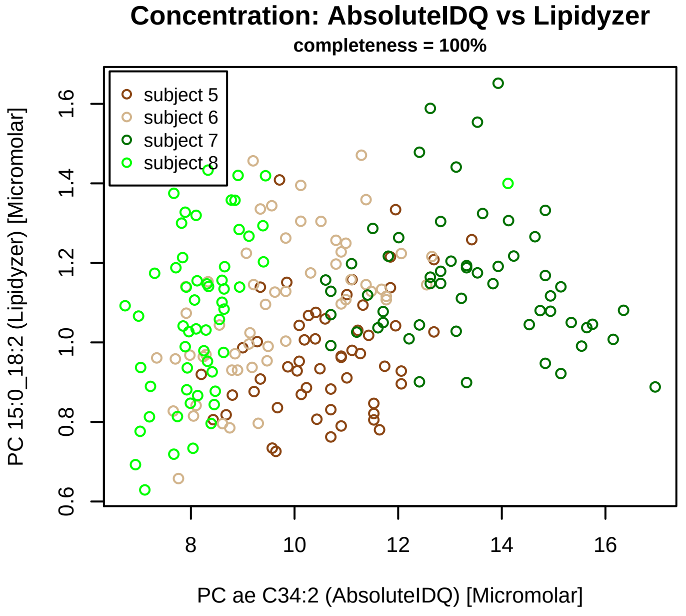

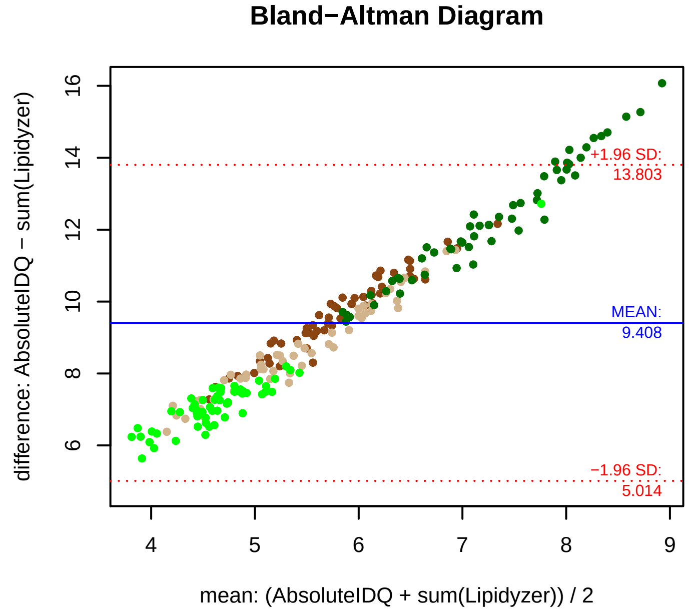

Stability of composition

PC 15:0_18:2 / PC ae C34:2
Shapiro−Wilk Test of log quotients, pv: 0.62993; OK
SW: ok; ANOVA: Comp. ~ Subject_ID −> pv = 0; DIFFERENCE
Wilcoxon: Challenge; fasting: 0.625; sport: 0.375; OLTT: 1

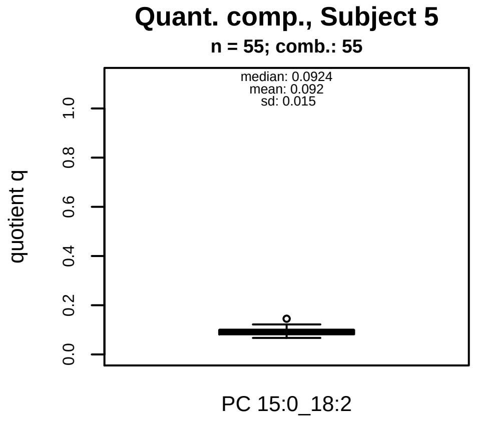

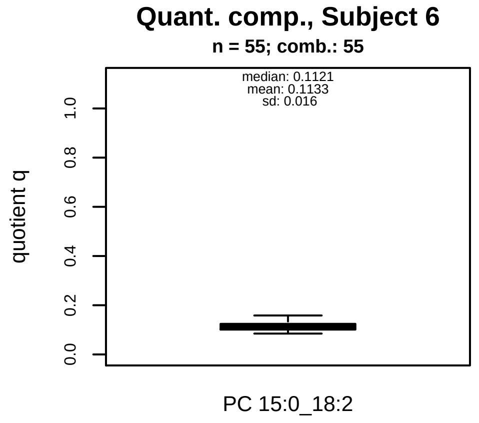

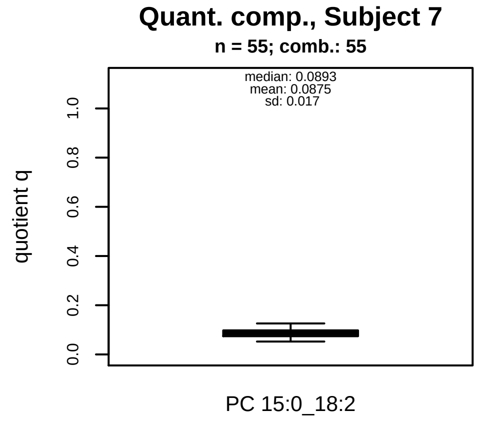

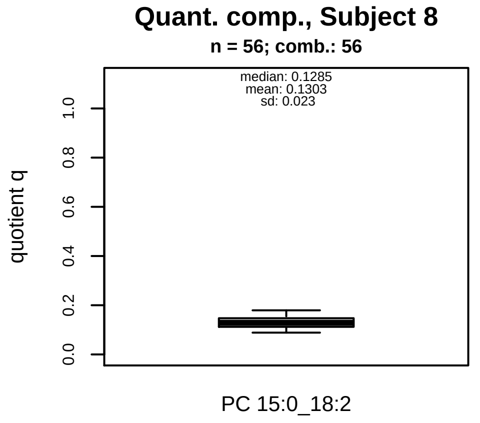

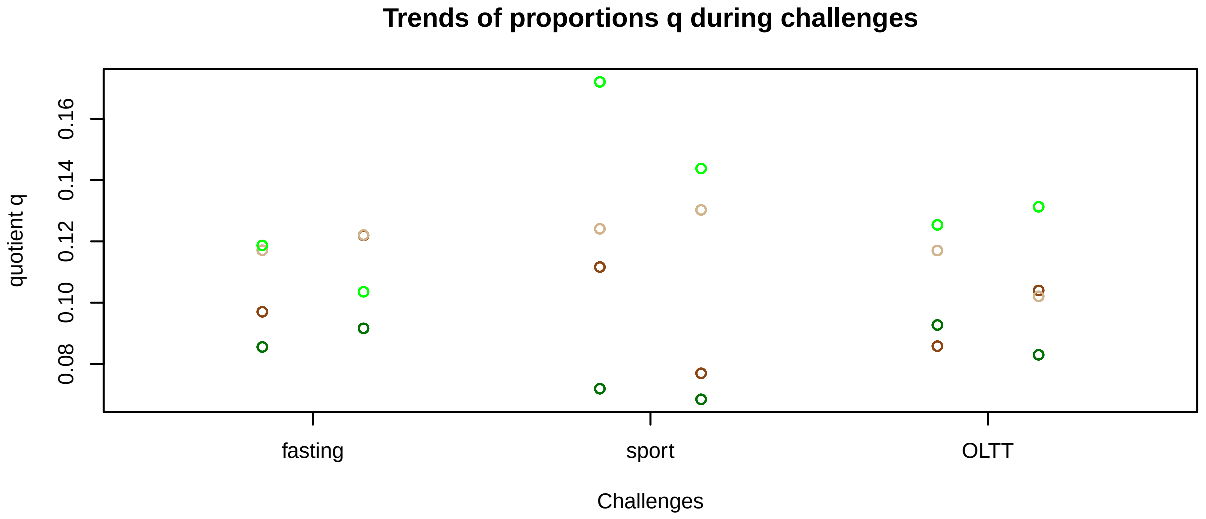

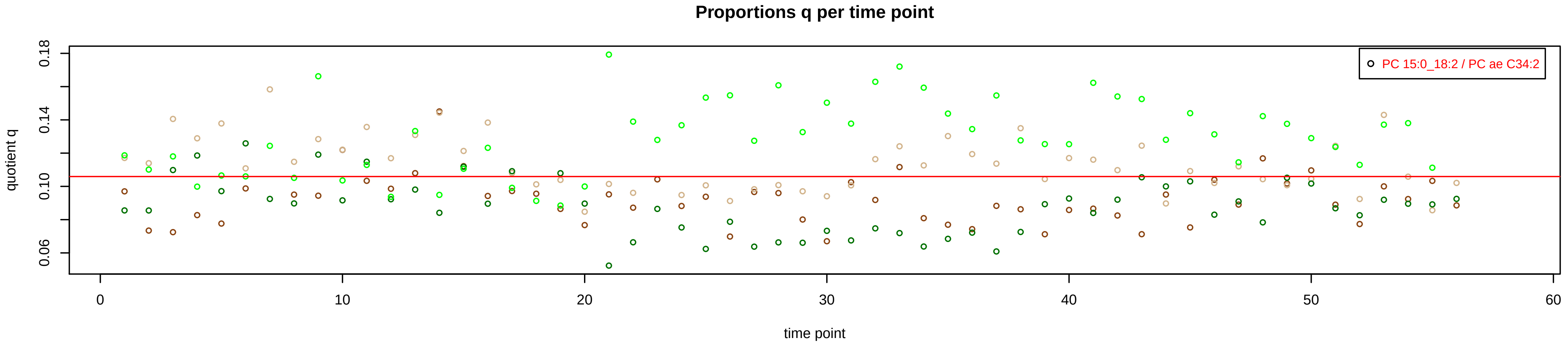

PC ae C34:3 = PC 15:0_18:3 + R

PC 15:0_18:3 excluded because of missingness > 75%

No independent Variable with coverage >0.25 out of PC 15:0_18:3

Qualitative composition

PC ae C34:3 consists of:
PC 15:0_18:3, PC O−16:0_18:3, PC O−16:1_18:2, PC 13:0_2
PC 16:1_17:2
and further compounds

PC ae C36:1 = PC 17:0_18:1 + R

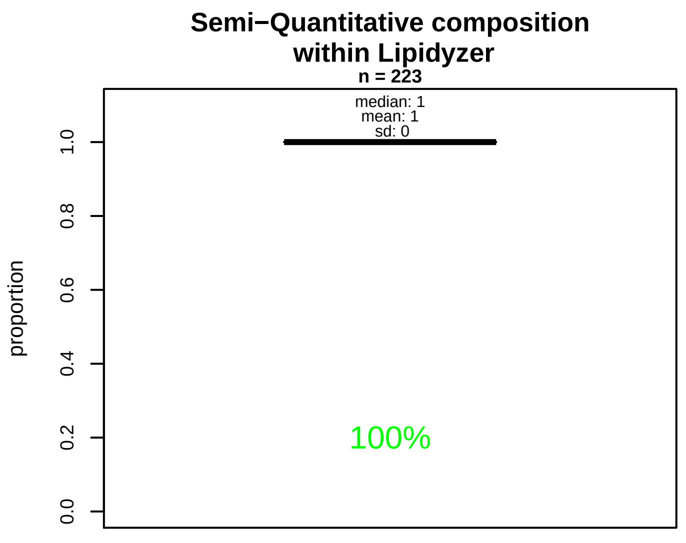

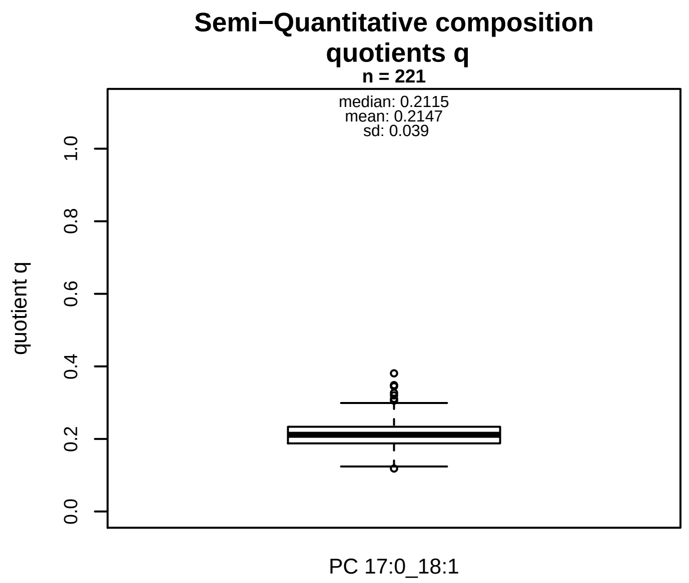

Semi−Quantitative composition: Lipidyzer

PC ae C36:1 consists of:
PC 17:0_18:1 100%
and of (not quantified):
PC O−16:0_20:1, PC O−18:0_18:1, PC O−20:0_16:1, PC O−
PC 13:0_22:1, PC 14:1_21:0, PC 15:0_20:1, PC 15:1_20:0,
PC 16:0_19:1, PC 16:1_19:0, PC 17:1_18:0, PC 18:4_18:4,
[13C1]SM 39:1
and further compounds

Composition: mean of proportions q

conc(PC ae C36:1) * 0.2147 = conc(PC 17:0_18:1) [var(q)=0.2147]
Percentiles: 5%−>0.1601, 25%−>0.1877, 75%−>0.2335, 95%−>0.2878

Linear model

```
PC ae C36:1 ~ b * ( PC 17:0_18:1 )
```

b = 4.23026

R^2 = 0.64372

Ranges

Measure	AbsoluteIDQ	sum(Lipidyzer)	delta
Min	3.28	0.65	2.63
Max	14.62	2.52	12.1
Mean	7.04	1.48	5.56
Median	6.97	1.5	5.47
SD	2.25	0.42	1.82

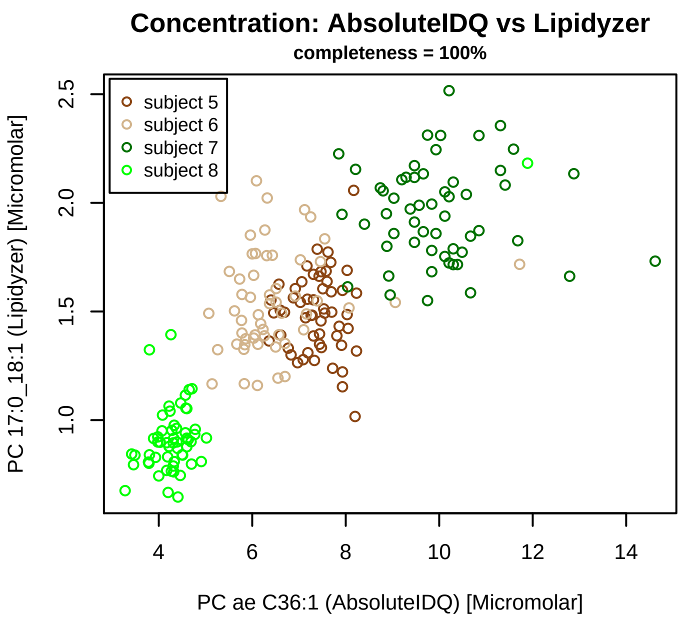

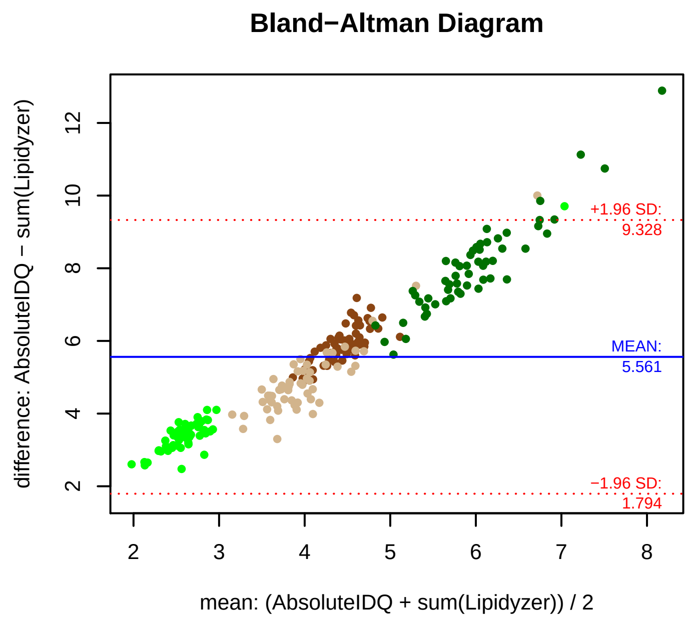

Stability of composition

PC 17:0_18:1 / PC ae C36:1
Shapiro−Wilk Test of log quotients, pv: 0.04571; OK
SW: ok; ANOVA: Comp. ~ Subject_ID −> pv = 0.5455; EQUAL
Wilcoxon: Challenge; fasting: 0.25; sport: 0.375; OLTT: 0.875

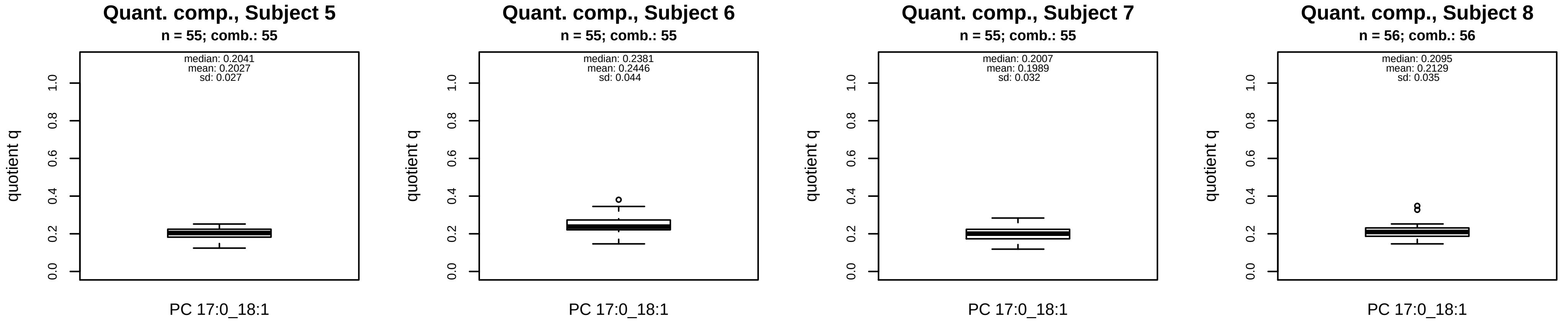

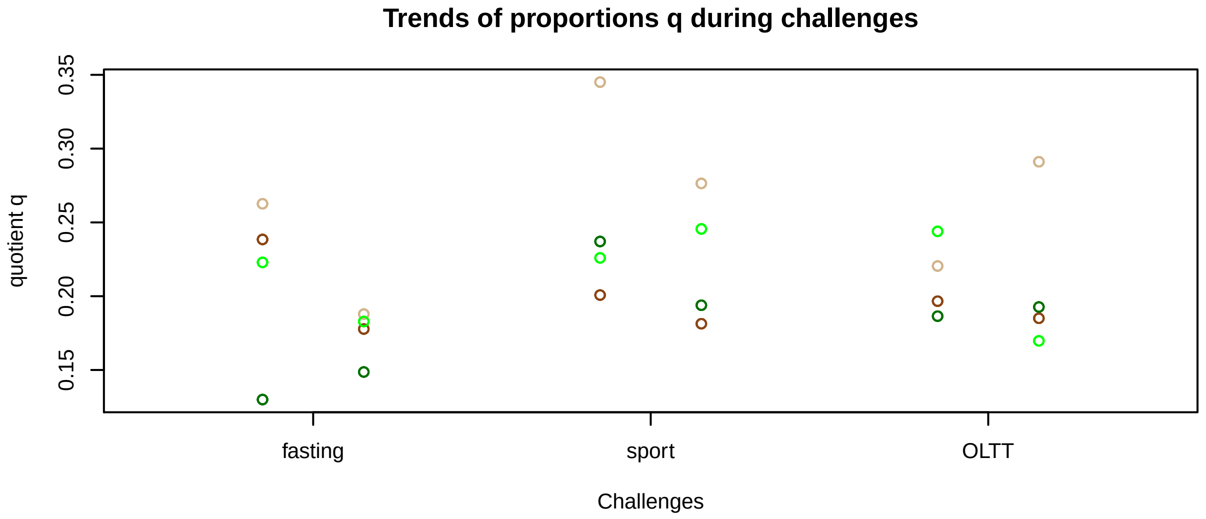

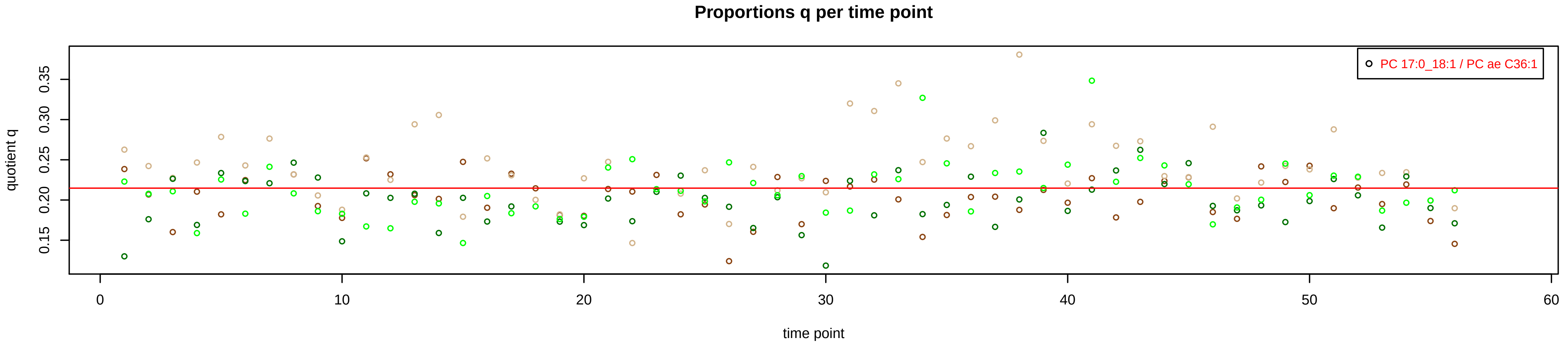

PC ae C36:2 = PC 17:0_18:2 + R

Semi−Quantitative composition within Lipidyzer

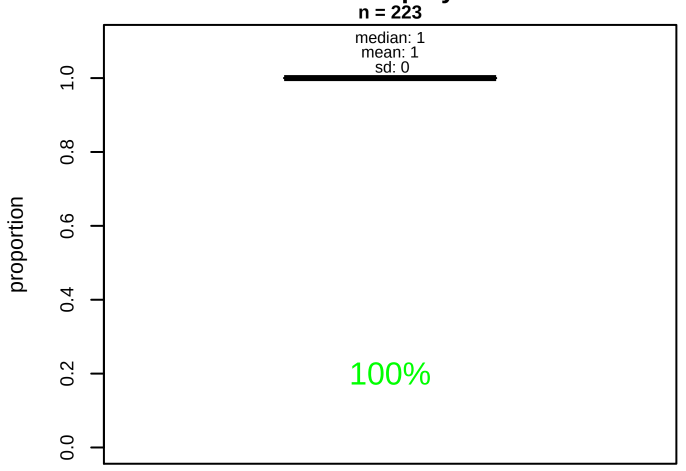

Semi−Quantitative composition quotients q

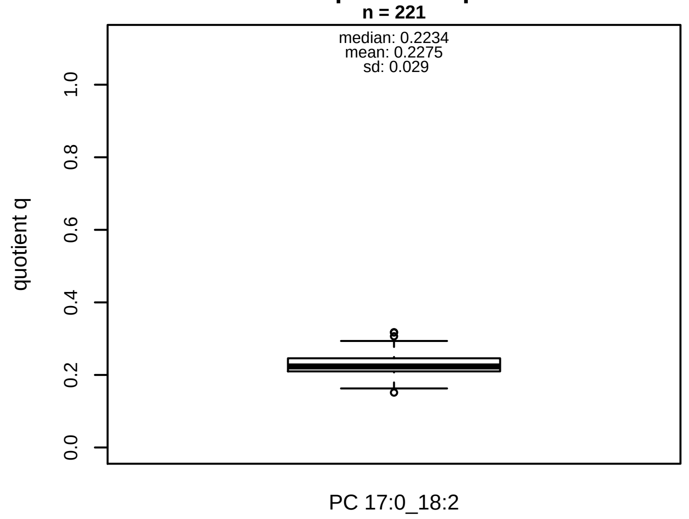

Semi−Quantitative composition: Lipidyzer

PC ae C36:2 consists of:
PC 17:0_18:2 100%
and of (not quantified):
PC O−16:0_20:2, PC O−18:0_18:2, PC O−18:1_18:1, PC O−
PC 13:0_22:2, PC 15:0_20:2, PC 15:1_20:1, PC 16:1_19:1,
[13C1]SM 39:2
and further compounds

Composition: mean of proportions q

conc(PC ae C36:2) * 0.2275 = conc(PC 17:0_18:2) [var(q)=0.2275]
Percentiles: 5%−>0.1837, 25%−>0.2098, 75%−>0.2459, 95%−>0.2799

Linear model

```
PC ae C36:2 ~ b * ( PC 17:0_18:2 )
```

b = 3.27954

R^2 = 0.52816

Ranges

Measure	AbsoluteIDQ	sum(Lipidyzer)	delta
Min	9.2	1.76	7.44
Max	19.24	4.41	14.83
Mean	13.38	3.02	10.36
Median	13.03	2.96	10.07
SD	2.27	0.51	1.76

Concentration: AbsoluteIDQ vs Lipidyzer

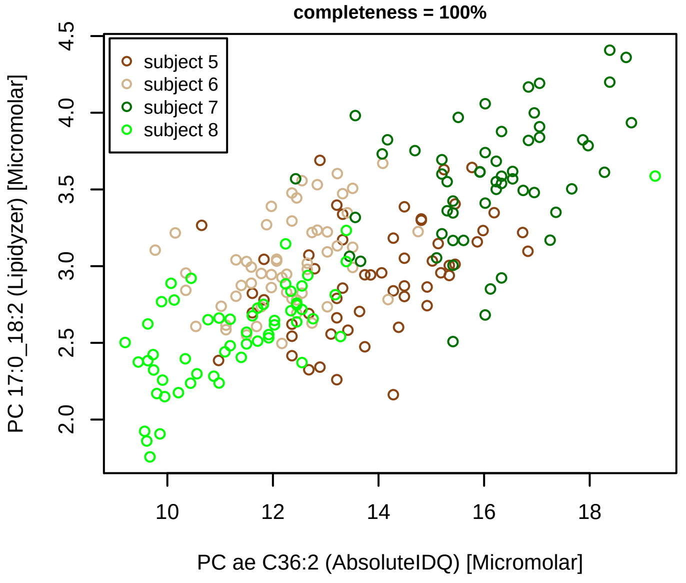

Bland−Altman Diagram

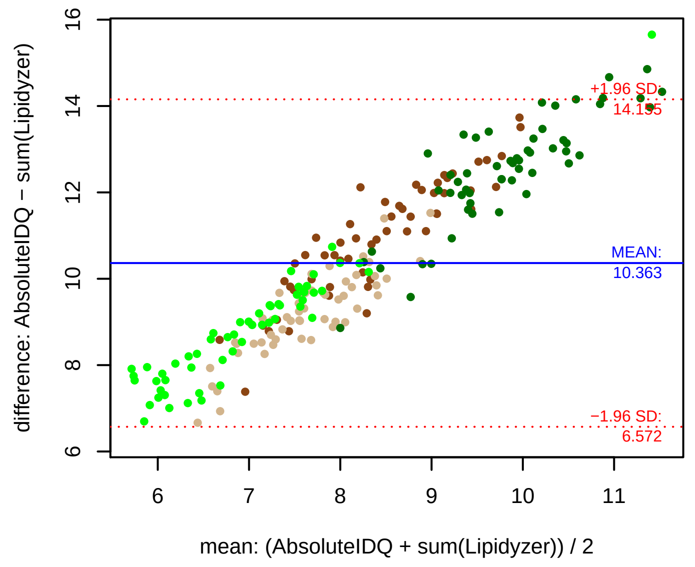

Stability of composition

PC 17:0_18:2 / PC ae C36:2
Shapiro−Wilk Test of log quotients, pv: 0.867; OK
SW: ok; ANOVA: Comp. ~ Subject_ID −> pv = 0.19747; EQUAL
Wilcoxon: Challenge; fasting: 0.625; sport: 0.375; OLTT: 0.125

Quant. comp., Subject 5

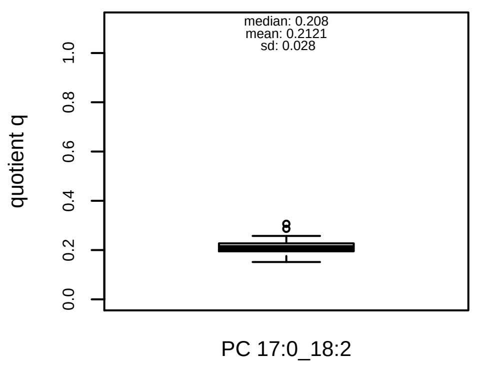

Quant. comp., Subject 6

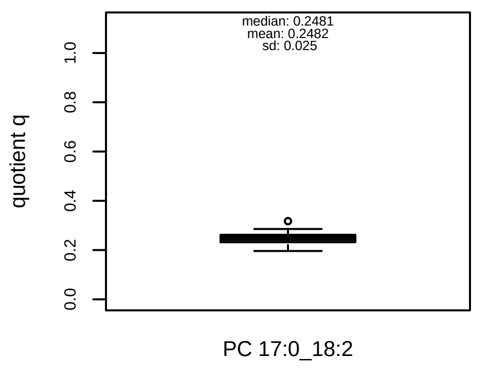

Quant. comp., Subject 7

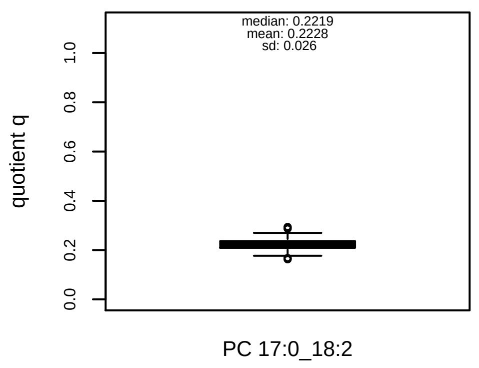

Quant. comp., Subject 8

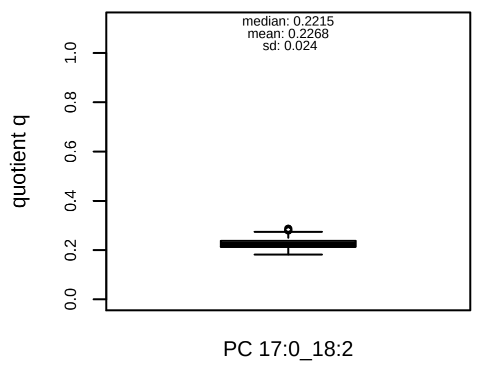

Trends of proportions q during challenges

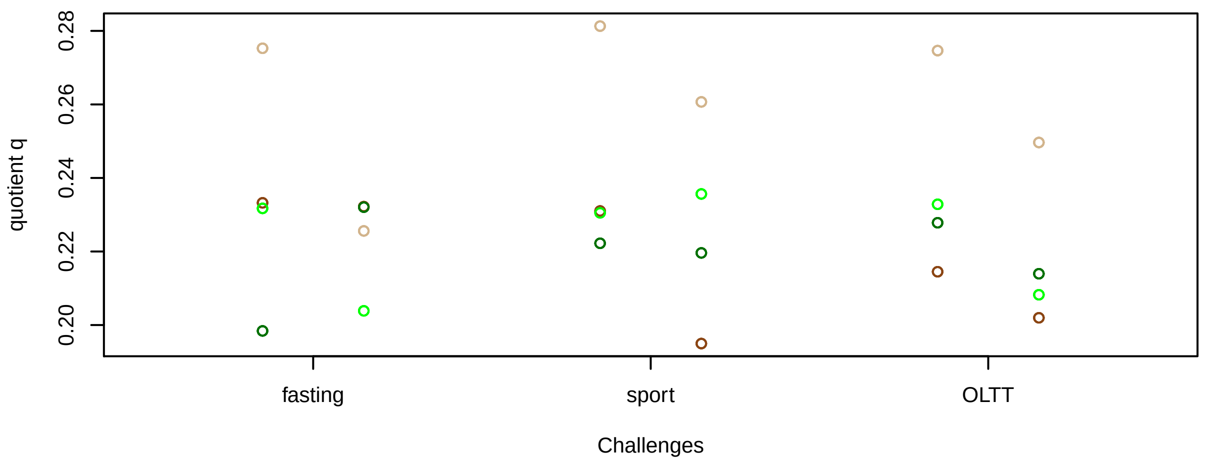

Proportions q per time point

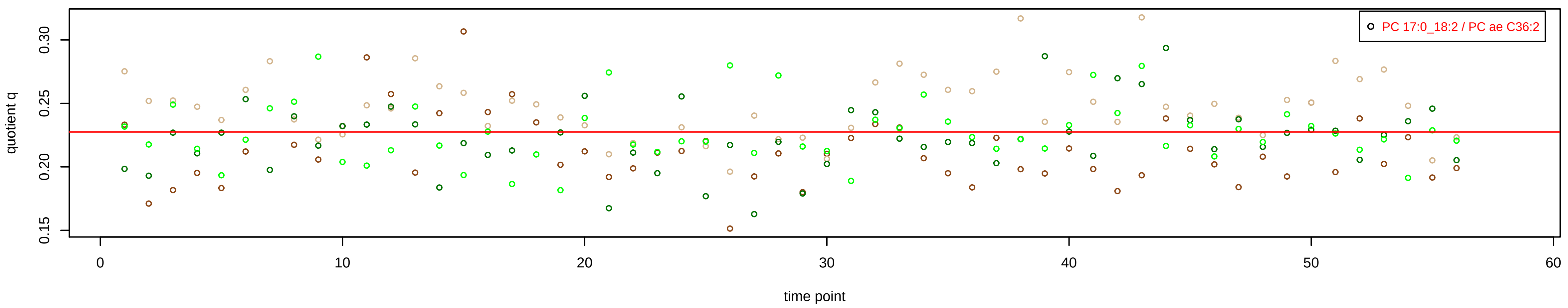

PC ae C36:3 = PC 15:0_20:3 + PC 17:0_18:3 + R

PC 17:0_18:3 excluded because of missingness > 75%

Semi−Quantitative composition within Lipidyzer

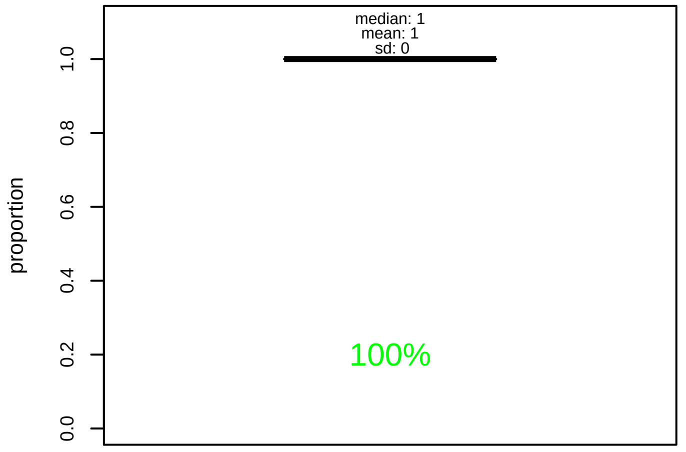

Semi−Quantitative composition quotients q

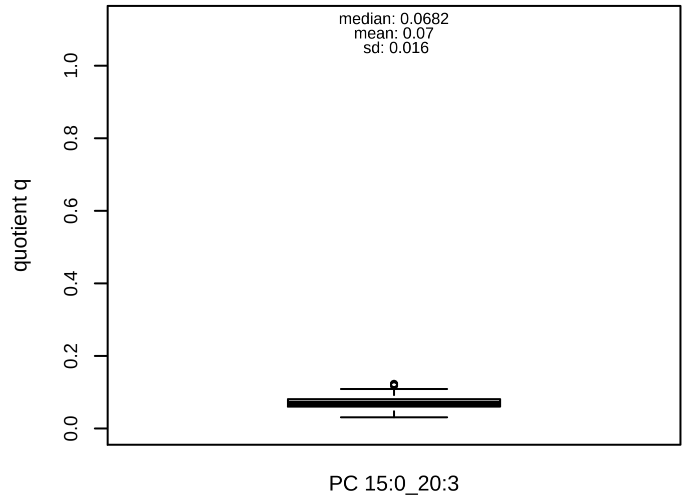

Semi−Quantitative composition: Lipidyzer

PC ae C36:3 consists of:
PC 15:0_20:3 100%
and of (not quantified):
PC 17:0_18:3, PC O−16:0_20:3, PC O−18:0_18:3, PC O−18:
PC O−16:1_20:2, PC 15:1_20:2, PC 17:1_18:2, PC 17:2_18::
and further compounds

Composition: mean of proportions q

conc(PC ae C36:3) * 0.07 = conc(PC 15:0_20:3) [var(q)=0.07]
Percentiles: 5%−>0.0458, 25%−>0.0603, 75%−>0.0809, 95%−>0.095

Linear model

```
PC ae C36:3 ~ b * ( PC 15:0_20:3 )
```

b = 8.36338

R^2 = 0.49946

Ranges

Measure	AbsoluteIDQ	sum(Lipidyzer)	delta
Min	4.88	0.19	4.69
Max	13.58	1.29	12.29
Mean	7.83	0.55	7.28
Median	7.65	0.54	7.11
SD	1.97	0.17	1.8

Concentration: AbsoluteIDQ vs Lipidyzer

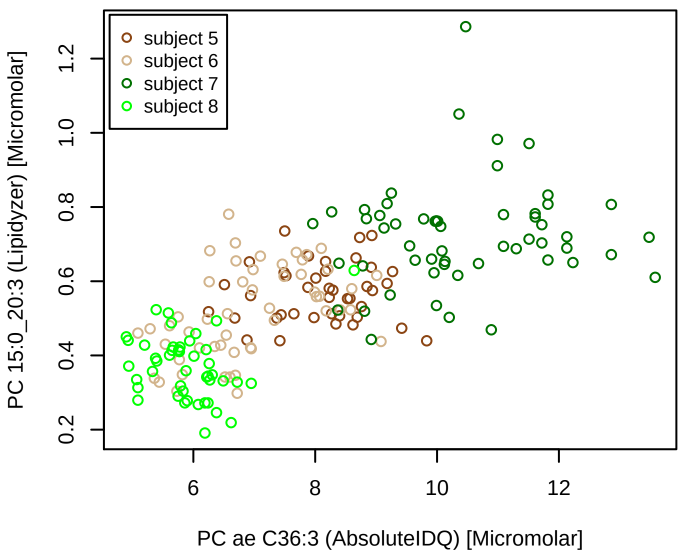

Bland−Altman Diagram

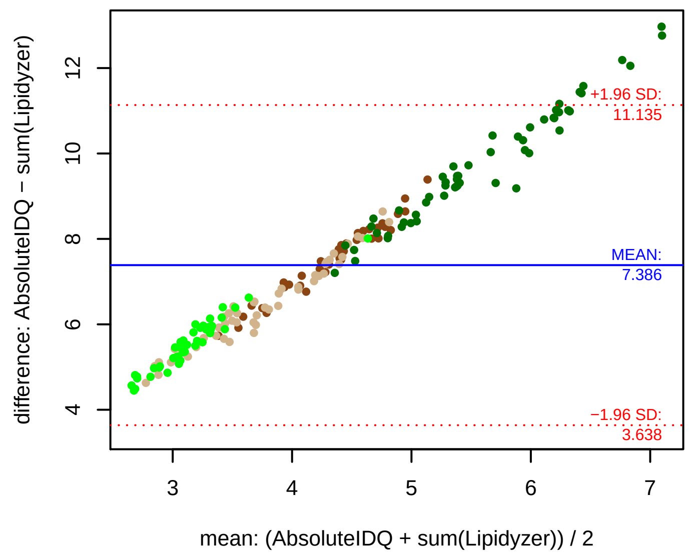

Stability of composition

PC 15:0_20:3 / PC ae C36:3
Shapiro−Wilk Test of log quotients, pv: 0.11199; OK
SW: ok; ANOVA: Comp. ~ Subject_ID −> pv = 0.00182; DIFFERENCE
Wilcoxon: Challenge; fasting: 0.25; sport: 0.75; OLTT: 0.625

Quant. comp., Subject 5

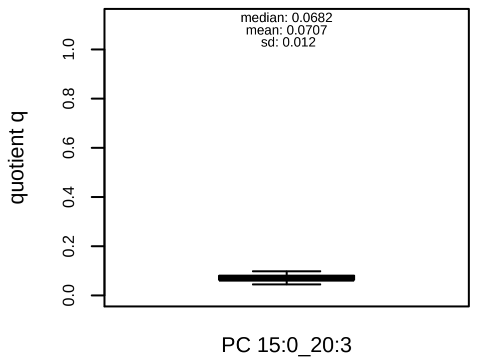

Quant. comp., Subject 6

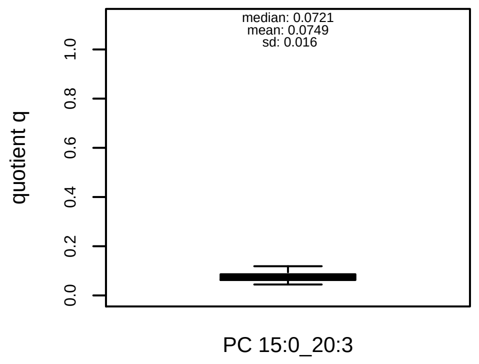

Quant. comp., Subject 7

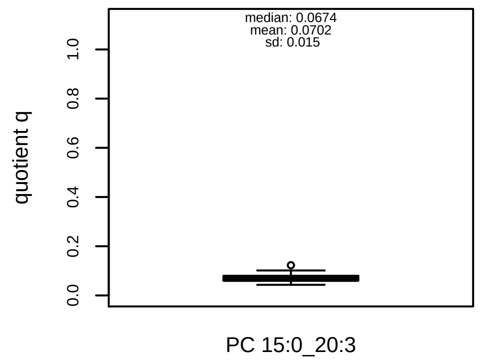

Quant. comp., Subject 8

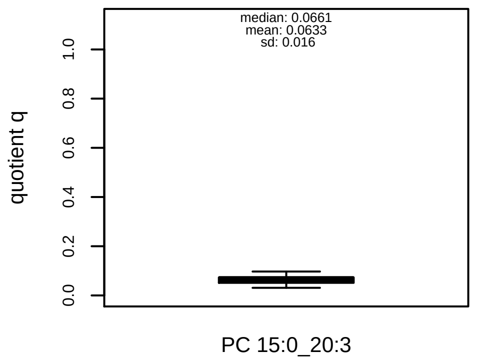

Trends of proportions q during challenges

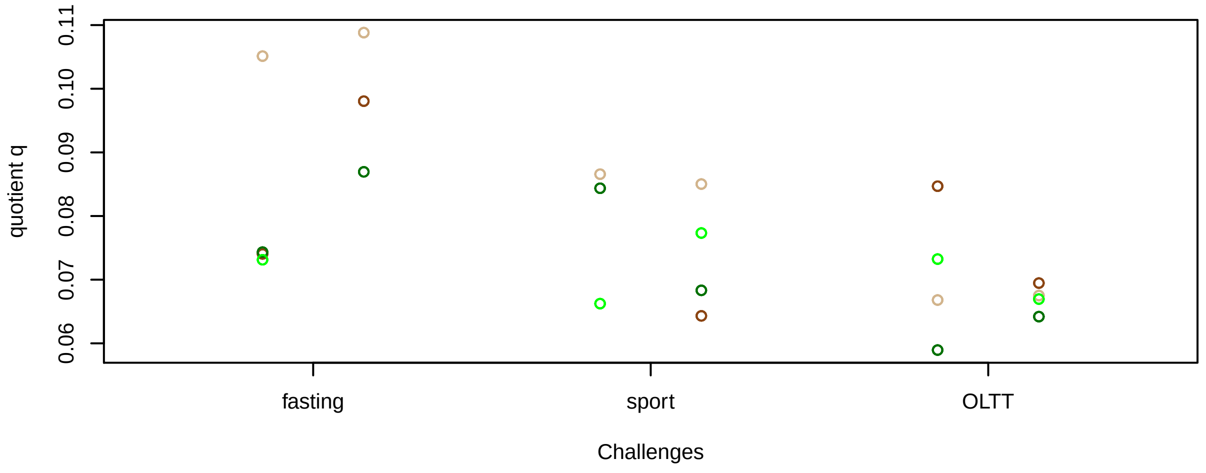

Proportions q per time point

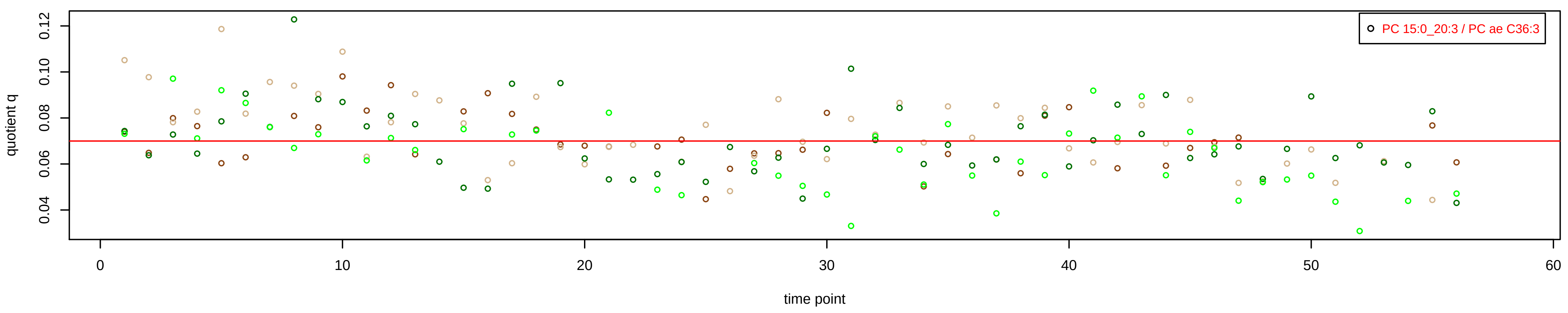

PC ae C36:4 = PC 15:0_20:4 + R

Semi−Quantitative composition within Lipidyzer

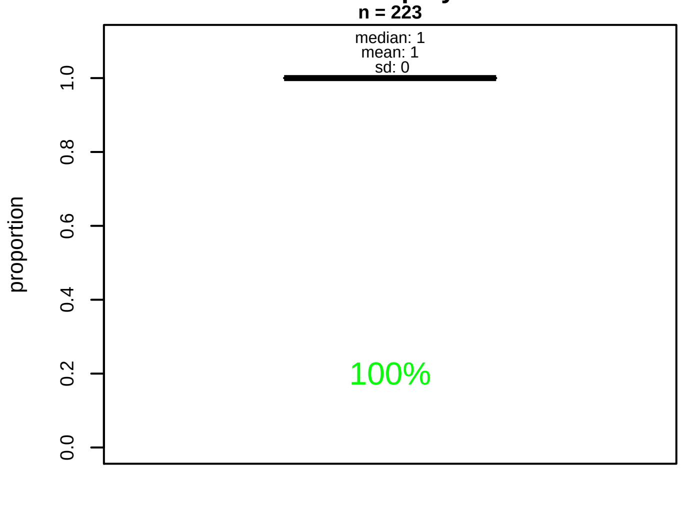

Semi−Quantitative composition quotients q

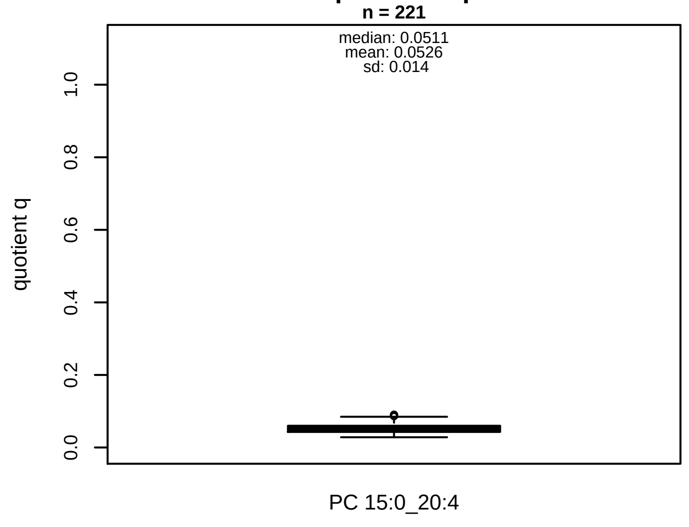

Semi−Quantitative composition: Lipidyzer

PC ae C36:4 consists of:
PC 15:0_20:4 100%
and of (not quantified):
PC O−16:0_20:4, PC O−16:1_20:3, PC O−18:0_18:4, PC O−
PC O−18:2_18:2, PC 13:0_22:4, PC 15:1_20:3, PC 17:0_18:
PC 17:2_18:2
and further compounds

Composition: mean of proportions q

conc(PC ae C36:4) * 0.0526 = conc(PC 15:0_20:4) [var(q)=0.0526]
Percentiles: 5%−>0.0339, 25%−>0.0429, 75%−>0.0599, 95%−>0.0793

Linear model

```
PC ae C36:4 ~ b * ( PC 15:0_20:4 )
```

b = 11.15708

R^2 = 0.25993

Ranges

Measure	AbsoluteIDQ	sum(Lipidyzer)	delta
Min	8.59	0.45	8.14
Max	26.01	1.8	24.21
Mean	17.54	0.88	16.66
Median	18.55	0.86	17.69
SD	4.63	0.21	4.42

Concentration: AbsoluteIDQ vs Lipidyzer

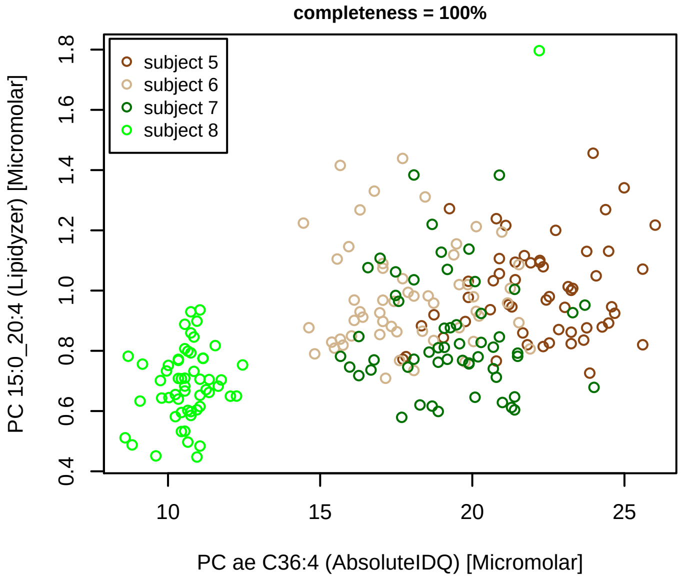

Bland−Altman Diagram

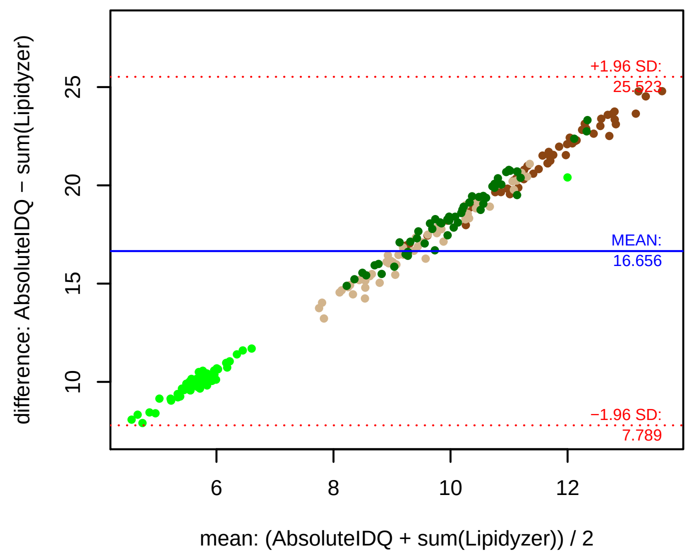

Stability of composition

PC 15:0_20:4 / PC ae C36:4
Shapiro−Wilk Test of log quotients, pv: 0.32439; OK
SW: ok; ANOVA: Comp. ~ Subject_ID −> pv = 0; DIFFERENCE
Wilcoxon: Challenge; fasting: 1; sport: 0.875; OLTT: 0.25

Quant. comp., Subject 5

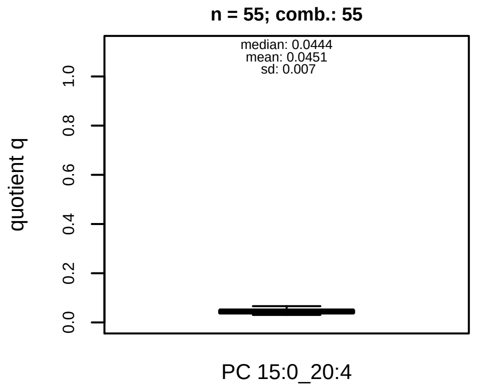

Quant. comp., Subject 6

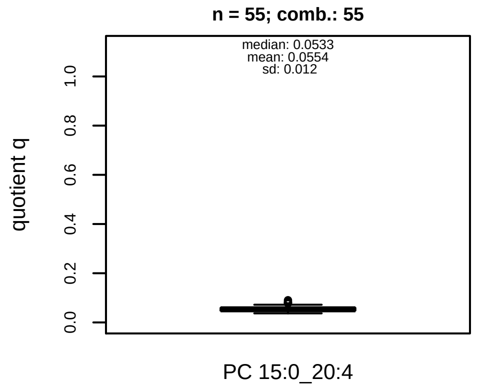

Quant. comp., Subject 7

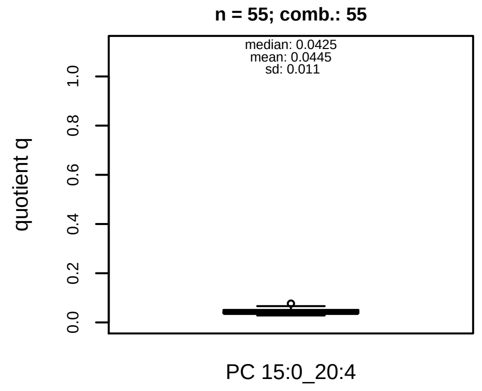

Quant. comp., Subject 8

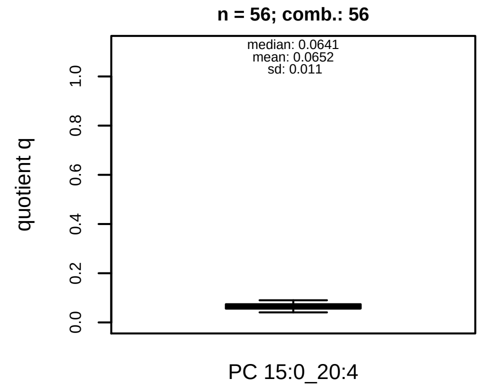

Trends of proportions q during challenges

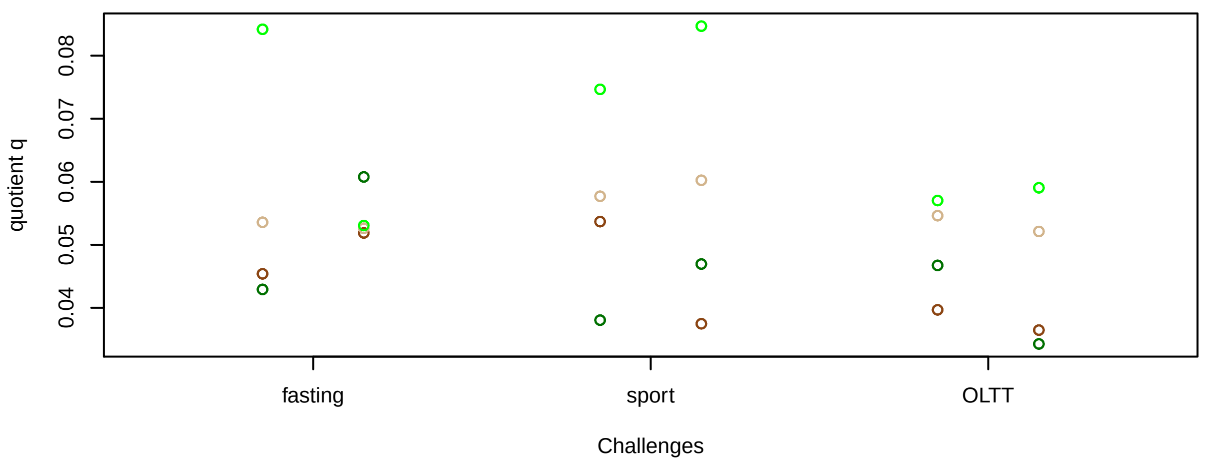

Proportions q per time point

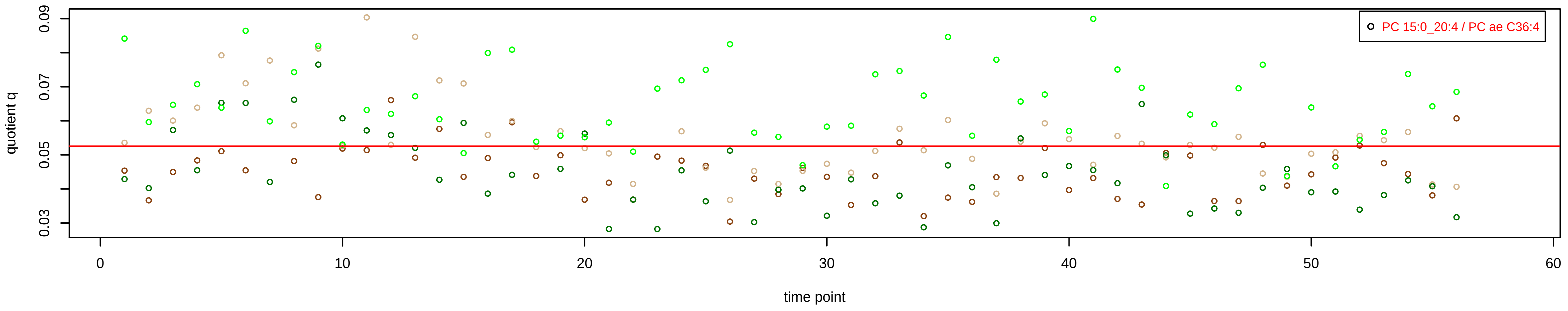

PC ae C36:5 = PC 15:0_20:5 + R

PC 15:0_20:5 excluded because of missingness > 75%

No independent Variable with coverage >0.25 out of PC 15:0_20:5

Qualitative composition

PC ae C36:5 consists of:
PC 15:0_20:5, PC O−16:0_20:5, PC O−16:1_20:4, PC O−18:
PC O−18:2_18:3, PC 15:1_20:4, PC 17:1_18:4, PC 17:2_18:3
and further compounds

PC ae C38:0 = PC 18:2_20:5 + R

PC 18:2_20:5 excluded because of missingness > 75%

No independent Variable with coverage >0.25 out of PC 18:2_20:5

Qualitative composition

PC ae C38:0 consists of:
PC 18:2_20:5, PC O−16:0_22:0, PC O−18:0_20:0, PC 15:0_2
PC 16:0_21:0, PC 17:0_20:0, PC 18:0_19:0, PC 16:1_22:6,
PC 18:4_20:3
and further compounds

PC ae C38:2 = R

No independent Variable measured.

Qualitative composition

PC ae C38:2 consists of:
PC O−16:0_22:2, PC O−18:0_20:2, PC O−18:1_20:1, PC O−
PC O−16:1_22:1, PC 15:0_22:2, PC 15:1_22:1, PC 17:0_20:2
PC 17:1_20:1, PC 17:2_20:0, PC 18:1_19:1, PC 18:2_19:0,
PC 18:4_20:5
and further compounds

PC ae C38:3 = PC 17:0_20:3 + R

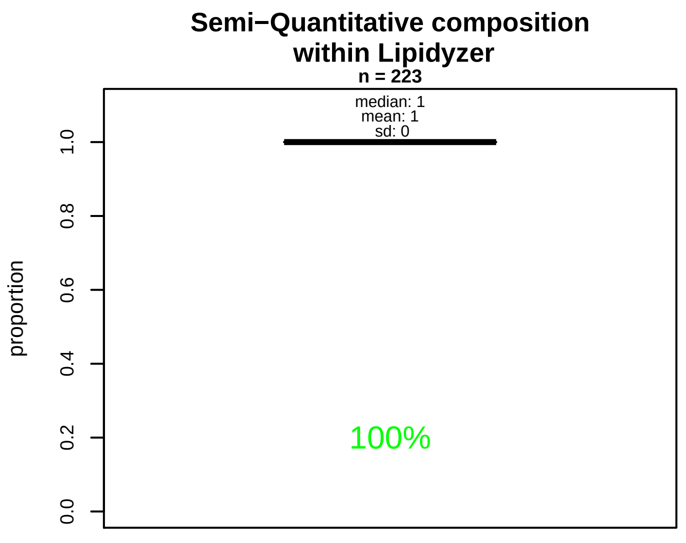

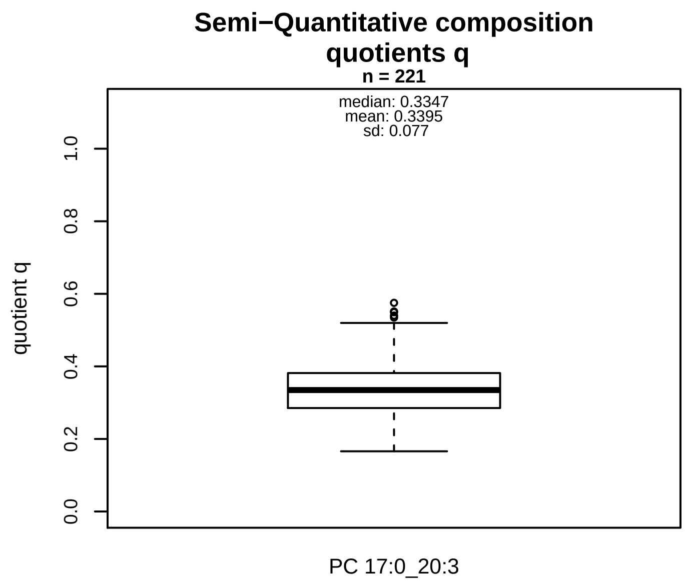

Semi−Quantitative composition: Lipidyzer

PC ae C38:3 consists of:
PC 17:0_20:3 100%
and of (not quantified):
PC O−18:0_20:3, PC O−16:1_22:2, PC O−18:1_20:2, PC O−
PC O−18:3_20:0, PC 15:1_22:2, PC 17:1_20:2, PC 17:2_20:
PC 18:3_19:0
and further compounds

Composition: mean of proportions q

conc(PC ae C38:3) * 0.3395 = conc(PC 17:0_20:3) [var(q)=0.3395]
Percentiles: 5%−>0.2248, 25%−>0.2852, 75%−>0.3816, 95%−>0.4767

Linear model

```
PC ae C38:3 ~ b * ( PC 17:0_20:3 )
```

b = 1.66896

R^2 = 0.64485

Ranges

Measure	AbsoluteIDQ	sum(Lipidyzer)	delta
Min	2	0.43	1.57
Max	6.15	2.75	3.4
Mean	3.68	1.27	2.41
Median	3.73	1.27	2.46
SD	0.97	0.47	0.5

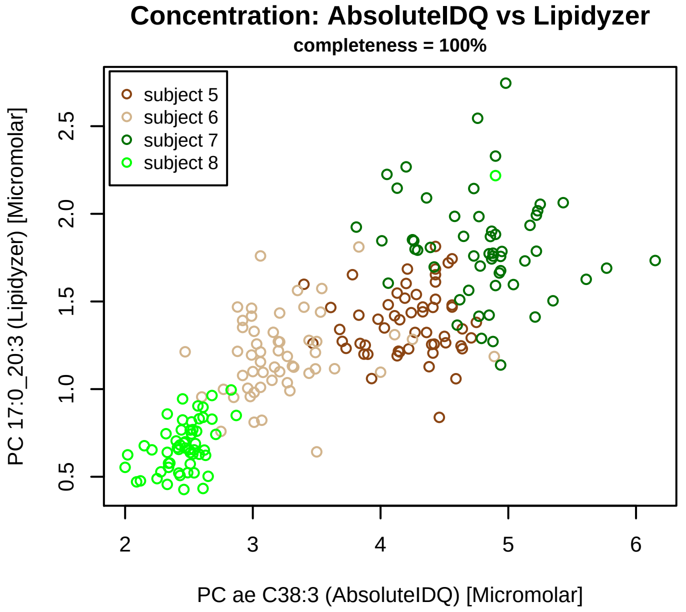

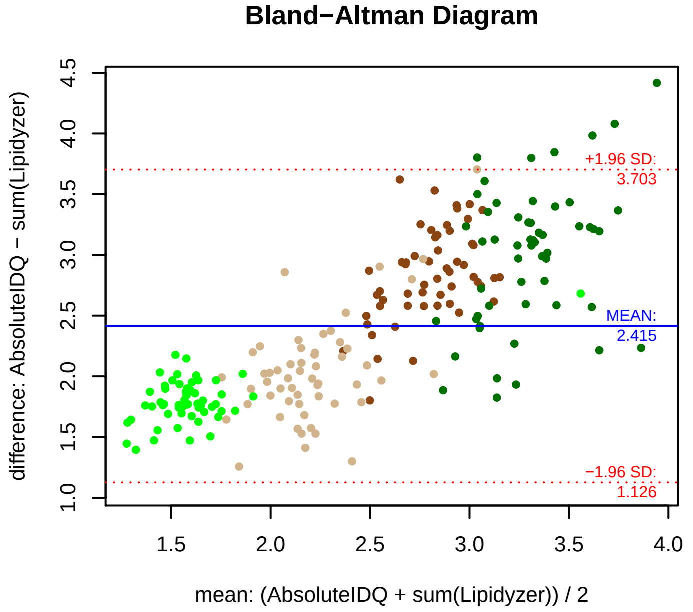

Stability of composition

PC 17:0_20:3 / PC ae C38:3
Shapiro−Wilk Test of log quotients, pv: 0.30664; OK
SW: ok; ANOVA: Comp. ~ Subject_ID −> pv = 0.00011; DIFFERENCE
Wilcoxon: Challenge; fasting: 0.875; sport: 0.875; OLTT: 0.125

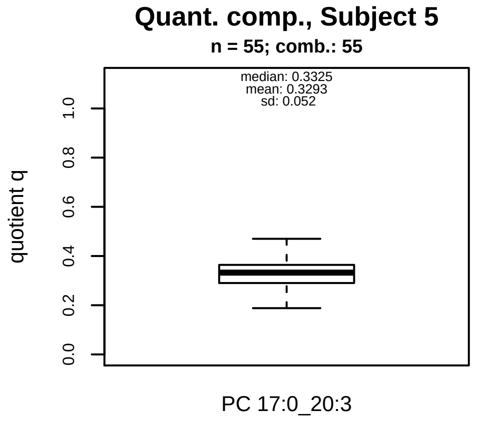

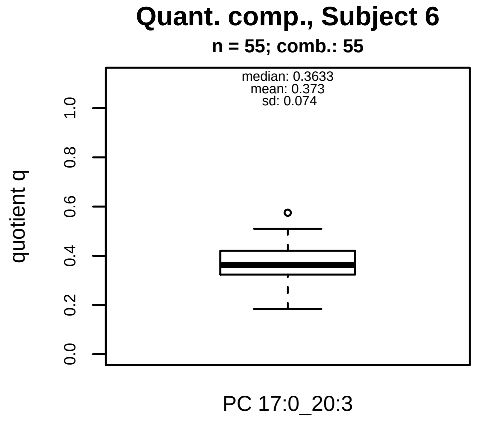

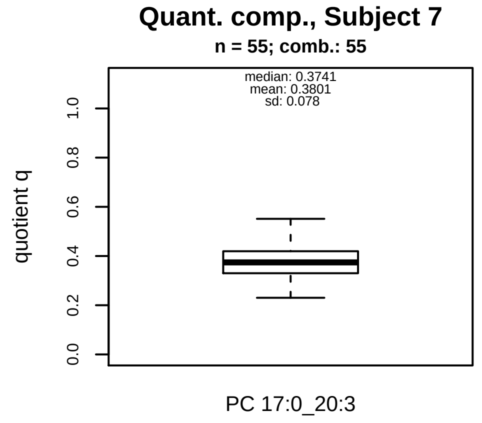

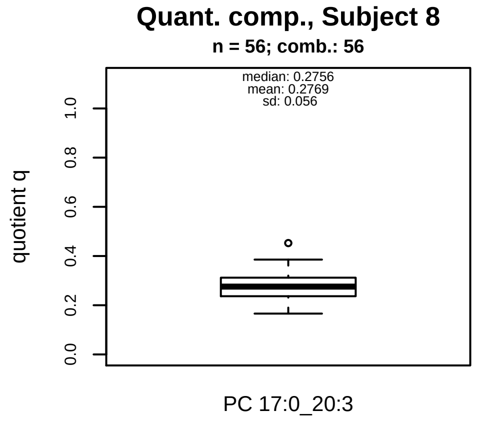

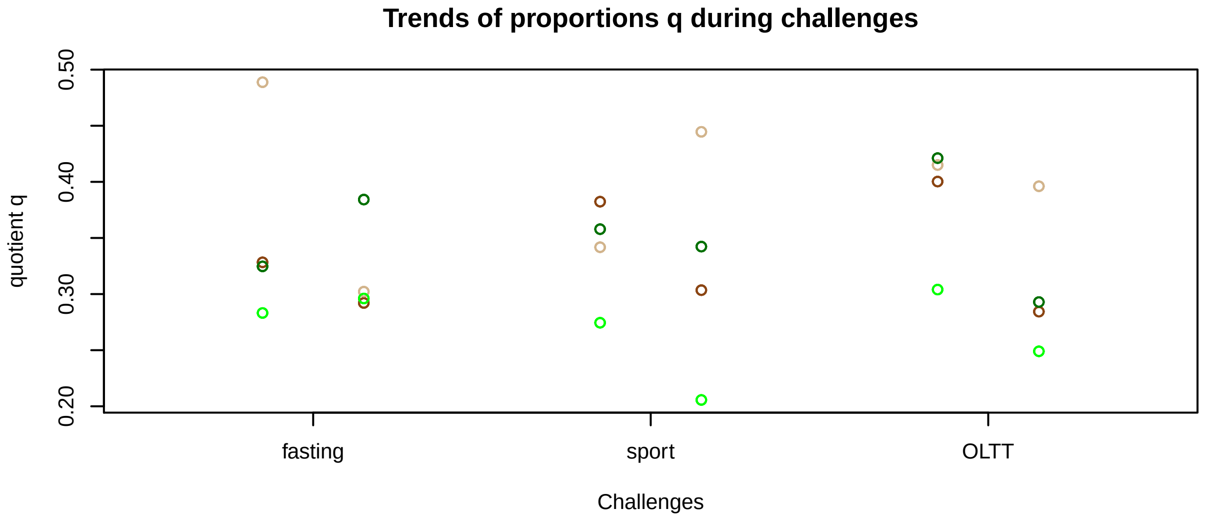

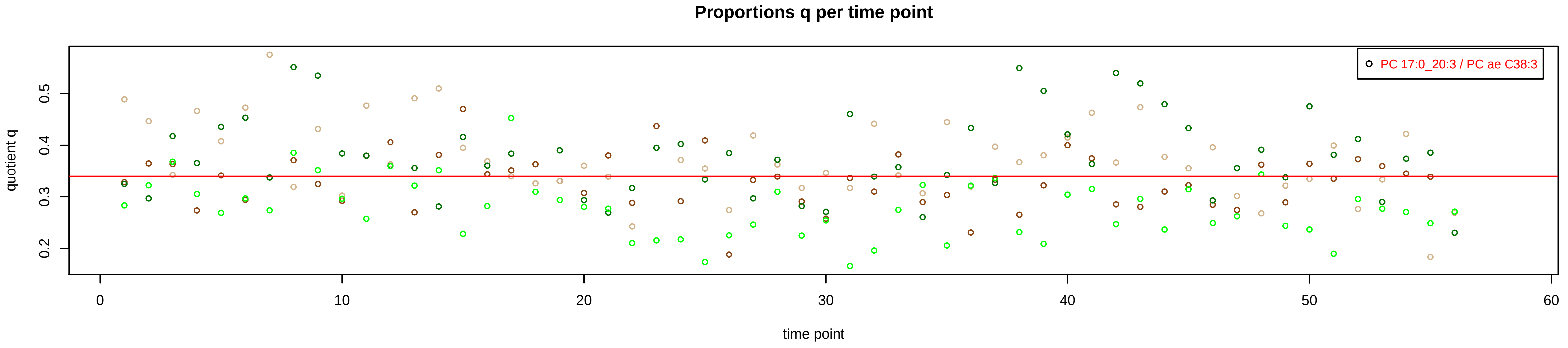

PC ae C38:4 = PC 17:0_20:4 + R

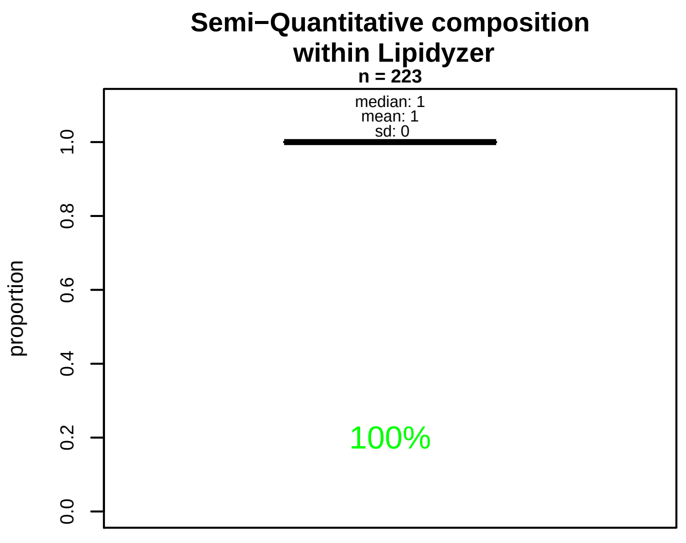

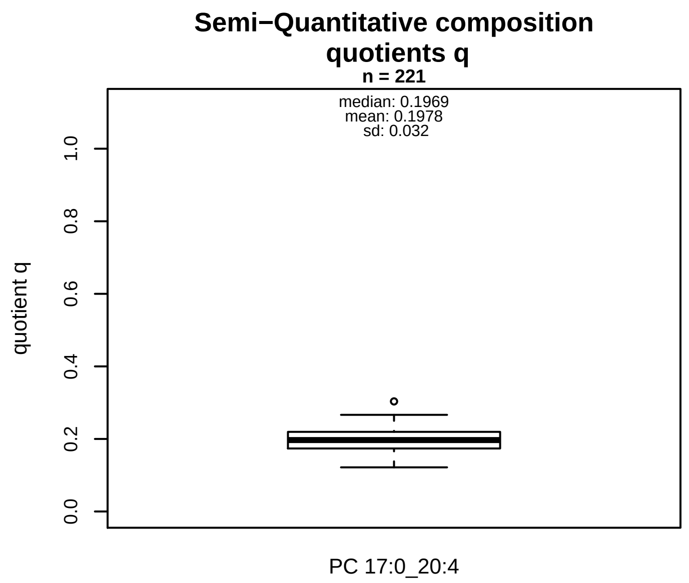

Semi−Quantitative composition: Lipidyzer

PC ae C38:4 consists of:
PC 17:0_20:4 100%
and of (not quantified):
PC O−16:0_22:4, PC O−18:0_20:4, PC O−20:0_18:4, PC O−
PC O−18:2_20:2, PC O−20:1_18:3, PC 15:0_22:4, PC 17:1_2
PC 18:4_19:0
and further compounds

Composition: mean of proportions q

conc(PC ae C38:4) * 0.1978 = conc(PC 17:0_20:4) [var(q)=0.1978]
Percentiles: 5%−>0.1495, 25%−>0.1737, 75%−>0.2195, 95%−>0.2547

Linear model

```
PC ae C38:4 ~ b * ( PC 17:0_20:4 )
```

b = 3.91691

R^2 = 0.64147

Ranges

Measure	AbsoluteIDQ	sum(Lipidyzer)	delta
Min	6.25	1.05	5.2
Max	21	4.38	16.62
Mean	13.93	2.73	11.2
Median	14.55	2.81	11.74
SD	3.65	0.75	2.91

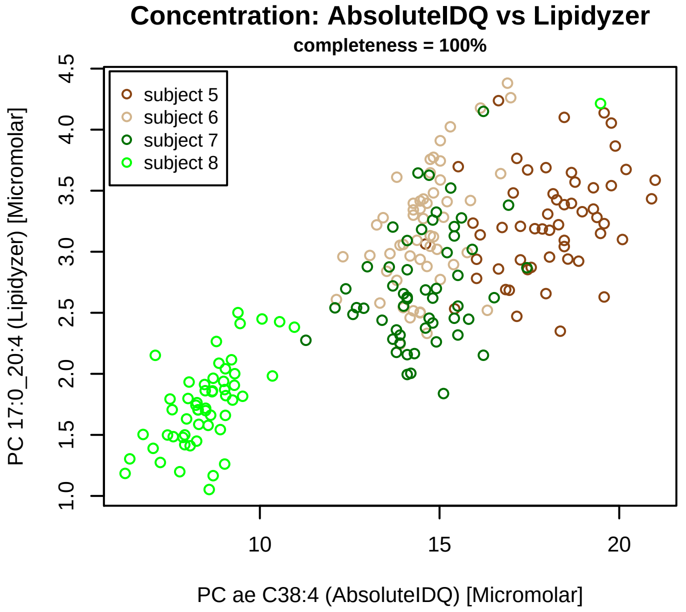

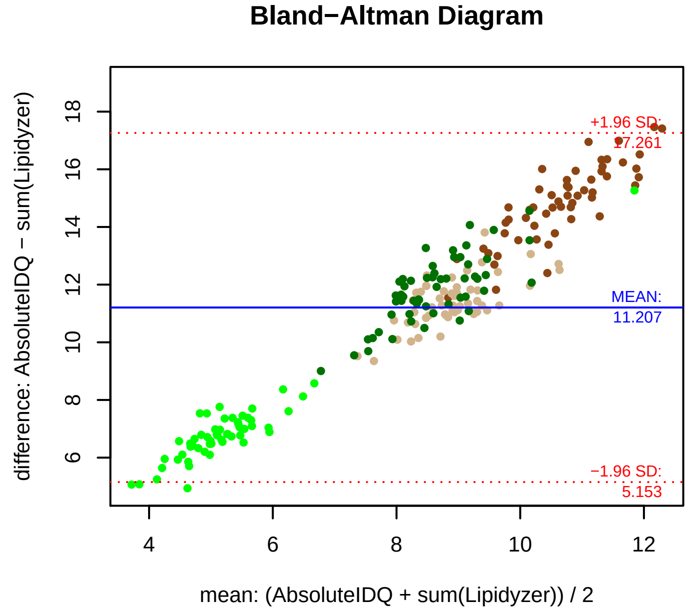

Stability of composition

PC 17:0_20:4 / PC ae C38:4
Shapiro−Wilk Test of log quotients, pv: 0.19617; OK
SW: ok; ANOVA: Comp. ~ Subject_ID −> pv = 0.01837; EQUAL
Wilcoxon: Challenge; fasting: 0.875; sport: 0.375; OLTT: 0.125

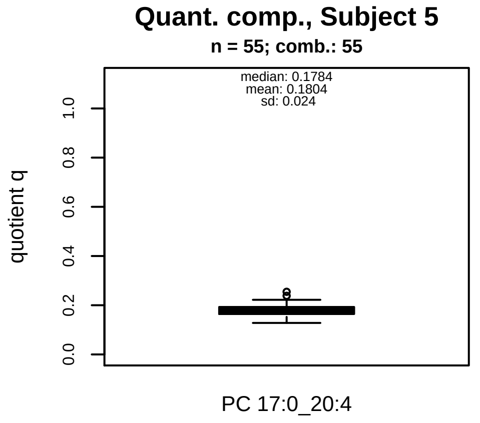

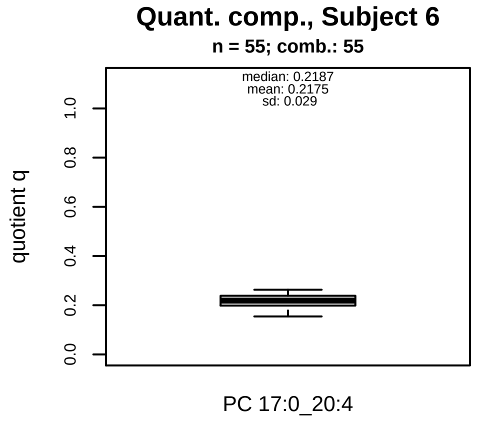

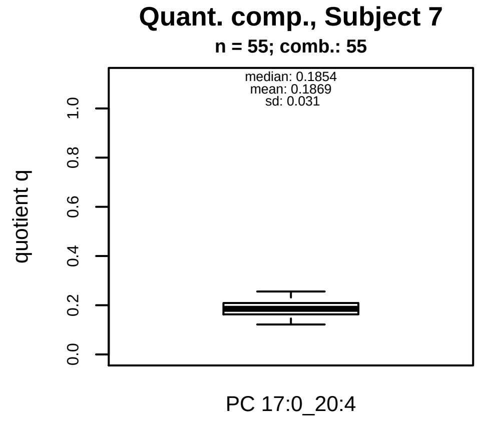

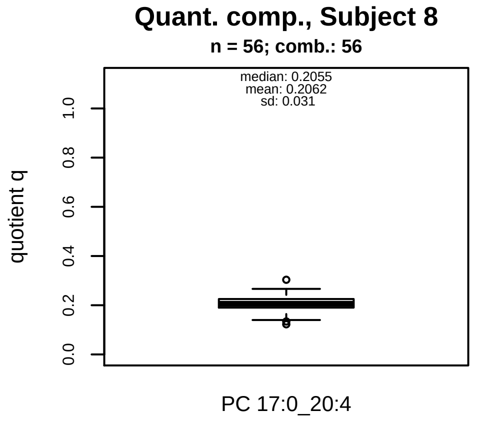

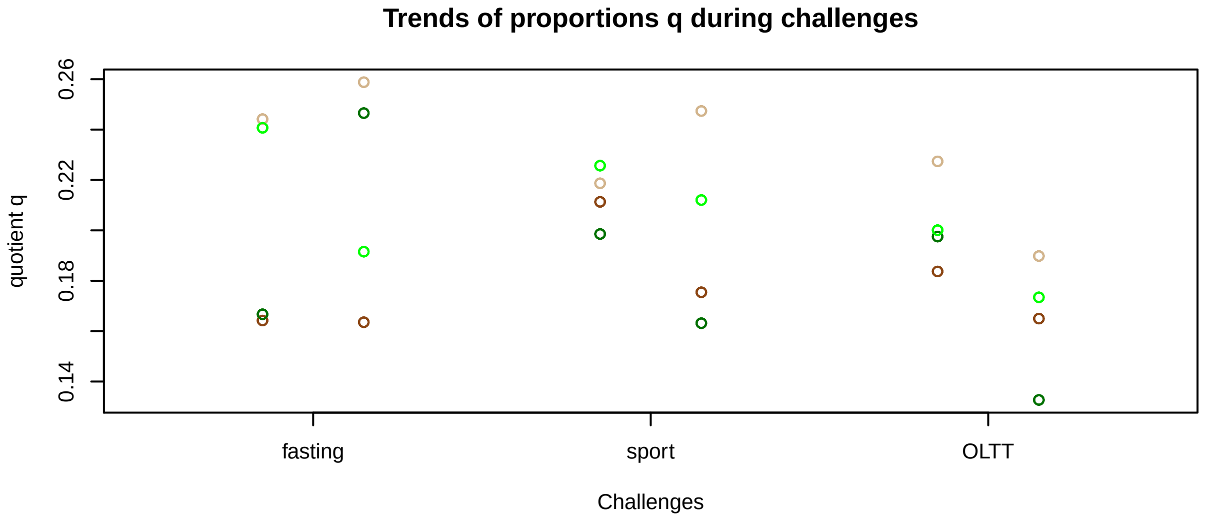

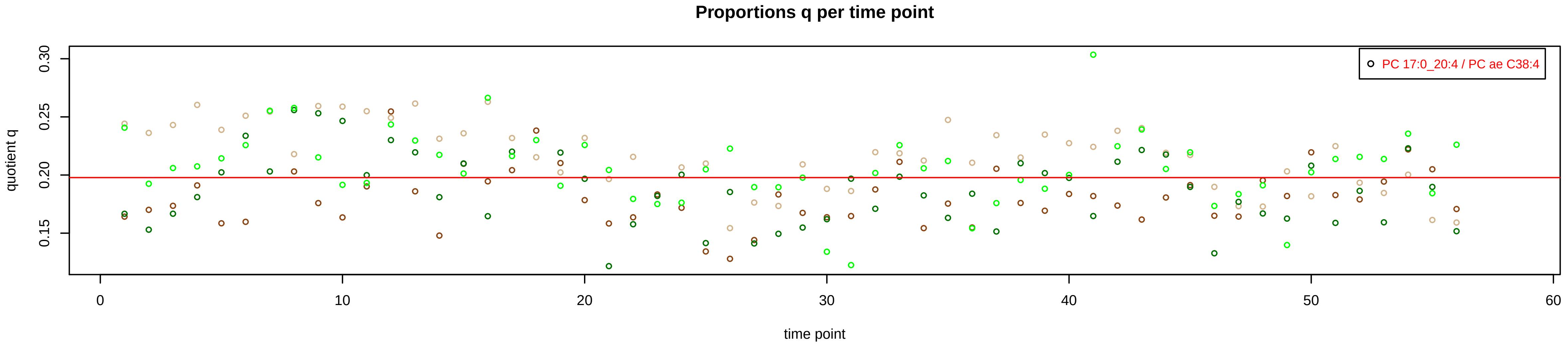

PC ae C38:5 = PC 15:0_22:5 + PC 17:0_20:5 + R

PC 15:0_22:5 excluded because of missingness > 75%

Semi−Quantitative composition within Lipidyzer

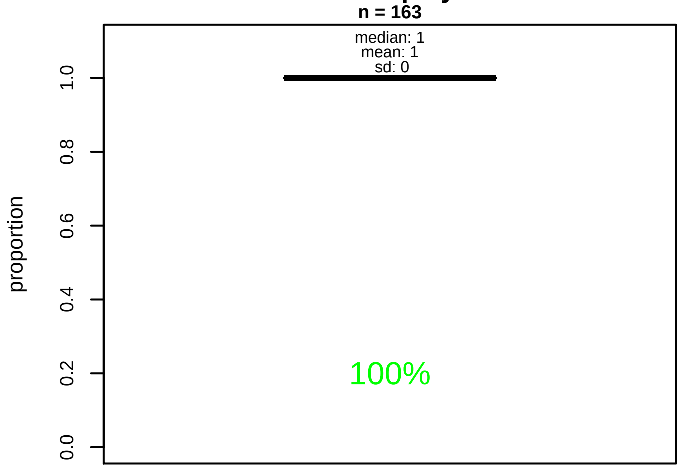

Semi−Quantitative composition quotients q

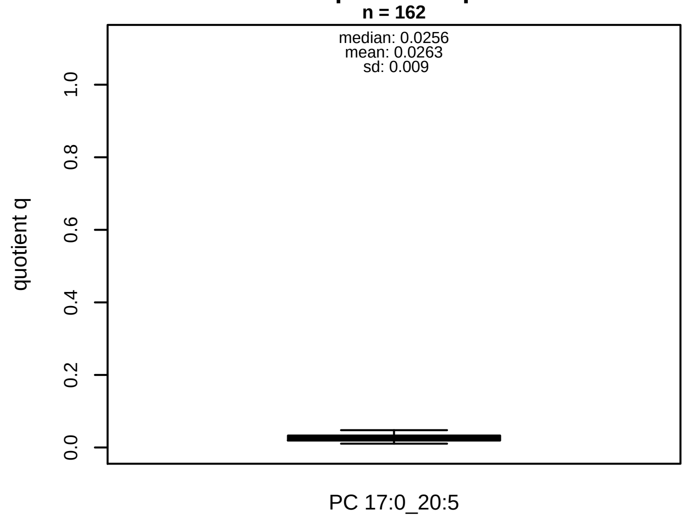

Semi−Quantitative composition: Lipidyzer

PC ae C38:5 consists of:
PC 17:0_20:5 100%
and of (not quantified):
PC 15:0_22:5, PC O−16:0_22:5, PC O−16:1_22:4, PC O−18:
PC O−18:1_20:4, PC O−18:2_20:3, PC O−18:4_20:1, PC 15:
PC 18:4_19:1
and further compounds

Composition: mean of proportions q

conc(PC ae C38:5) * 0.0263 = conc(PC 17:0_20:5) [var(q)=0.0263]
Percentiles: 5%−>0.0145, 25%−>0.0191, 75%−>0.0328, 95%−>0.0408

Linear model

```
PC ae C38:5 ~ b * ( PC 17:0_20:5 )
```

b = 3.44394

R^2 = 0.02174

Ranges

Measure	AbsoluteIDQ	sum(Lipidyzer)	delta
Min	12.5	0.2	12.3
Max	27.9	0.99	26.91
Mean	19.34	0.5	18.84
Median	19	0.5	18.5
SD	3.67	0.15	3.52

Concentration: AbsoluteIDQ vs Lipidyzer

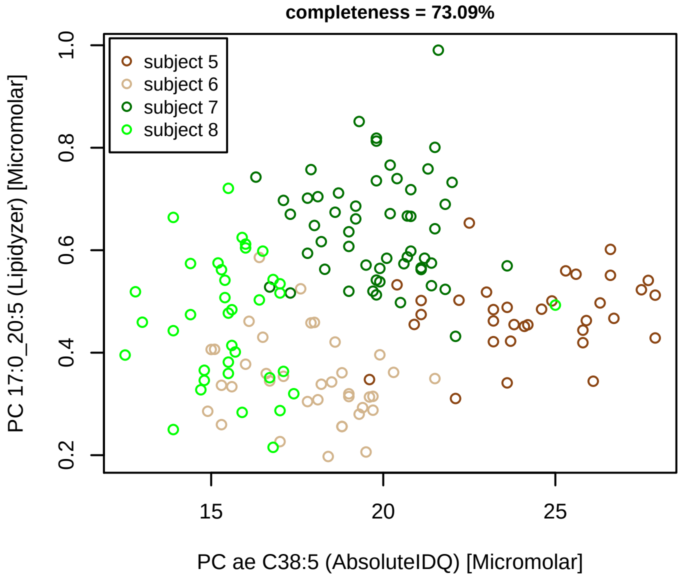

Bland−Altman Diagram

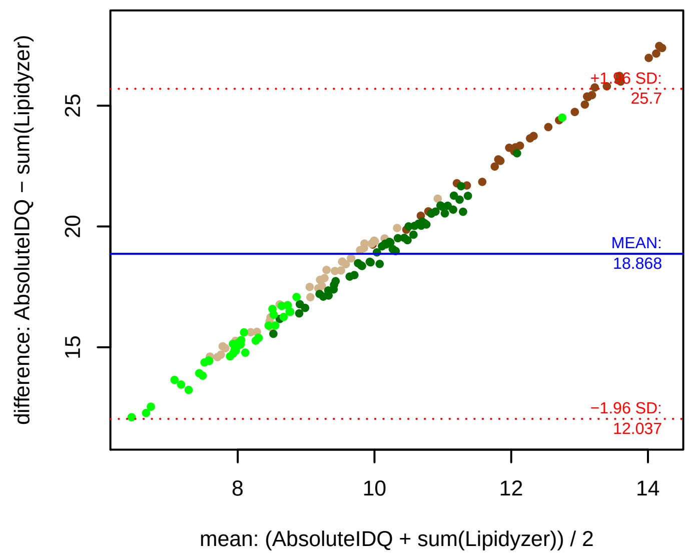

Stability of composition

PC 17:0_20:5 / PC ae C38:5
Shapiro−Wilk Test of log quotients, pv: 0.02234; OK
SW: ok; ANOVA: Comp. ~ Subject_ID −> pv = 0; DIFFERENCE
Wilcoxon: Challenge; fasting: 0.5; sport: 0.875; OLTT: 0.125

Quant. comp., Subject 5

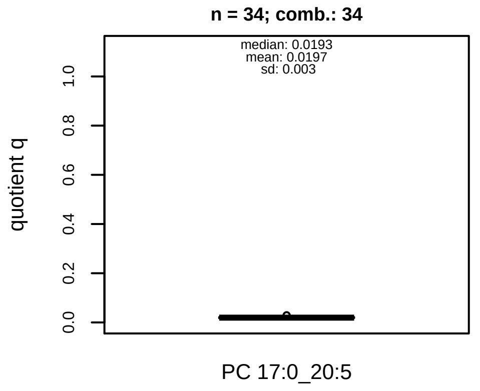

Quant. comp., Subject 6

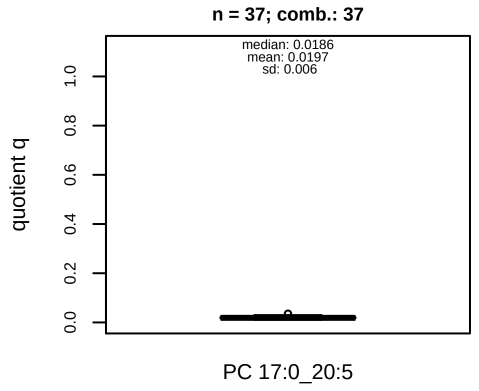

Quant. comp., Subject 7

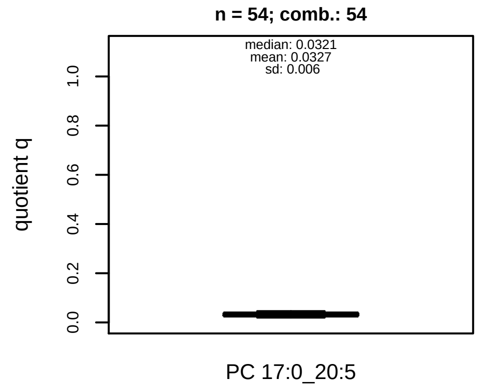

Quant. comp., Subject 8

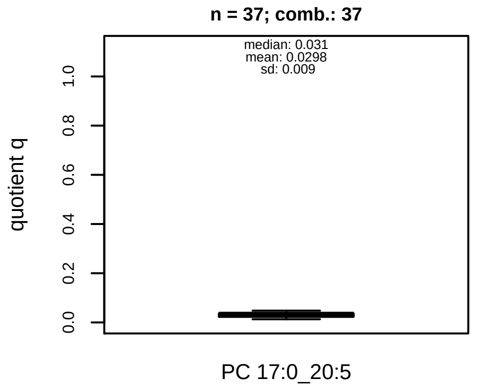

Trends of proportions q during challenges

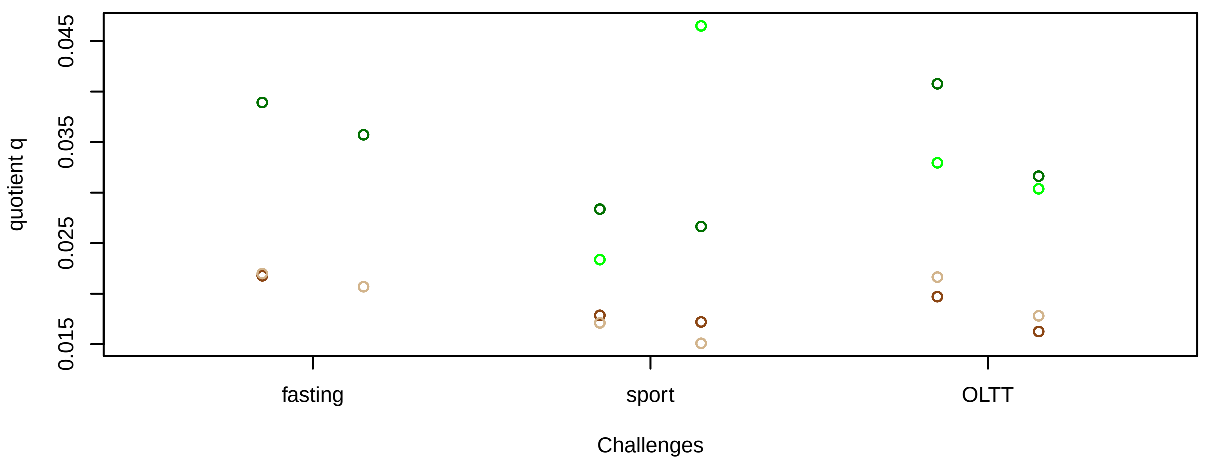

Proportions q per time point

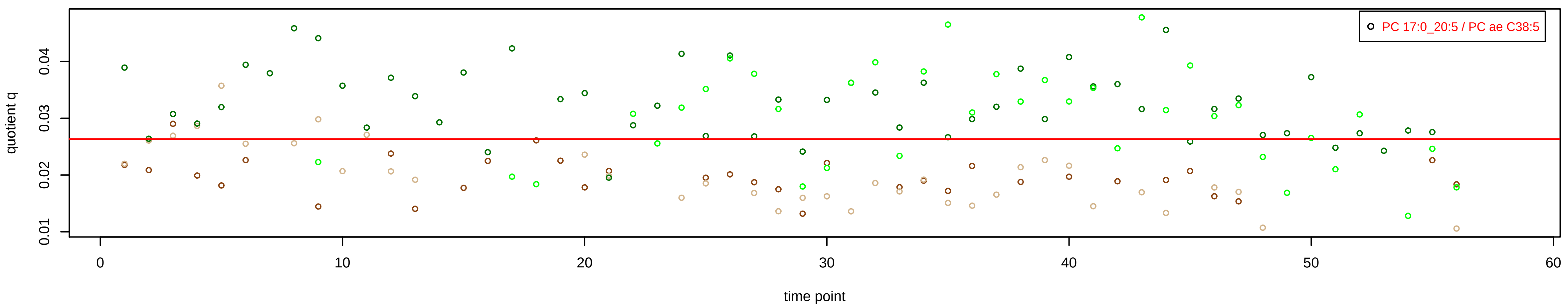

PC ae C38:6 = PC 15:0_22:6 + R

Semi−Quantitative composition within Lipidyzer
n = 188

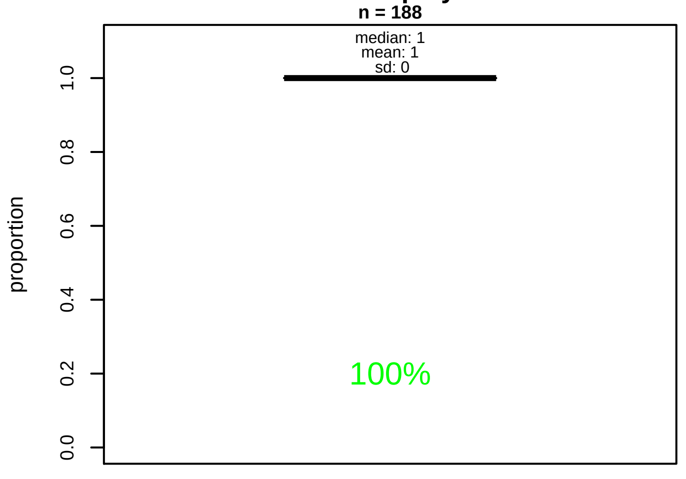

Semi−Quantitative composition quotients q
n = 186

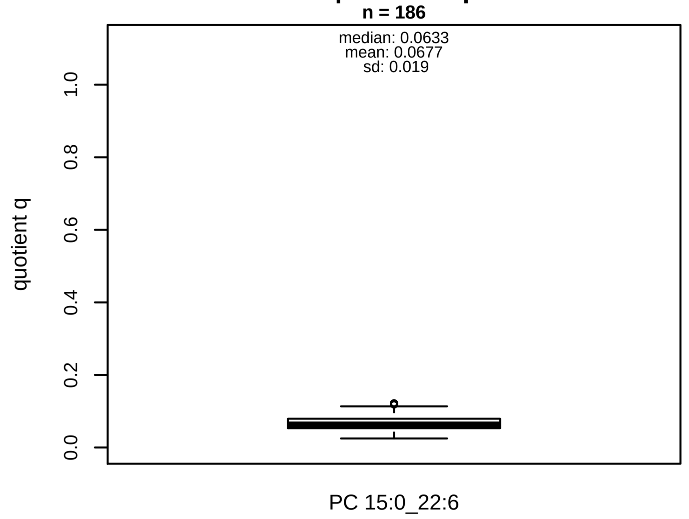

Semi−Quantitative composition: Lipidyzer

PC ae C38:6 consists of:
PC 15:0_22:6 100%
and of (not quantified):
PC O−16:0_22:6, PC O−18:1_20:5, PC O−18:2_20:5, PC 17:
PC 17:2_20:4
and further compounds

Composition: mean of proportions q

conc(PC ae C38:6) * 0.0677 = conc(PC 15:0_22:6) [var(q)=0.0677]
Percentiles: 5%−>0.0433, 25%−>0.0532, 75%−>0.0786, 95%−>0.1084

Linear model

```
PC ae C38:6 ~ b * ( PC 15:0_22:6 )
```

b = 5.55836

R^2 = 0.12603

Ranges

Measure	AbsoluteIDQ	sum(Lipidyzer)	delta
Min	5.29	0.17	5.12
Max	12.77	0.96	11.81
Mean	7.85	0.51	7.34
Median	7.32	0.51	6.81
SD	1.87	0.12	1.75

Concentration: AbsoluteIDQ vs Lipidyzer
completeness = 84.3%

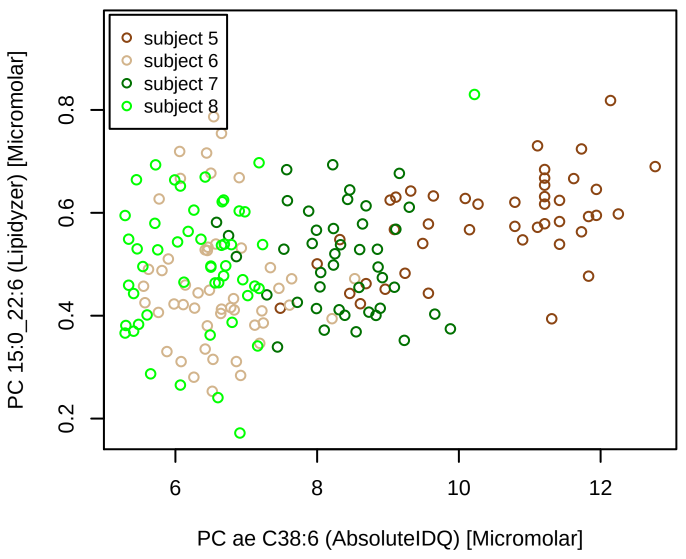

Bland−Altman Diagram

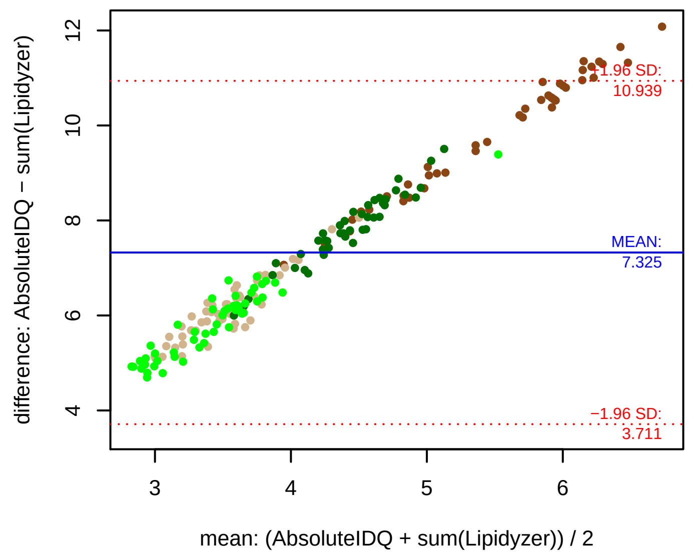

Stability of composition

PC 15:0_22:6 / PC ae C38:6
Shapiro−Wilk Test of log quotients, pv: 0.22571; OK
SW: no; Kruskal: Comp. ~ Subject_ID −> pv = 0; DIFFERENCE
Wilcoxon: Challenge; fasting: 0.75; sport: 0.625; OLTT: 0.5

Quant. comp., Subject 5
n = 44; comb.: 44

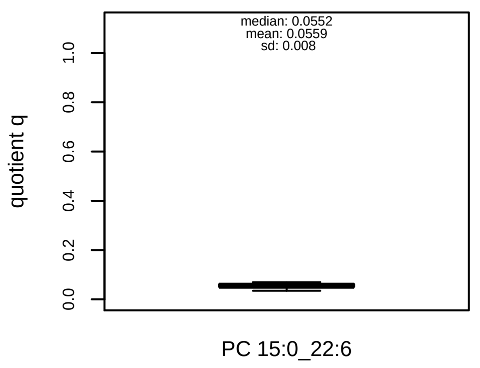

Quant. comp., Subject 6
n = 49; comb.: 49

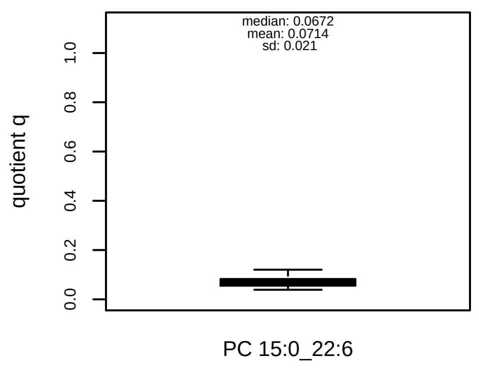

Quant. comp., Subject 7
n = 43; comb.: 43

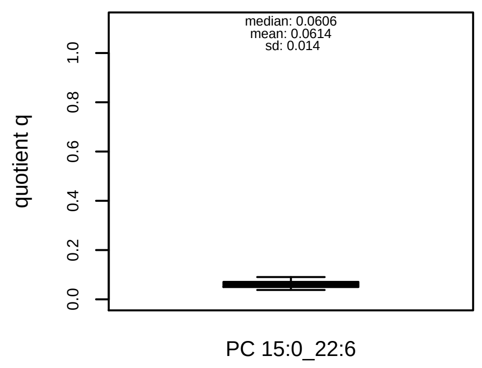

Quant. comp., Subject 8
n = 50; comb.: 50

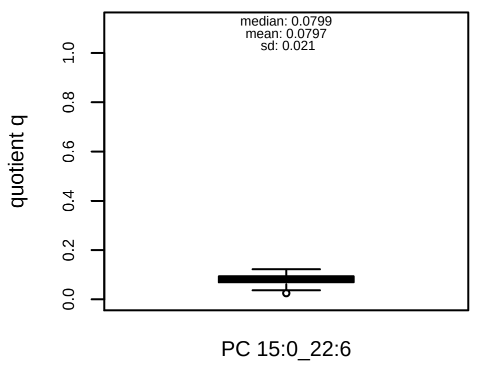

Trends of proportions q during challenges

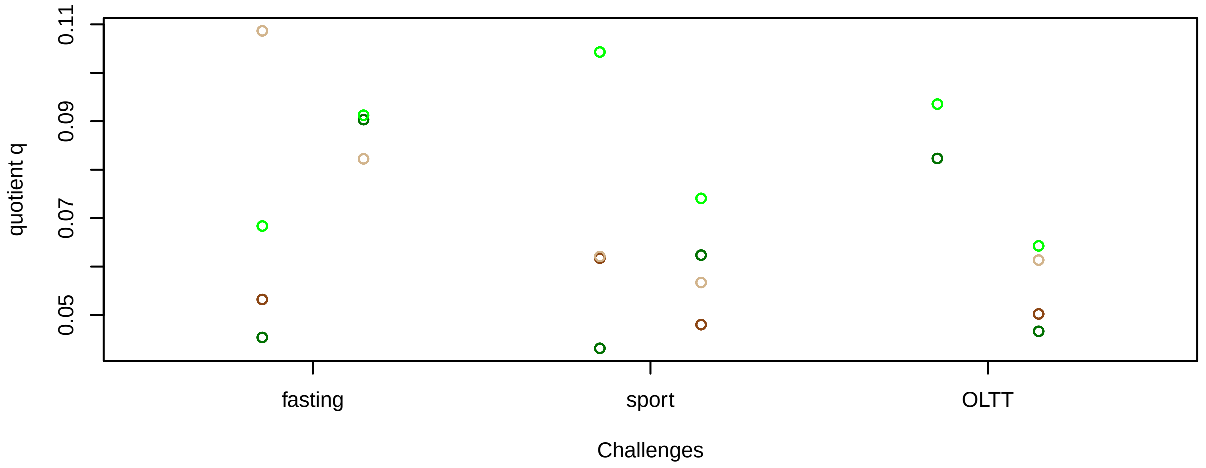

Proportions q per time point

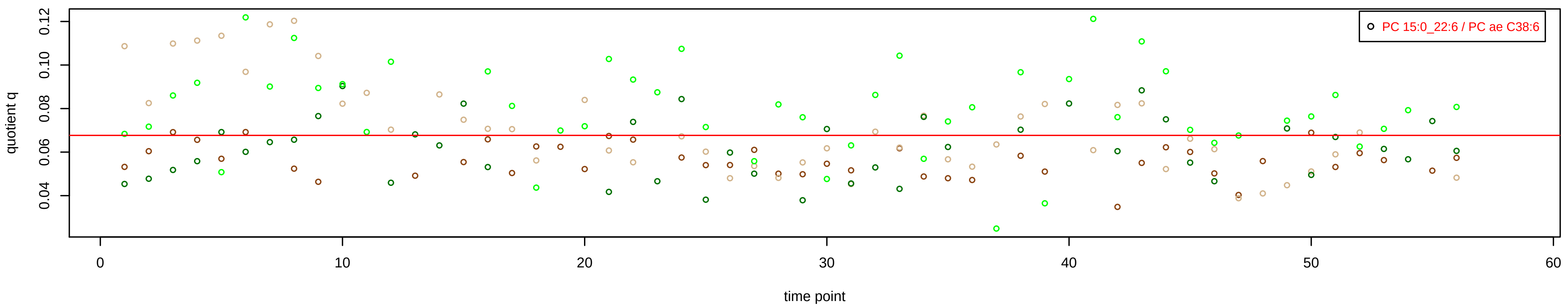

PC ae C40:1 = PC 18:2_22:6 + R

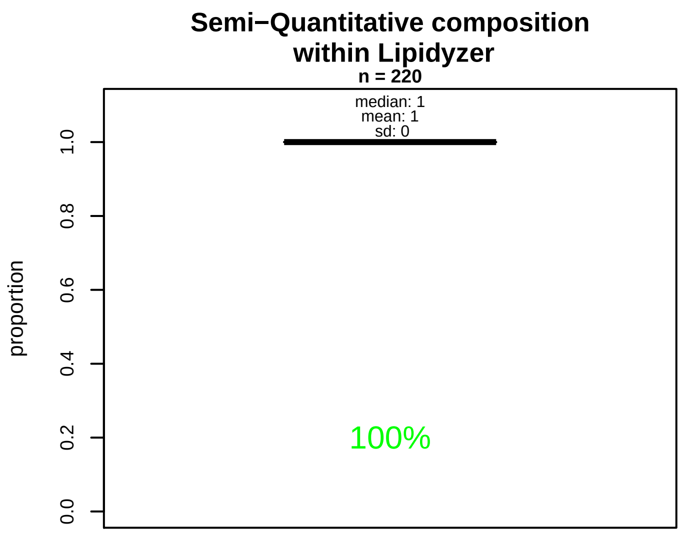

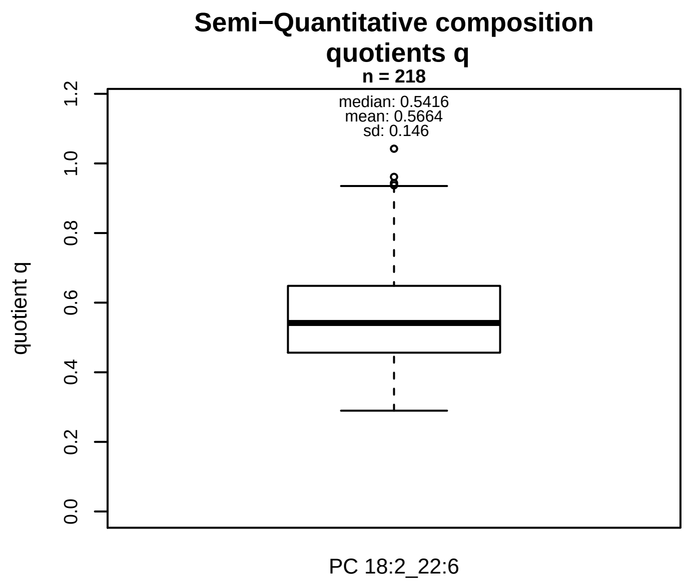

Semi−Quantitative composition: Lipidyzer

PC ae C40:1 consists of:
PC 18:2_22:6 100%
and of (not quantified):
PC O−18:0_22:1, PC O−18:1_22:0, PC O−20:0_20:1, PC 17:
PC 17:1_22:0, PC 18:1_21:0, PC 19:0_20:1, PC 19:1_20:0,
PC 20:4_20:4
and further compounds

Composition: mean of proportions q

conc(PC ae C40:1) * 0.5664 = conc(PC 18:2_22:6) [var(q)=0.5664]
Percentiles: 5%−>0.3782, 25%−>0.4566, 75%−>0.648, 95%−>0.8648

Linear model

```
PC ae C40:1 ~ b * ( PC 18:2_22:6 )
```

b = 0.44624

R^2 = 0.20278

Ranges

Measure	AbsoluteIDQ	sum(Lipidyzer)	delta
Min	1.27	0.5	0.77
Max	2.64	2.11	0.53
Mean	1.82	1.03	0.79
Median	1.8	0.96	0.84
SD	0.31	0.31	0

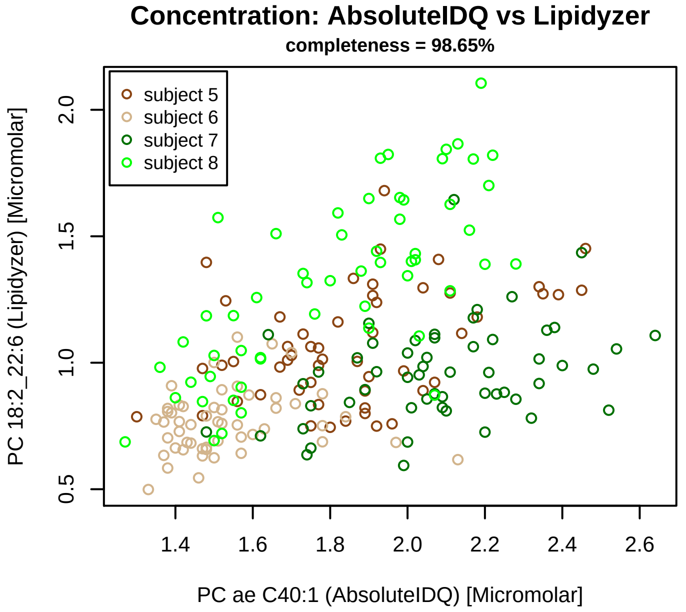

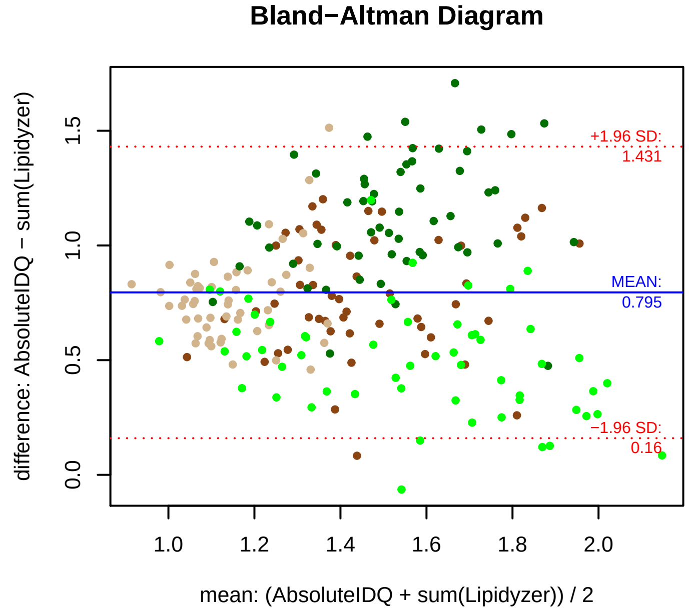

Stability of composition

PC 18:2_22:6 / PC ae C40:1
Shapiro−Wilk Test of log quotients, pv: 0.32761; OK
SW: ok; ANOVA: Comp. ~ Subject_ID −> pv = 6e−05; DIFFERENCE
Wilcoxon: Challenge; fasting: 1; sport: 1; OLTT: 0.875

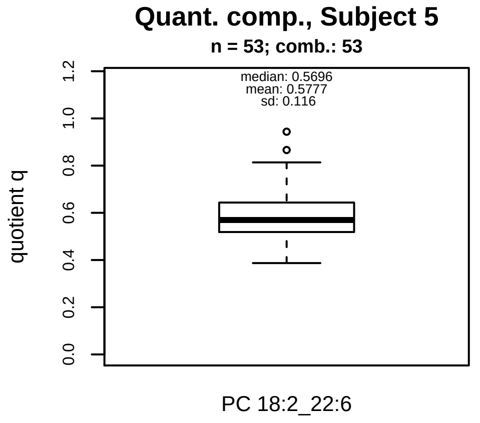

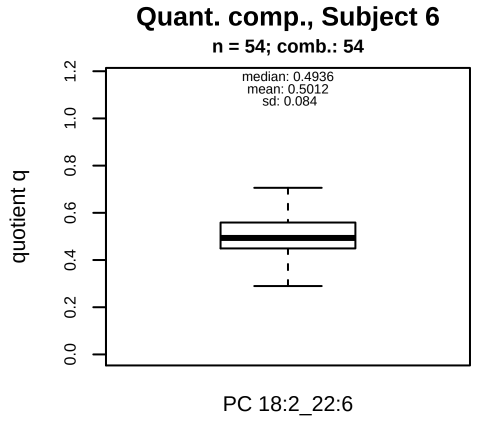

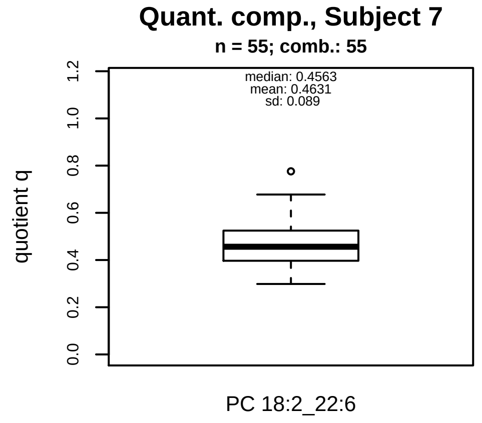

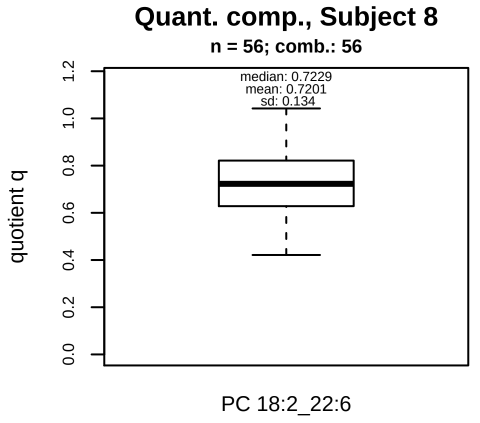

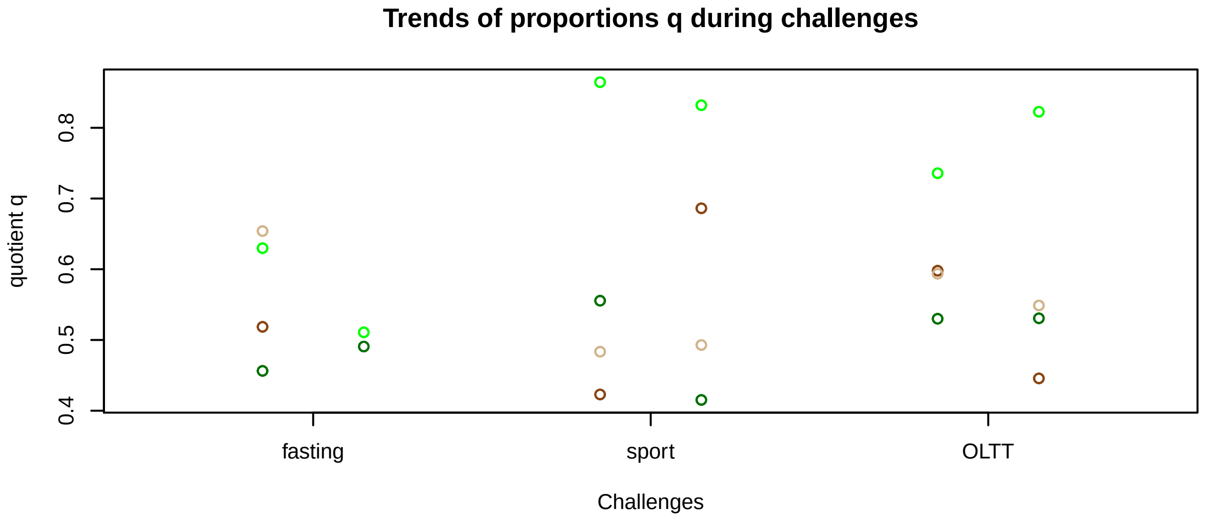

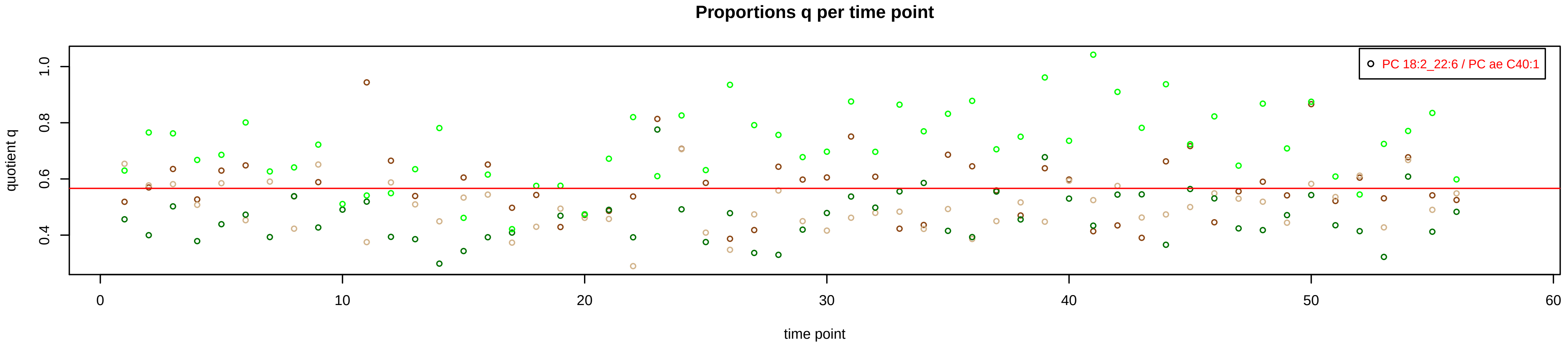

PC ae C40:2 = R

No independent Variable measured.

Qualitative composition

PC ae C40:2 consists of:
PC O−18:0_22:2,   PC O−18:1_22:1,   PC O−18:2_22:0,   PC O−[illegible]
PC O−20:1_20:1,   PC 17:0_22:2,   PC 17:1_22:1,   PC 17:2_22:[illegible]
PC 18:2_21:0,   PC 19:0_20:2,   PC 19:1_20:1,   PC 18:3_22:6,
[13C1]SM 43:2
and further compounds

PC ae C40:3 = R

No independent Variable measured.

Qualitative composition

PC ae C40:3 consists of:
PC O−18:1_22:2, PC O−18:2_22:1, PC O−20:0_20:3, PC O−
PC O−22:0_18:3, PC 17:1_22:2, PC 17:2_22:1, PC 18:3_21:(
PC 19:0_20:3, PC 19:1_20:2, PC 18:4_22:6, PC 20:5_20:5,
and further compounds

PC ae C40:4 = R

No independent Variable measured.

Qualitative composition

PC ae C40:4 consists of:
PC O−18:0_22:4, PC O−18:2_22:2, PC O−20:0_20:4, PC O−
PC 17:0_22:4, PC 17:2_22:2, PC 18:4_21:0, PC 19:0_20:4,
PC 19:1_20:3
and further compounds

PC ae C40:5 = PC 17:0_22:5 + R

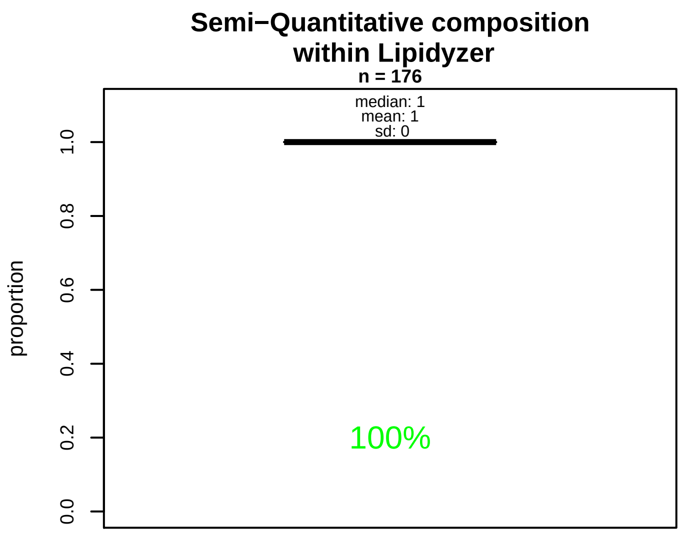

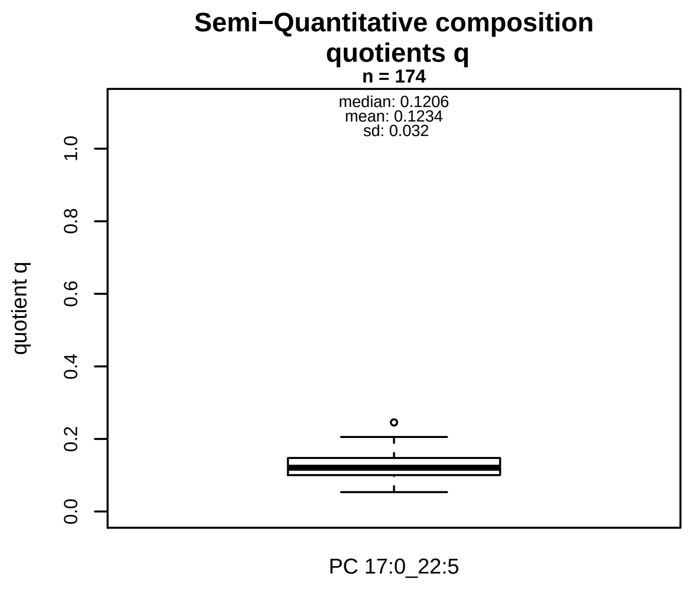

Semi−Quantitative composition: Lipidyzer

PC ae C40:5 consists of:
PC 17:0_22:5 100%
and of (not quantified):
PC O−18:0_22:5, PC O−18:1_22:4, PC O−20:0_20:5, PC O−
PC 16:0_23:5, PC 17:1_22:4, PC 19:0_20:5, PC 19:1_20:4,
and further compounds

Composition: mean of proportions q

conc(PC ae C40:5) * 0.1234 = conc(PC 17:0_22:5) [var(q)=0.1234]
Percentiles: 5%−>0.0708, 25%−>0.1003, 75%−>0.1471, 95%−>0.1735

Linear model

```
PC ae C40:5 ~ b * ( PC 17:0_22:5 )
```

b = 2.47007

R^2 = 0.19655

Ranges

Measure	AbsoluteIDQ	sum(Lipidyzer)	delta
Min	2.22	0.15	2.07
Max	5.64	0.85	4.79
Mean	3.66	0.45	3.22
Median	3.53	0.44	3.09
SD	0.73	0.13	0.6

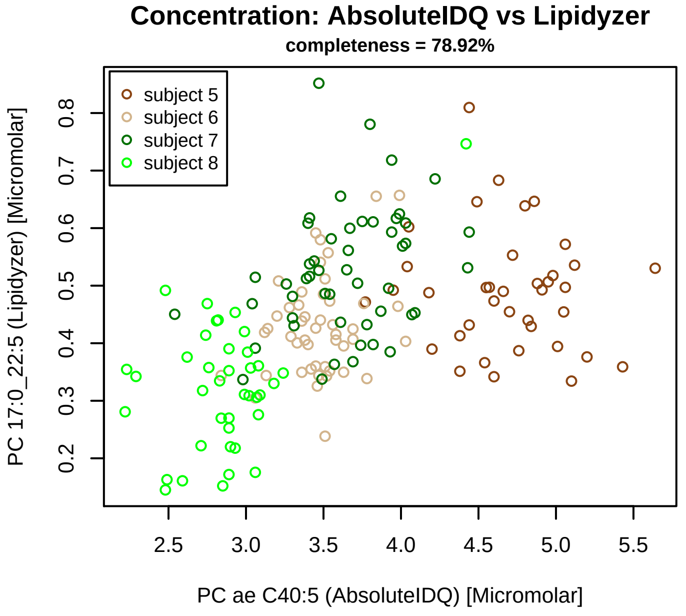

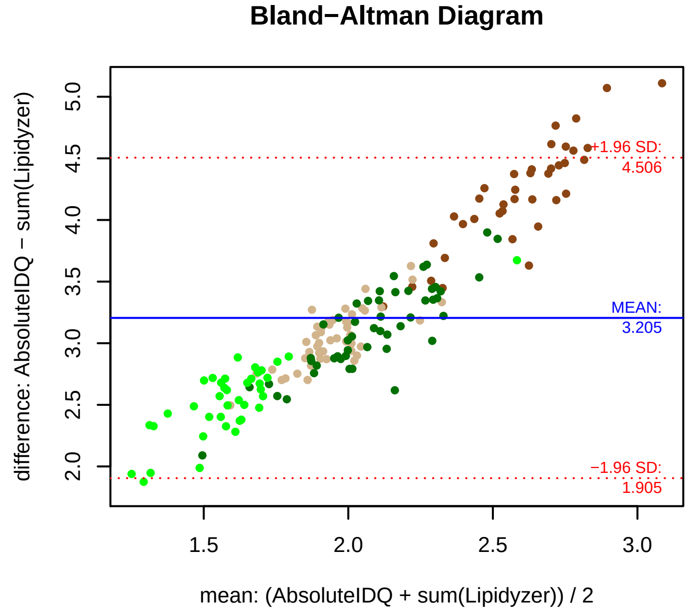

Stability of composition

PC 17:0_22:5 / PC ae C40:5
Shapiro−Wilk Test of log quotients, pv: 0.00815; NO
SW: ok; ANOVA: Comp. ~ Subject_ID −> pv = 0.09316; EQUAL
Wilcoxon: Challenge; fasting: 1; sport: 0.375; OLTT: 0.5

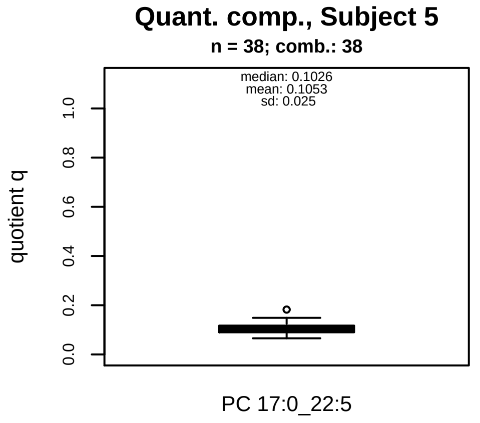

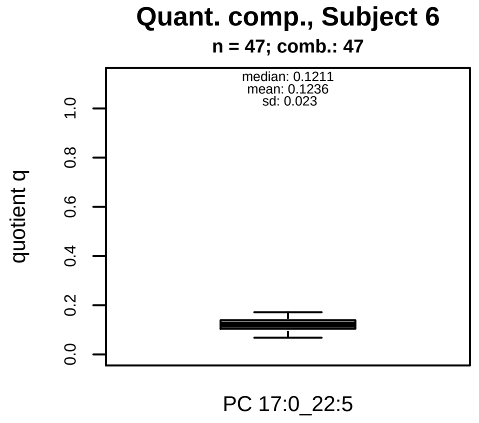

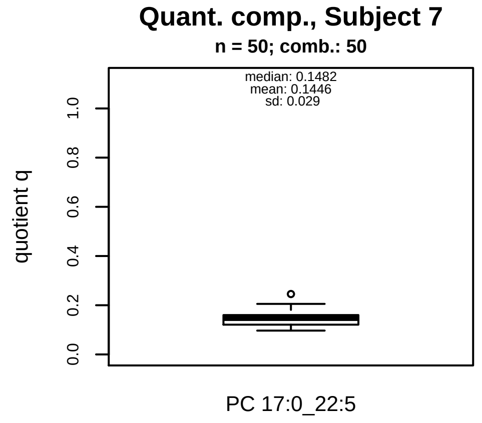

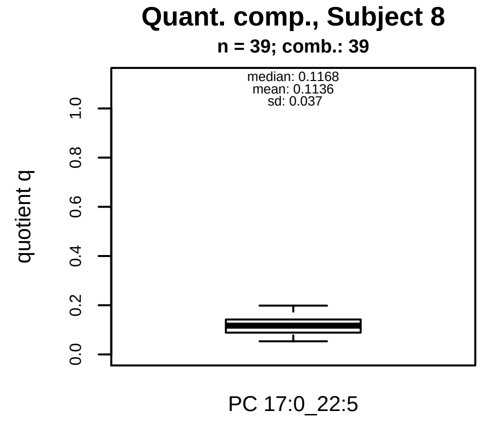

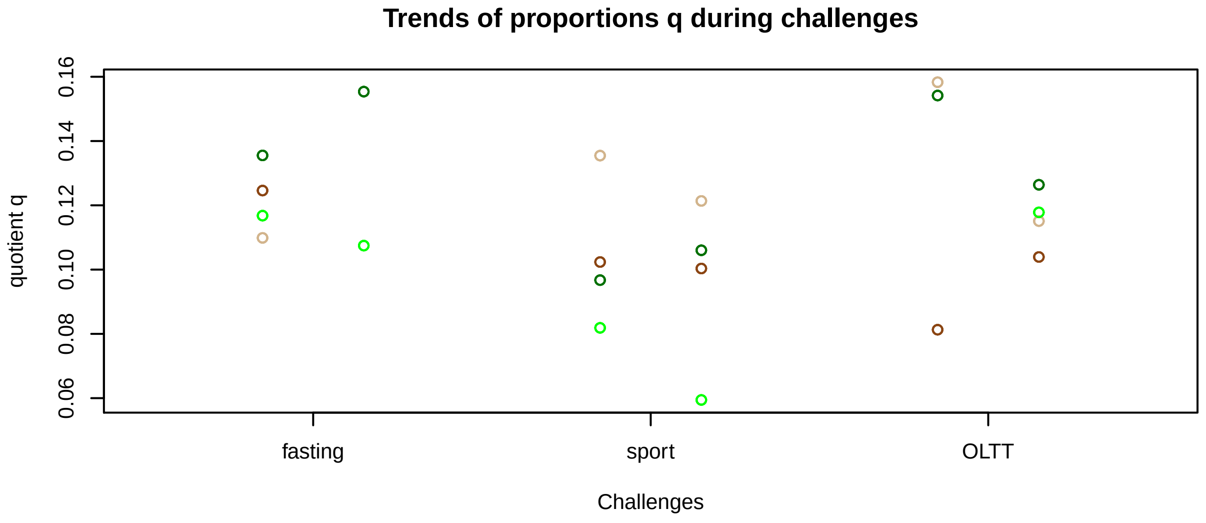

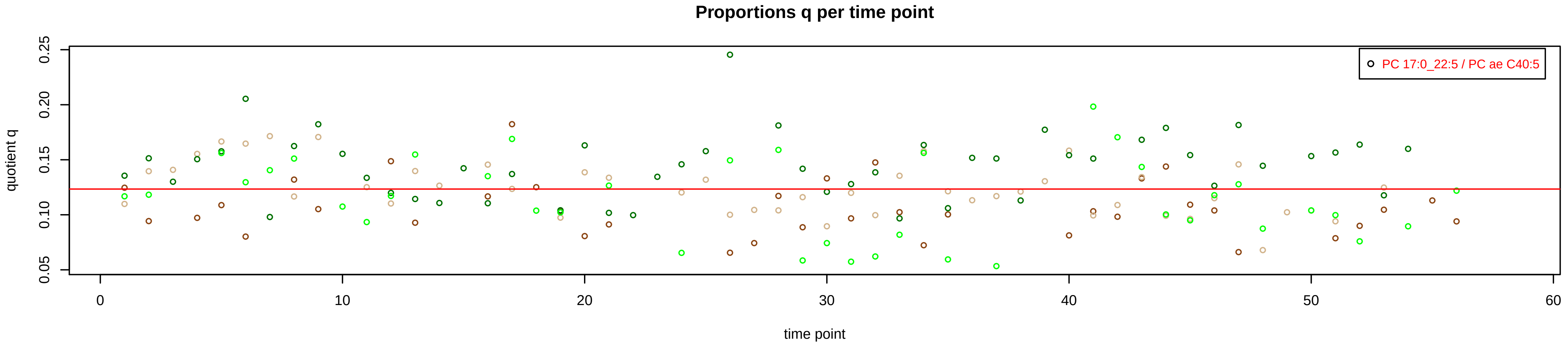

PC ae C40:6 = PC 17:0_22:6 + R

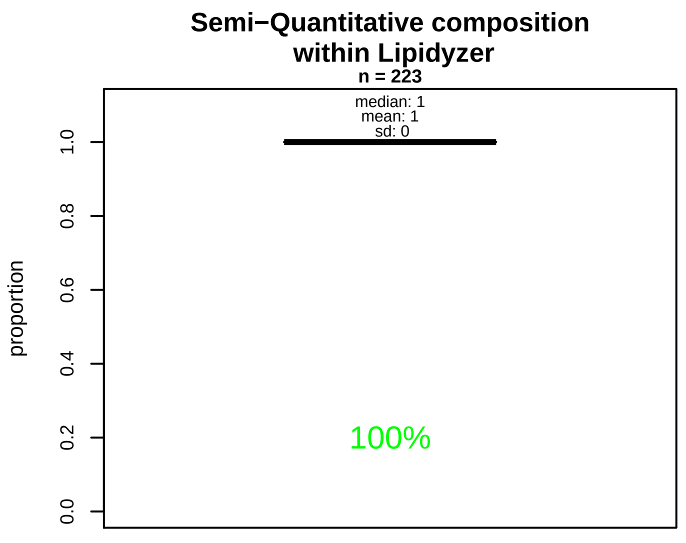

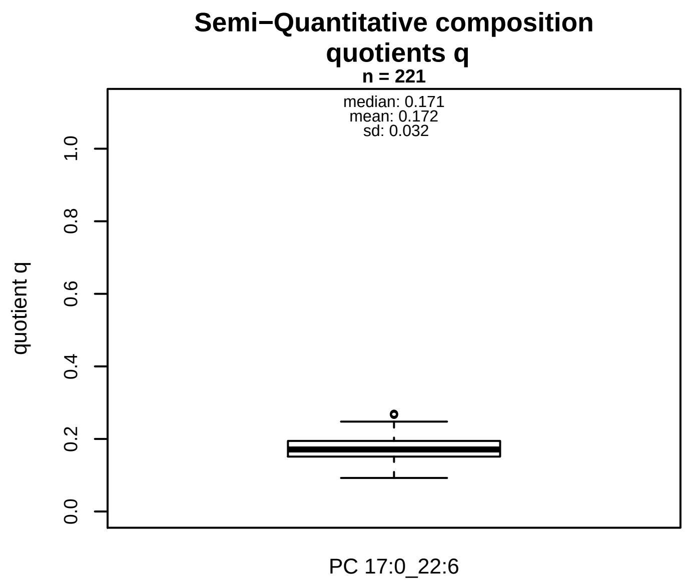

Semi−Quantitative composition: Lipidyzer

PC ae C40:6 consists of:
PC 17:0_22:6 100%
and of (not quantified):
PC O−18:0_22:6, PC O−18:2_22:4, PC O−20:1_20:5, PC 17:
PC 19:1_20:5
and further compounds

Composition: mean of proportions q

conc(PC ae C40:6) * 0.172 = conc(PC 17:0_22:6) [var(q)=0.172]
Percentiles: 5%−>0.1216, 25%−>0.1513, 75%−>0.1945, 95%−>0.2262

Linear model

```
PC ae C40:6 ~ b * ( PC 17:0_22:6 )
```

b = 3.28445

R^2 = 0.56301

Ranges

Measure	AbsoluteIDQ	sum(Lipidyzer)	delta
Min	3.54	0.43	3.11
Max	8.53	1.63	6.9
Mean	5.34	0.92	4.42
Median	5.01	0.87	4.14
SD	1.12	0.26	0.86

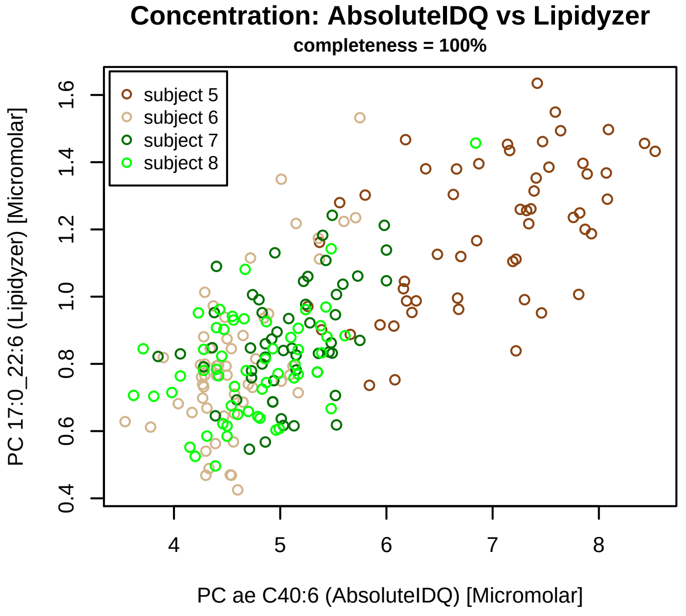

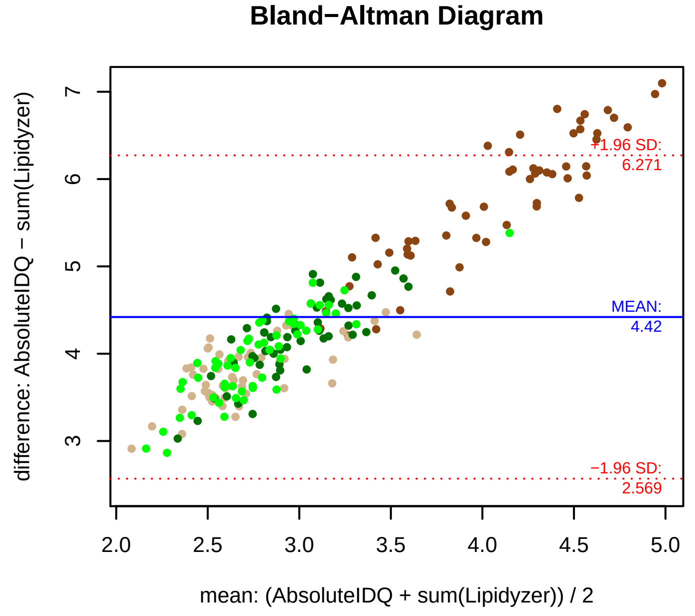

Stability of composition

PC 17:0_22:6 / PC ae C40:6
Shapiro−Wilk Test of log quotients, pv: 0.15729; OK
SW: ok; ANOVA: Comp. ~ Subject_ID −> pv = 0.41419; EQUAL
Wilcoxon: Challenge; fasting: 0.875; sport: 0.625; OLTT: 0.25

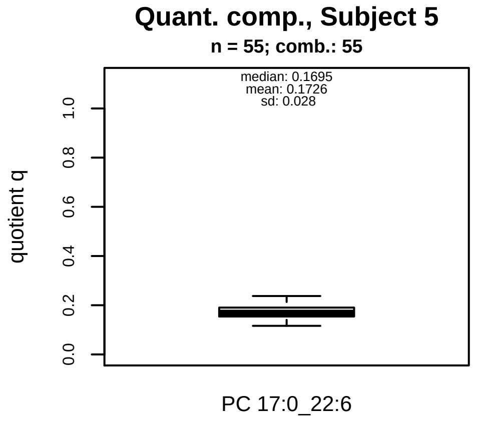

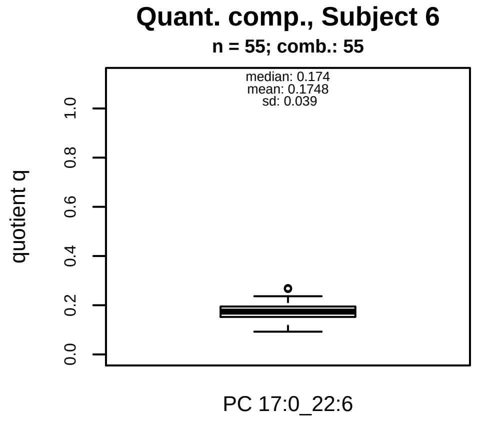

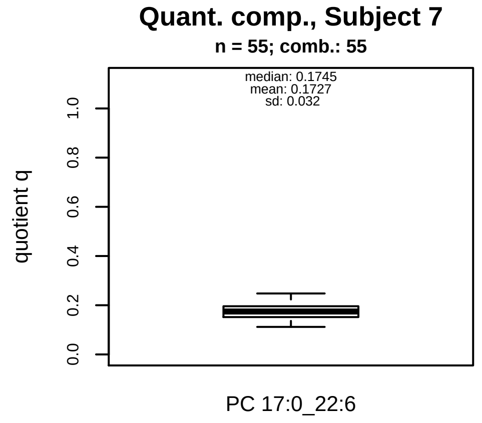

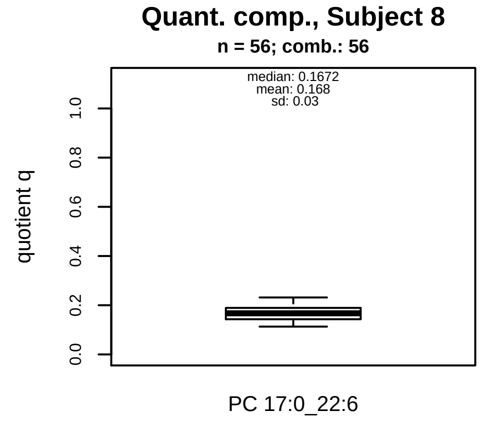

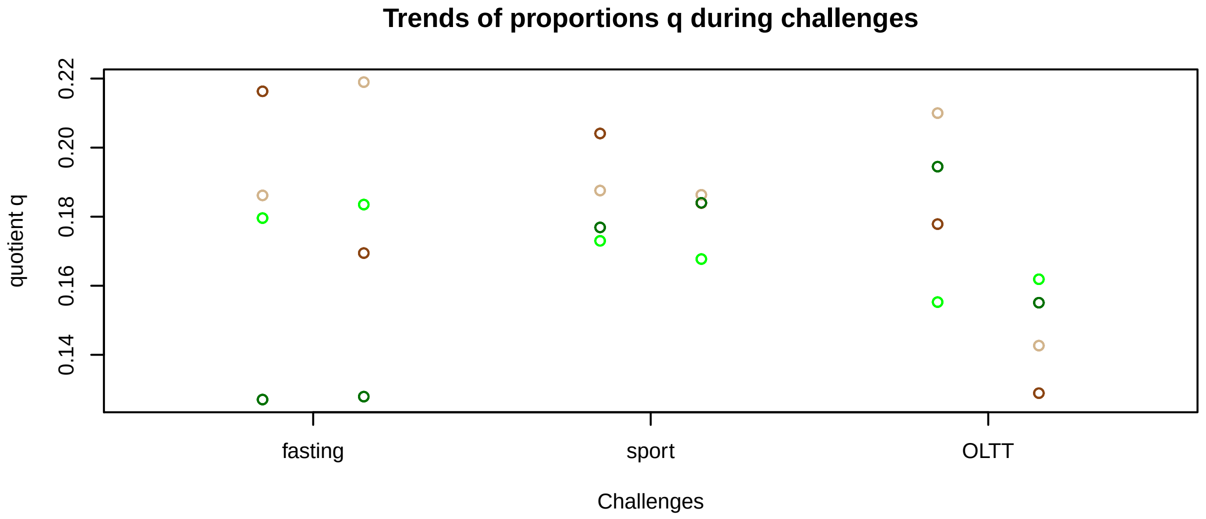

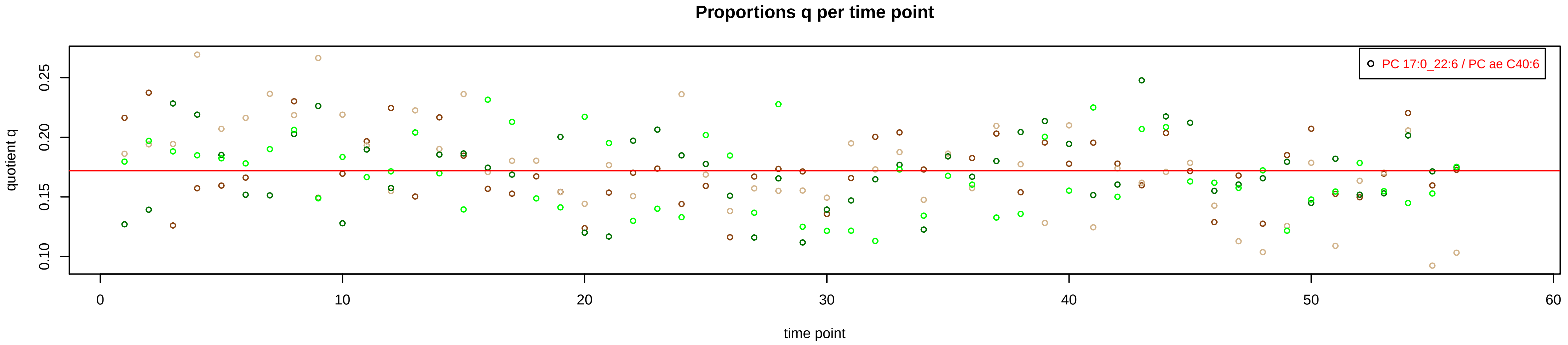

PC ae C42:0 = R

No independent Variable measured.

Qualitative composition

PC ae C42:0 consists of:
PC O−20:0_22:0, PC 19:0_22:0, PC 20:0_21:0, PC 23:0_18:[illegible]
PC 20:5_22:2
and further compounds

PC ae C42:1 = R

No independent Variable measured.

Qualitative composition

PC ae C42:1 consists of:
PC O−20:0_22:1, PC O−18:1_24:0, PC O−20:1_22:0, PC 19:
PC 19:1_22:0, PC 20:1_21:0, PC 20:2_22:6, PC 20:4_22:4,
and further compounds

PC ae C42:2 = R

No independent Variable measured.

Qualitative composition

PC ae C42:2 consists of:
PC O−20:0_22:2, PC O−18:2_24:0, PC O−20:1_22:1, PC O−
PC 19:0_22:2, PC 19:1_22:1, PC 20:2_21:0, PC 20:3_22:6,
PC 20:5_22:4
and further compounds

PC ae C42:3 = R

No independent Variable measured.

Qualitative composition

PC ae C42:3 consists of:
PC O−24:0_18:3, PC O−20:1_22:2, PC O−18:2_24:1, PC 21:
PC 19:1_22:2, PC 20:3_21:0, PC 20:5_22:5, PC 20:4_22:6,
and further compounds

PC ae C42:4 = R

Qualitative composition

PC ae C42:4 consists of:
PC O−20:0_22:4,   PC 19:0_22:4,   PC 20:4_21:0,   PC 20:5_22:[illegible]
and further compounds

No independent Variable measured.

PC ae C42:5 = R

No independent Variable measured.

Qualitative composition

PC ae C42:5 consists of:
PC 20:5_21:0
and further compounds

PC ae C44:3 = R

No independent Variable measured.

Qualitative composition

PC ae C44:3 consists of:
PC 22:4_22:6
and further compounds

PC ae C44:4 = R

No independent Variable measured.

Qualitative composition

PC ae C44:4 consists of:
PC O−24:0_20:4, PC O−22:1_22:3, PC O−22:2_22:2, PC 21:
and further compounds

PC ae C44:5 = R

No independent Variable measured.

Qualitative composition

PC ae C44:5 consists of:
PC 22:6_22:6
and further compounds

PC ae C44:6 = R

No independent Variable measured.

Qualitative composition

PC ae C44:6 consists of:
PC 21:0_22:6
and further compounds
